# Accurate, automated taxonomic assignment of genebank accessions: a new method demonstrated using high-throughput marker data from 10,000 *Capsicum *spp*.* accessions

**DOI:** 10.1007/s00122-023-04441-8

**Published:** 2023-09-11

**Authors:** M. Timothy Rabanus-Wallace, Nils Stein

**Affiliations:** 1 Leibniz Institute of Plant Genetics and Crop Plant Research (IPK), Gatersleben, Saxony-Anhalt Germany; 2 The University of Melbourne, Melbourne, Australia; 3 Center of Integrated Breeding Research (CiBreed), Georg-August-University, Göttingen, Germany

## Abstract

**Key message:**

We demonstrate how an algorithm that uses cheap genetic marker data can ensure the taxonomic assignments of genebank samples are complete, intuitive, and consistent—which enhances their value.

**Abstract:**

To maximise the benefit of genebank resources, accurate and complete taxonomic assignments are imperative. The rise of genebank genomics allows genetic methods to be used to ensure this, but these need to be largely automated since the number of samples dealt with is too great for efficient manual recategorisation, however no clearly optimal method has yet arisen. A recent landmark genebank genomic study sequenced over 10,000 genebank accessions of peppers (*Capsicum *spp.), a species of great commercial, cultural, and scientific importance, which suffers from much taxonomic ambiguity. Similar datasets will, in coming decades, be produced for hundreds of plant taxa, affording a perfect opportunity to develop automated taxonomic correction methods in advance of the incipient genebank genomics explosion, alongside providing insights into pepper taxonomy in general. We present a marker-based taxonomic assignment approach that combines ideas from several standard classification algorithms, resulting in a highly flexible and customisable classifier suitable to impose intuitive assignments, even in highly reticulated species groups with complex population structures and evolutionary histories. Our classifier performs favourably compared with key alternative methods. Possible sensible alterations to pepper taxonomy based on the results are proposed for discussion by the relevant communities.

**Supplementary Information:**

The online version contains supplementary material available at 10.1007/s00122-023-04441-8.

## Introduction

The importance of genebanks for the collection, maintenance, and exploitation of plant genetic diversity has been recognised for well over a century (Mascher et al. 2019). Accurately characterising and describing this genetic diversity is key to its exploitation. The rise of genebank genomics, and associated herbarium genomics (Bakker et al. 2020), has liberated this effort from its former dependence on measurable phenotype-based approaches such as morphology, phenology, cytogenetics, and chemical assays, allowing direct interrogation of the genetic code of, in notable recent cases, tens of thousands of accessions.

Linnean taxonomic classifiers have always served a useful purpose in this regard, but the Linnean system of nested classifiers with defined levels does not map neatly onto the realities of evolution and reproductive biology, and hence the classifications do not always reliably reflect true biological divisions (such as reproductive boundaries or cladistic monophyly). This generates much discussion, especially in cases such as hybridising species, ring species, or instances of horizontal gene transfer, where the Linnean system struggles most to act as a meaningful classifier. Plants, being particularly prone to such cases, often suffer taxonomic ambiguity (Rouhan and Gaudeul 2014). It is generally agreed, nevertheless, that taxonomic classifiers have great utility, functioning at a very minimum as shorthand labels for groups of genetically- or morphologically-similar individuals, whether or not they are truly monophyletic or reproductively-isolated groups. Typically, researchers working with members of a taxon gain an understanding of how well the assignments reflect, or fail to reflect, and biological factors of importance to their research. Ultimately, taxonomic assignments may variously function as reliable and convenient indicators of reproductive boundaries, crossability, phenotypic and physiological traits, cytology, agricultural value, and geographic origin, just to name a few. Genebank genomics—the sequencing of genebank accessions *en masse* for genomics applications such as population genetics, association studies, and enhanced genebank curation—is a rapidly growing field, with recent landmark studies sequencing several thousand or tens of thousands of accessions. Accurate and complete taxonomic assignments are imperative not just for maximising the benefit of genebank resources, but also to ensuring their exploitation, since researchers of all kinds have a tendency to focus on germplasms with complete passport information (Meyer 2015), resulting in neglecting a large fraction of the stored materials where documentation is less complete but may still be favourable for breeding.

One function of these studies is to reveal and correct taxonomic assignments or to add assignments where they are missing. The problem of automating taxonomic assignment is not unique to genebank genomics. For example, metagenomic studies face the problem of estimating taxon abundances using a mixed sample (Thomas et al., 2012), while bacterial sequence databases ensure taxonomic assignment consistency by automatically checking new submissions against a reference database of type assemblies (Ciufo et al. 2018). Genebank taxonomic correction is, however, a fairly novel task. It consists of using genetic data from a large number of accessions that already have assignments recorded in their passport information, using those assignments to ascertain how these assignments map onto the genetic diversity space of the accessions, and then identifying accessions that do not conform with this mapping. The data one corrects is also the source of information by which correction is achieved, something common to both smoothing and clustering algorithms. The process has occasionally been done manually where very few corrections have been called for (e.g., Singh et al. 2019; Milner et al. 2019). Multiple methods for automated taxonomic assignment, including some borrowed from literature on species barcoding and metabarcoding, have been suggested and tried in comparative studies (see van Bemmelen van der Plaat et al. 2021 for summary; Austerlitz et al. 2009; Weitschek et al. 2014). No all-round best tool has been established, though *k*-nearest-neighbours (in particular with $k = 3$) classifiers are perhaps the best candidate and generally performed well on test datasets despite their sensitivity to parameter selection and to variation in sample sizes between different taxa in the sample (van Bemmelen van der Plaat et al. 2021).

A recent study on *Capsicum* accessions from a pan-Eurasian selection of genebanks (Tripodi et al. 2021) featured over 10,000 accessions in total, valorising the dataset with investigations into genetic predictors of agriculturally-relevant traits, and the prominent influence of human trade and migration on the distribution of peppers around the world. Domesticated peppers have significant economic importance, their visual and gustatory diversity, including the characteristic pungency of many varieties, making them a staple component of cuisines from across the globe. Close to 40 million tonnes of peppers were grown in 2020, with Africa, Eurasia, and the Americas all contributing significantly to the total global yield (FAOSTAT, accessed 11/8/2022). These cultivated peppers fall mainly within the species *C. annuum* (e.g. bell pepper, cayenne pepper, jalapeno), with significant contributions from *C. frutescens*, *C. chinense*, and *C. pubsescens*, and minor contributions from a host of other semi-domesticated and wild divisions such as *C. baccatum*, *C. chacoense*, and *C. eximium*. The difficulties involved in *Capsicum* taxonomy are reviewed in detail by Eshbaugh (2012), who emphasises that inconsistent assignments frequently occur, with boundaries between even major established *Capsicum* divisions such as *C. frutescens* and *C. chinense* often being unclear. As noted by Eshbaugh, this is partially a consequence of a lack of firm criteria upon which the divisions are based, though fruit shape, pungency, chromosome number, and more subtle phenotypic characters such as the morphology of the corolla, have all played significant parts in various taxonomic delineation schemes. With large and active communities of breeders and researchers accessing genebank pepper accessions and working with them to produce new pepper varieties with desirable agricultural properties and/or cosmetic/culinary appeal, updating the taxonomic assignments of accessions to reflect true genetic demes is a highly desirable and productive pursuit.

**Fig. 1:**
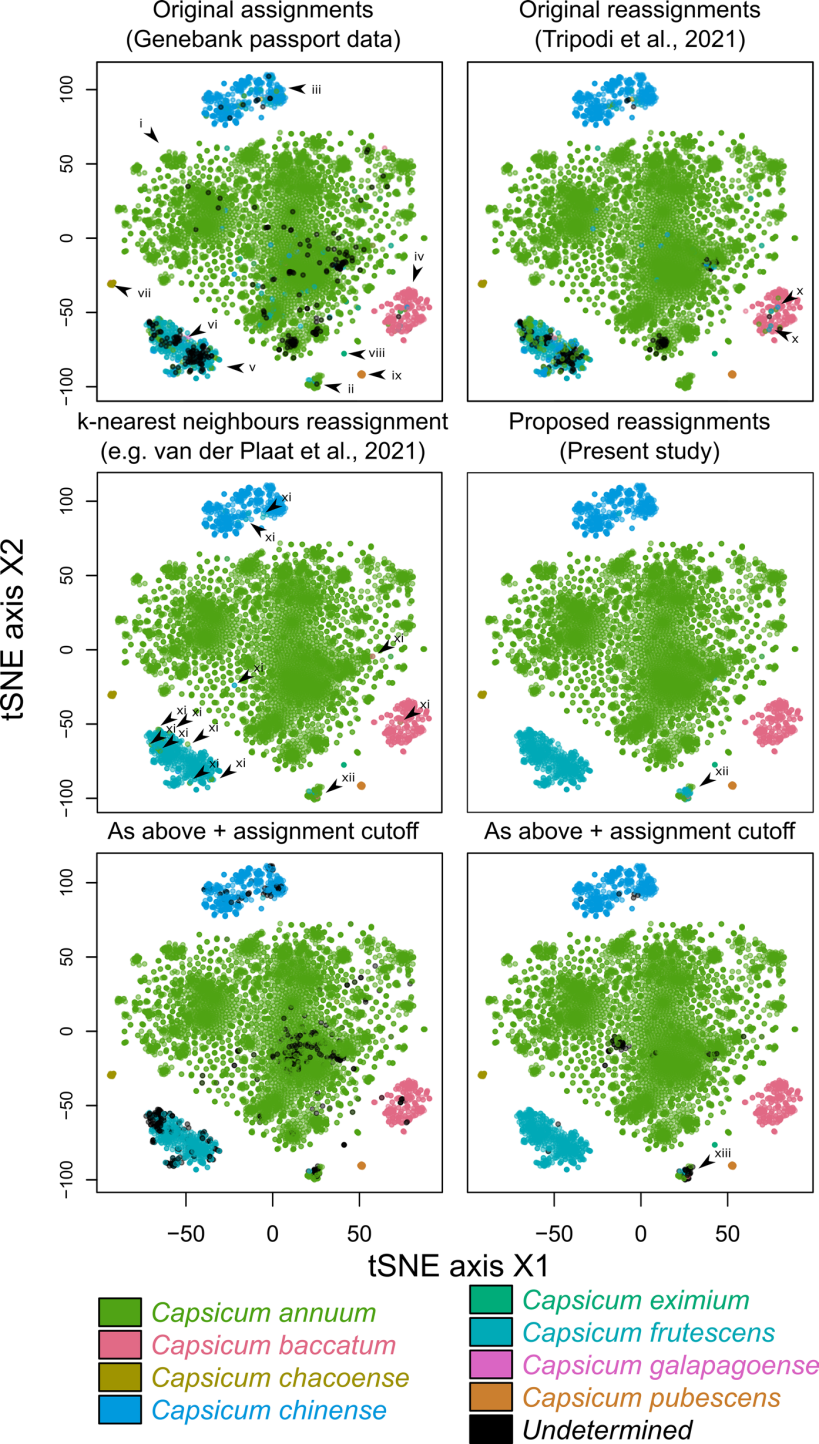
t-SNE plots representing the relationships among the G2P-Sol pepper accessions used in the study (trimmed dataset—see “Methods”) and their classifications under different schemes, as represented with t-SNE plots (calculated from IBS distances, perplexity = 30). Markers labelled with Roman numerals are referred to in the main text. For *k*-nearest-neighbours, van Bemmelen van der Plaat (2021) recommend *k* = 3 in general. We use $k = 6$, which we judged to give the best result possible for this dataset (see Supplementary Materials for a range of values). At $k = 7$, *C. eximium* (marker ix) are reassigned as *C. chacoense.* The unassignment cutoff $r$ is set to 4/6. Parameters for the kernel-based method are $\lambda = 320$, $d = 0.2$, $r = 0.66$, $k = \infty$

The Tripodi et al. (2021) study applied a clustering-based method to suggest alternative taxonomic classifications for putatively-misclassified *Capsicum* accessions: briefly, accessions were clustered using a UPGMA algorithm with an arbitrarily-defined distance cutoff, and minority members of clusters dominated by a single majority taxonomic assignment (80% of members, in this case), were all given the majority assignment. This method suffers from several flaws, most critically, sensitivity to over-representation of members of one taxon, which is also suffered by the methods reviewed by van Bemmelen van der Plaat et al. (2021): taxa comprising many samples are by default more likely to dominate clusters, no matter what the cluster size. The capsicum dataset, being dominated by the species *Capsicum annuum*, is particularly vulnerable to methodological biases of this kind. In addition, the composition of clusters is somewhat arbitrary, and results would be expected to differ if the sampled taxa were altered. Furthermore, appropriate visual inspection methods reveal that many taxa clearly associated with a distinct deme having some obvious majority assignment were not given the obvious assignment, most likely owing to their falling—mainly by chance—into a heterogeneous cluster. While better optimisation of the cluster size cutoff and majority cutoff parameters may have helped to alleviate this effect, it is ultimately a result of the clustering-based method itself, and without employing any visualisation tools for the specific purpose of assessing how intuitive the assignment results were, the study ultimately left much room for improvement on the taxonomic assignment front (see section on visualisation in methods; Fig. 1).

We present here a method of automatically producing pragmatic taxonomic assignments for genebank *Capsicum* accessions that aims to align with human intuition, and to possess broader utility for genebank genomics projects.

## Methods

### A new, hybrid approach

Taxonomic assignments are typically based on various measures of similarity that either suit the conventions of the research community (such as morphology, cytology, genetics, phenology, reproductive biology, evolutionarily factors, and so on), or which are simply convenient. These assignments, we argue, ought to be respected, but can be occasionally modified for pragmatic reasons to ensure better conformity with the expectation that membership in a taxon entails genetic similarity with other members of the same, but does not exclude the possibility of paraphyletic groupings.

We therefore suggest a method that examines each individual in the dataset and suggests an assignment based on the assignments of the individuals nearest to it, based on identity-by-state (IBS) genetic distance, similar to the *k*-nearest-neighbours approach that achieved equal-best all-round success in the comparative study of van Bemmelen van der Plaat (2021), by creating a hybrid method that combines *k*-nearest-neighbours with a variant of the kernel-based Parzen-Rosenblatt Window approach: The suggested assignment of each individual in the dataset to a taxon is made based on the assignments of all other individuals within a predefined genetic distance $d$ from the focal individual. Neighbours with an “undetermined” assignment are ignored. The assignment of each neighbour contributes weight to a potential assignment of the focal individual. This weight depends on two factors. Firstly, a score expressing the influence of each of the focal individual’s neighbours, which decays exponentially, i.e. by a factor of $e^{{ - \lambda \cdot {\text{distance}}}}$, with some predefined constant $\lambda$ controlling the decay rate. Each potential assignment is given a total score by summing the weights of neighbours with that assignment. Secondly, the scores for each potential assignment are (optionally) normalised by the inverse of the frequency of neighbours bearing that assignment (i.e. individuals within $d$ of the focal), a means to alleviate bias towards oversampled taxa. For instance, for a focal individual with ten neighbours within $d$, 4 from taxon A and 6 from taxon B, the total score for A will be scaled by 10/4 and the total score from B scaled by 10/6. Individuals receive no contribution from themselves, meaning their assignment is entirely dependent on that of their neighbours. Where the highest-scoring potential assignment out of all the possible assignments for an individual proportionally greater than some predefined cutoff $r$, then this assignment is suggested for the focal individual, otherwise an “undetermined” assignment is suggested. A parameter $k$ for excluding all but the $k$-nearest-neighbours is also incorporated. Running the method with $\lambda = 0$, $d = \infty$, and the weighting of neighbours inversely to their taxon’s frequency disabled, is almost equivalent to the *k*-nearest-neighbours approach, with the exception that the focal individual is not counted among the k neighbours.

### Visualisation

The challenge of assessing how well different classifiers work is complicated by the lack of objective “correct” classifications. We therefore settle for a pragmatic compromise, and aim to establish ‘intuitive’ taxon assignments that simply ensure individuals assigned to a taxon are most-or equally-genetically similar to other members of that taxon, and that any obvious genetic demes correspond one-to-one to a unique taxon. Where possible, we aimed to preserve any defensible assignments given by the genebank passport data. Achieving such intuitive assignments demands a visualisation method optimised to display genetic similarity and dissimilarity among samples in two dimensions, with minimal loss of information. T-SNE, a dimensionality reduction and visualisation method that specifically aims to optimally preserve inter-item distances, was judged ideal for this purpose. Visual inspection of t-SNE plots (Supplementary Data) was used to select appropriate values of $\lambda$, $d$, $r$, and $k$ (see Fig. 1).

### Comparing methods

For the sake of comparison, the results of the UPGMA-clustering-based method of Tripodi et al. (2021) were also inspected, and an additional reclassification was done using the *k*-nearest-neighbours method recommended by van Bemmelen van der Plaat (2021). Statistics comparing the outcomes of the methods were generated using the deduplicated dataset, omitting the mixed cluster (Fig. 1, xii), with parameters for the kernel approach set as per Fig. 1, including the unassignment cutoff *r*.

### Data curation and treatment of duplicates

The IBS matrix used was generated by Tripodi et al. (2021). Briefly, short reads from 10,262 *Capsicum* samples were aligned to the *C. annuum* CM334 (Kim et al. 2017) reference genome, and a set of 26,566 biallelic SNPs obtained for analysis after filtering (variant quality > = 40, minimum read depth for homo- and heterozygotes = 2 and 4, respectively, minor allele count  > = 50, heterozygous call rate < 5%, missing data < 20%) and imputation via the FILLIN algorithm. Before creating assignments, the dataset was trimmed to include only columns and rows containing no invalid IBS scores (some IBS scores are marked N.A. in the dataset owing to, for instance, insufficient data), and which were not identified as duplicate accessions in the study of Tripodi et al. (2021). In the case of duplicate accessions, one was randomly selected to represent the whole set of duplicates. The final assignment of the chosen accession was then transferred to the rest of the set.

### Implementation and benchmarking

**Fig. 2:**
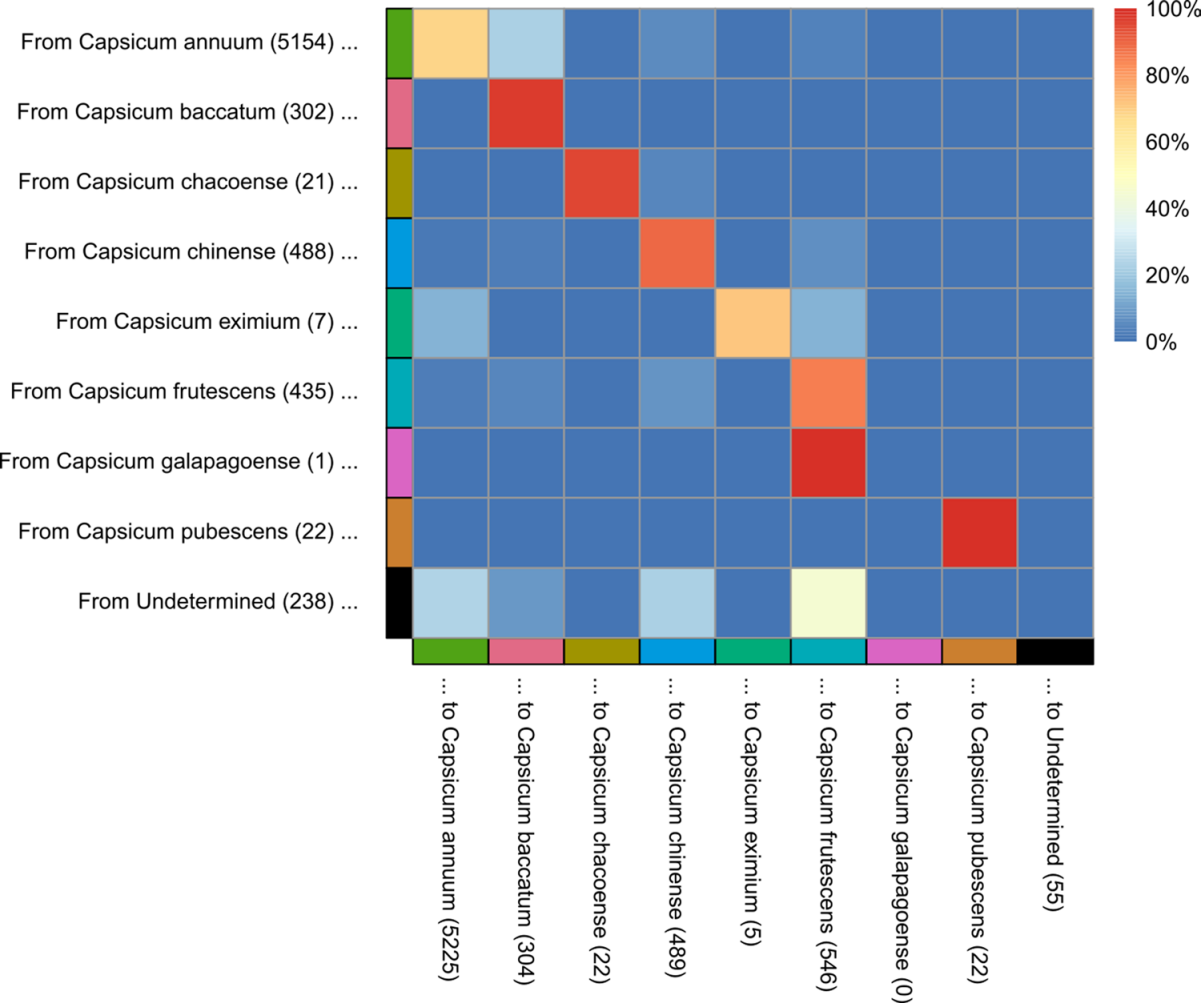
Reclassification rates for *Capsicum* divisions in the dataset using the kernel-based method presented in this study

The methods were implemented in R in a series of scripts prefixed with numbers and named according to their roles, which are (0) To set up the R environment and load packages and define relevant functions (1) to curate the raw IBS matrix, removing duplicates and rows/columns containing ‘NA’ values; (2) to create a tSNE plot coordinates from the IBS matrix, (3) to run the assignment function over a range of parameter values if required, plotting the results as per Figs. 1 and 2. The assignment function takes on average 7.23 s when run on a HP Envy laptop [16 Gb RAM, Intel(R) Core(TM) i7-1065G7 CPU at 1.30 GHz, 1498 MHz, 4 cores, 8 logical processors], dropping to 4.35 s when the *k*-nearest-neighbours functionality is disabled.

## Results

### Passport classifications

Visual comparison of the three methods of classification show marked differences in efficacy, with assignments using *k*-nearest-neighbours and the present method being far more intuitive and corresponding with demes evident in the t-SNE plots (Fig. 1). The passport classifications visualised by t-SNE shows that *C. annuum* refers to one major deme (Fig. 1i; henceforth roman numerals refer to markers in Fig. 1), although it appears to dominate a second smaller deme that contains a mixture of taxa (ii). *C. annuum*’s dominance in this secondary deme may, however, owe only to its being so well represented in the dataset compared with other taxa. Other important demes are dominated respectively by *C. chinense* (iii), *C. baccatum* (iv), and *C. frutescens* (v). Members of this *C. frutescens* deme are frequently classified as *C. annuum*, and also includes the dataset’s only instance of *C. galapagoense* (vi). Occasional unintuitive assignments can be seen in all the major demes but seems especially common in the *C. frutescens* deme, likely due to some ambiguity in morphology-based classification, perhaps explaining the large proportion of undetermined accessions in this deme. Other minor taxa assignments *C. chacoense* (vii), *C. baccatum* (viii), and *C. eximium* (ix) appear in this dataset to represent genetically separated groups well deserving of a distinct designation.

### UPGMA-clustering-based reclassifications

The Tripodi et al. (2021) UPGMA-clustering-based reclassifications make only minor improvements. A good proportion of taxon-undetermined accessions are given assignments but many remain, and while some reclassification appears to remove non-intuitive assignments, many of these remain too. However, some of the reassignments appear to actually introduce new errors, for instance within the *C. baccatum* deme, where small clusters that by chance capture closely-related misclassified taxa within a larger deme enable members of the dominant taxon to be reclassified as the minority taxon (x).

### *K*-nearest-neighbours

The *k*-nearest-neighbours approach makes significant improvements both to assigning undetermined accessions and to correcting unintuitive assignments, but is very much subject to a problem analogous to that just described for the UPGMA-clustering-based method above. This problem persists even when the neighbour count is increased, but before it eliminates all remaining undetermined or unintuitively-classified accessions, it begins to reclassify minor taxa with few representatives such as *C. eximium* (ix) as members of clearly-genetically-distinct taxa (in this case, *C. annuum* or *C. pubsescens*, depending on $k$; Supplementary Data). Overcoming this problem by introducing weightings based on distance and neighbour assignment frequency was a major motivation for the kernel-based method introduced in this publication.

### Kernel-based approach (present study)

The kernel-based method successfully overcomes these problems, unifying assignments in the one-taxon-dominated demes, including those with very few members, with, in the authors’ judgement, no unintuitive “out of place” assignments (Fig. 1). And, the number and nature of the reassignments made to achieve this level of congruency is substantial. Compared with the UPGMA-clustering method, the kernel approach suggested approximately three times as many assignment alterations (2.4% vs. 7.5% of all accessions). Of all the cases in which the kernel method suggested alterations, only 30.1% of the final assignments matched those suggested by the clustering method. And where the clustering method decreased the number of undetermined assignments by 31.8%, the kernel method gave 98% of undetermined accessions an assignment. The best results were attained with $k = \infty$ (effectively disabling the *k*-nearest-neighbour functionality). The inverse weighting of neighbours by assignment frequency proved essential for achieving this level of accuracy without causing genetically-distinct taxa with low sample numbers to be collapsed into other taxa. Similarly, manipulation of the decay parameter is particularly important to strike a balance between under-sensitivity and overgeneralisation (Supplementary Data). The ability of the kernel-based method to give the user very fine control over a number of continuous parameters (c.f. discontinuous parameters like $k$ in a *k*-nearest-neighbours approach) is responsible for this essential flexibility, which we believe will make the method a valuable contribution to achieving the equivalent tasks across diverse datasets. This includes continuous control over the parameter $r$, describing how much a potential assignment’s score must dominate for the focal individual to be given an assignment. The equivalent to $r$ in *k*-nearest-neighbours is discontinuous since it must designate some integer number of neighbours required to possess a shared assignment. The lower four panels of Fig. 1 demonstrate how this allows the kernel-based method to better choose a value of $r$ that eliminates unintuitive classifications but induces minimal extra undetermined samples that may require manual work to reclassify. Both take the lowest $r$ possible to remove obviously unintuitive assignments, but the kernel-based method is able to induce fewer unassigned calls within large demes where the assignment seems obvious.

### Quantifying the improvement

If we grant the assumption, based on (e.g.) Fig. 1, that the Kernel-based approach produces the most ‘intuitive’ assignments, we can define statistics to quantify the improvement in terms that relate to the use of genebank material. We define W50 (or “wrong-50”) as the number of accessions that can be randomly chosen before there is more than 50% probability that at least one ‘unintuitive’ assignment is encountered (in this case, an assignment that disagrees with the Kernel method, excluding the mixed cluster, Fig. 1, 12). By this measure, we estimate a W50 of ~ 8 for the raw passport data, of 18 for the best-performing cluster-based method, and 135 for the best KNN method.

**Fig. 3:**
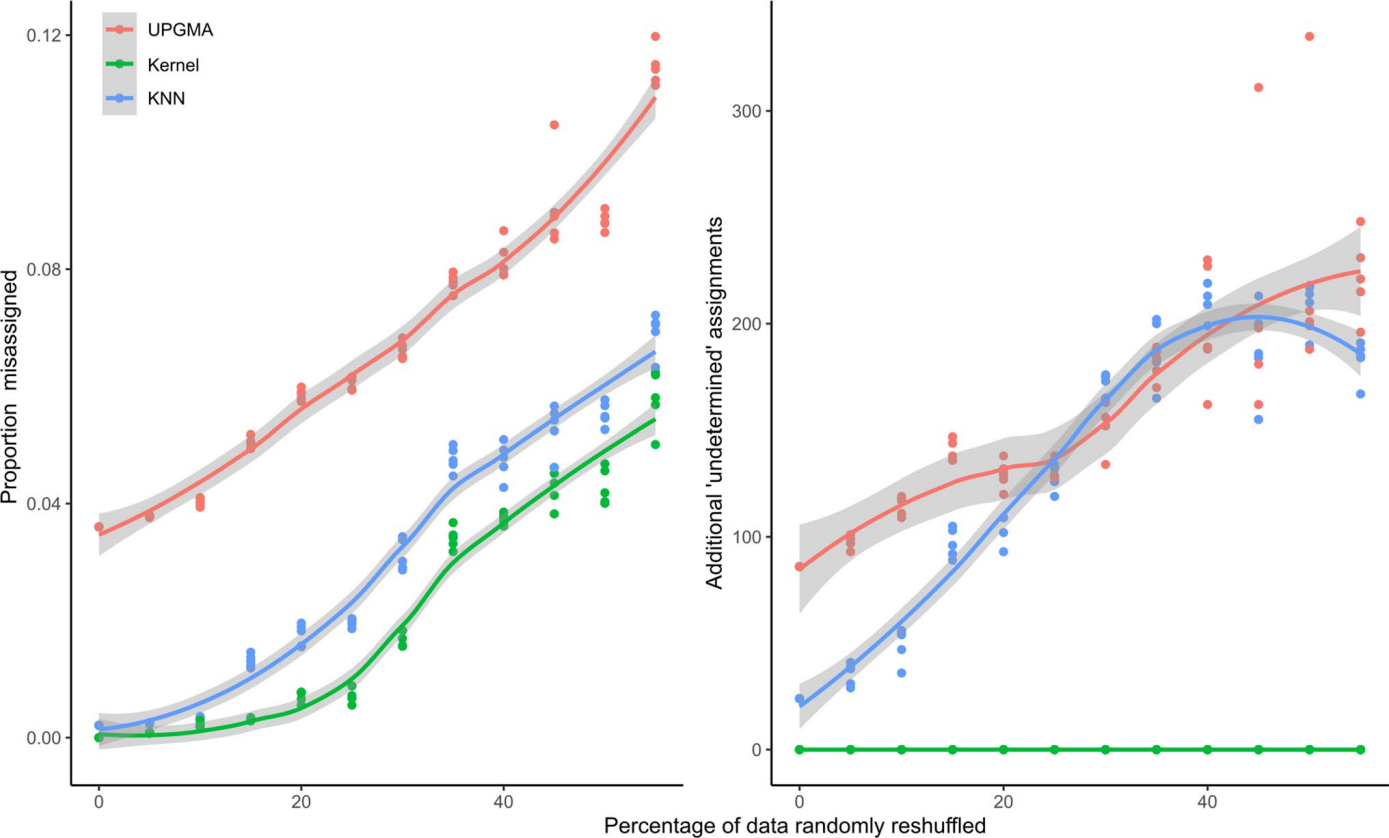
Sensitivity of results to random permutation of taxonomic assignments in the original dataset. Parameter setting for each method as per Fig. 2, but with ‘undetermined’ cutoffs (*r*) altered as described in results. Ten replicates were run per method/reshuffle rate. A LOESS smoother implemented in the R package ggplot2 (v.3.4.1; default parameter settings) is shown

We also attempted to quantify how each method loses effectiveness as the initial classifications become less and less homogeneous (Fig. 3). This is a concern in sample sets where (e.g.) phenotypic differences between taxa are subtle, or where many synonymous taxon names are defined. We therefore quantified, relative to the same reference set of classifications as above, the additional proportion of erroneous and ‘undetermined’ assignments that each method incurred, when an increasing proportion of the original assignments were randomly shuffled to induce noise. Since these two measured properties are a trade-off (i.e. creating many ‘undetermined’ assignments can reduce the proportion of incorrect assignments), we aimed to focus on misassignment, and allowed each method to create undetermined assignments only in the case of a score tie between candidate taxa (which only affects UPGMA and KNN), or a lack of data within the distance cutoff *r* (which only affects the kernel method). Despite being most ‘exposed’ to wrong assignments by incurring no additional ‘undetermined’ assignments, the kernel method outperformed all methods even when 50% of the data were randomly reshuffled.

### Discussion

#### Further comments on the kernel-based approach

The reassignments suggested by the approach support the existence of all pepper species featured in the study with the exception of perhaps *C. galapagoense*, though little can be concluded since it is here represented by a single accession. The limited number (*n* = 7) of *C. eximium* accessions in the study had a markedly high tendency to be putatively-misclassified *C. annuum* and *C. frutescens*, and *C. baccatum* included many putatively-misclassified *C. annuum*.

A key question raised by the observations of this study but no doubt broadly applicable to genebank genomic and taxonomic studies, is the appropriate treatment of genetic clusters that contain mixed species assignments, the best example of which here is marked ii in Fig. 1. A strength of the kernel-based approach is that, owing to frequency-based normalisation of potential taxon scores, the mixed cluster is not as susceptible to being subsumed into its most common member simply owing to that member being better represented in the dataset. This phenomenon affects the UPGMA-clustering-based method (Fig. 1), and *k*-nearest-neighbours for many larger values of $k$ that would otherwise make for sensible choices (Supplementary Data). By alleviating this bias, the mixed cluster retains its mixed character and moreover, when an assignment cutoff is imposed, it is quickly flagged to the investigator that the cluster is taxonomically ambiguous, owing to the prevalence of unassigned samples within it. We defer to the *Capsicum* taxonomy community to discuss the merits of assigning members of this cluster a specific, unified designation.

#### Implications of the results for *Capsicum* taxonomy

Equally important as parametric flexibility and ambiguity handling is the visualisation method used to assess how well chosen parameters achieve sensible results, especially in cases where the actual genetic structure of the population and distribution of taxon assignments over the genetic diversity space is initially unknown. Naturally every possible choice of visualisation tool has trade-offs and one must be cautious of, for example, the distortions that can be induced by dimensionality reduction. Such distortion may explain why some accessions are persistently given assignments that surprisingly do not match those of the accessions clustered around them in the plot (Supplementary Data). Tripodi et al. (2021) present some evidence that suggests highly-heterozygous early-generation hybrid individuals may be a key cause of this taxonomic ambiguity, and indeed the challenge of heterozygosis in obligate outcrossing species means that future studies employing methods such as that presented, which require a distance matrix, will need to consider more sophisticated distance measures than IBS before these methods can be applied.

#### Outlook

Solving the problem addressed in this study becomes increasingly important as genebank genomic studies increase in number, alongside other studies containing large numbers of taxa where establishing intuitive taxonomic assignments is an advantage. However, a one-size-fits-all approach is unlikely ever to arise, owing to inconsistency in (a) the genetic structure of the groups being analysed, (b) the conventional bases of taxonomic delineation within the taxon, and (c) the taxonomic level on which differentiation is needed. Here we analysed taxa that are classified as different species, which might have a stronger evolutionary basis than, e.g., intraspecific entities like subspecies, varieties and cultivars. Expanding the small pool of current solutions is therefore advantageous, as is expanding the pool of methods used to assess their effectiveness to include more intuitive, visual methods. The solution we present here contributes to solving this problem by (a) introducing a novel approach to both the classification and visual assessment steps that works well, and (b) demonstrating their effectiveness on a state-of-the-art dataset that will immediately benefit a large active research community working on an agronomically and culturally important crop. Since the method is fast to run, groups can easily experiment with parameters to suit their datasets. A possible extension to the method involves iteratively reclassifying samples to check whether a sensible stable assignment set is reached, and while this did not in our experimentation confer any significant advantage using the current dataset, we welcome future developments that explore this and other avenues to bring about further improvements.

### Supplementary Information

Below is the link to the electronic supplementary material.

Results of testing multiple parameter combinations on the classification of samples in the G2P-Sol Capsicum dataset using the kernel-based method presented in this study (PDF 101797 kb)

As per S1, but showing results of k-nearest-neighbour classification with different parameter values for k (PDF 9028 kb)

Table of taxonomic assignments given to the G2P-Sol pepper accessions in this study (CSV 1464 kb)

## Data Availability

The datasets analysed for this study are publicly available and hosted at zenodo.com under https://doi.org/10.5281/zenodo.7016070. Scripts implementing the method in R for these data are available at github.com/mtrw/genebank_taxonomic_assignment.
