# Supplementary material for: Accurate, automated taxonomic assignment of genebank accessions: a new method demonstrated using high-throughput marker data from 10,000 Capsicum spp. accessions
Source: Theor Appl Genet. 2023 Sep 11;136(10):208. doi: 10.1007/s00122-023-04441-8 (PMC10495273; doi:10.1007/s00122-023-04441-8)

Rabanus-Wallace 2022

Supplementary Materials S1

Description

The results of taxonomic reclassification of the G2P pepper dataset using the kernel-based method described in the main text, under a particular parameter combination over *d*, *lambda*, and *r*, given in the title of the leftmost panel. ***First of each {d,lambda} combination***: The distribution of IBS distances between members of the Capsicum dataset, shown using a kernel density approximation (R v4.0.5, function `density()`) with bandwidth as indicated below each panel. The height of the blue line (arbitrarily scaled) indicates the relative weighting given to distances under that particular parameter selection. ***Subsequent plots in each {d,lambda} combination***: Taxonomic assignments of individuals in the G2P pepper dataset, visualised using the t-SNE procedure with perplexity 30, as per main text figure 1. Colours as per figure 1, reproduced in the legend below.

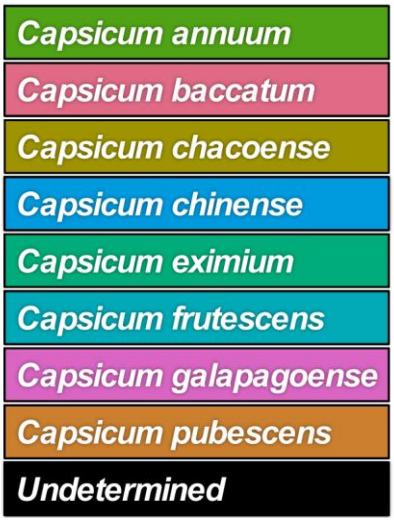

Distribution of IBS values with relative scaling factor for weighting (blue) lambda=0 d=0.01

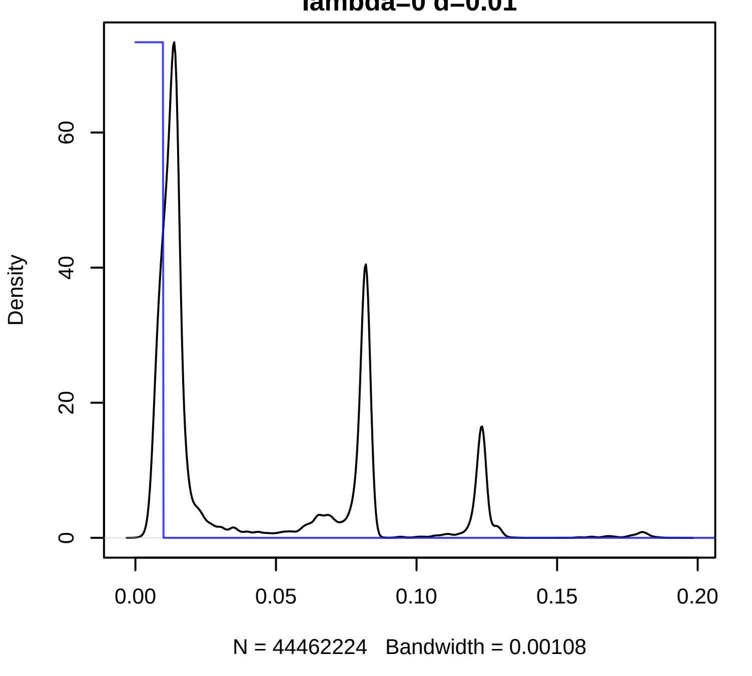

lambda=0 d=0.01 r=0 knn=Inf

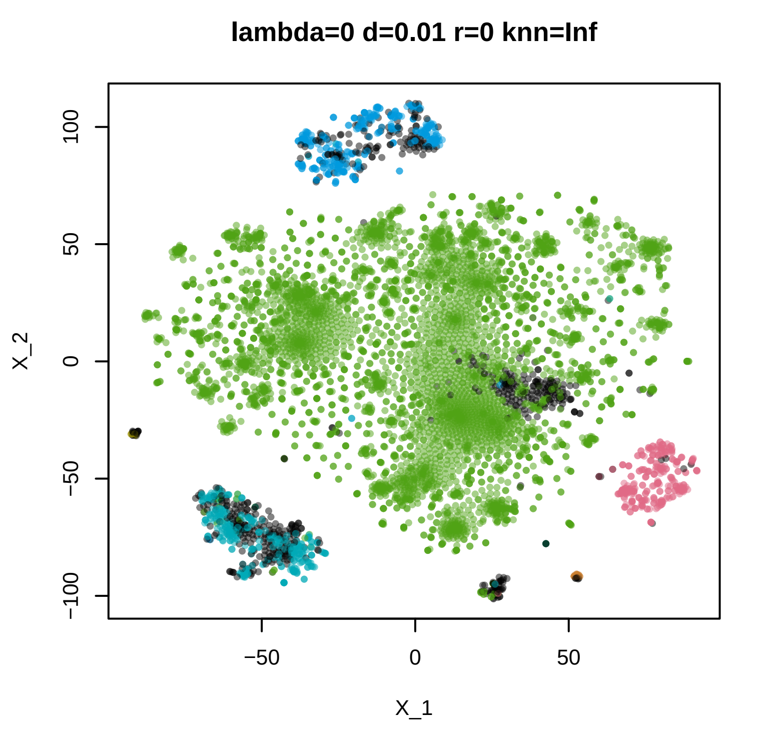

lambda=0 d=0.01 r=0.333333333333333 knn=Inf

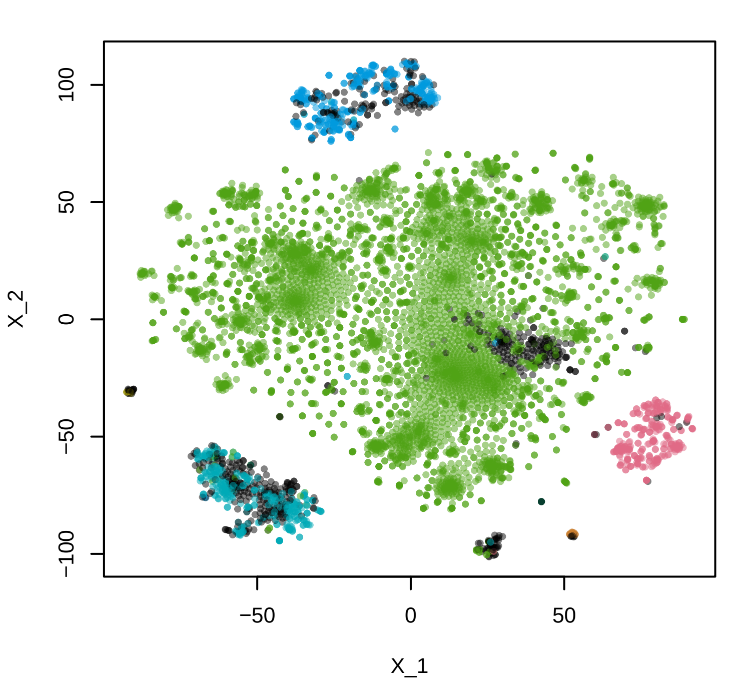

lambda=0 d=0.01 r=0.666666666666667 knn=Inf

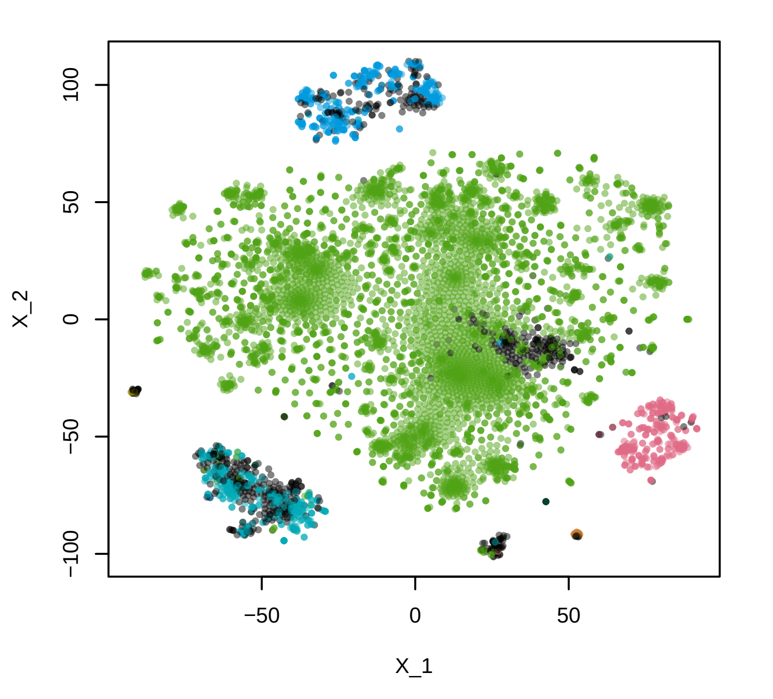

Distribution of IBS values with relative scaling factor for weighting (blue) lambda=0 d=0.0125

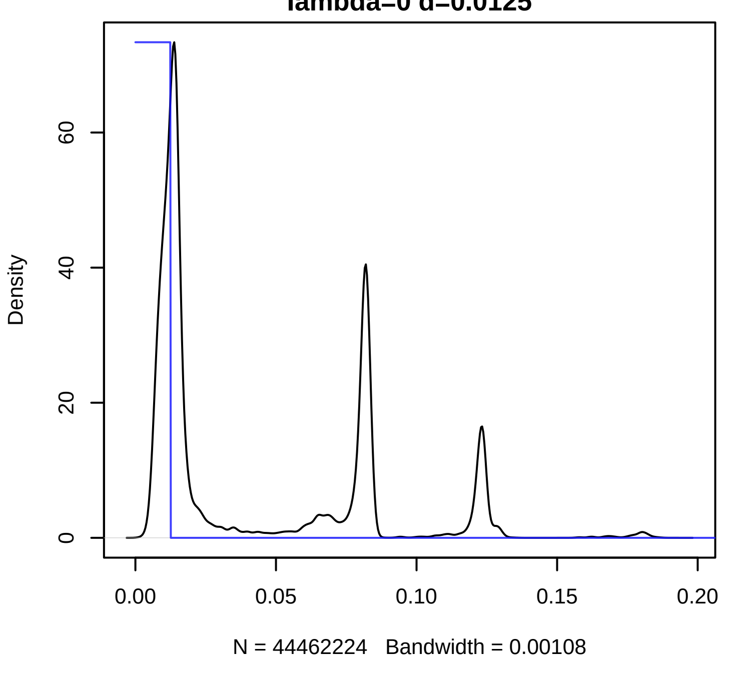

lambda=0 d=0.0125 r=0 knn=Inf

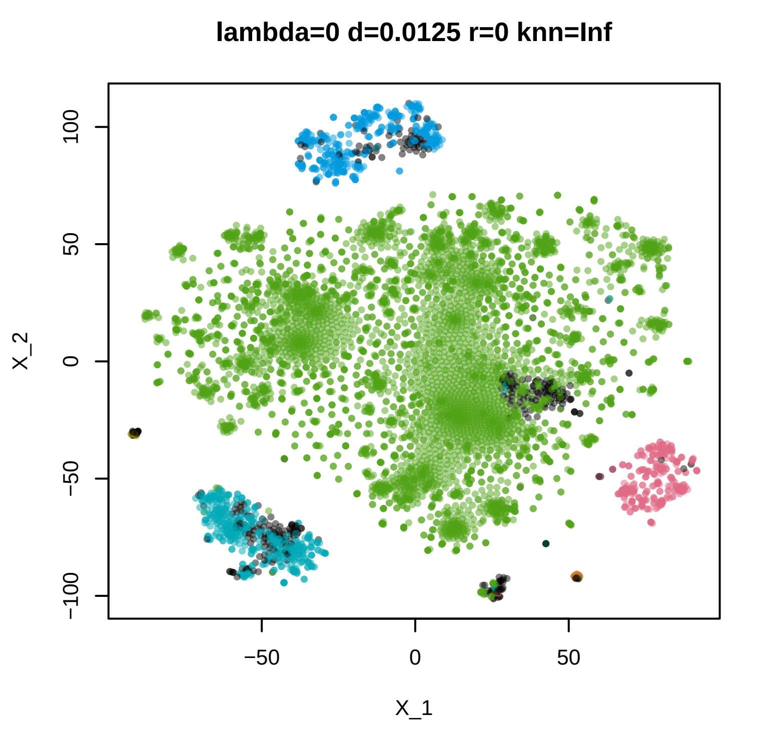

lambda=0 d=0.0125 r=0.333333333333333 knn=Inf

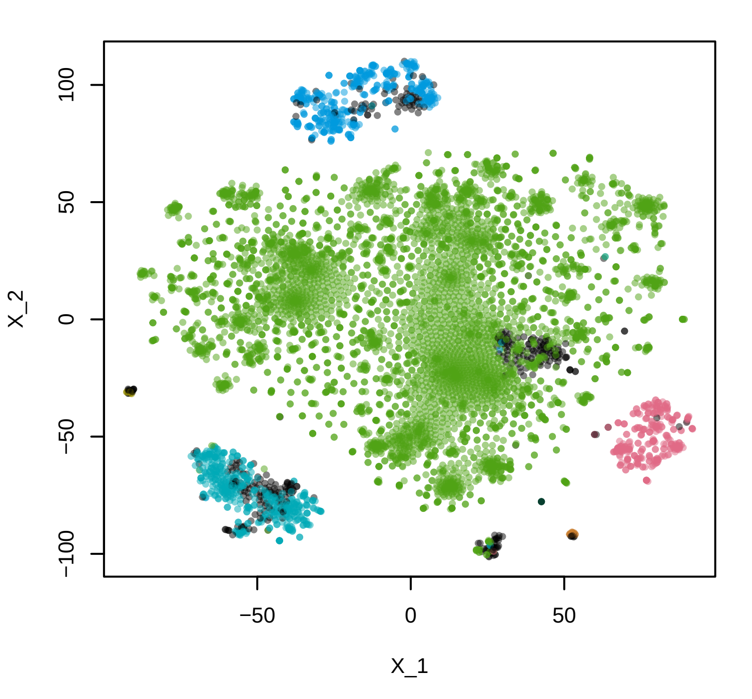

lambda=0 d=0.0125 r=0.666666666666667 knn=Inf

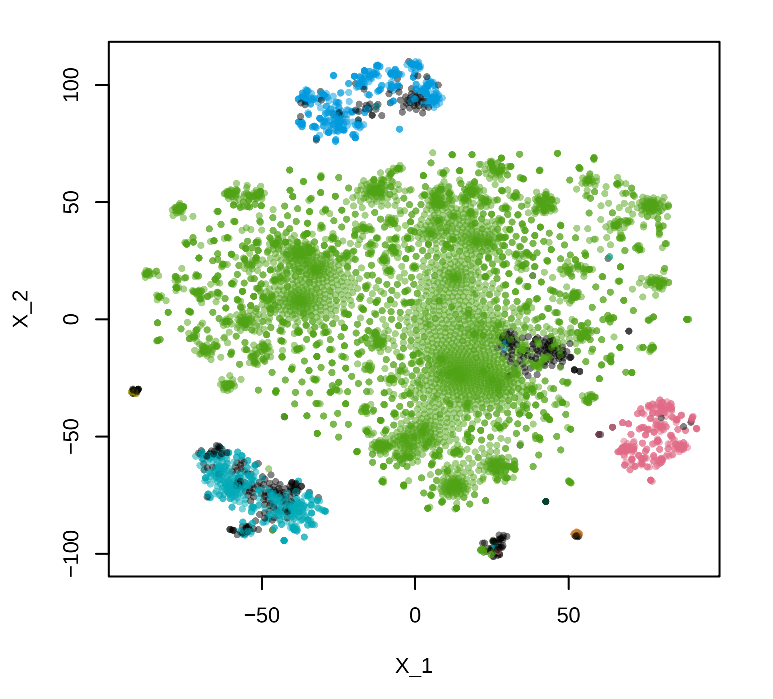

Distribution of IBS values with relative scaling factor for weighting (blue) lambda=0 d=0.015

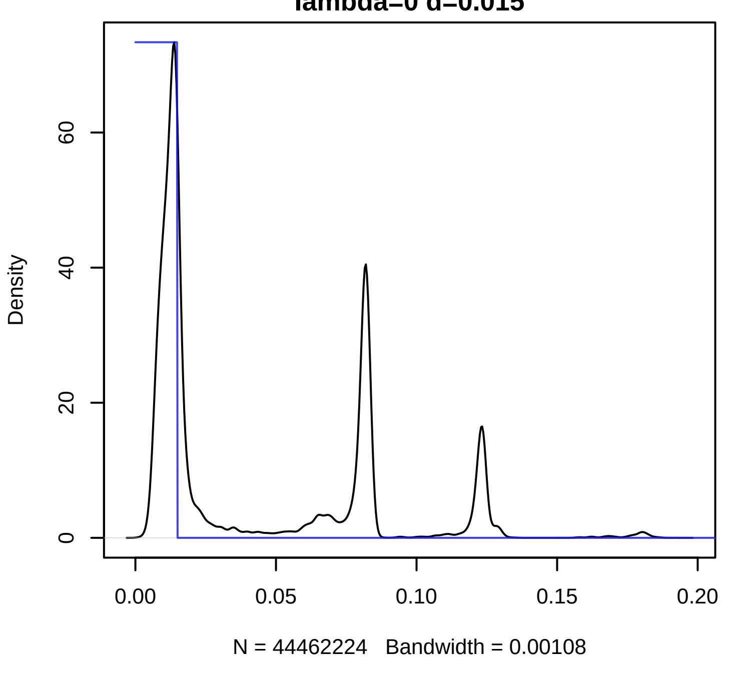

lambda=0 d=0.015 r=0 knn=Inf

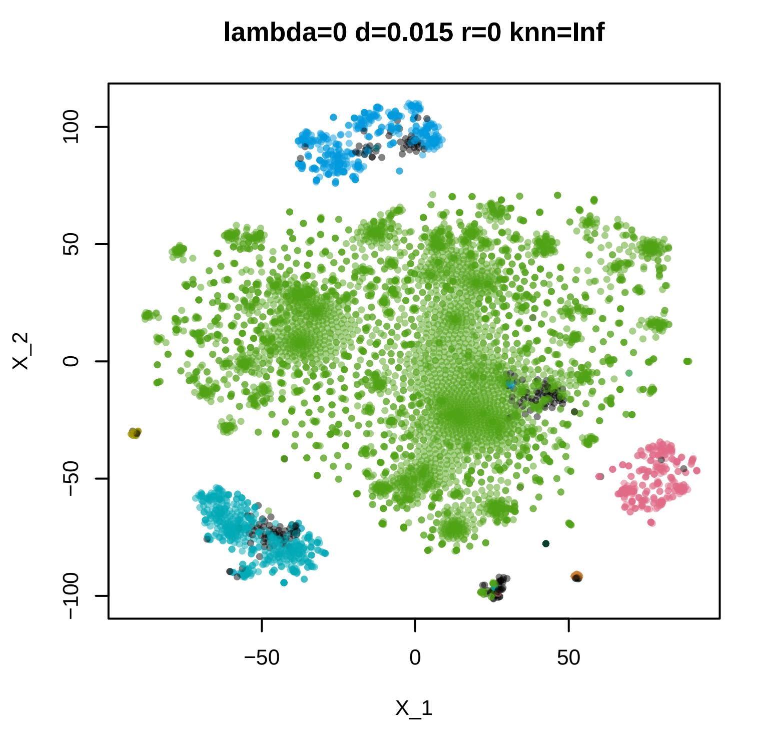

lambda=0 d=0.015 r=0.333333333333333 knn=Inf

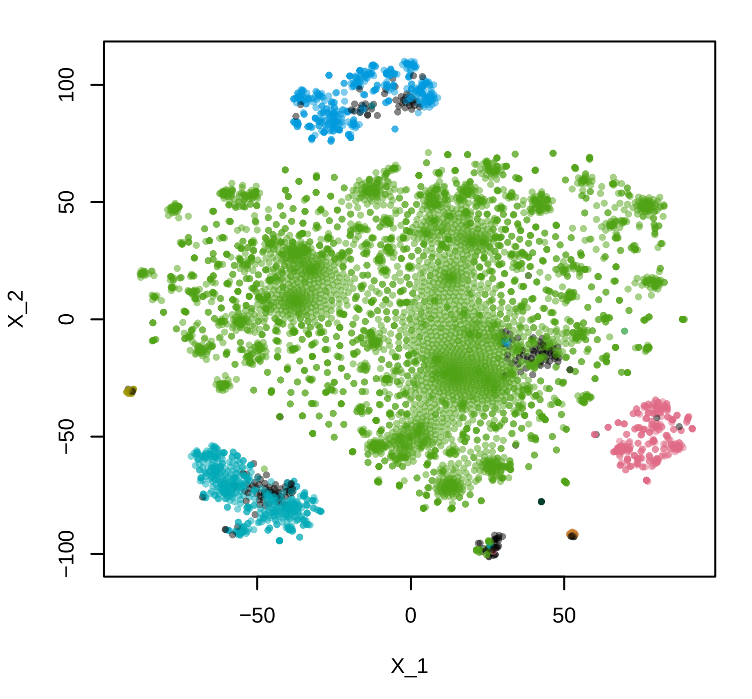

lambda=0 d=0.015 r=0.666666666666667 knn=Inf

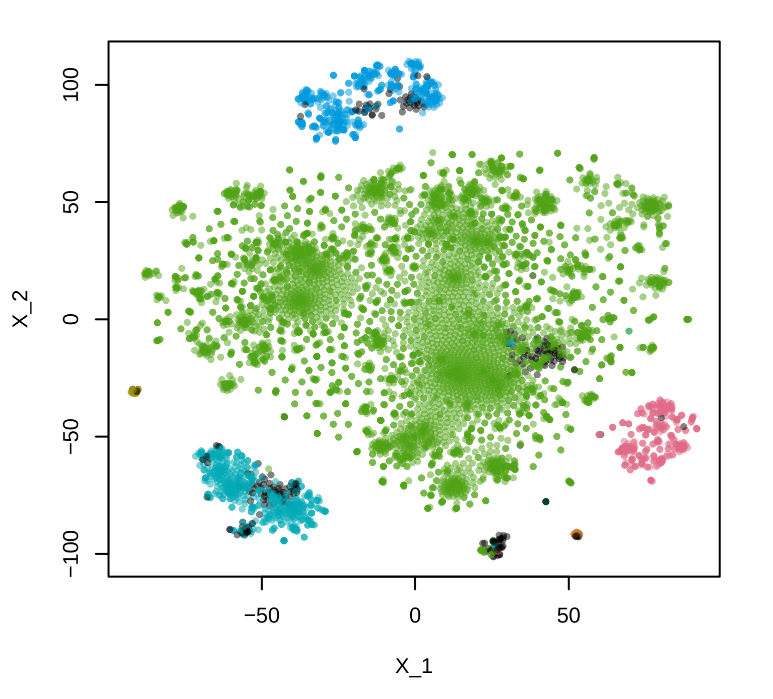

Distribution of IBS values with relative scaling factor for weighting (blue) lambda=0 d=0.0175

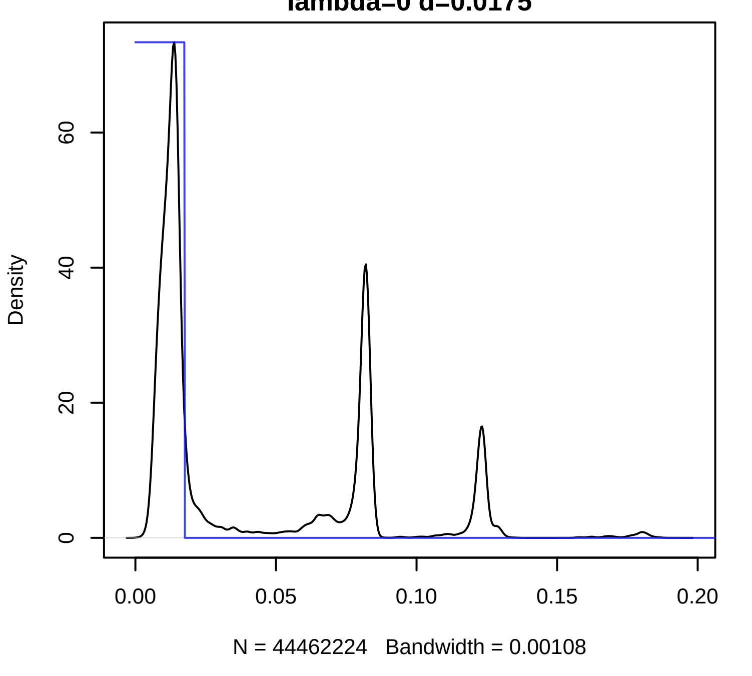

lambda=0 d=0.0175 r=0 knn=Inf

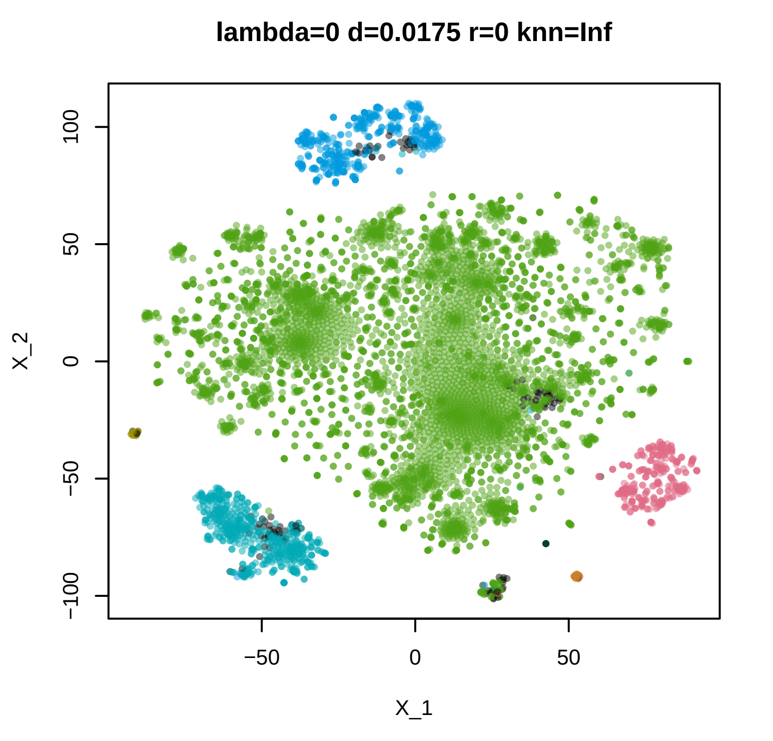

lambda=0 d=0.0175 r=0.333333333333333 knn=Inf

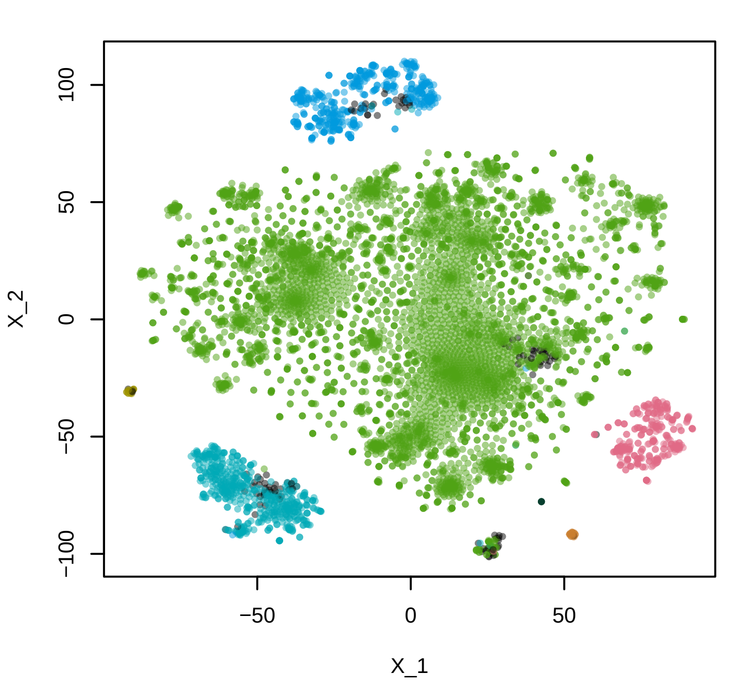

lambda=0 d=0.0175 r=0.666666666666667 knn=Inf

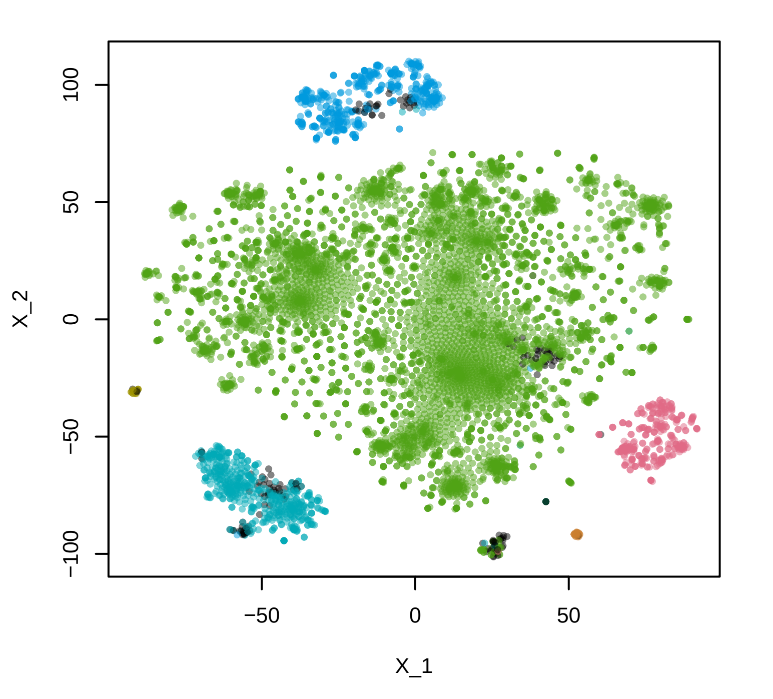

Distribution of IBS values with relative scaling factor for weighting (blue) lambda=0 d=0.02

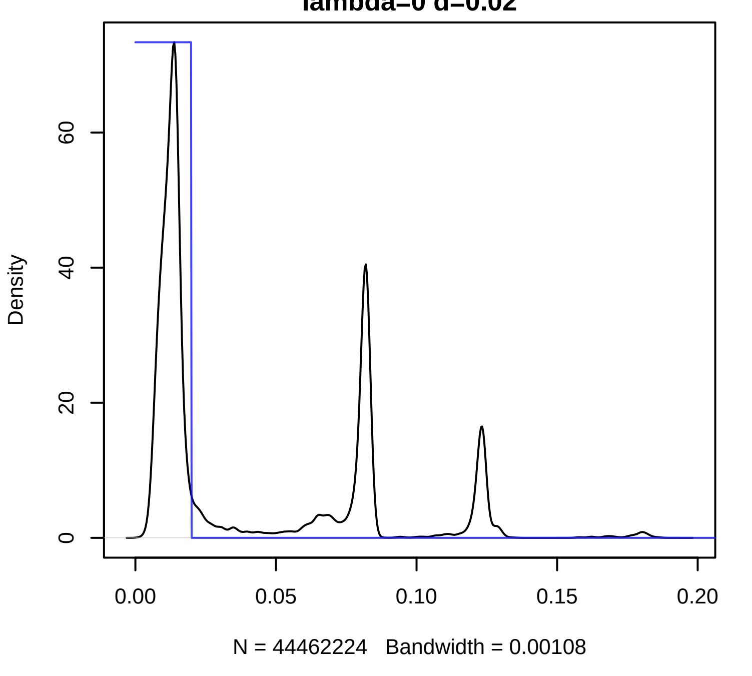

lambda=0 d=0.02 r=0 knn=Inf

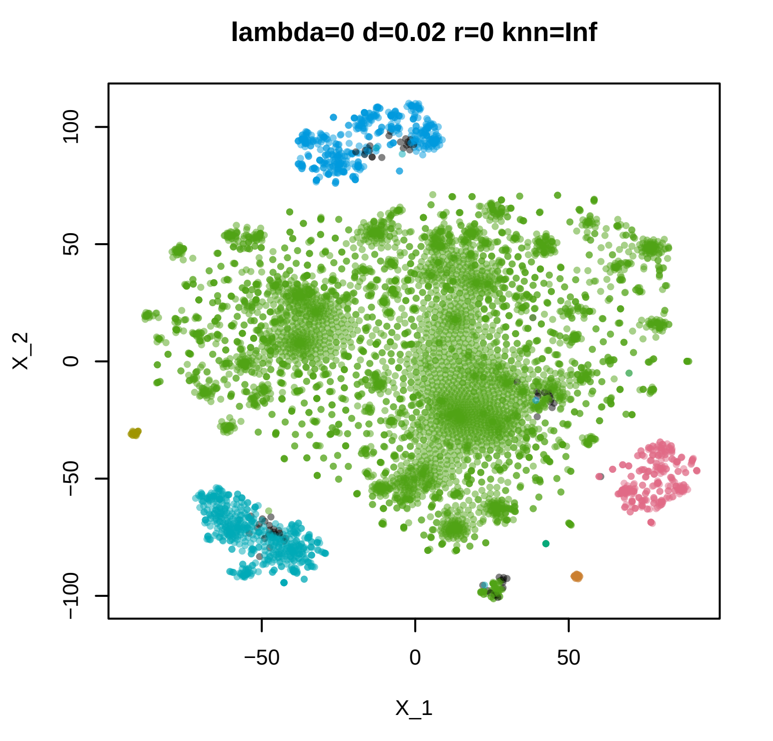

lambda=0 d=0.02 r=0.333333333333333 knn=Inf

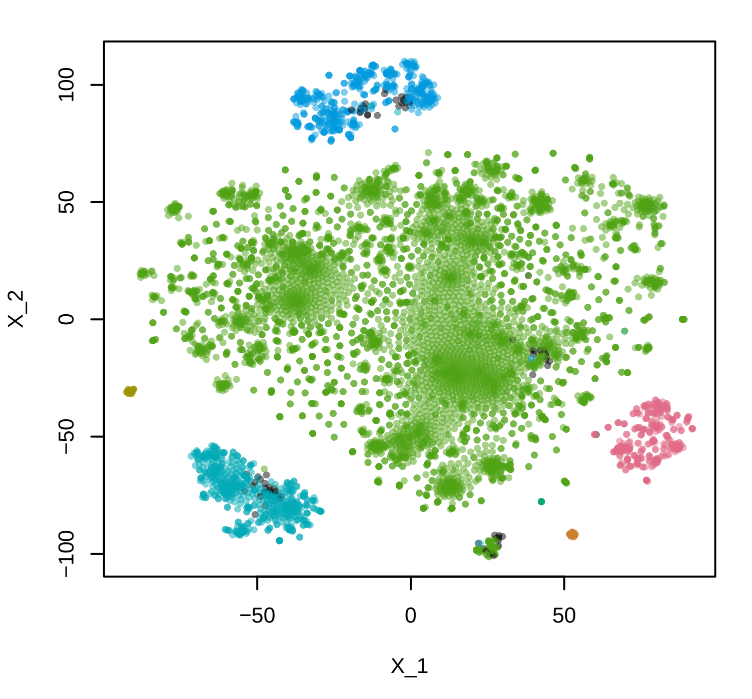

lambda=0 d=0.02 r=0.666666666666667 knn=Inf

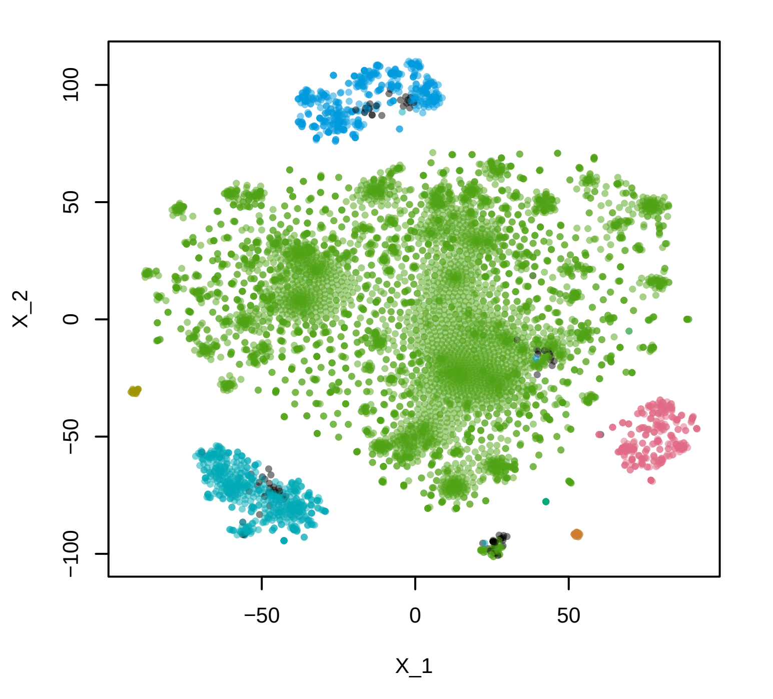

Distribution of IBS values with relative scaling factor for weighting (blue) lambda=0 d=0.0225

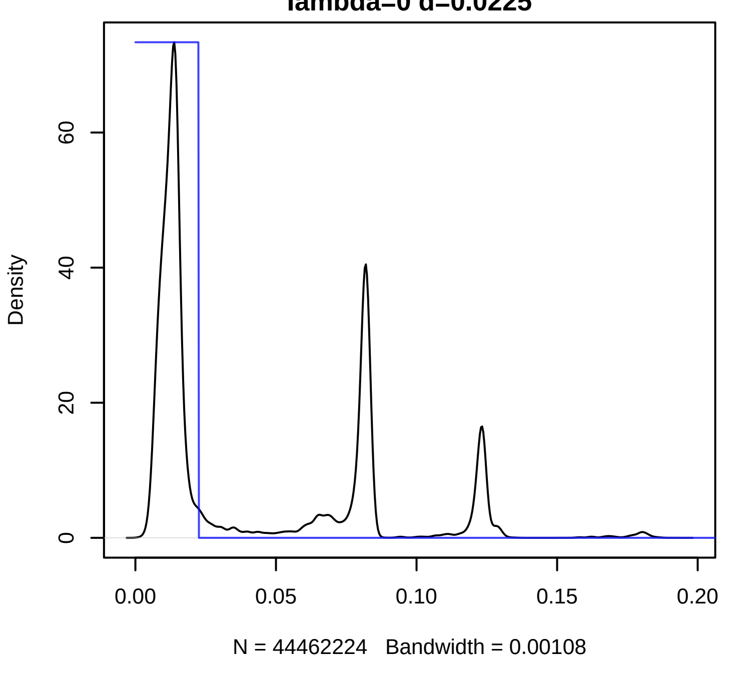

lambda=0 d=0.0225 r=0 knn=Inf

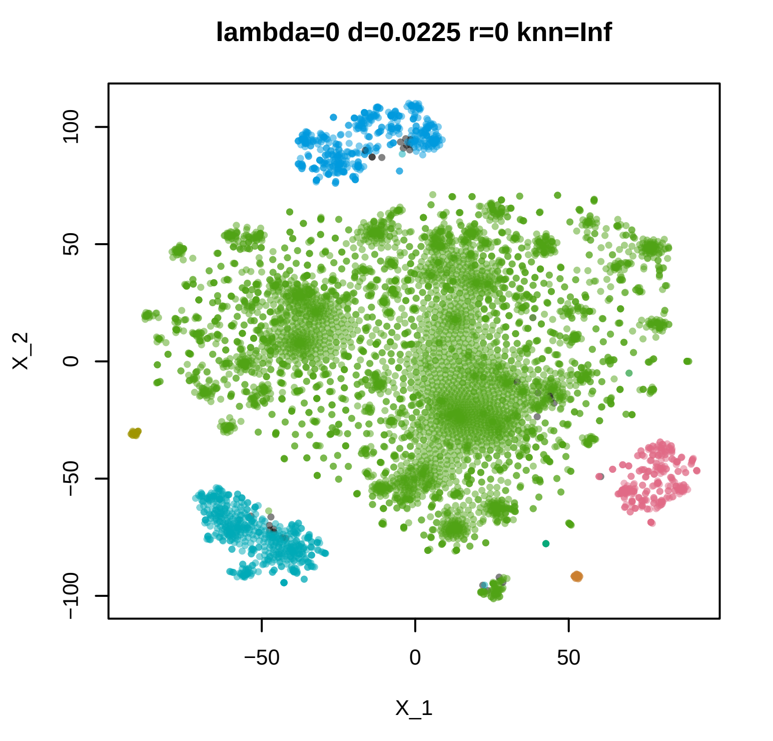

lambda=0 d=0.0225 r=0.333333333333333 knn=Inf

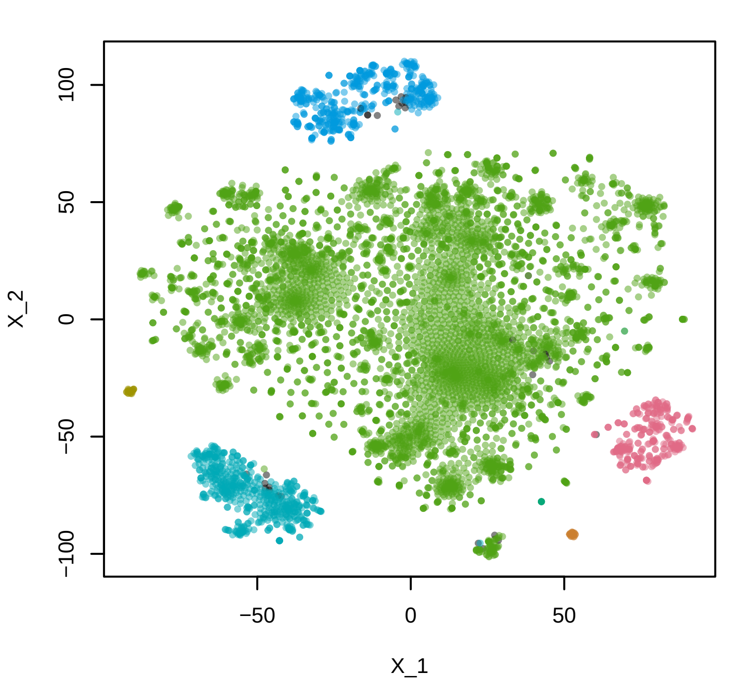

lambda=0 d=0.0225 r=0.666666666666667 knn=Inf

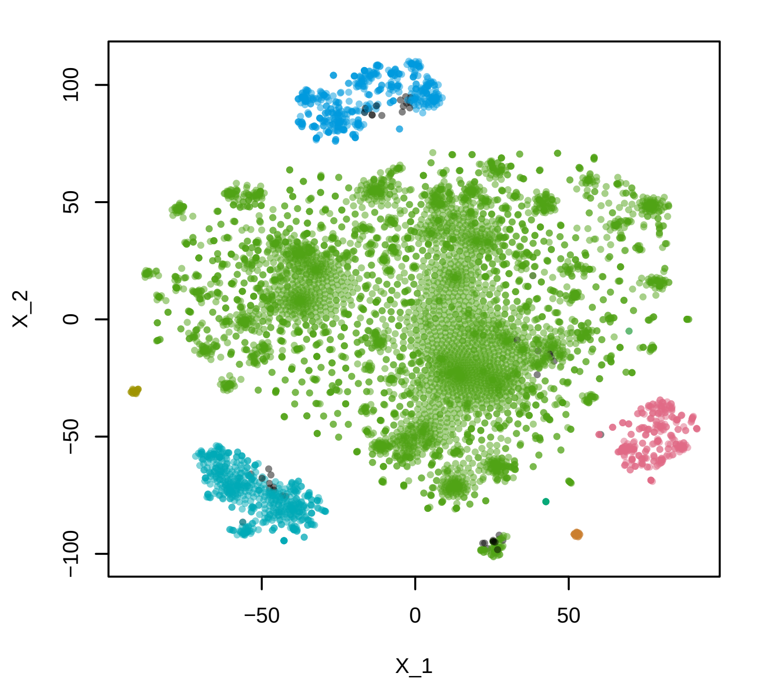

Distribution of IBS values with relative scaling factor for weighting (blue) lambda=0 d=0.025

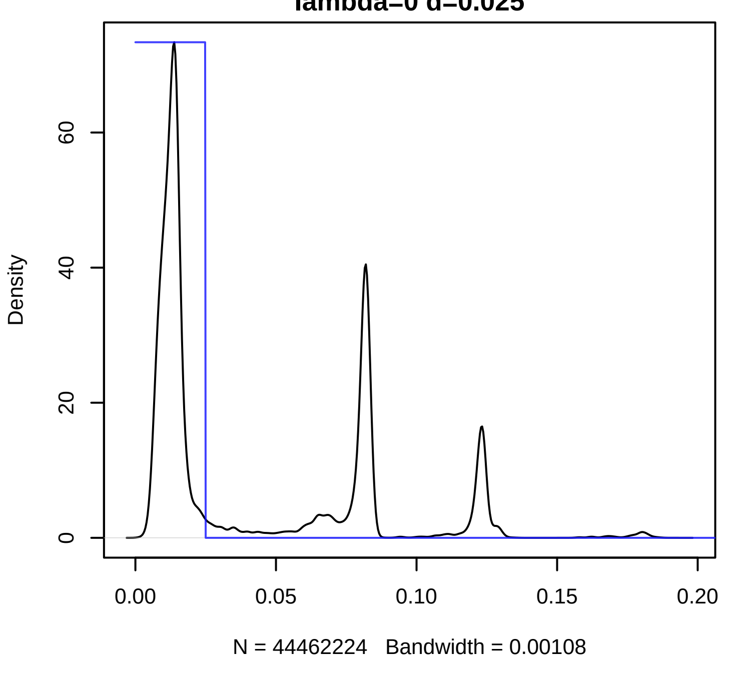

lambda=0 d=0.025 r=0 knn=Inf

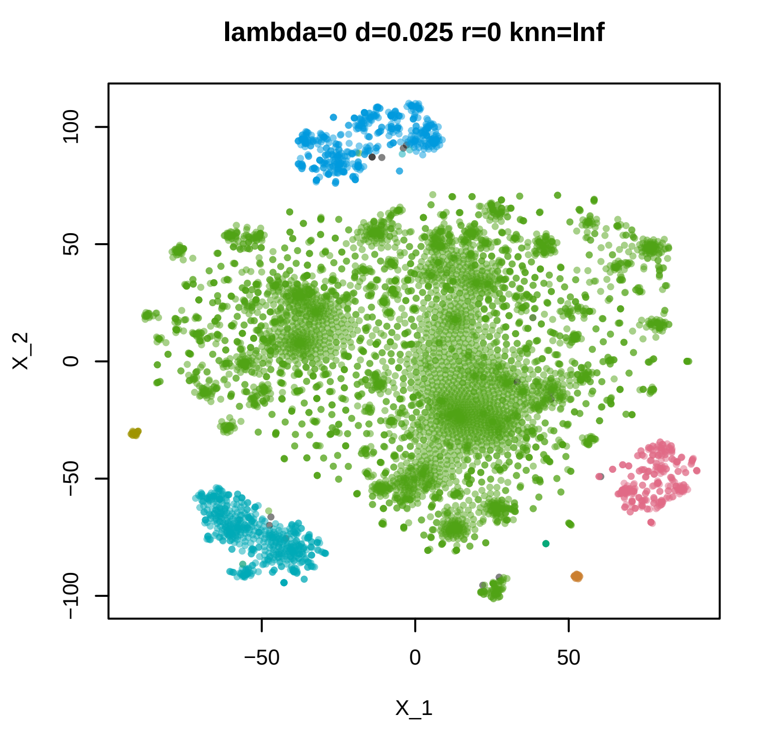

lambda=0 d=0.025 r=0.333333333333333 knn=Inf

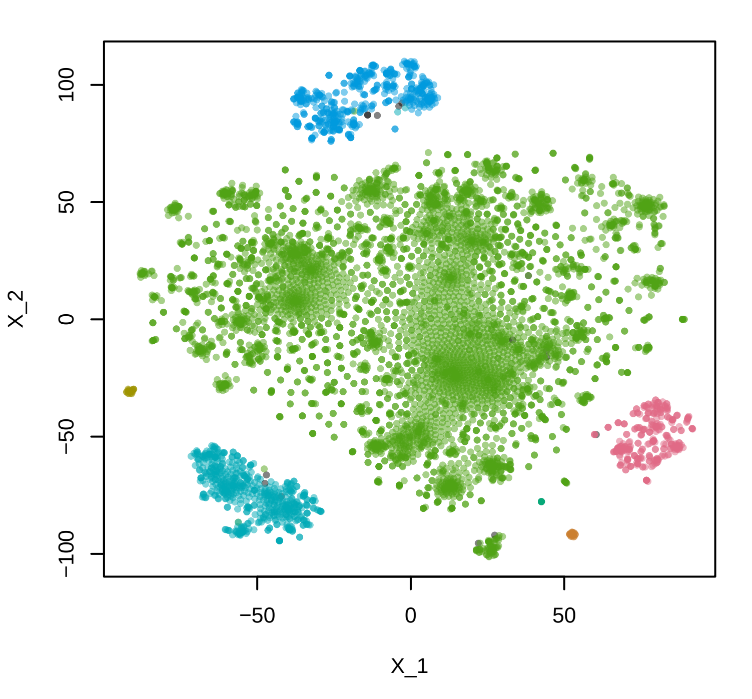

lambda=0 d=0.025 r=0.666666666666667 knn=Inf

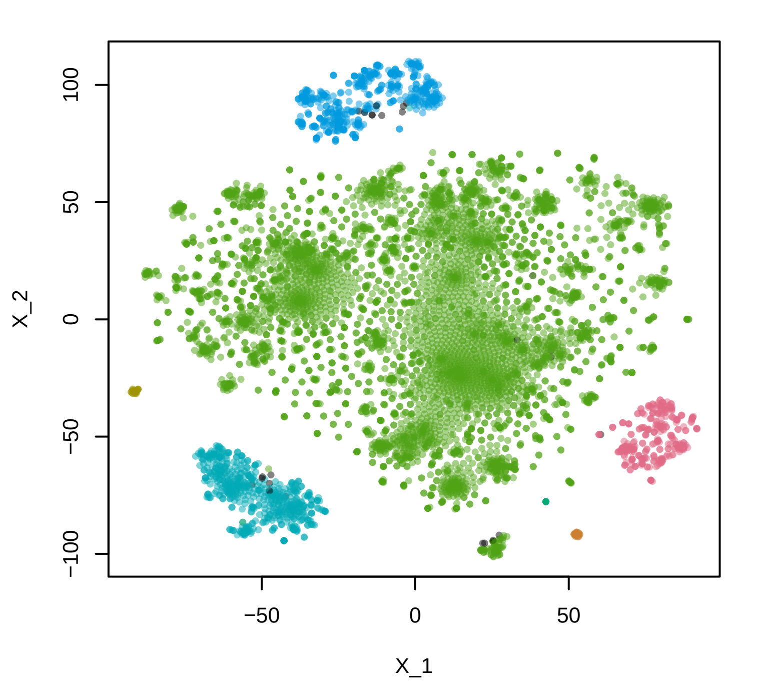

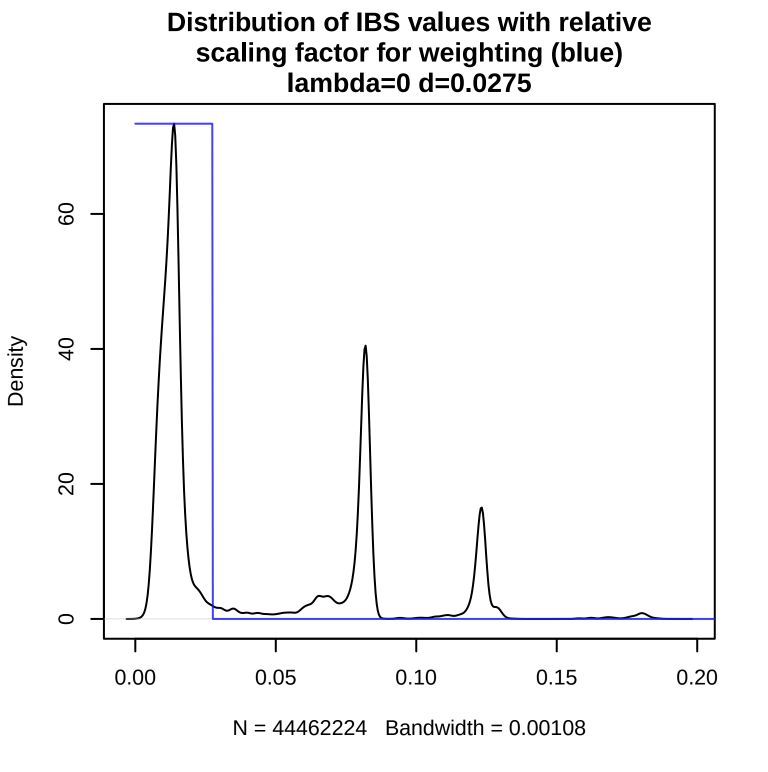
Distribution of IBS values with relative
scaling factor for weighting (blue)
lambda=0 d=0.0275
Density
N = 44462224 Bandwidth = 0.00108
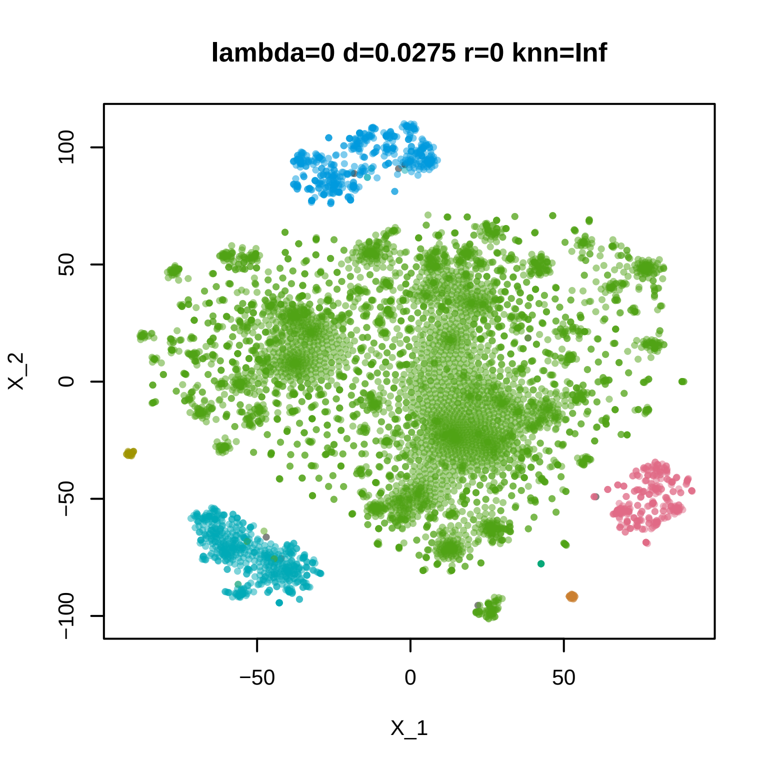
lambda=0 d=0.0275 r=0 knn=Inf
X_1
X_2
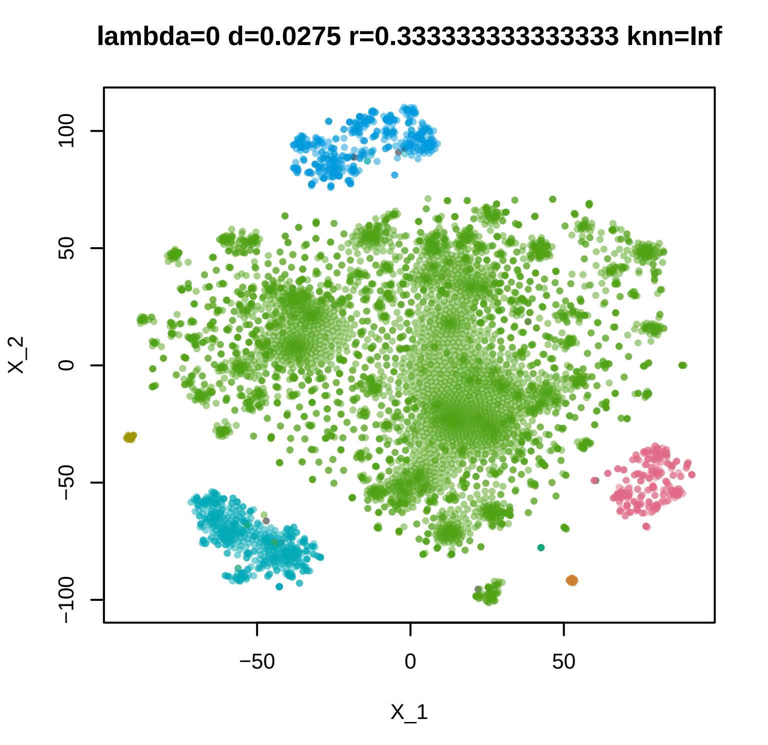
lambda=0 d=0.0275 r=0.333333333333333 knn=Inf
X_1
X_2
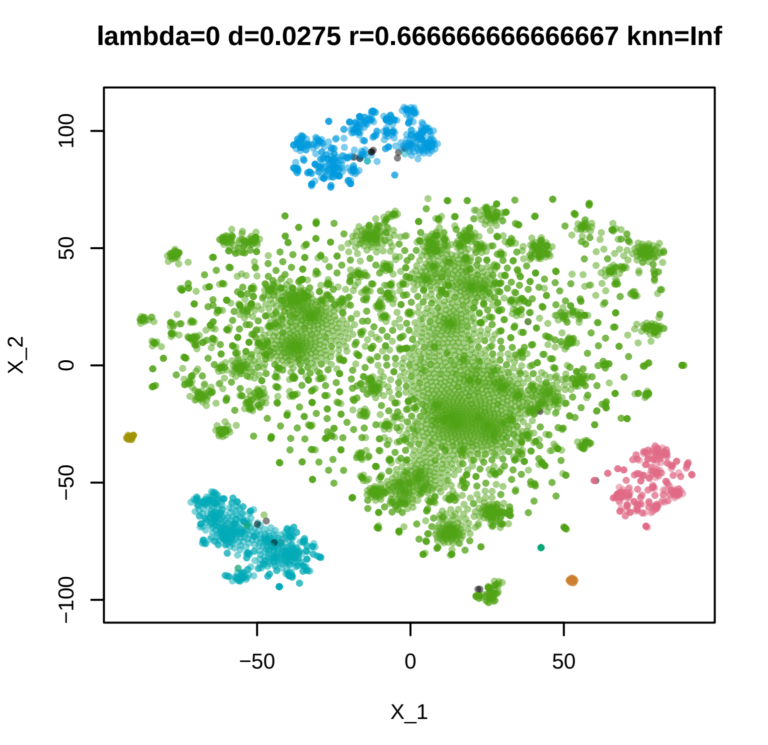
lambda=0 d=0.0275 r=0.666666666666667 knn=Inf
X_1
X_2
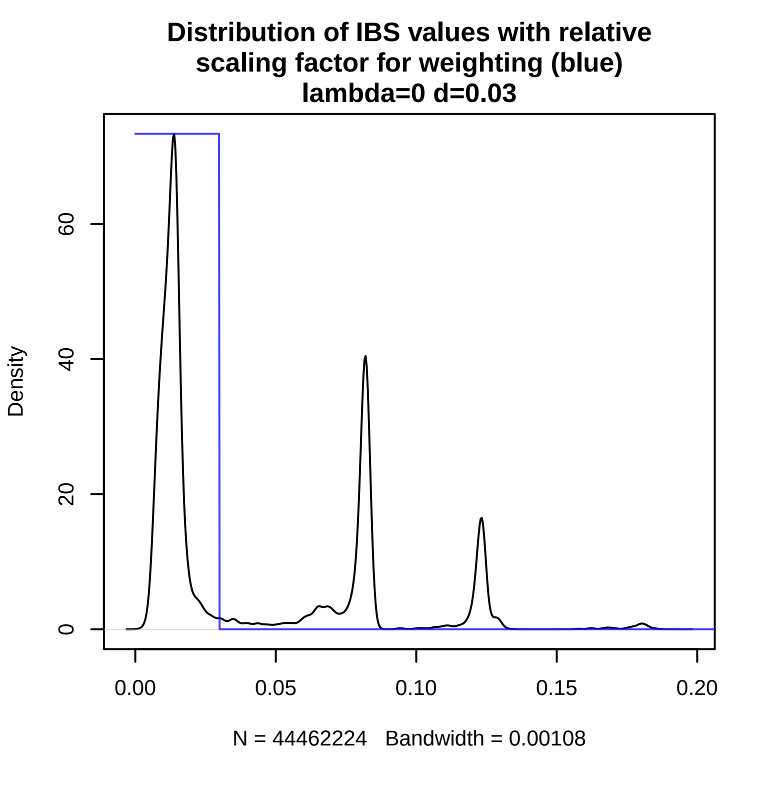
Distribution of IBS values with relative
scaling factor for weighting (blue)
lambda=0 d=0.03
Density
N = 44462224 Bandwidth = 0.00108
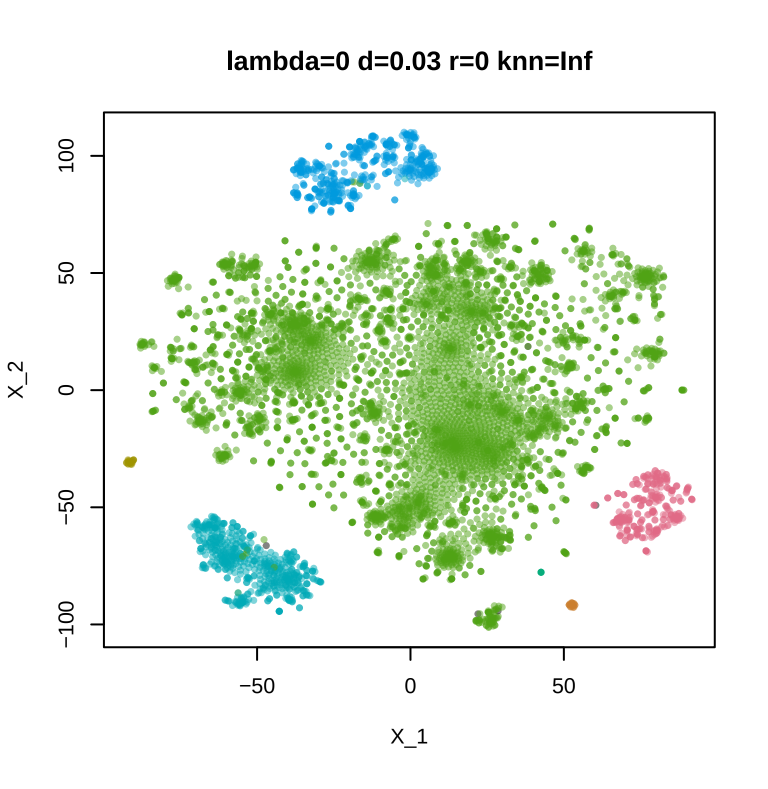
lambda=0 d=0.03 r=0 knn=Inf
X_1
X_2
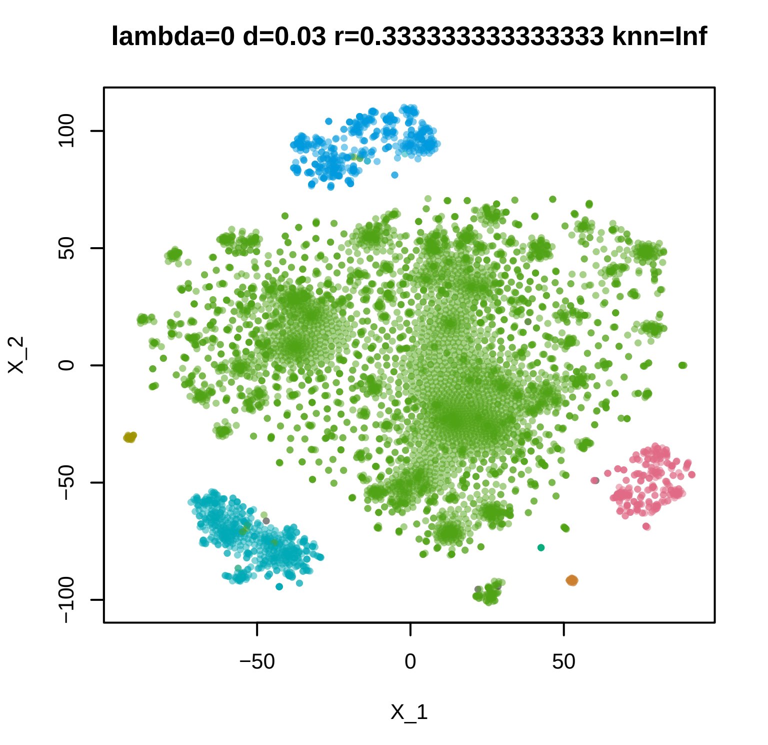
lambda=0 d=0.03 r=0.333333333333333 knn=Inf
X_1
X_2
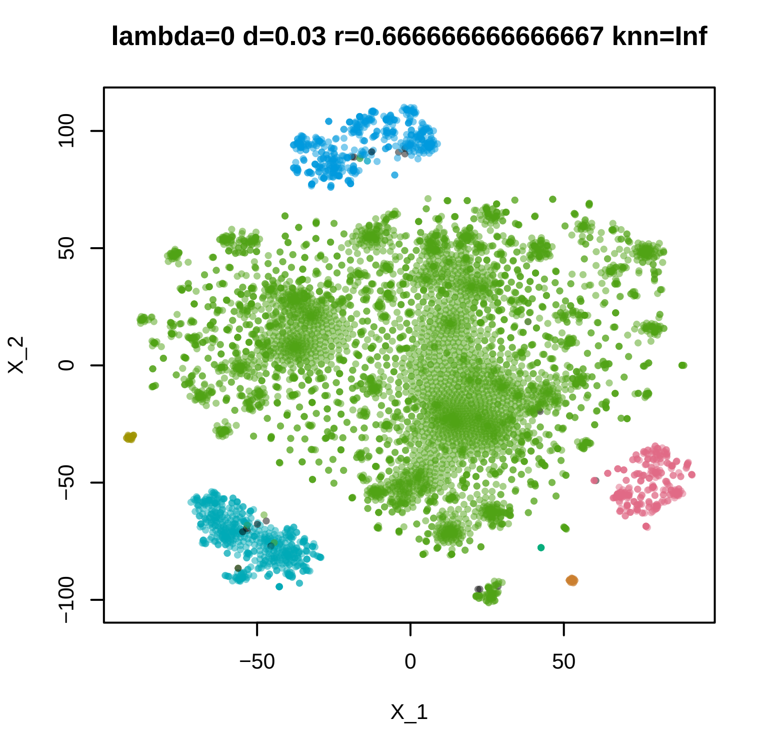
lambda=0 d=0.03 r=0.666666666666667 knn=Inf
X_1
X_2
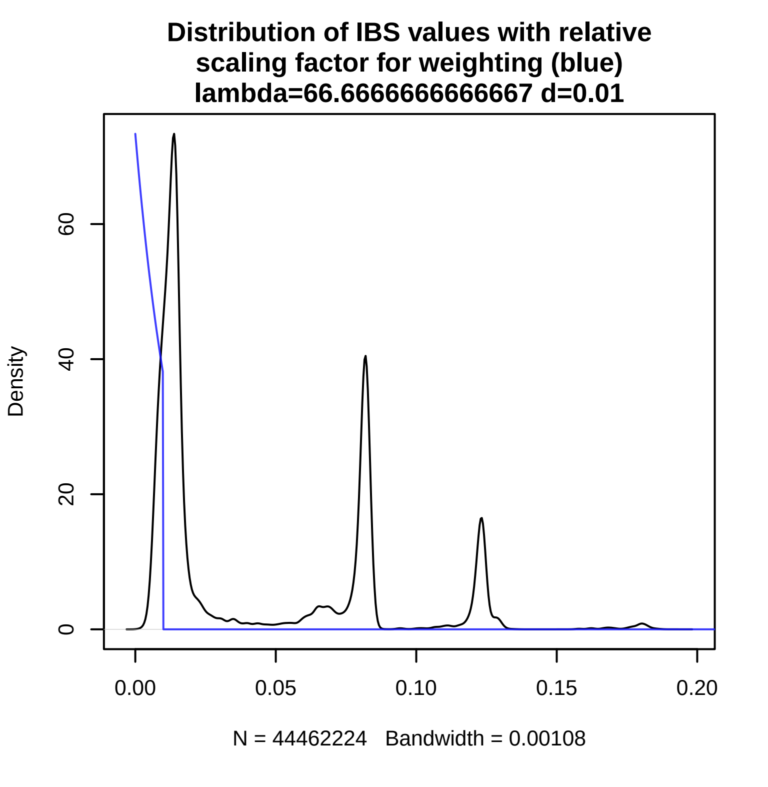
Distribution of IBS values with relative
scaling factor for weighting (blue)
lambda=66.6666666666667 d=0.01
Density
N = 44462224 Bandwidth = 0.00108
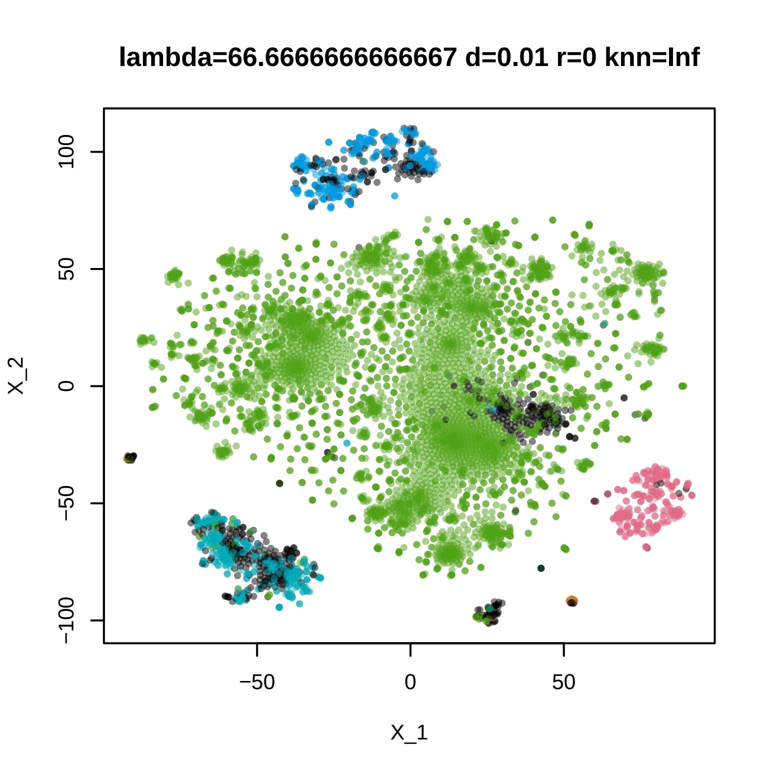
lambda=66.6666666666667 d=0.01 r=0 knn=Inf
X_1
X_2
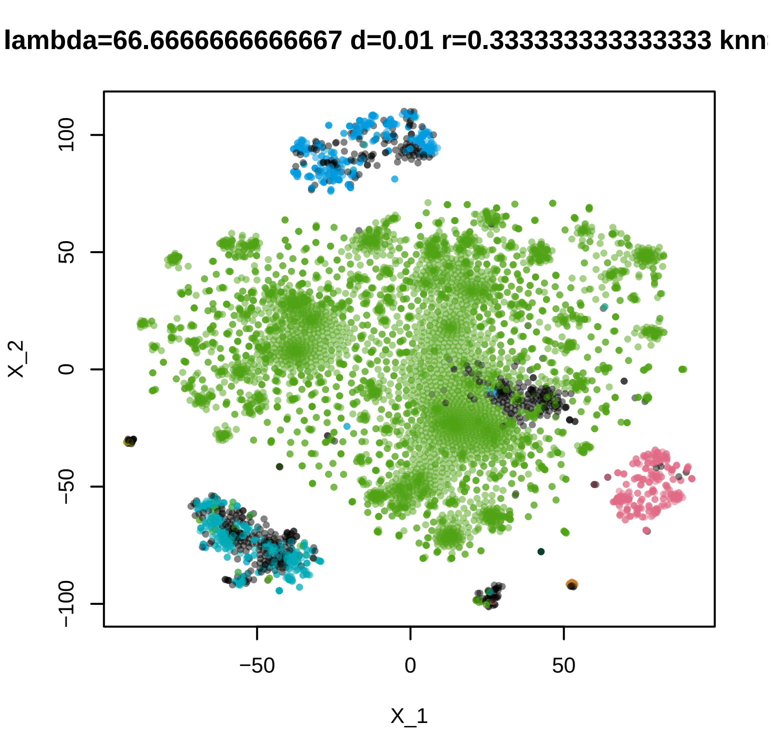
lambda=66.6666666666667 d=0.01 r=0.333333333333333 knn
X_1
X_2
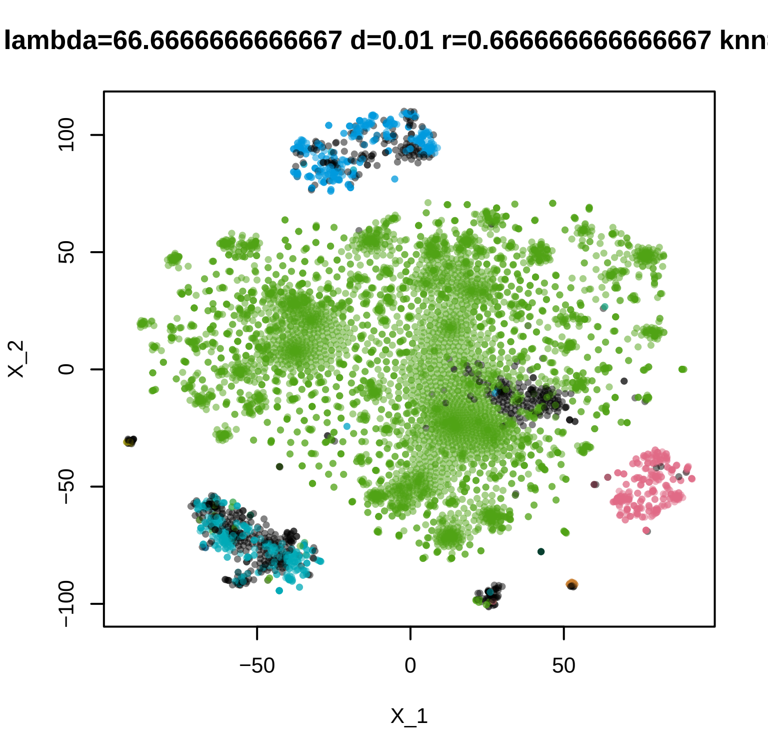
lambda=66.6666666666667 d=0.01 r=0.666666666666667
X_1
X_2
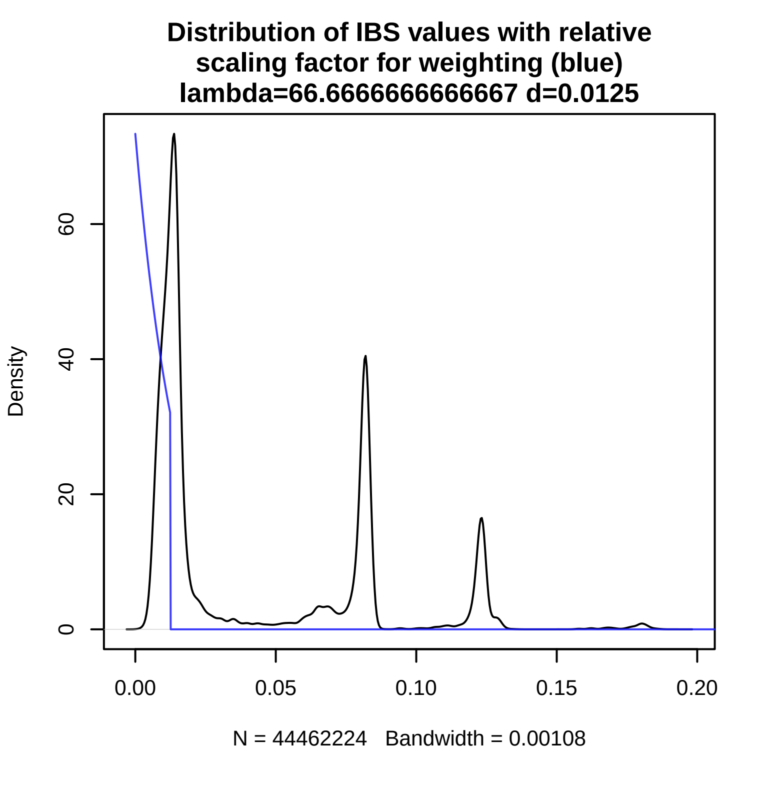
Distribution of IBS values with relative
scaling factor for weighting (blue)
lambda=66.6666666666667 d=0.0125
Density
N = 44462224 Bandwidth = 0.00108
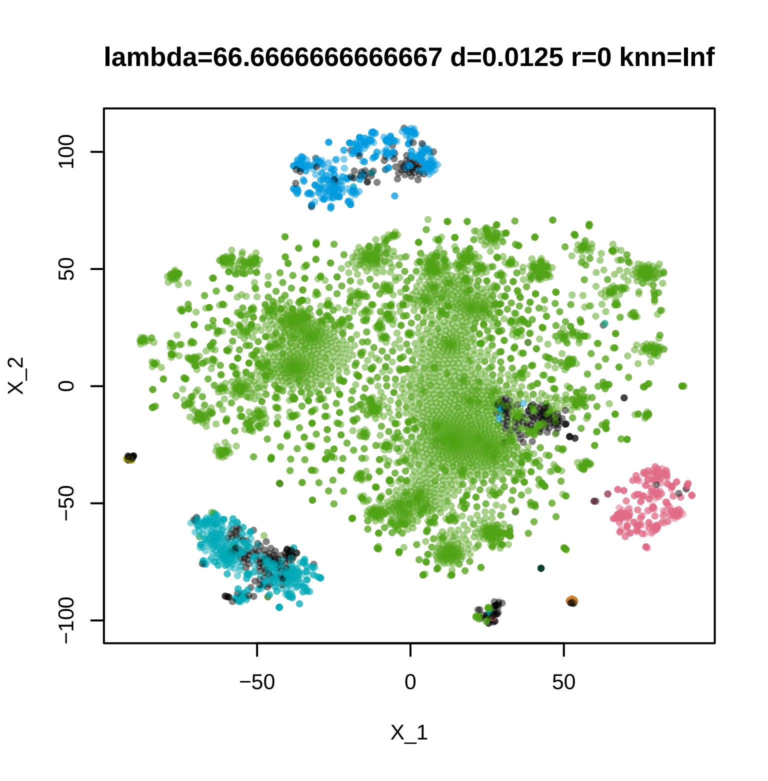
lambda=66.6666666666667 d=0.0125 r=0 knn=Inf
X_1
X_2
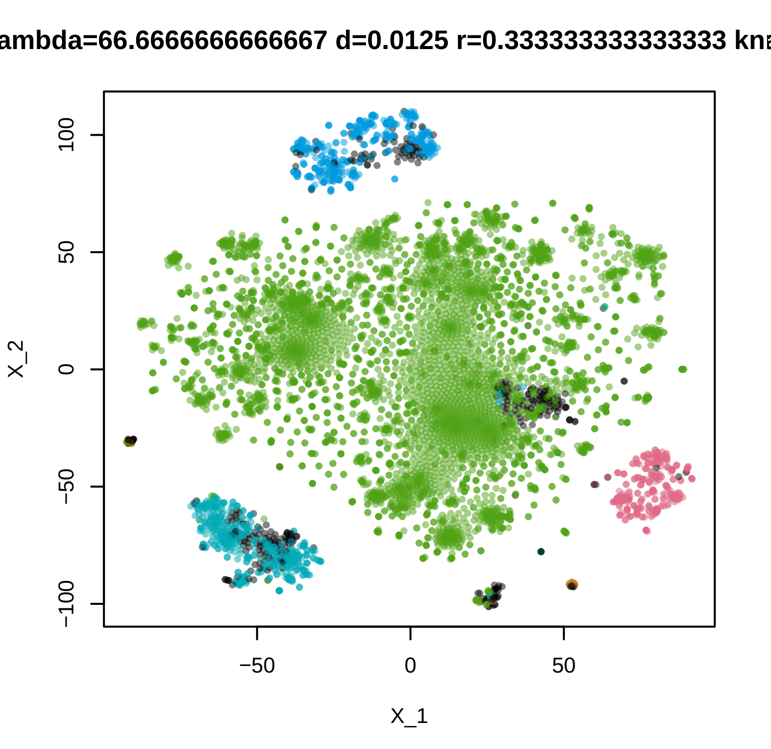
ambda=66.6666666666667 d=0.0125 r=0.333333333333333 kn
X_1
X_2
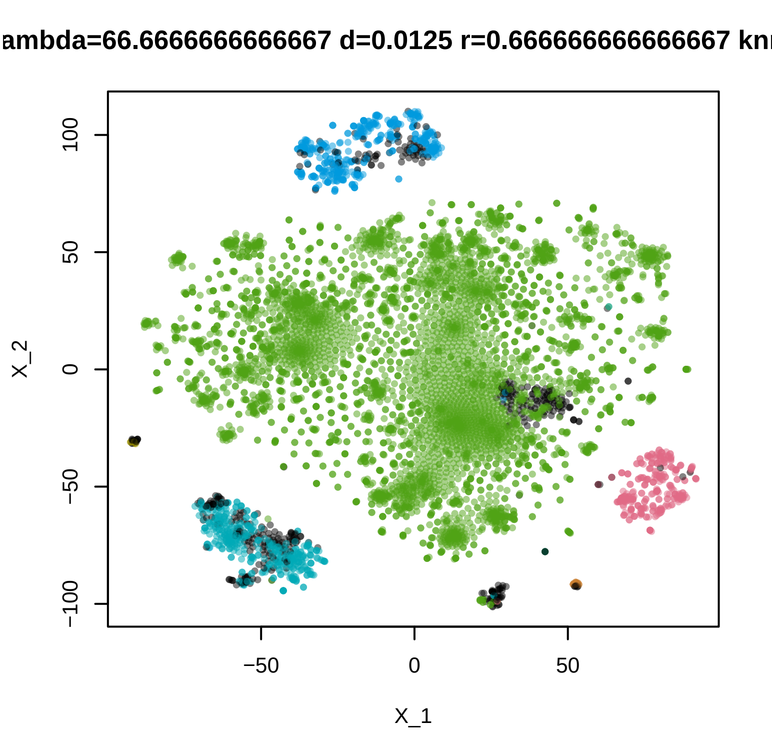
ambda=66.6666666666667 d=0.0125 r=0.666666666666667
X_1
X_2
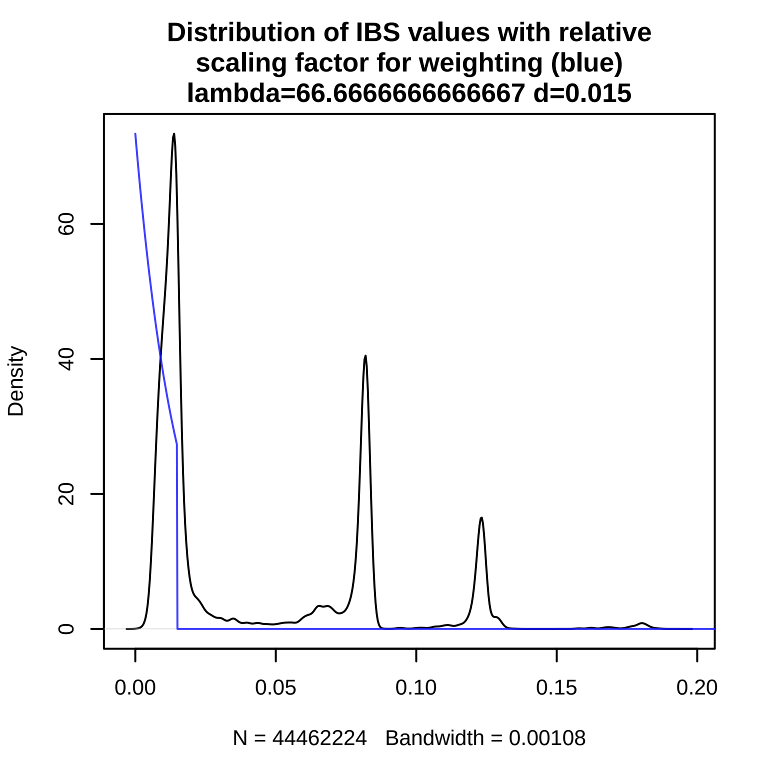
Distribution of IBS values with relative
scaling factor for weighting (blue)
lambda=66.6666666666667 d=0.015
Density
N = 44462224 Bandwidth = 0.00108
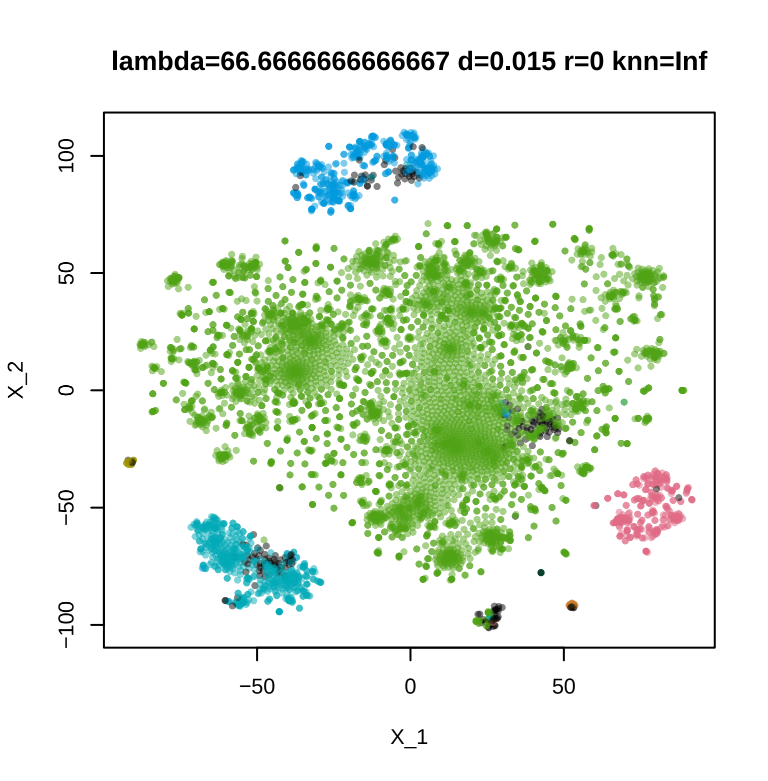
lambda=66.6666666666667 d=0.015 r=0 knn=Inf
X_1
X_2
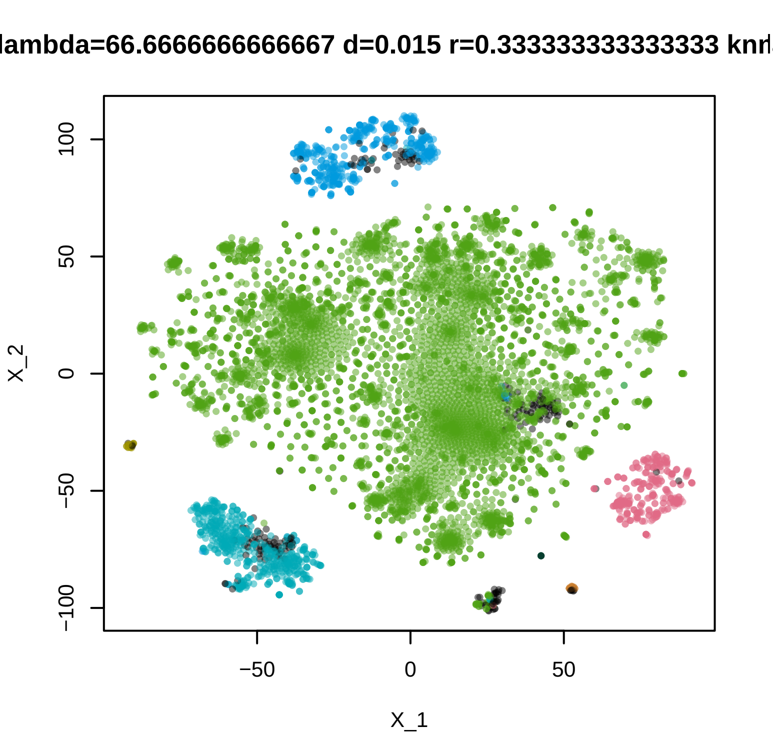
ambda=66.6666666666667 d=0.015 r=0.333333333333333 kn
X_1
X_2
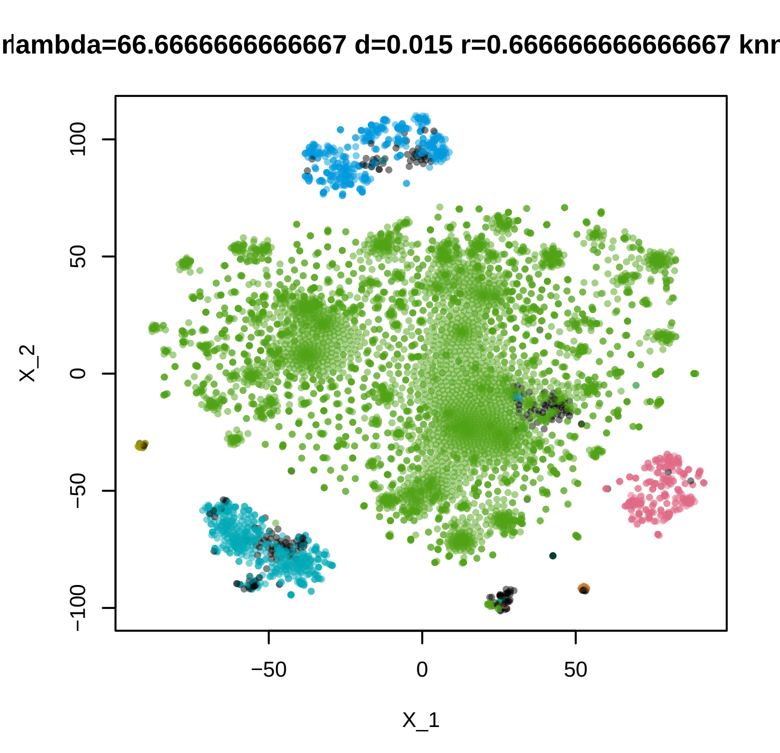
ambda=66.6666666666667 d=0.015 r=0.666666666666667
X_1
X_2
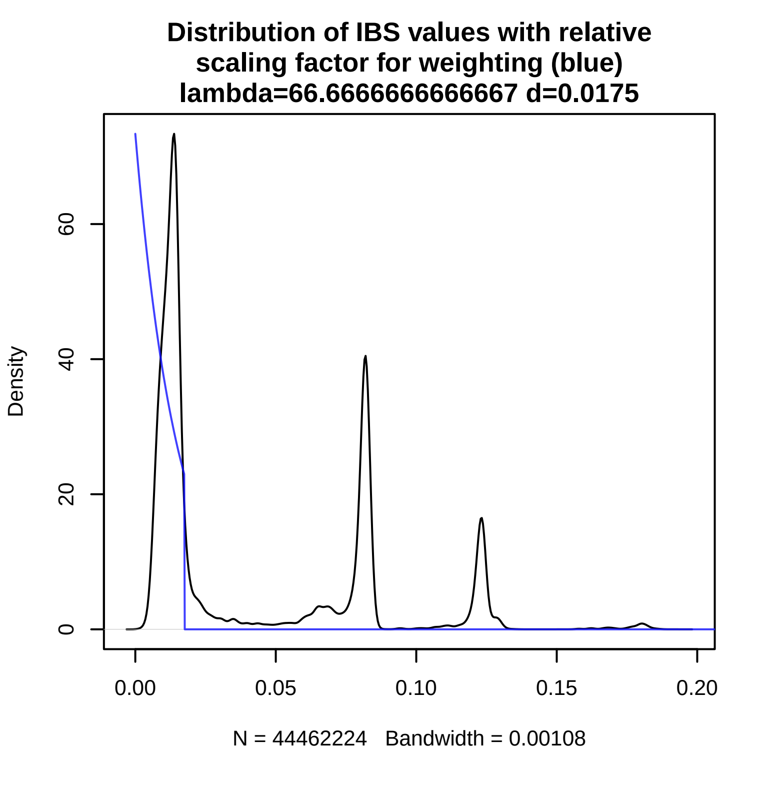
Distribution of IBS values with relative
scaling factor for weighting (blue)
lambda=66.6666666666667 d=0.0175
Density
N = 44462224 Bandwidth = 0.00108
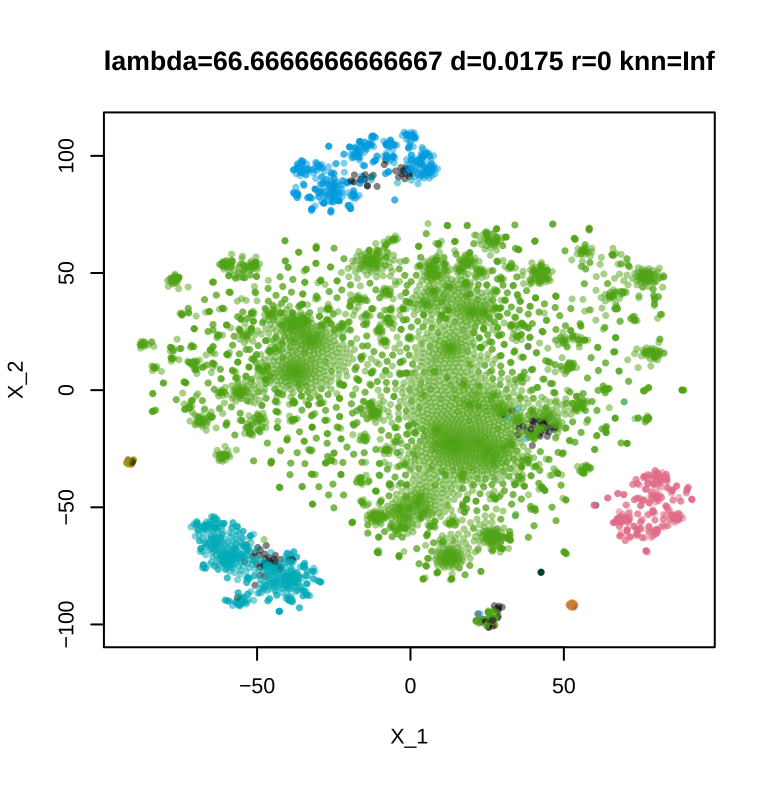
lambda=66.6666666666667 d=0.0175 r=0 knn=Inf
X_1
X_2
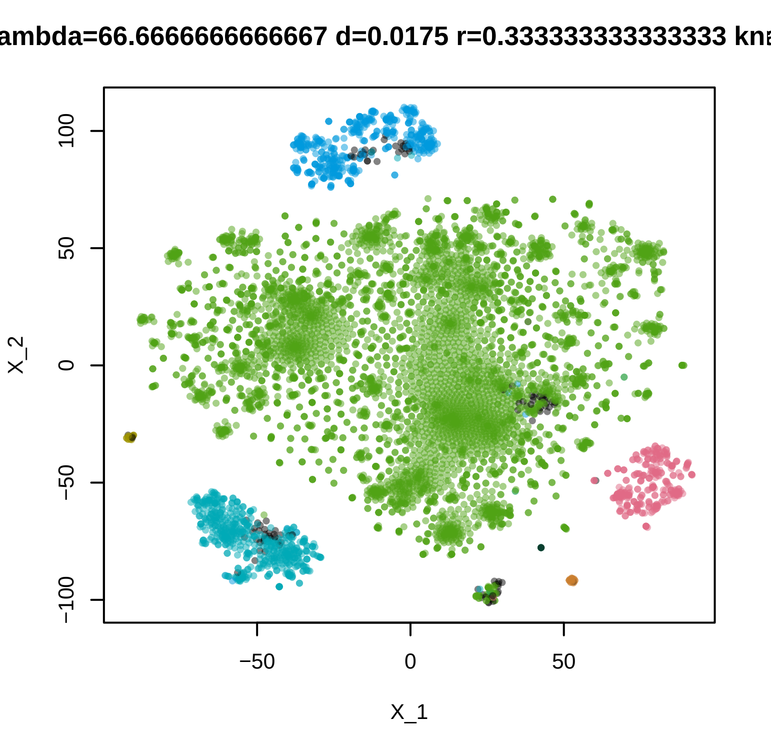
ambda=66.6666666666667 d=0.0175 r=0.333333333333333 kn
X_1
X_2
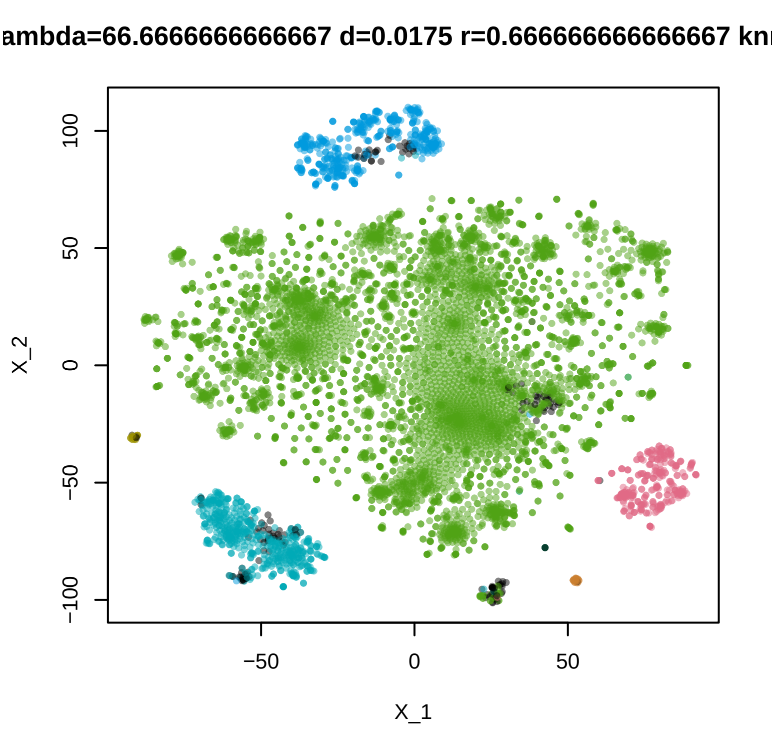
ambda=66.6666666666667 d=0.0175 r=0.666666666666667
X_1
X_2
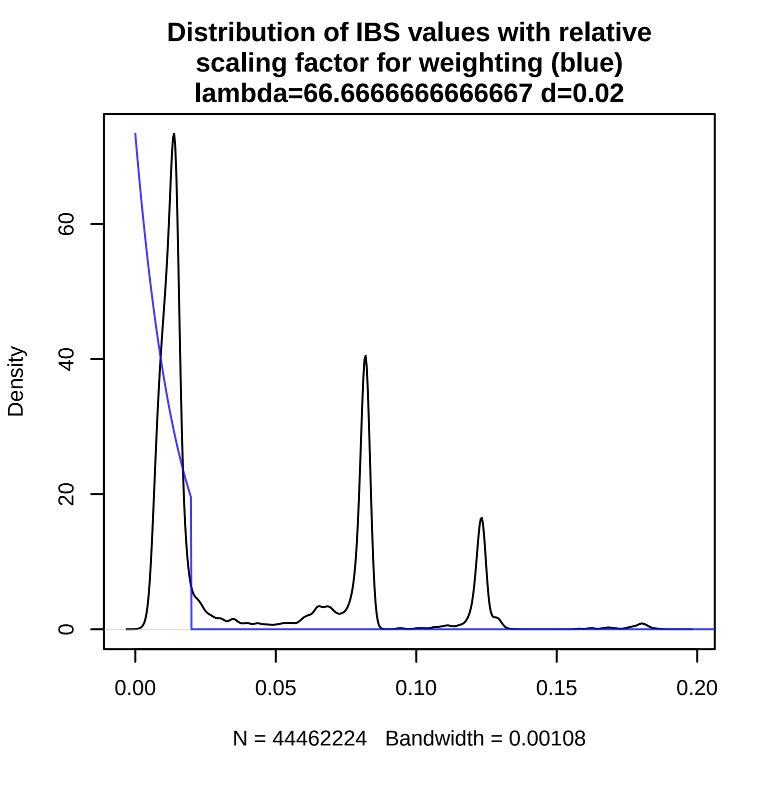
Distribution of IBS values with relative
scaling factor for weighting (blue)
lambda=66.6666666666667 d=0.02
Density
N = 44462224 Bandwidth = 0.00108
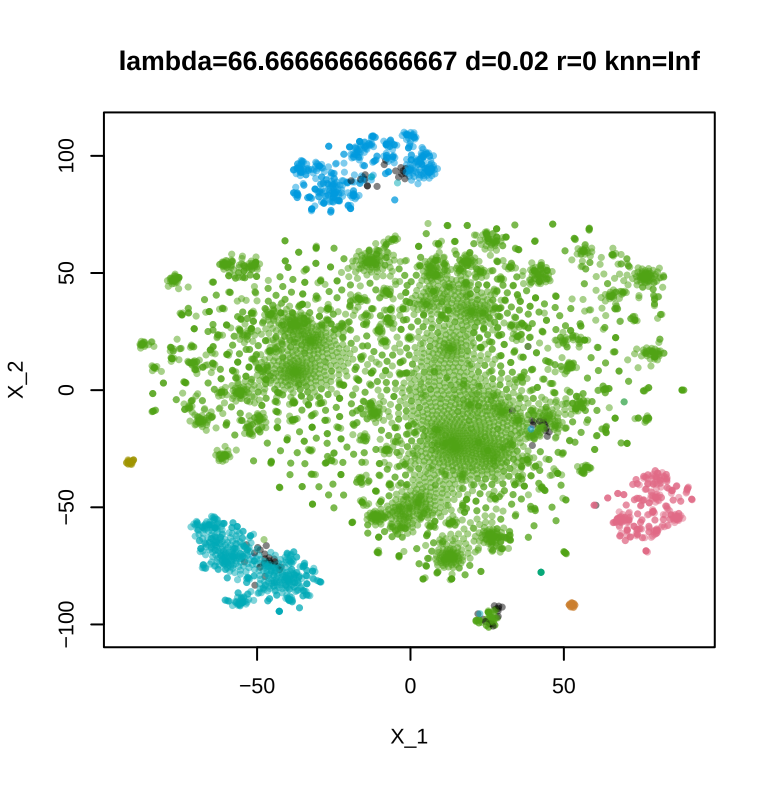
lambda=66.6666666666667 d=0.02 r=0 knn=Inf
X_1
X_2
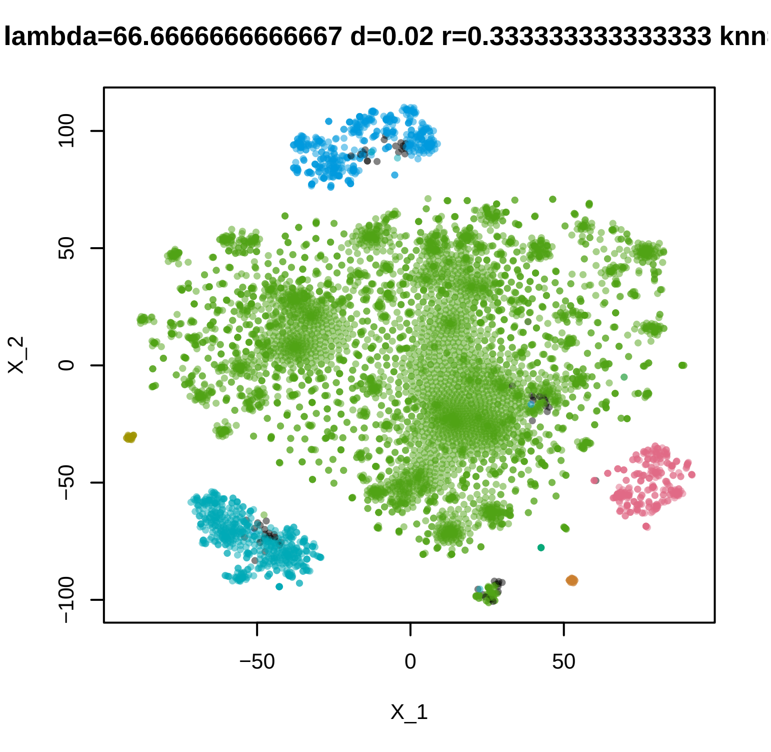
lambda=66.6666666666667 d=0.02 r=0.333333333333333 knn
X_1
X_2
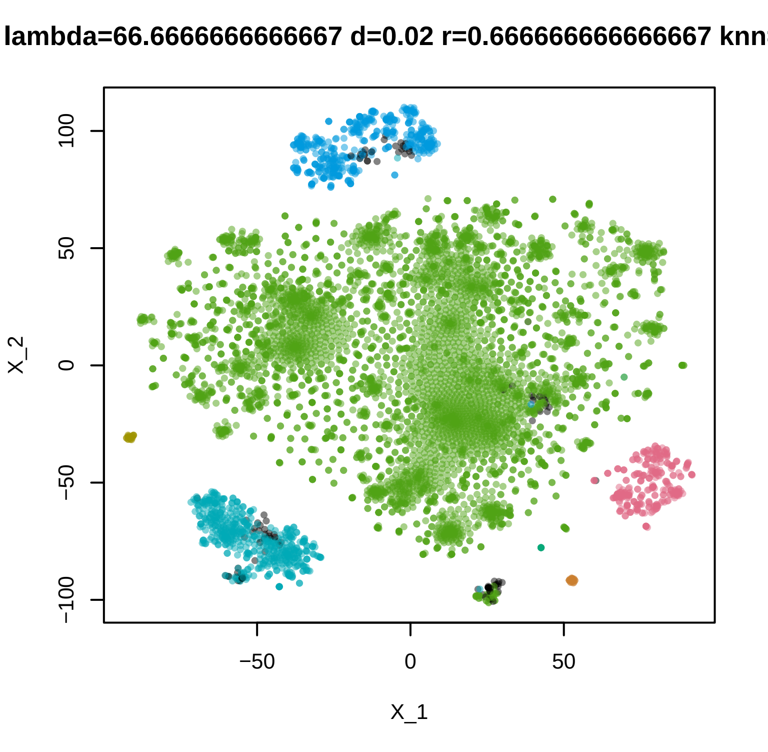
lambda=66.6666666666667 d=0.02 r=0.666666666666667
X_1
X_2

Distribution of IBS values with relative scaling factor for weighting (blue) lambda=66.6666666666667 d=0.0225

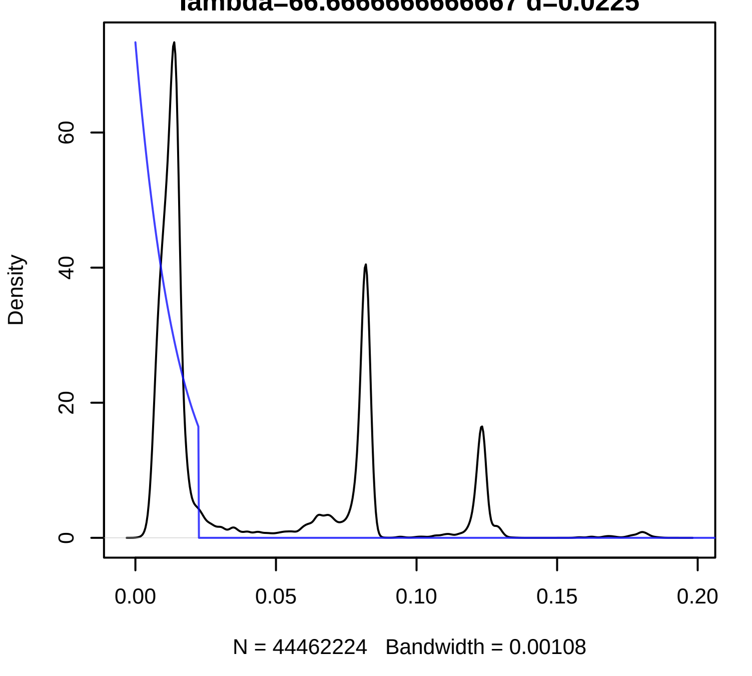

lambda=66.6666666666667 d=0.0225 r=0 knn=Inf

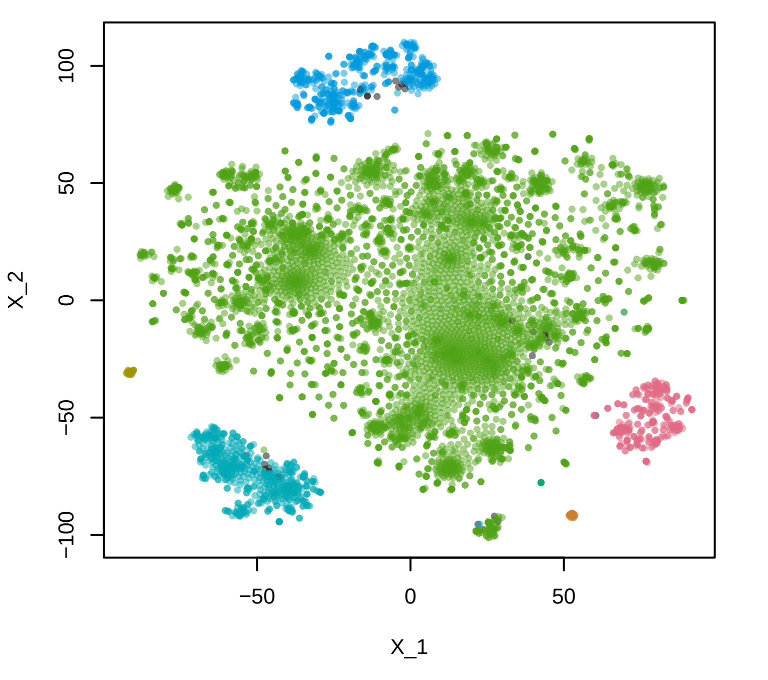

lambda=66.6666666666667 d=0.0225 r=0.333333333333333 knn

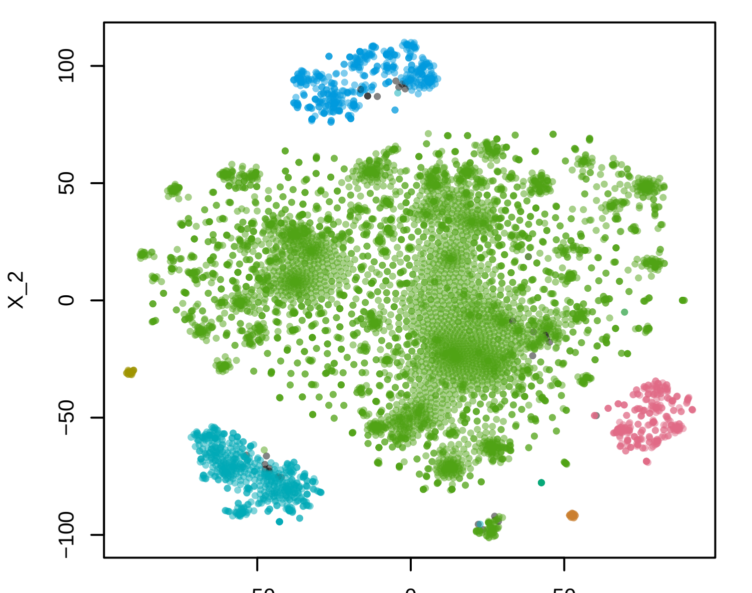

lambda=66.6666666666667 d=0.0225 r=0.666666666666667 kn

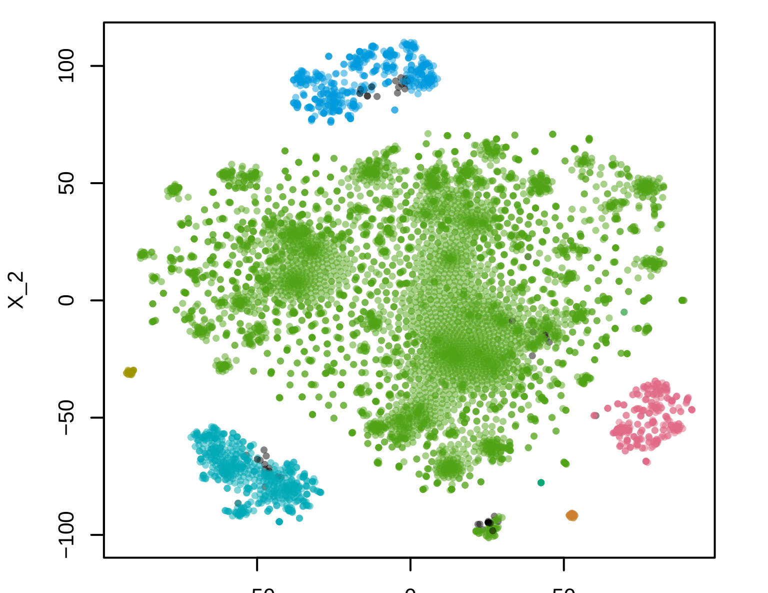

Distribution of IBS values with relative scaling factor for weighting (blue) lambda=66.6666666666667 d=0.025

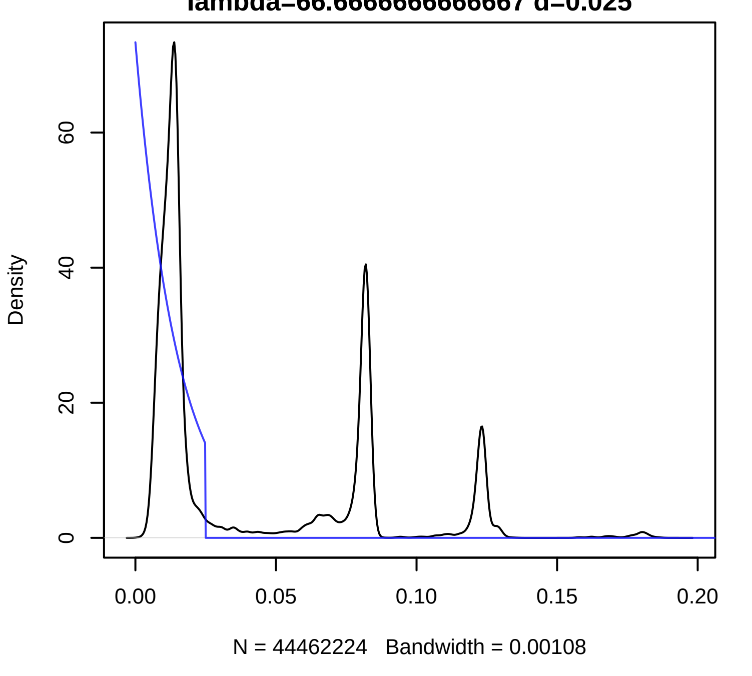

lambda=66.6666666666667 d=0.025 r=0 knn=Inf

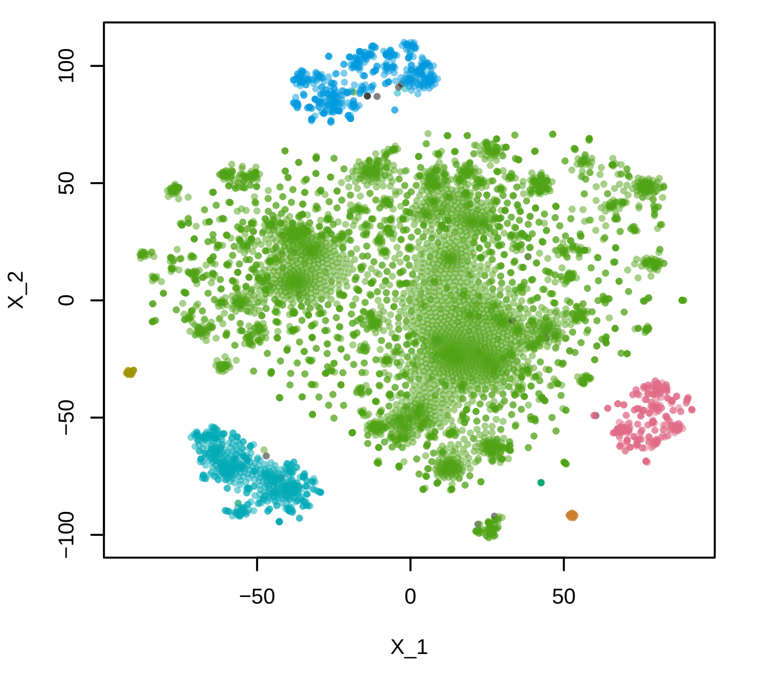

lambda=66.6666666666667 d=0.025 r=0.333333333333333 knn

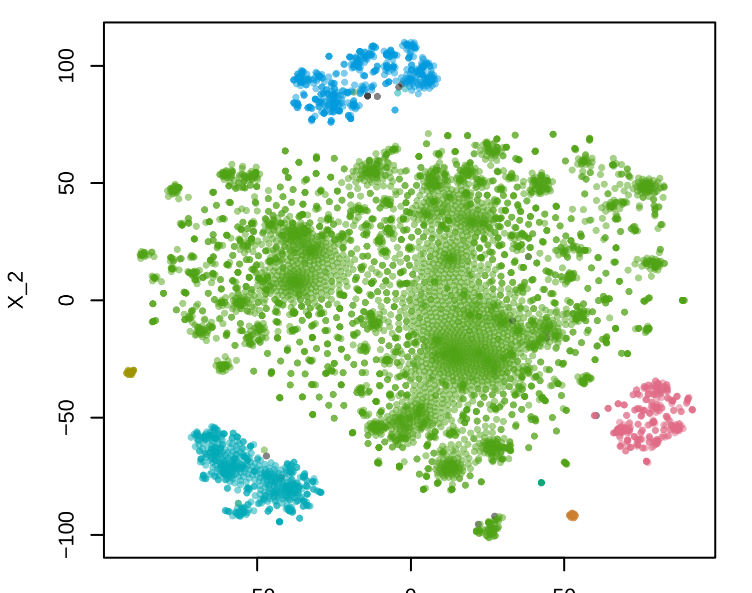

lambda=66.6666666666667 d=0.025 r=0.666666666666667 knn

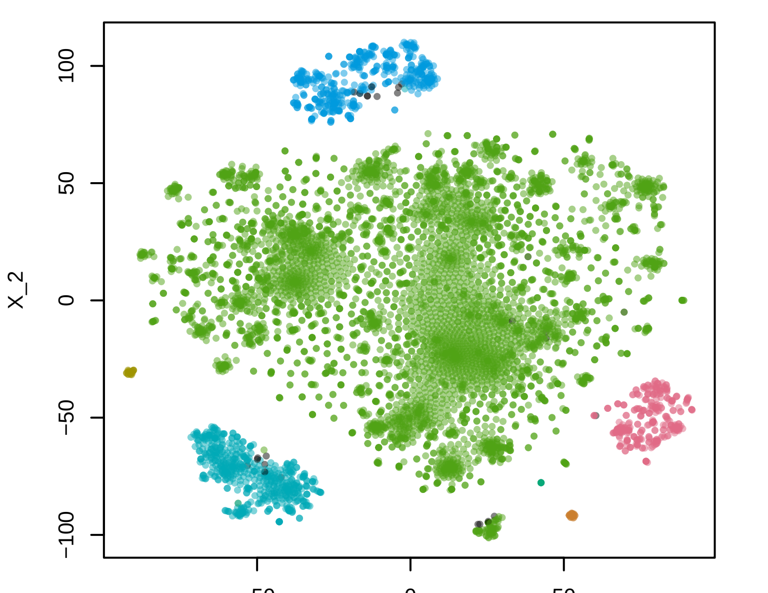

Distribution of IBS values with relative scaling factor for weighting (blue) lambda=66.6666666666667 d=0.0275

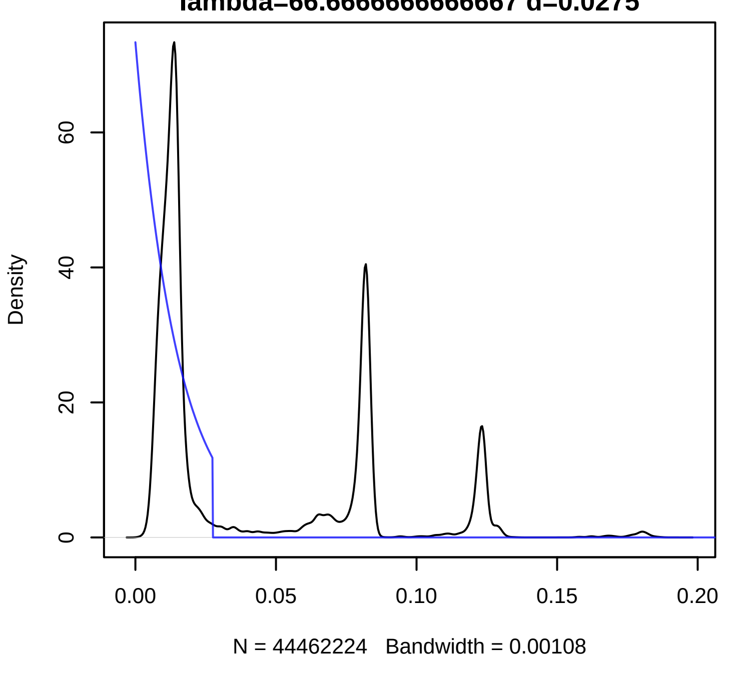

lambda=66.6666666666667 d=0.0275 r=0 knn=Inf

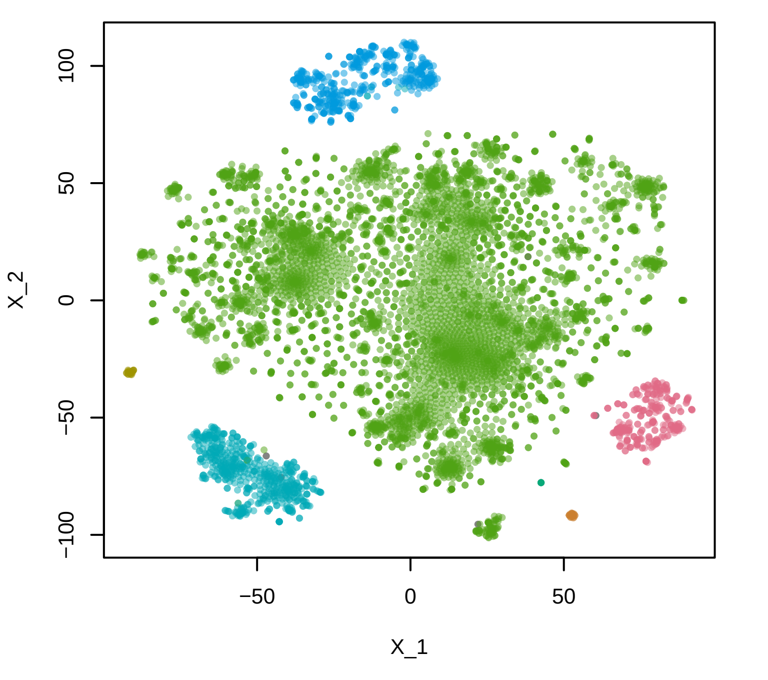

lambda=66.6666666666667 d=0.0275 r=0.333333333333333 knn

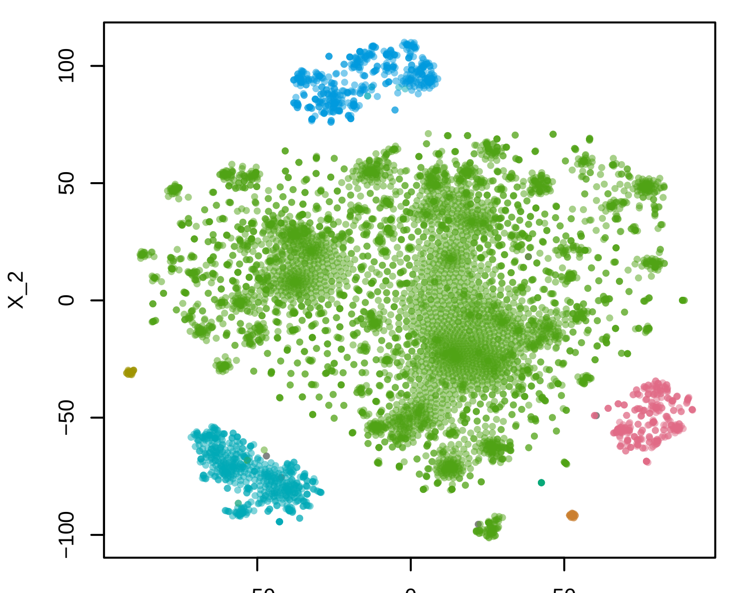

lambda=66.6666666666667 d=0.0275 r=0.666666666666667 knn

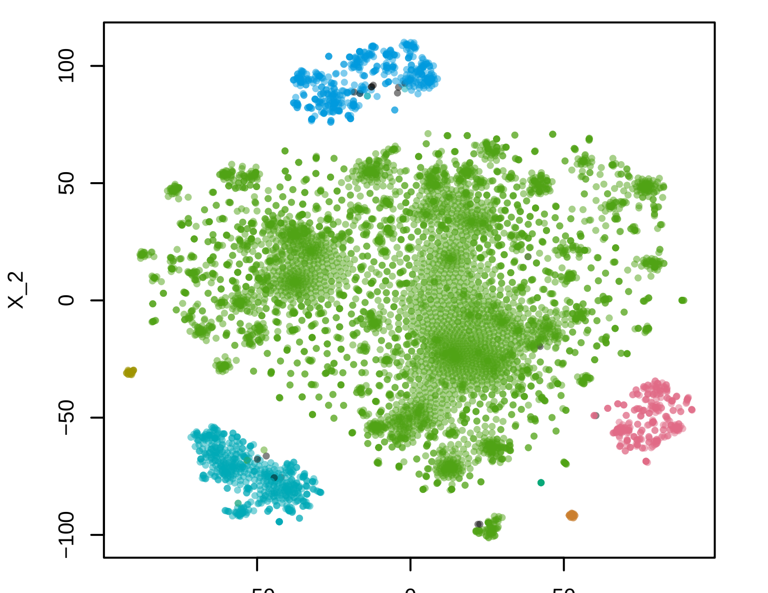

Distribution of IBS values with relative scaling factor for weighting (blue) lambda=66.6666666666667 d=0.03

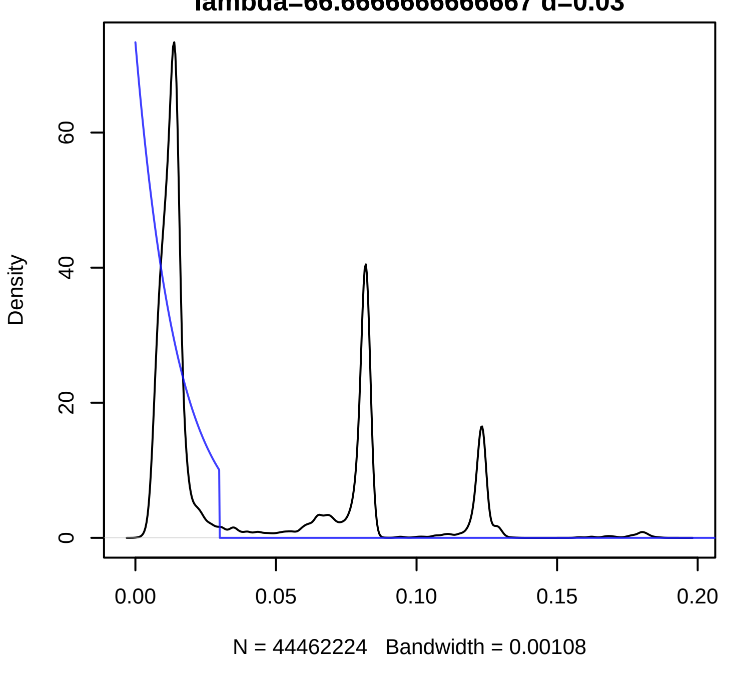

lambda=66.6666666666667 d=0.03 r=0 knn=Inf

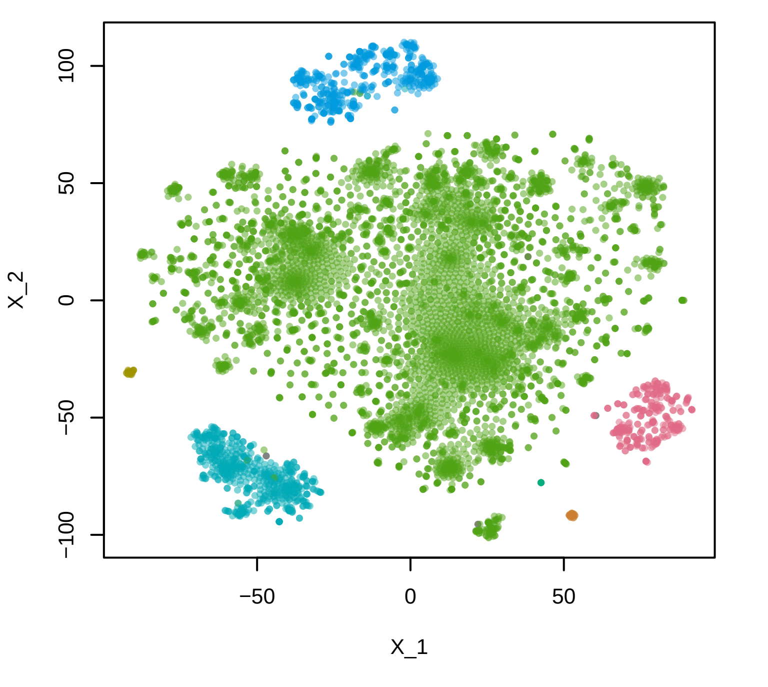

lambda=66.6666666666667 d=0.03 r=0.333333333333333 knn

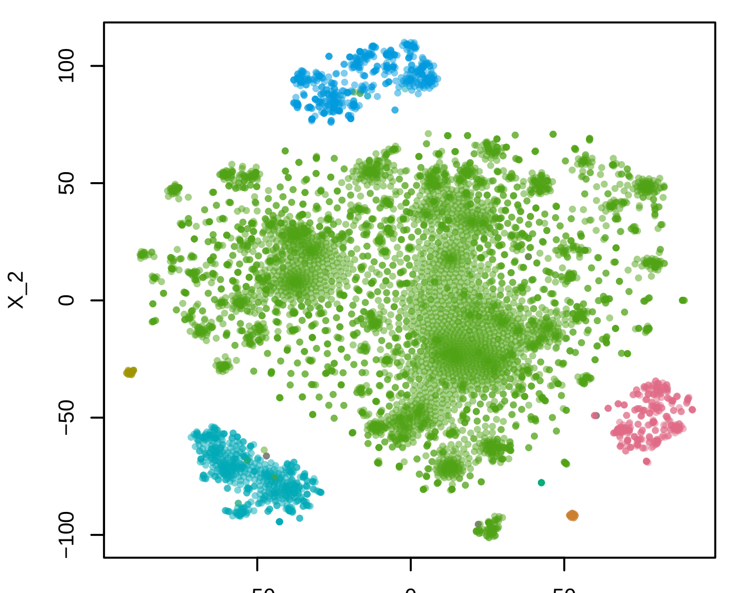

lambda=66.6666666666667 d=0.03 r=0.666666666666667 knn

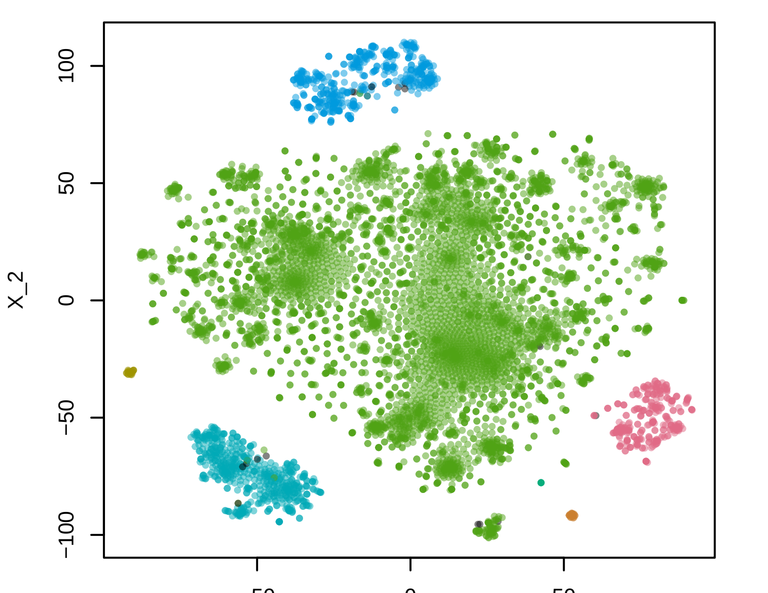

Distribution of IBS values with relative scaling factor for weighting (blue) lambda=133.333333333333 d=0.01

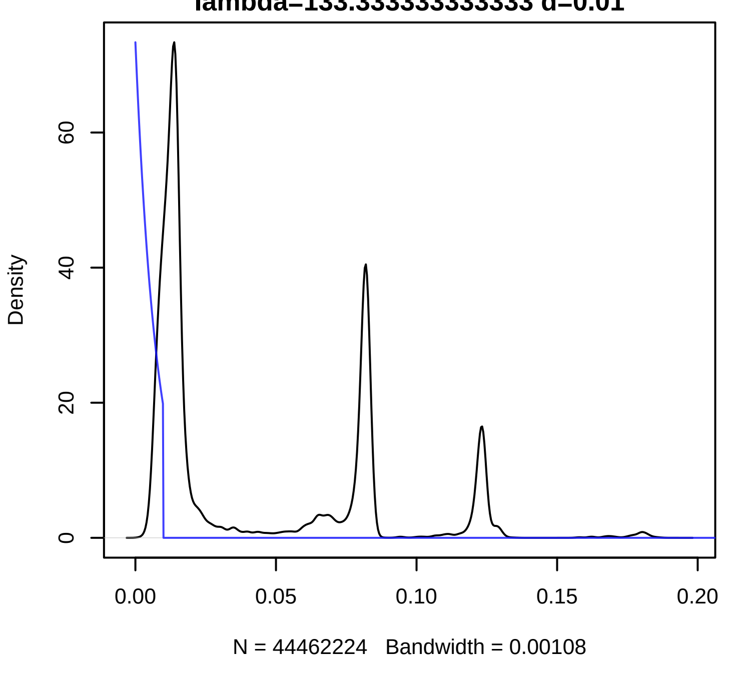

lambda=133.333333333333 d=0.01 r=0 knn=Inf

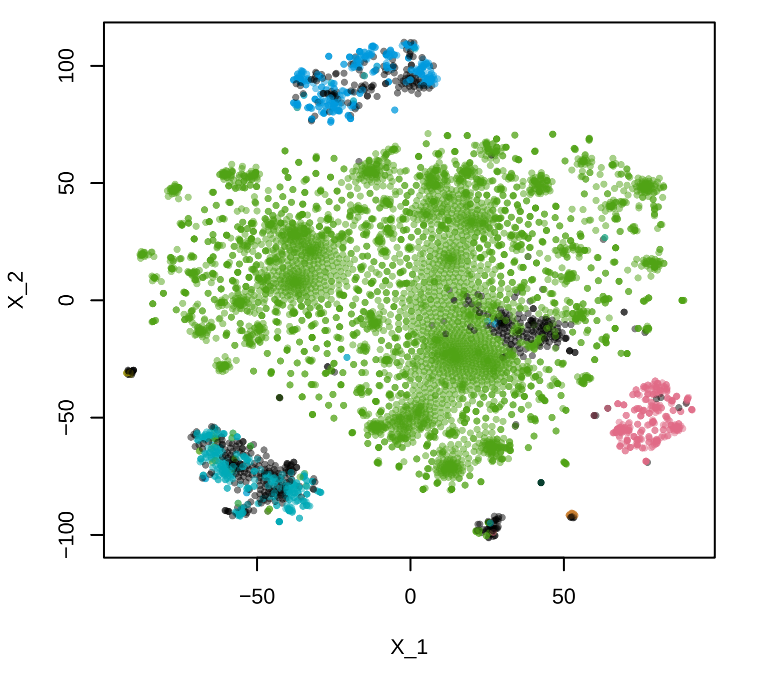

lambda=133.333333333333 d=0.01 r=0.333333333333333 knn

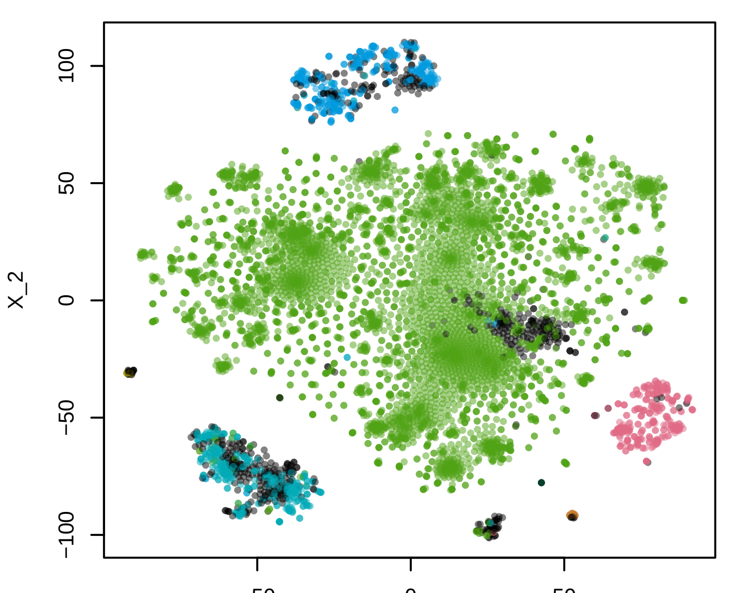

lambda=133.333333333333 d=0.01 r=0.666666666666667 knn

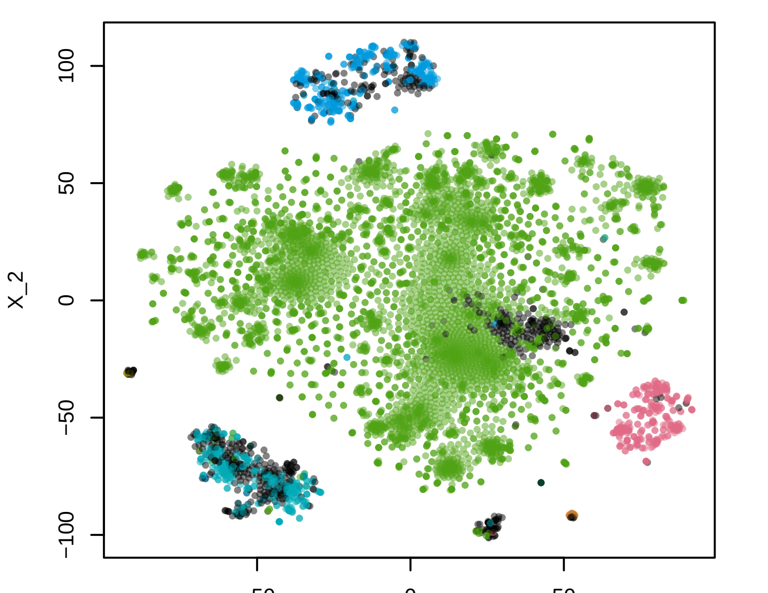

Distribution of IBS values with relative scaling factor for weighting (blue) lambda=133.333333333333 d=0.0125

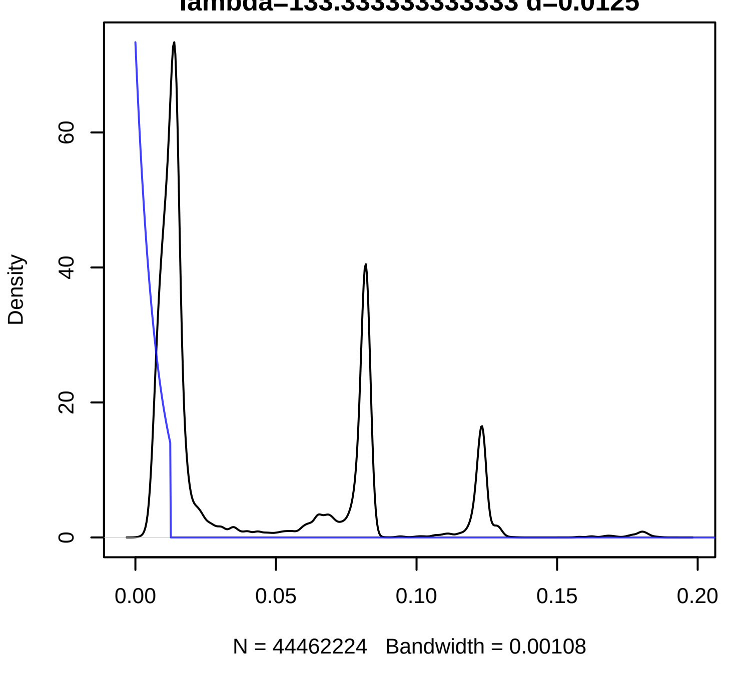

lambda=133.333333333333 d=0.0125 r=0 knn=Inf

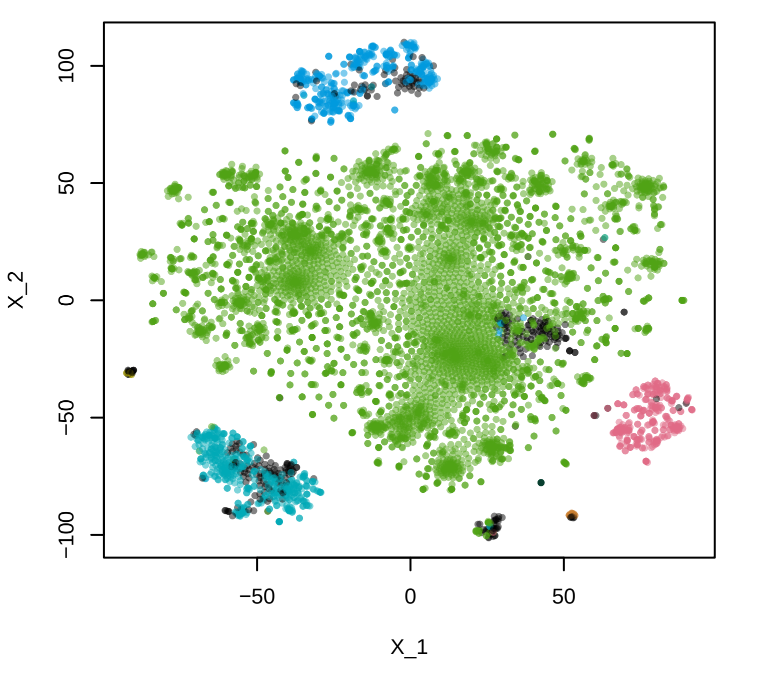

lambda=133.333333333333 d=0.0125 r=0.333333333333333 knn

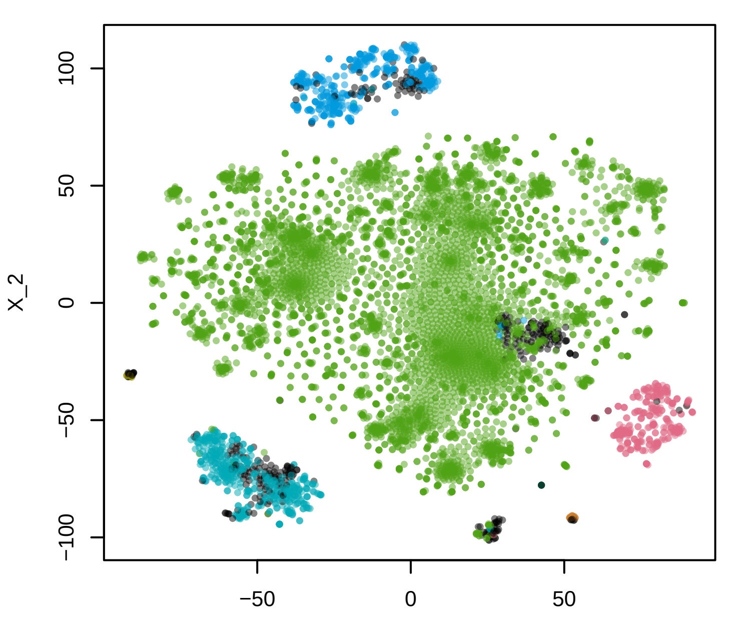

lambda=133.333333333333 d=0.0125 r=0.666666666666667 knn

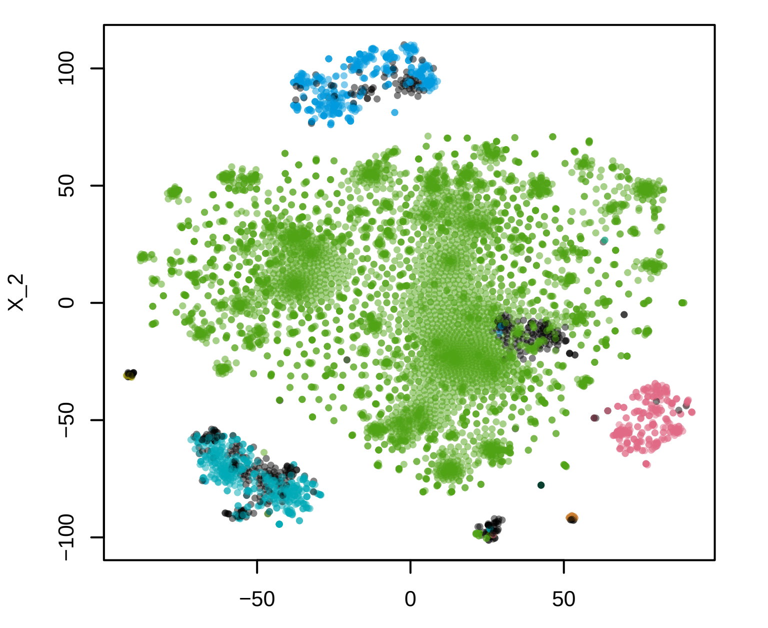

Distribution of IBS values with relative scaling factor for weighting (blue) lambda=133.333333333333 d=0.015

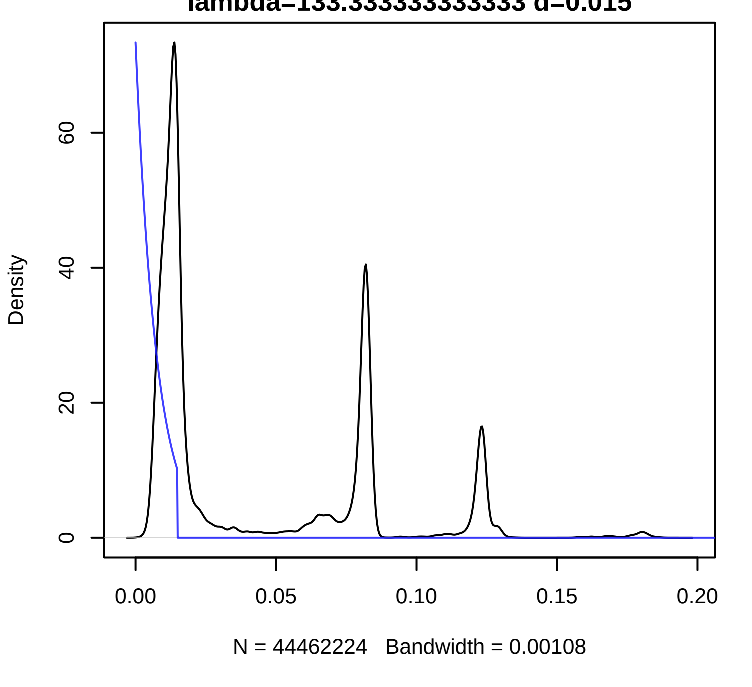

lambda=133.333333333333 d=0.015 r=0 knn=Inf

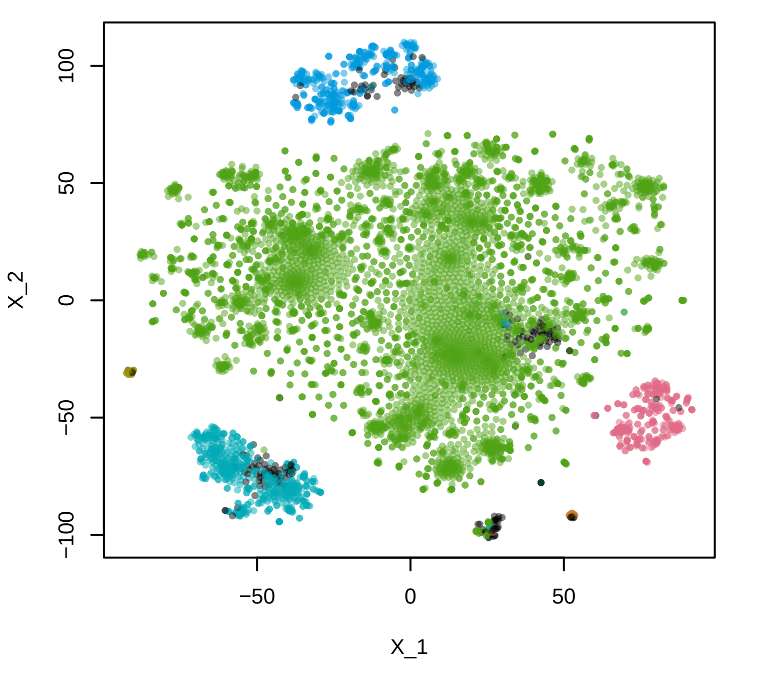

lambda=133.333333333333 d=0.015 r=0.333333333333333 kn

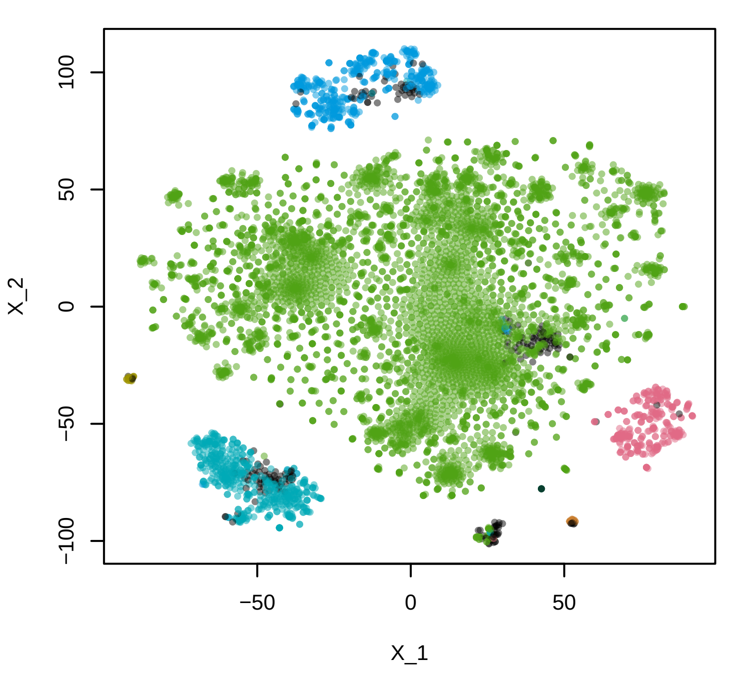

lambda=133.333333333333 d=0.015 r=0.666666666666667 kn

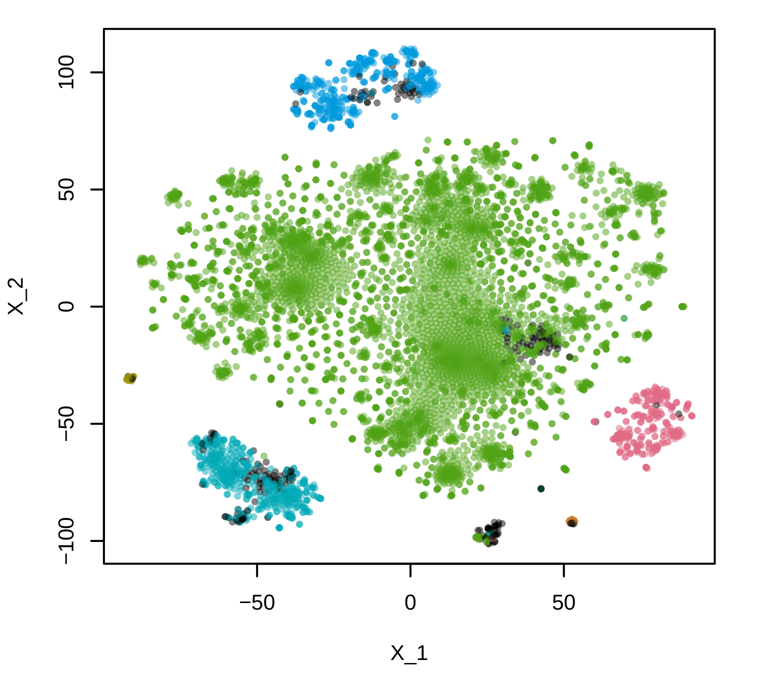

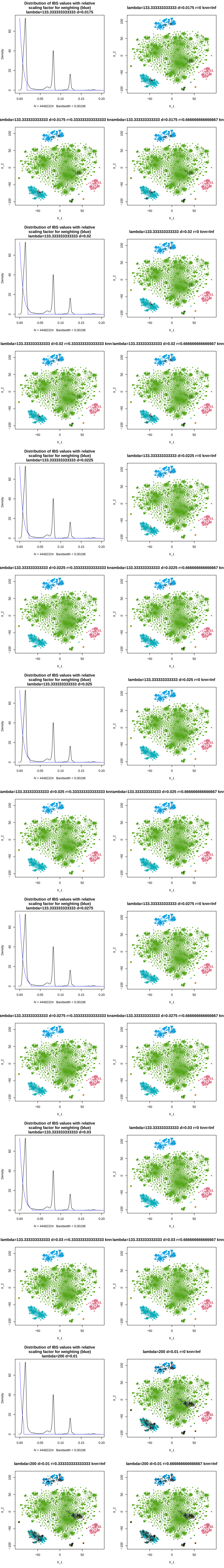
Distribution of IBS values with relative scaling factor for weighting (blue) lambda=133.333333333333 d=0.0175
Density
N = 44462224 Bandwidth = 0.00108
lambda=133.333333333333 d=0.0175 r=0 knn=Inf
X_1
X_2
lambda=133.333333333333 d=0.0175 r=0.333333333333333 knn
lambda=133.333333333333 d=0.0175 r=0.666666666666667 knn=Inf
Distribution of IBS values with relative scaling factor for weighting (blue) lambda=133.333333333333 d=0.02
lambda=133.333333333333 d=0.02 r=0 knn=Inf
lambda=133.333333333333 d=0.02 r=0.333333333333333 knn
lambda=133.333333333333 d=0.02 r=0.666666666666667 knn=Inf
Distribution of IBS values with relative scaling factor for weighting (blue) lambda=133.333333333333 d=0.0225
lambda=133.333333333333 d=0.0225 r=0 knn=Inf
lambda=133.333333333333 d=0.0225 r=0.333333333333333 knn
lambda=133.333333333333 d=0.0225 r=0.666666666666667 knn=Inf
Distribution of IBS values with relative scaling factor for weighting (blue) lambda=133.333333333333 d=0.025
lambda=133.333333333333 d=0.025 r=0 knn=Inf
lambda=133.333333333333 d=0.025 r=0.333333333333333 knn
lambda=133.333333333333 d=0.025 r=0.666666666666667 knn=Inf
Distribution of IBS values with relative scaling factor for weighting (blue) lambda=133.333333333333 d=0.0275
lambda=133.333333333333 d=0.0275 r=0 knn=Inf
lambda=133.333333333333 d=0.0275 r=0.333333333333333 knn
lambda=133.333333333333 d=0.0275 r=0.666666666666667 knn=Inf
Distribution of IBS values with relative scaling factor for weighting (blue) lambda=133.333333333333 d=0.03
lambda=133.333333333333 d=0.03 r=0 knn=Inf
lambda=133.333333333333 d=0.03 r=0.333333333333333 knn
lambda=133.333333333333 d=0.03 r=0.666666666666667 knn=Inf
Distribution of IBS values with relative scaling factor for weighting (blue) lambda=200 d=0.01
lambda=200 d=0.01 r=0 knn=Inf
lambda=200 d=0.01 r=0.333333333333333 knn=Inf
lambda=200 d=0.01 r=0.666666666666667 knn=Inf

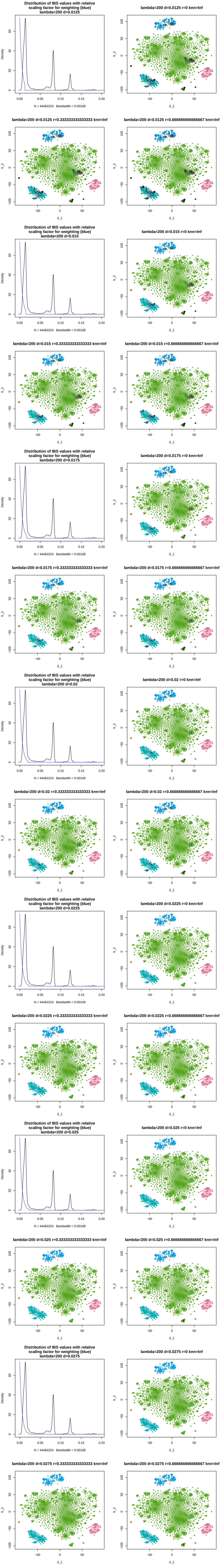

Distribution of IBS values with relative scaling factor for weighting (blue) lambda=200 d=0.0125
Density
N = 44462224 Bandwidth = 0.00108
lambda=200 d=0.0125 r=0 knn=Inf
X_1
X_2
lambda=200 d=0.0125 r=0.333333333333333 knn=Inf
lambda=200 d=0.0125 r=0.666666666666667 knn=Inf
Distribution of IBS values with relative scaling factor for weighting (blue) lambda=200 d=0.015
N = 44462224 Bandwidth = 0.00108
lambda=200 d=0.015 r=0 knn=Inf
lambda=200 d=0.015 r=0.333333333333333 knn=Inf
lambda=200 d=0.015 r=0.666666666666667 knn=Inf
Distribution of IBS values with relative scaling factor for weighting (blue) lambda=200 d=0.0175
N = 44462224 Bandwidth = 0.00108
lambda=200 d=0.0175 r=0 knn=Inf
lambda=200 d=0.0175 r=0.333333333333333 knn=Inf
lambda=200 d=0.0175 r=0.666666666666667 knn=Inf
Distribution of IBS values with relative scaling factor for weighting (blue) lambda=200 d=0.02
N = 44462224 Bandwidth = 0.00108
lambda=200 d=0.02 r=0 knn=Inf
lambda=200 d=0.02 r=0.333333333333333 knn=Inf
lambda=200 d=0.02 r=0.666666666666667 knn=Inf
Distribution of IBS values with relative scaling factor for weighting (blue) lambda=200 d=0.0225
N = 44462224 Bandwidth = 0.00108
lambda=200 d=0.0225 r=0 knn=Inf
lambda=200 d=0.0225 r=0.333333333333333 knn=Inf
lambda=200 d=0.0225 r=0.666666666666667 knn=Inf
Distribution of IBS values with relative scaling factor for weighting (blue) lambda=200 d=0.025
N = 44462224 Bandwidth = 0.00108
lambda=200 d=0.025 r=0 knn=Inf
lambda=200 d=0.025 r=0.333333333333333 knn=Inf
lambda=200 d=0.025 r=0.666666666666667 knn=Inf
Distribution of IBS values with relative scaling factor for weighting (blue) lambda=200 d=0.0275
N = 44462224 Bandwidth = 0.00108
lambda=200 d=0.0275 r=0 knn=Inf
lambda=200 d=0.0275 r=0.333333333333333 knn=Inf
lambda=200 d=0.0275 r=0.666666666666667 knn=Inf

Distribution of IBS values with relative scaling factor for weighting (blue)
lambda=200 d=0.03

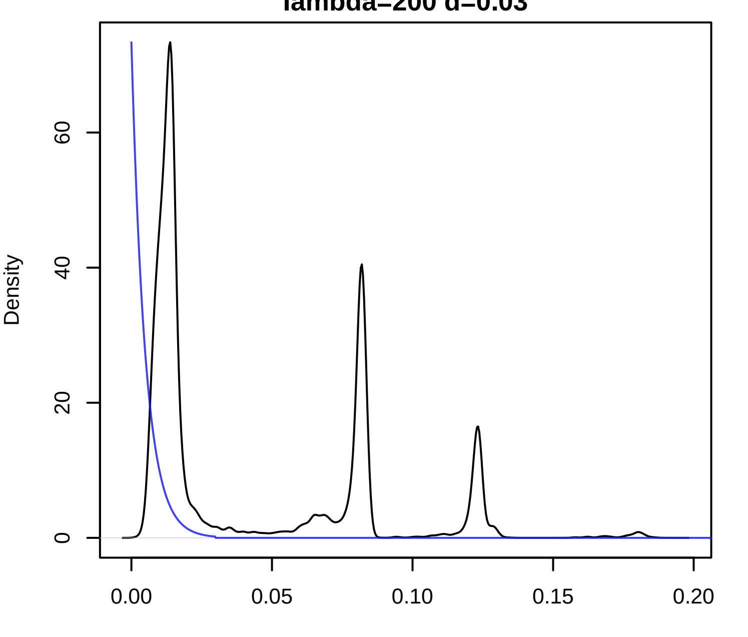

lambda=200 d=0.03 r=0 knn=Inf

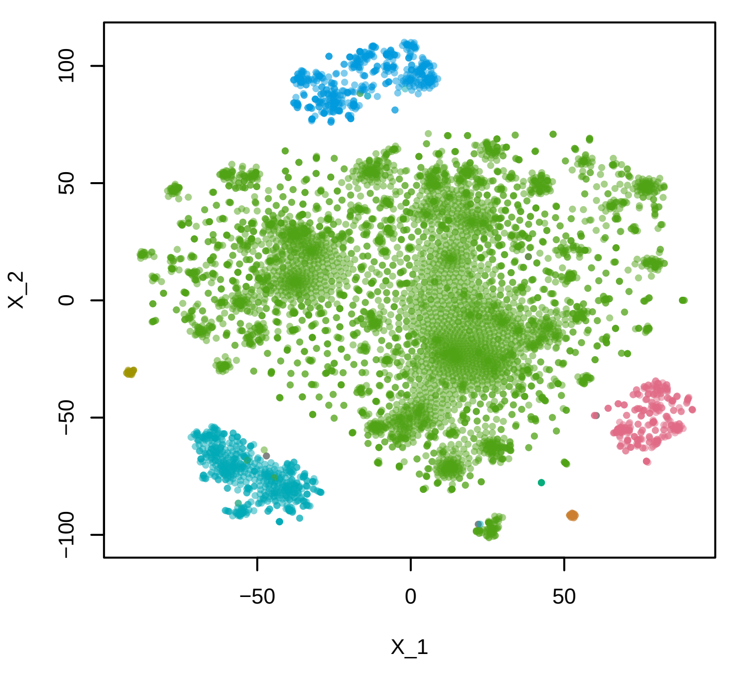

lambda=200 d=0.03 r=0.333333333333333 knn=Inf

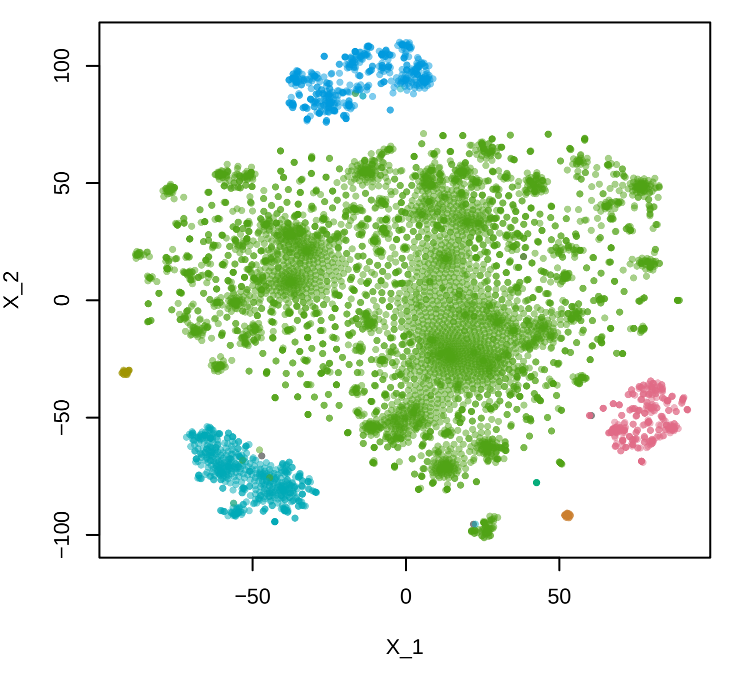

lambda=200 d=0.03 r=0.666666666666667 knn=Inf

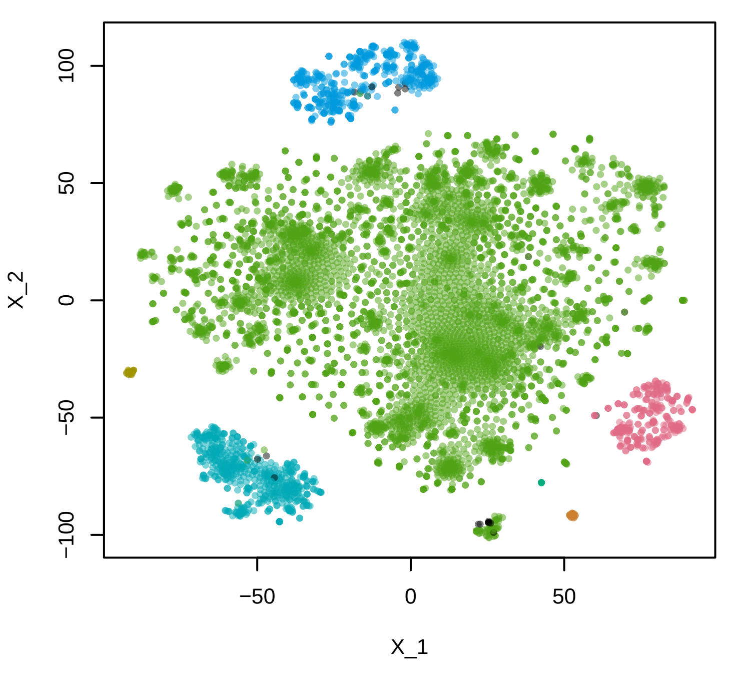

Distribution of IBS values with relative scaling factor for weighting (blue)
lambda=266.666666666667 d=0.01

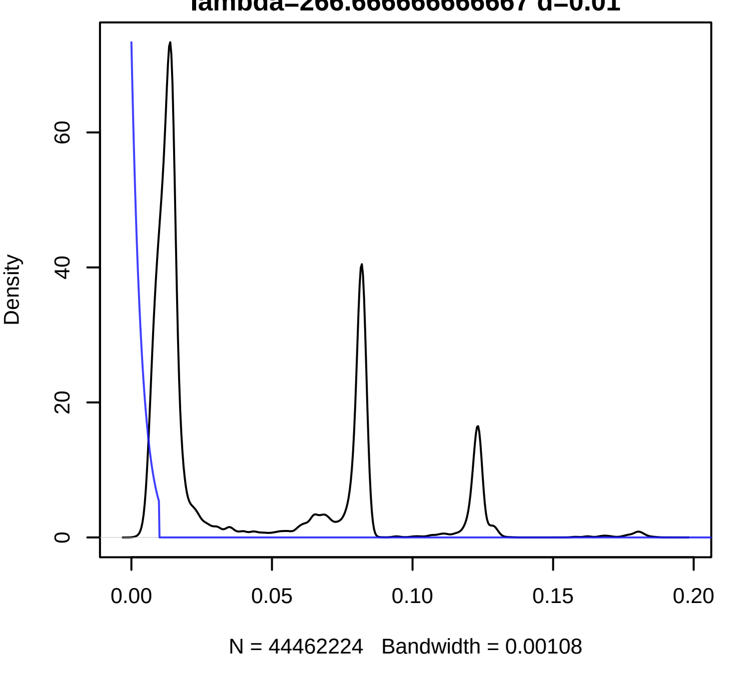

lambda=266.666666666667 d=0.01 r=0 knn=Inf

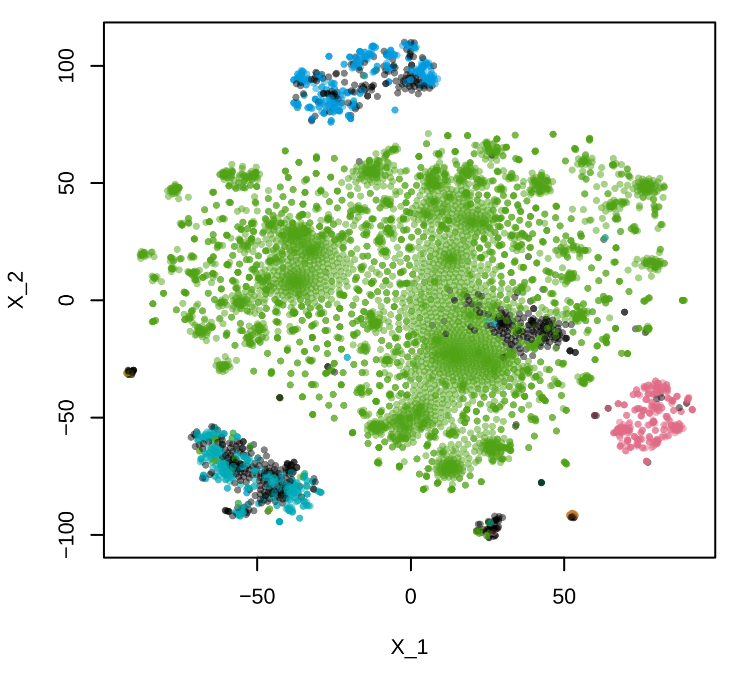

lambda=266.666666666667 d=0.01 r=0.333333333333333 knn

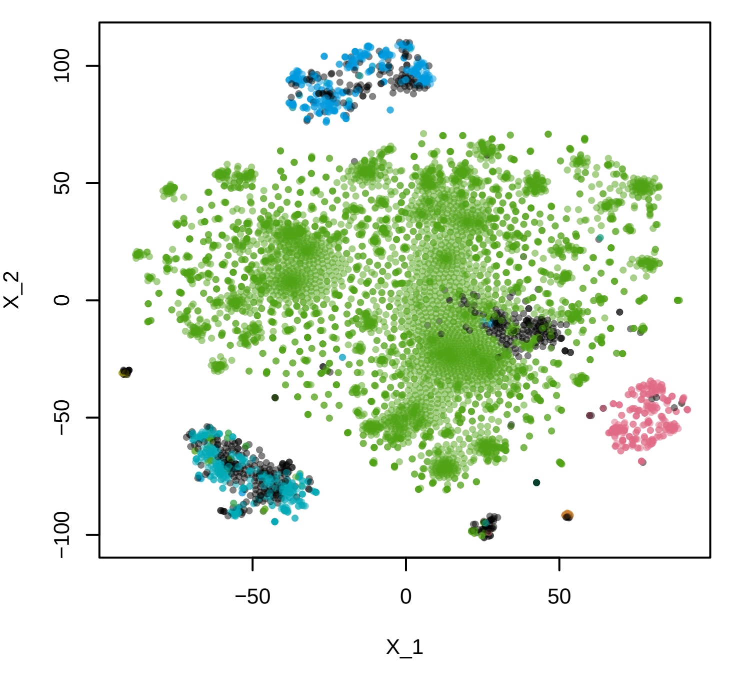

lambda=266.666666666667 d=0.01 r=0.666666666666667 knn

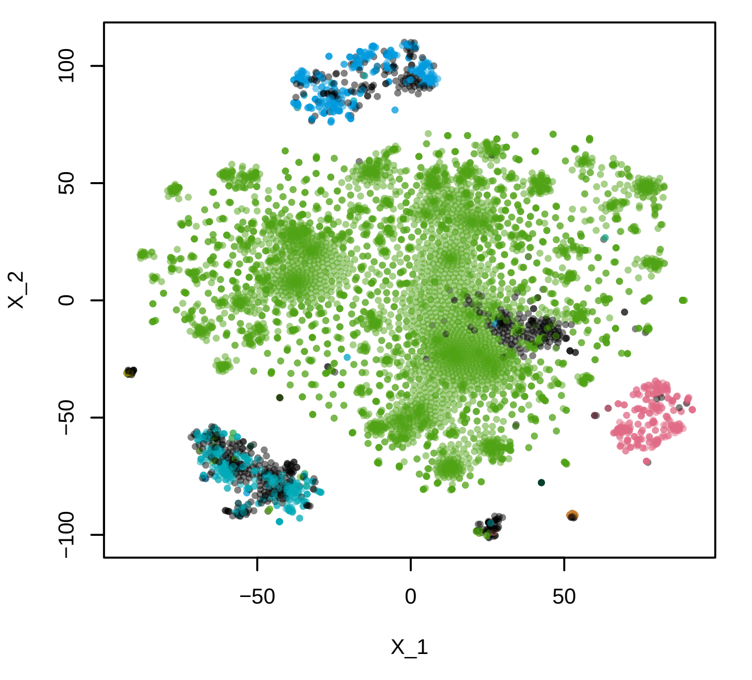

Distribution of IBS values with relative scaling factor for weighting (blue)
lambda=266.666666666667 d=0.0125

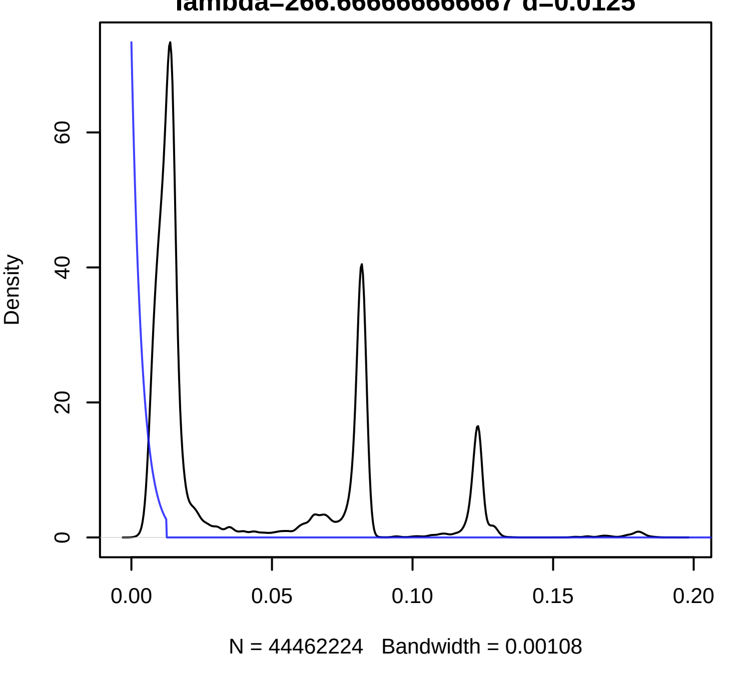

lambda=266.666666666667 d=0.0125 r=0 knn=Inf

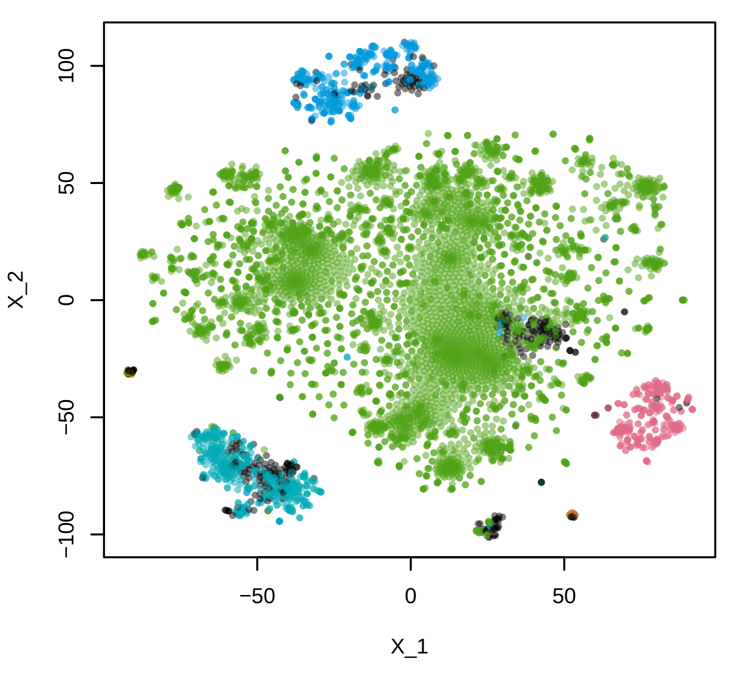

lambda=266.666666666667 d=0.0125 r=0.333333333333333 kn

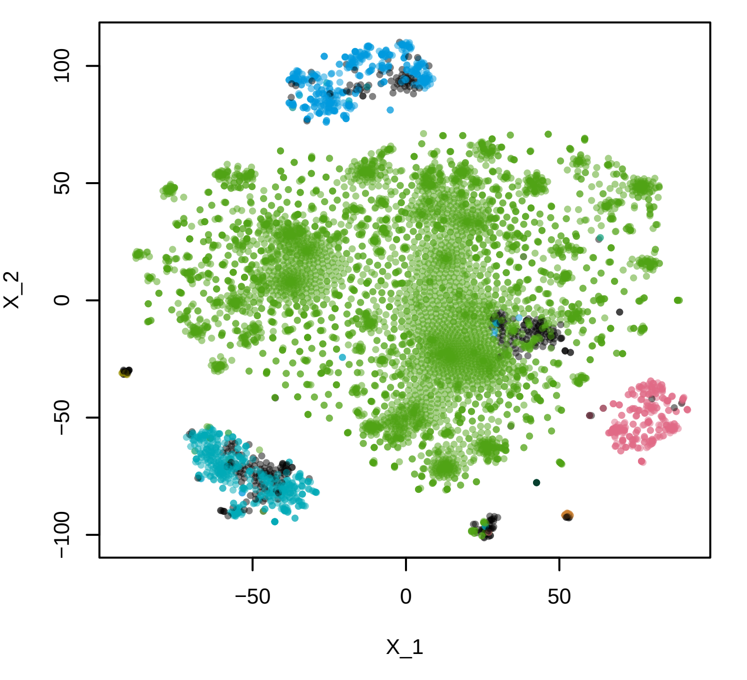

lambda=266.666666666667 d=0.0125 r=0.666666666666667 kn

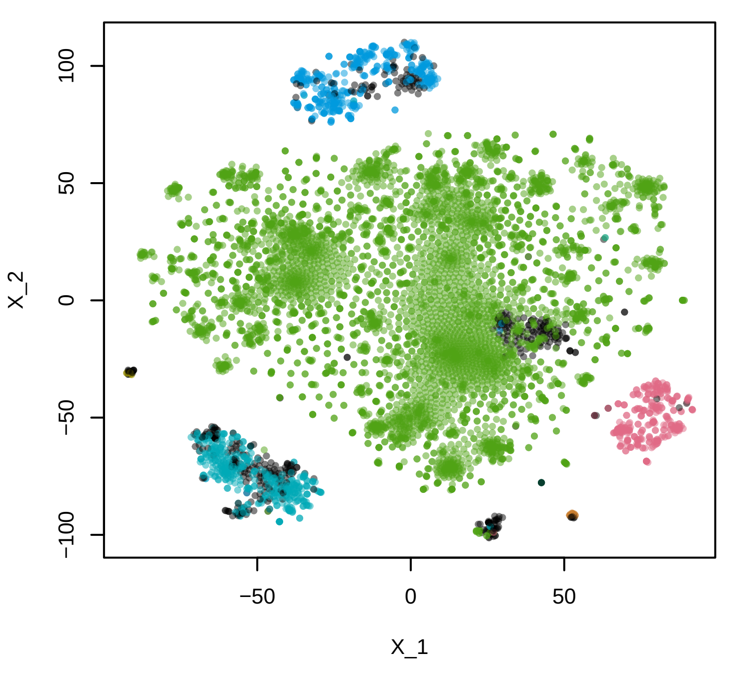

Distribution of IBS values with relative scaling factor for weighting (blue)
lambda=266.666666666667 d=0.015

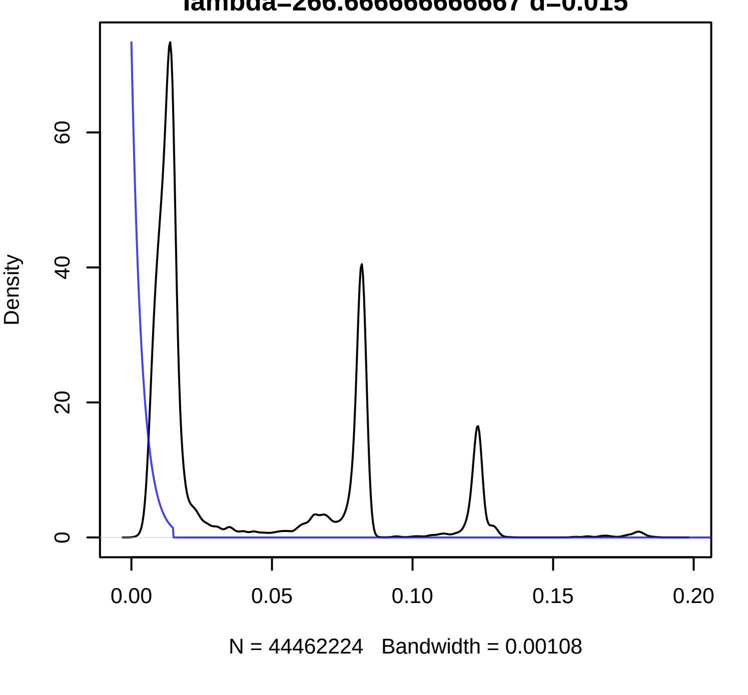

lambda=266.666666666667 d=0.015 r=0 knn=Inf

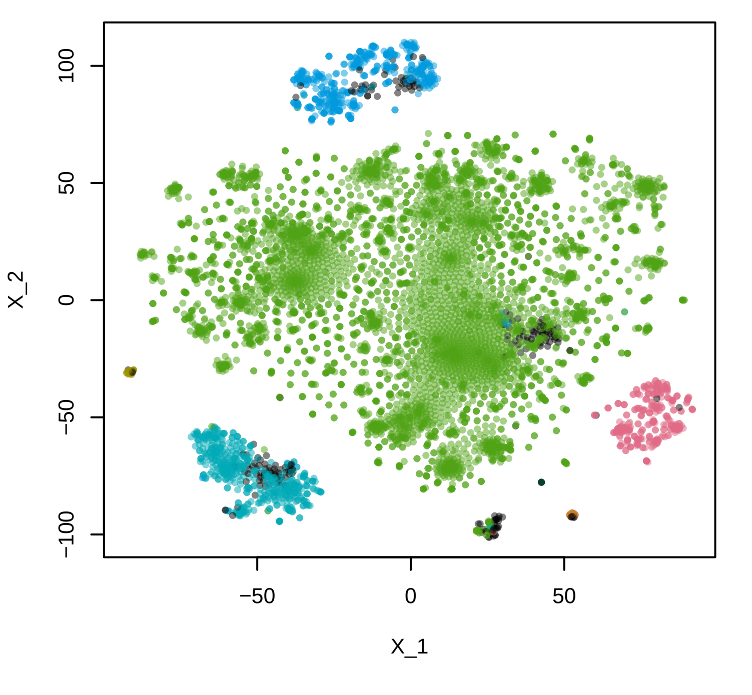

lambda=266.666666666667 d=0.015 r=0.333333333333333 kn

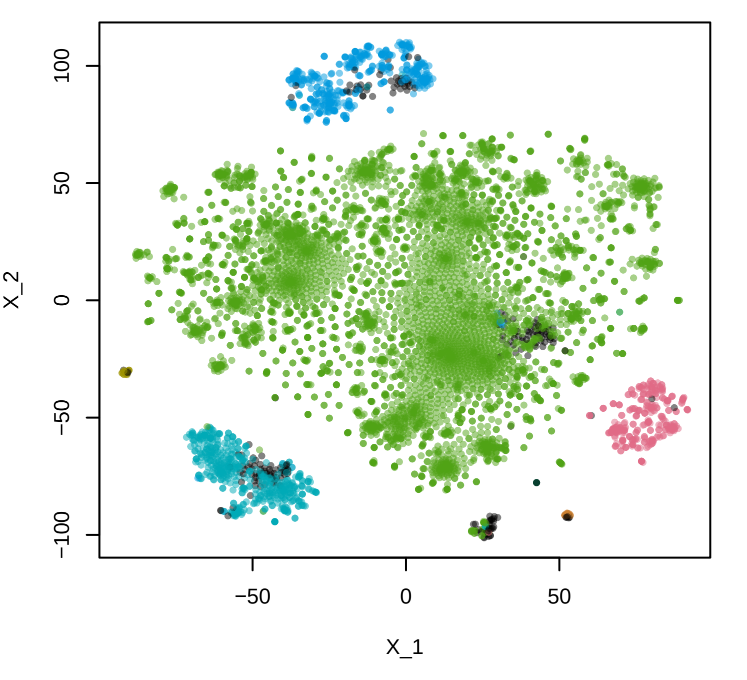

lambda=266.666666666667 d=0.015 r=0.666666666666667 kn

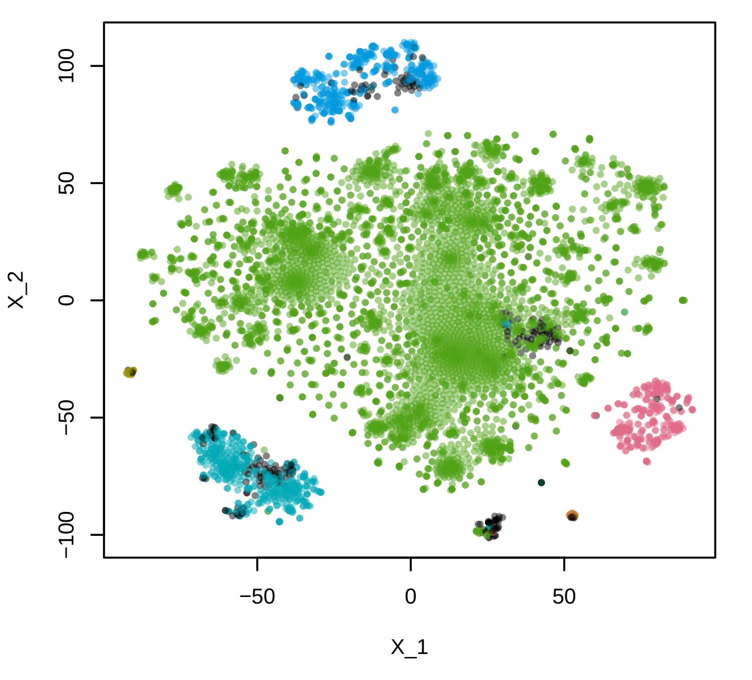

Distribution of IBS values with relative scaling factor for weighting (blue)
lambda=266.666666666667 d=0.0175

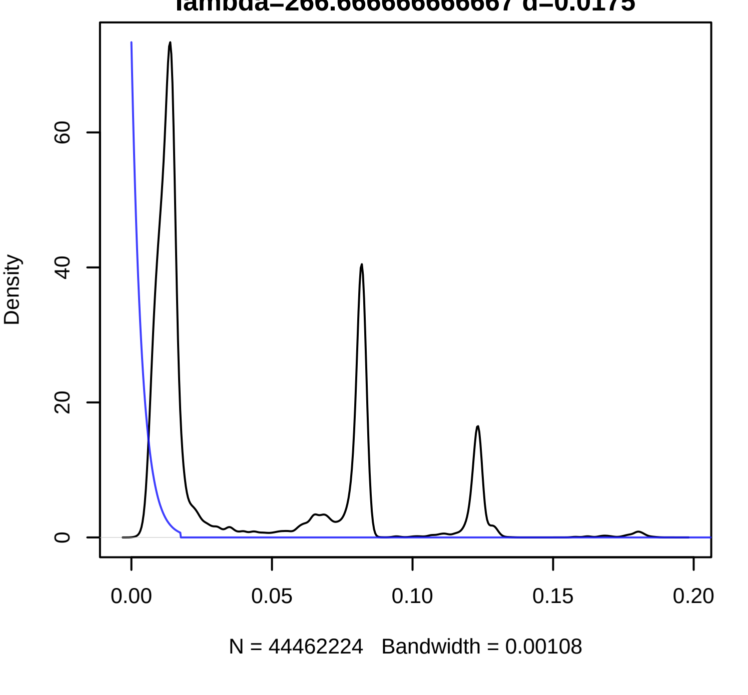

lambda=266.666666666667 d=0.0175 r=0 knn=Inf

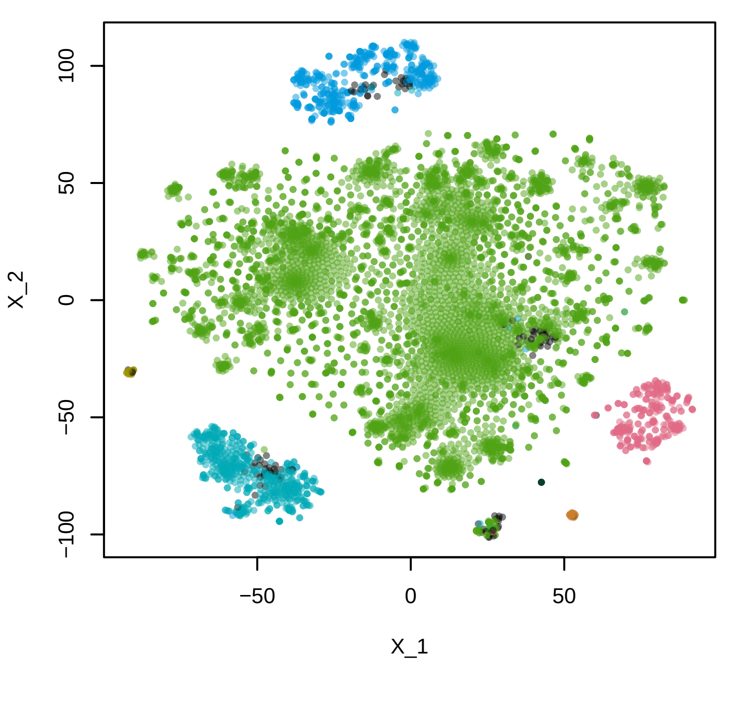

lambda=266.666666666667 d=0.0175 r=0.333333333333333 kn

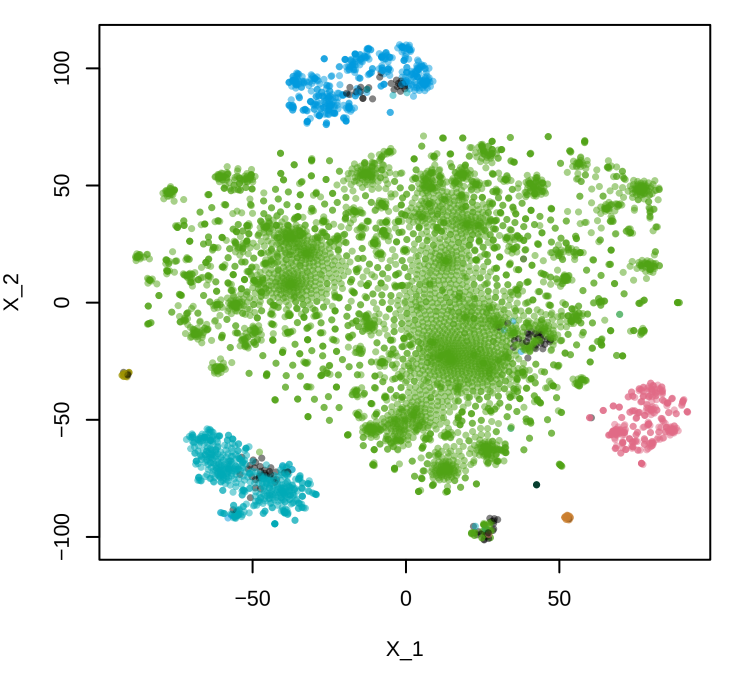

lambda=266.666666666667 d=0.0175 r=0.666666666666667 kn

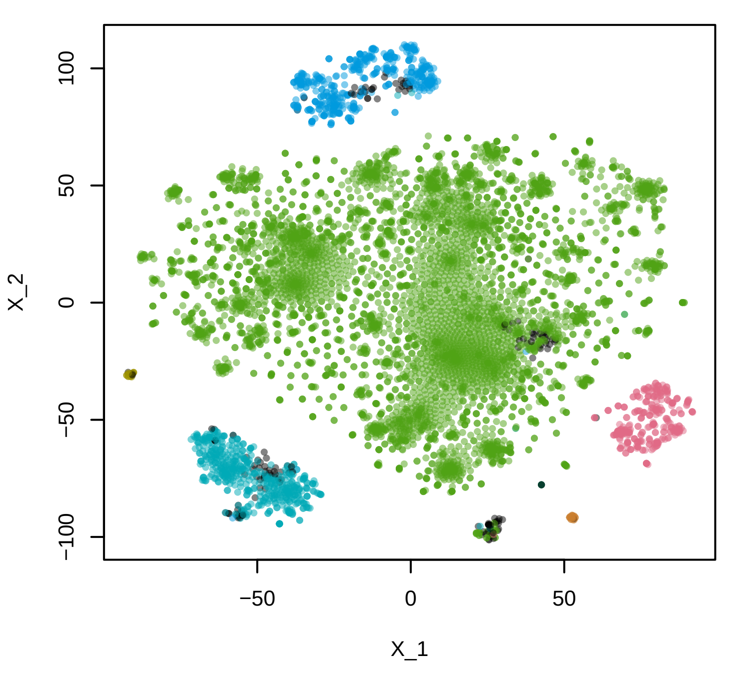

Distribution of IBS values with relative scaling factor for weighting (blue)
lambda=266.666666666667 d=0.02

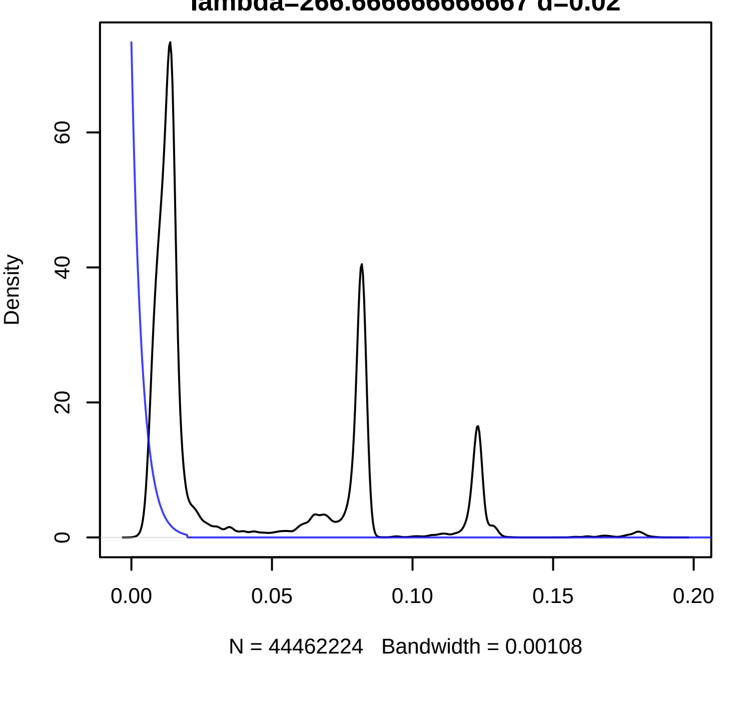

lambda=266.666666666667 d=0.02 r=0 knn=Inf

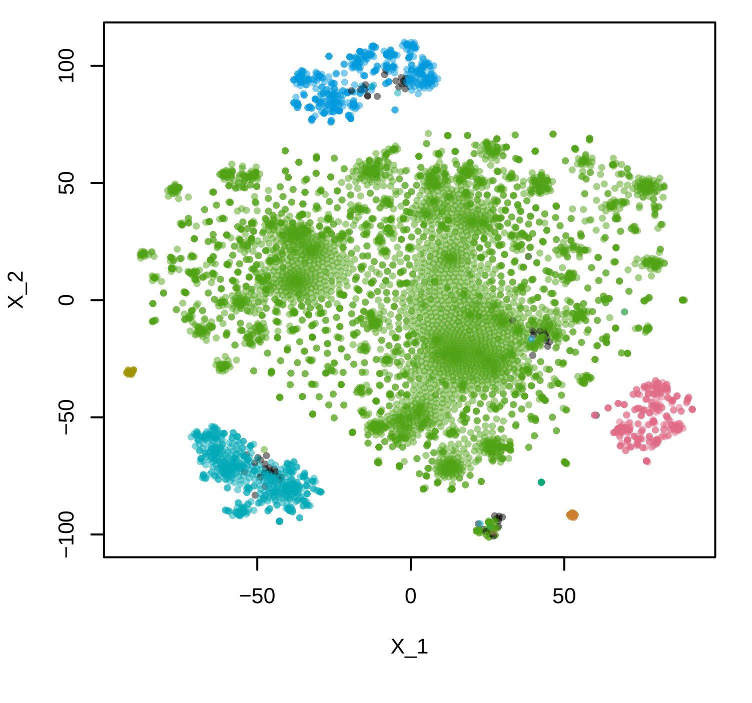

lambda=266.666666666667 d=0.02 r=0.333333333333333 knn

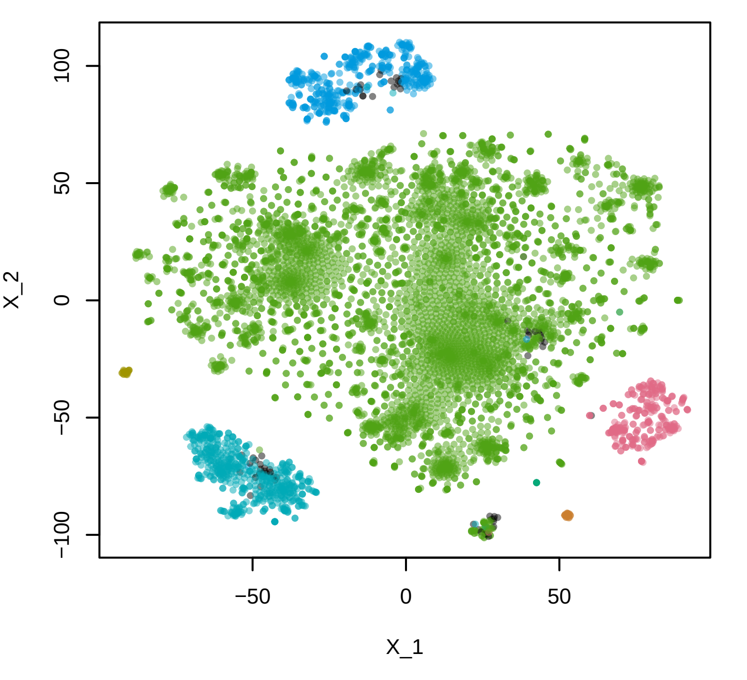

lambda=266.666666666667 d=0.02 r=0.666666666666667 knn

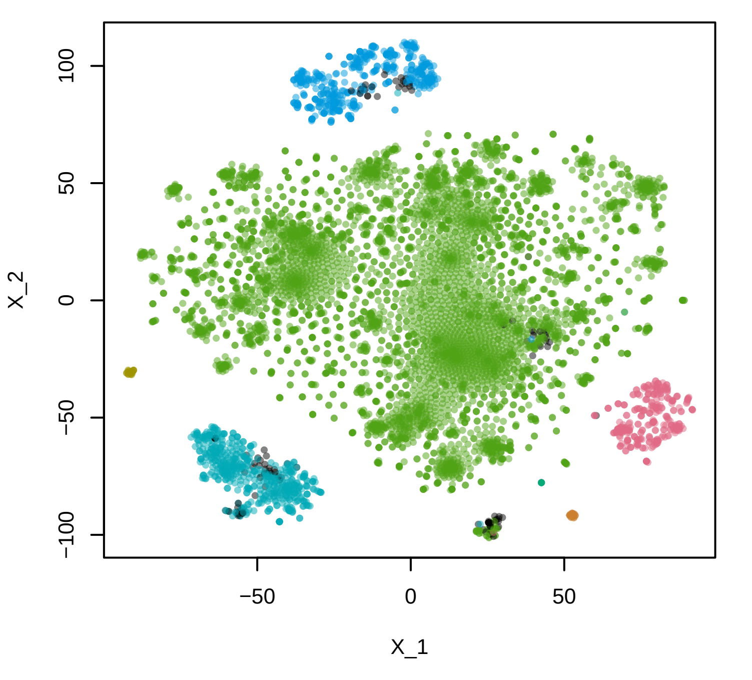

Distribution of IBS values with relative scaling factor for weighting (blue)
lambda=266.666666666667 d=0.0225

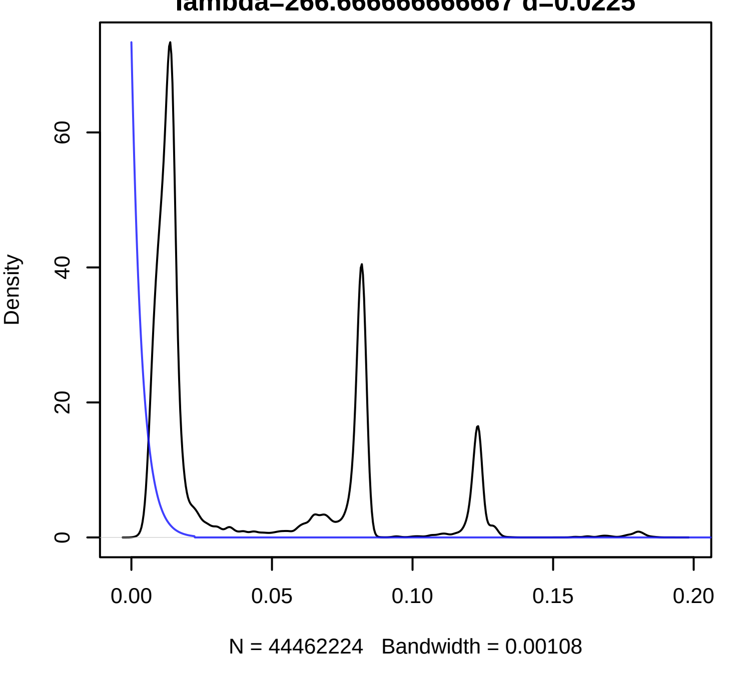

lambda=266.666666666667 d=0.0225 r=0 knn=Inf

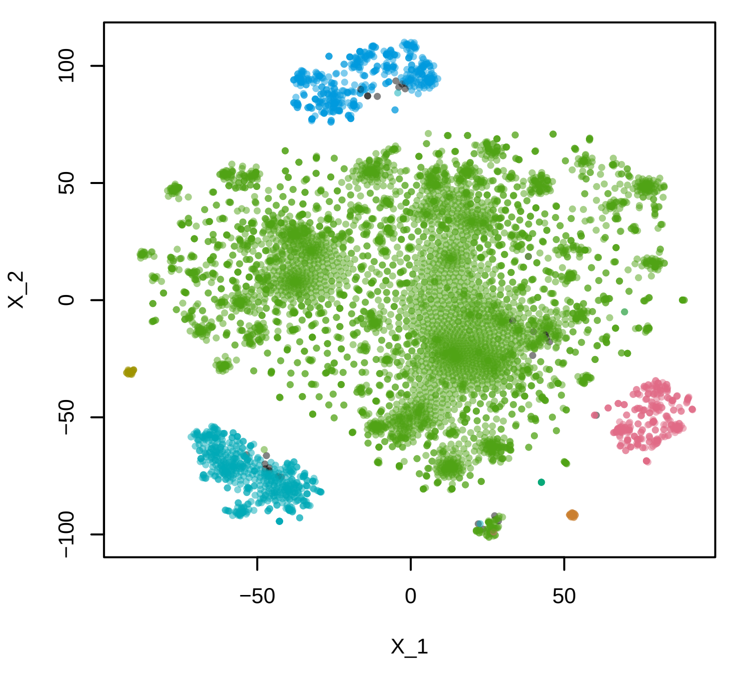

lambda=266.666666666667 d=0.0225 r=0.333333333333333 kn

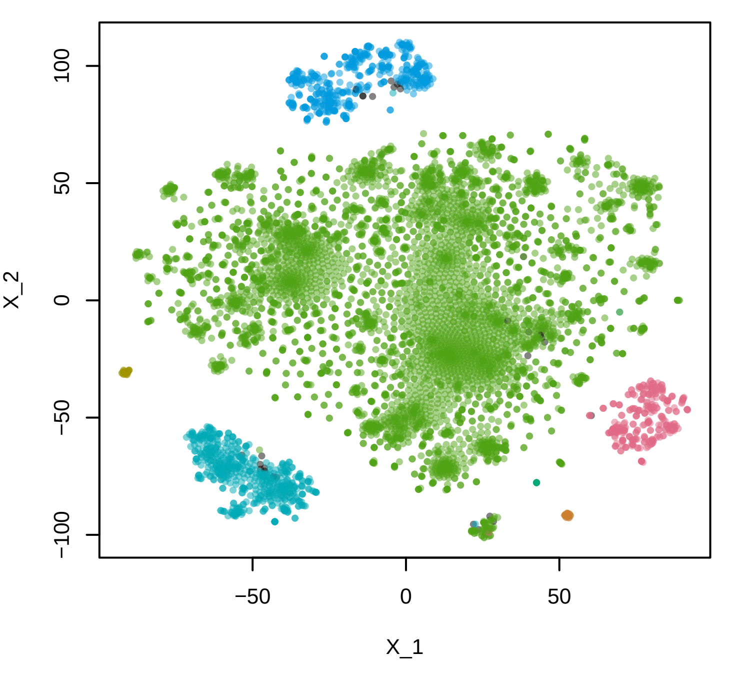

lambda=266.666666666667 d=0.0225 r=0.666666666666667 kn

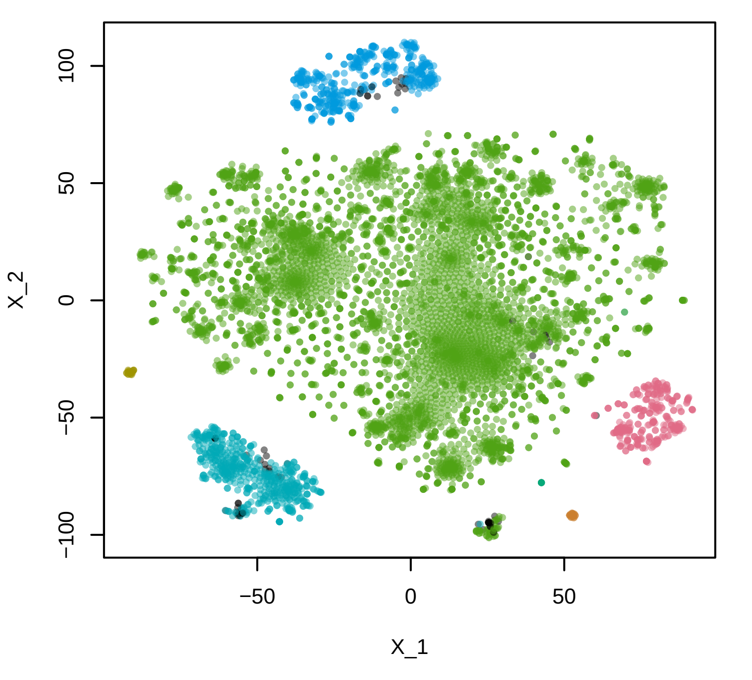

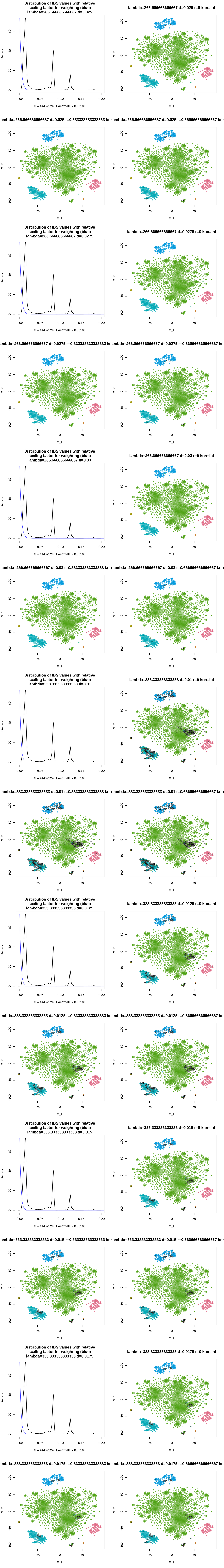
Distribution of IBS values with relative scaling factor for weighting (blue) lambda=266.666666666667 d=0.025
N = 44462224 Bandwidth = 0.00108
lambda=266.666666666667 d=0.025 r=0 knn=Inf
lambda=266.666666666667 d=0.025 r=0.333333333333333 knn
lambda=266.666666666667 d=0.025 r=0.666666666666667 knn
Distribution of IBS values with relative scaling factor for weighting (blue) lambda=266.666666666667 d=0.0275
N = 44462224 Bandwidth = 0.00108
lambda=266.666666666667 d=0.0275 r=0 knn=Inf
lambda=266.666666666667 d=0.0275 r=0.333333333333333
lambda=266.666666666667 d=0.0275 r=0.666666666666667
Distribution of IBS values with relative scaling factor for weighting (blue) lambda=266.666666666667 d=0.03
N = 44462224 Bandwidth = 0.00108
lambda=266.666666666667 d=0.03 r=0 knn=Inf
lambda=266.666666666667 d=0.03 r=0.333333333333333 knn
lambda=266.666666666667 d=0.03 r=0.666666666666667
Distribution of IBS values with relative scaling factor for weighting (blue) lambda=333.333333333333 d=0.01
N = 44462224 Bandwidth = 0.00108
lambda=333.333333333333 d=0.01 r=0 knn=Inf
lambda=333.333333333333 d=0.01 r=0.333333333333333 knn
lambda=333.333333333333 d=0.01 r=0.666666666666667
Distribution of IBS values with relative scaling factor for weighting (blue) lambda=333.333333333333 d=0.0125
N = 44462224 Bandwidth = 0.00108
lambda=333.333333333333 d=0.0125 r=0 knn=Inf
lambda=333.333333333333 d=0.0125 r=0.333333333333333 kn
lambda=333.333333333333 d=0.0125 r=0.666666666666667
Distribution of IBS values with relative scaling factor for weighting (blue) lambda=333.333333333333 d=0.015
N = 44462224 Bandwidth = 0.00108
lambda=333.333333333333 d=0.015 r=0 knn=Inf
lambda=333.333333333333 d=0.015 r=0.333333333333333 kn
lambda=333.333333333333 d=0.015 r=0.666666666666667
Distribution of IBS values with relative scaling factor for weighting (blue) lambda=333.333333333333 d=0.0175
N = 44462224 Bandwidth = 0.00108
lambda=333.333333333333 d=0.0175 r=0 knn=Inf
lambda=333.333333333333 d=0.0175 r=0.333333333333333 kn
lambda=333.333333333333 d=0.0175 r=0.666666666666667
Density
X_1
X_2

Distribution of IBS values with relative scaling factor for weighting (blue) lambda=333.333333333333 d=0.02

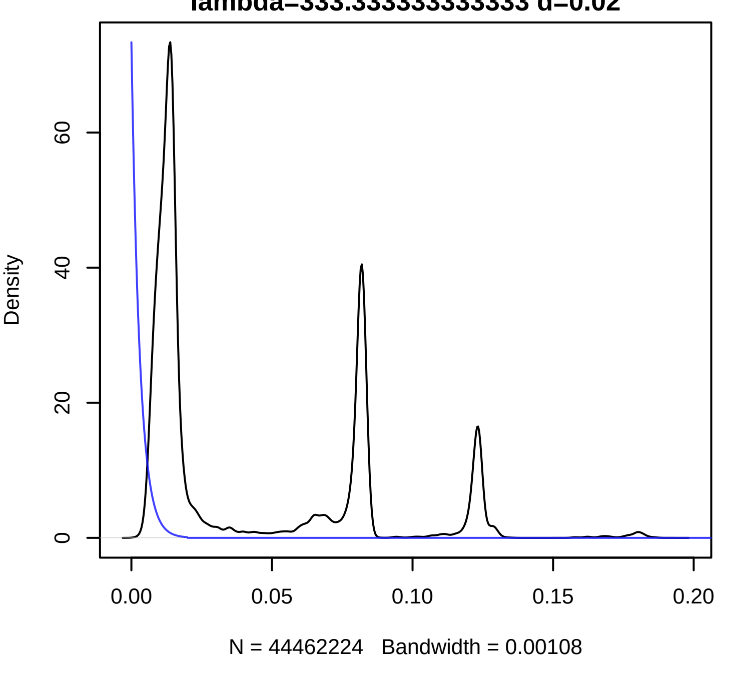

lambda=333.333333333333 d=0.02 r=0 knn=Inf

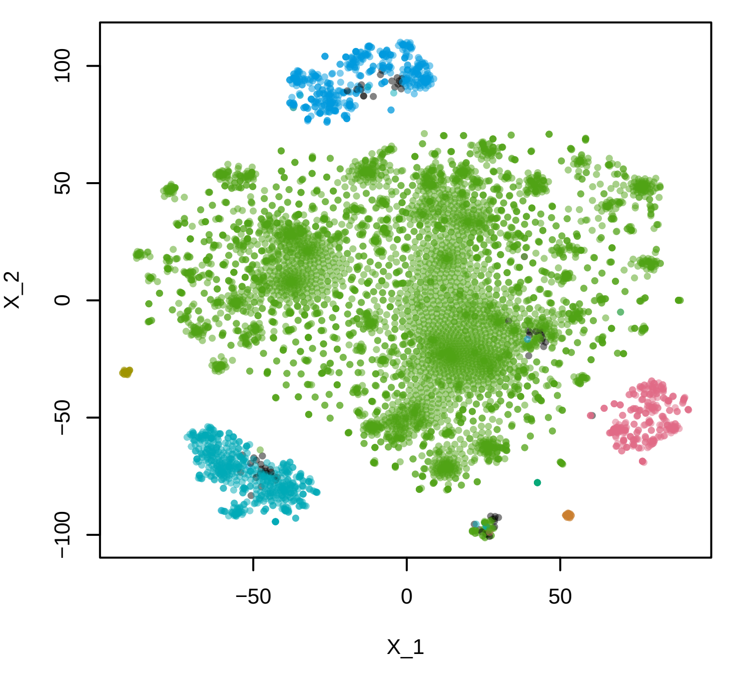

ambda=333.333333333333 d=0.02 r=0.333333333333333 knn

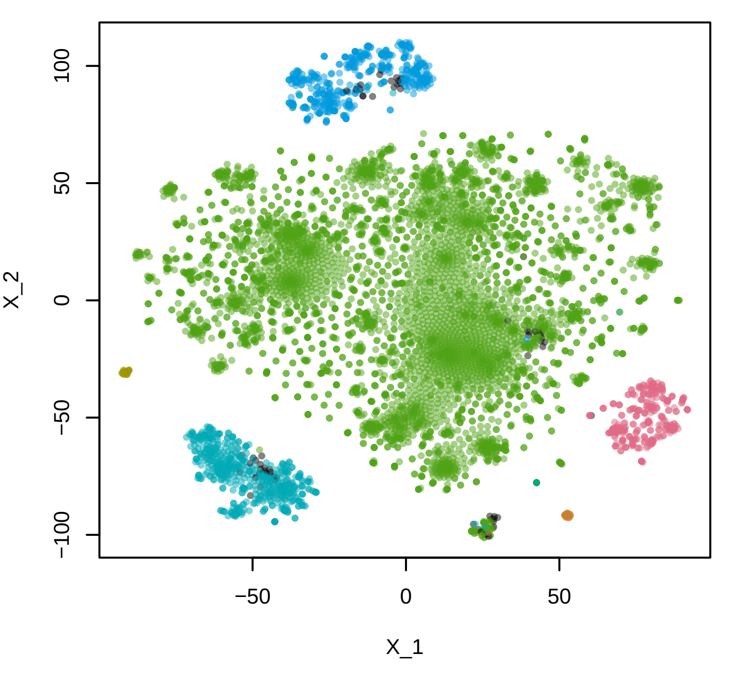

lambda=333.333333333333 d=0.02 r=0.666666666666667 knn=Inf

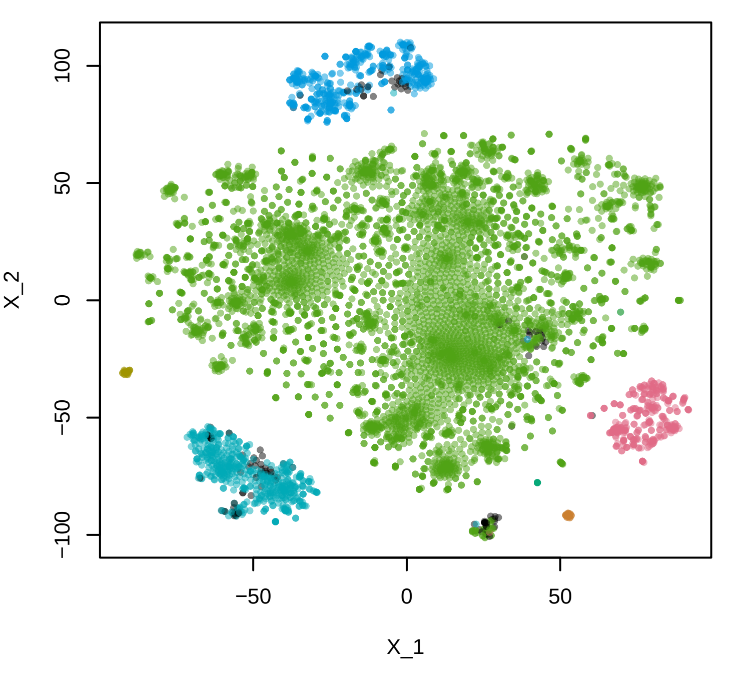

Distribution of IBS values with relative scaling factor for weighting (blue) lambda=333.333333333333 d=0.0225

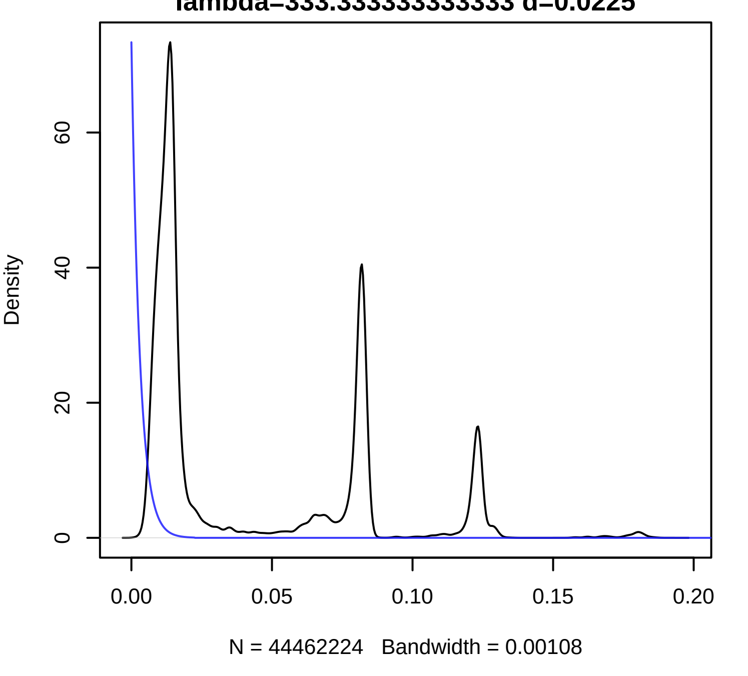

lambda=333.333333333333 d=0.0225 r=0 knn=Inf

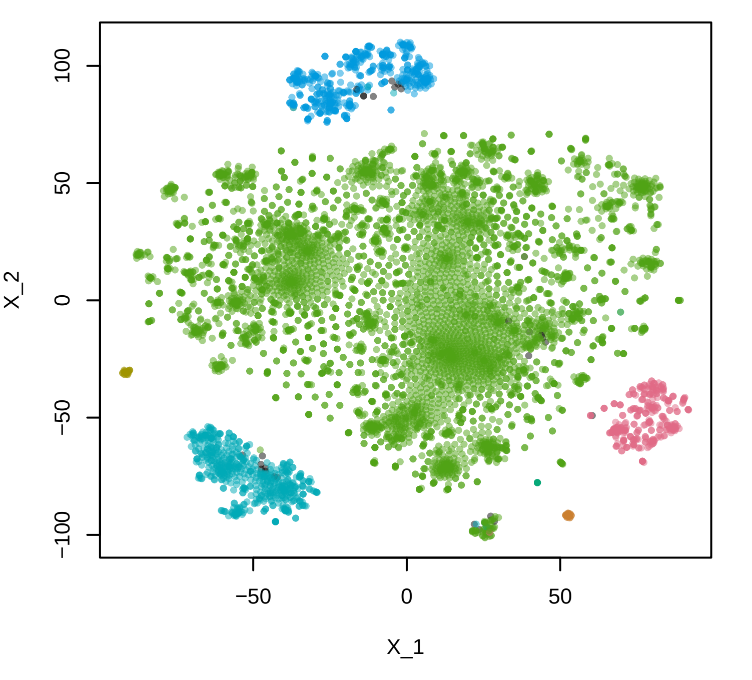

ambda=333.333333333333 d=0.0225 r=0.333333333333333 kn

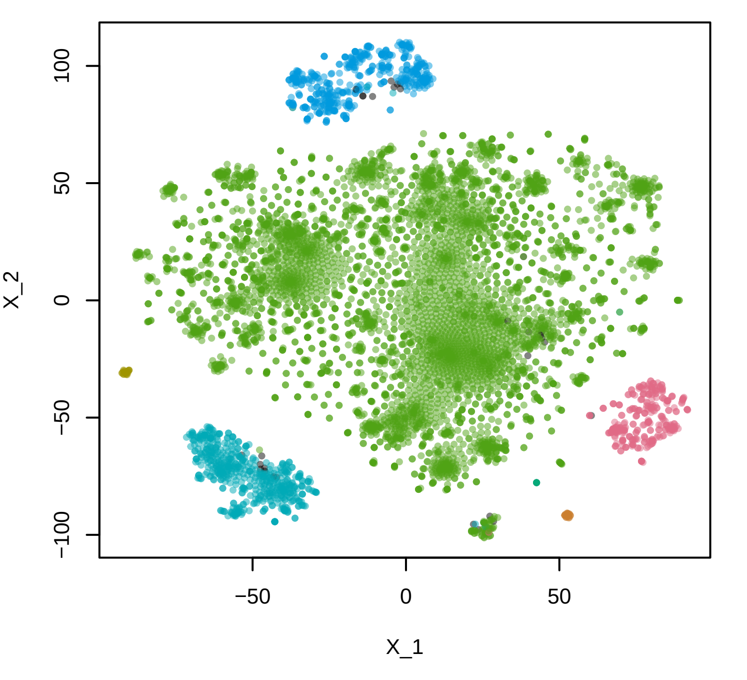

ambda=333.333333333333 d=0.0225 r=0.666666666666667 kn

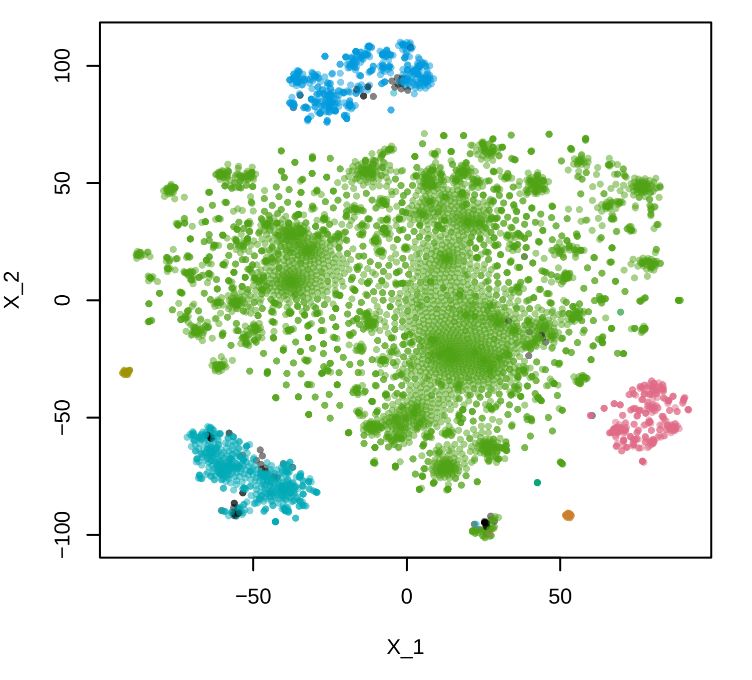

Distribution of IBS values with relative scaling factor for weighting (blue) lambda=333.333333333333 d=0.025

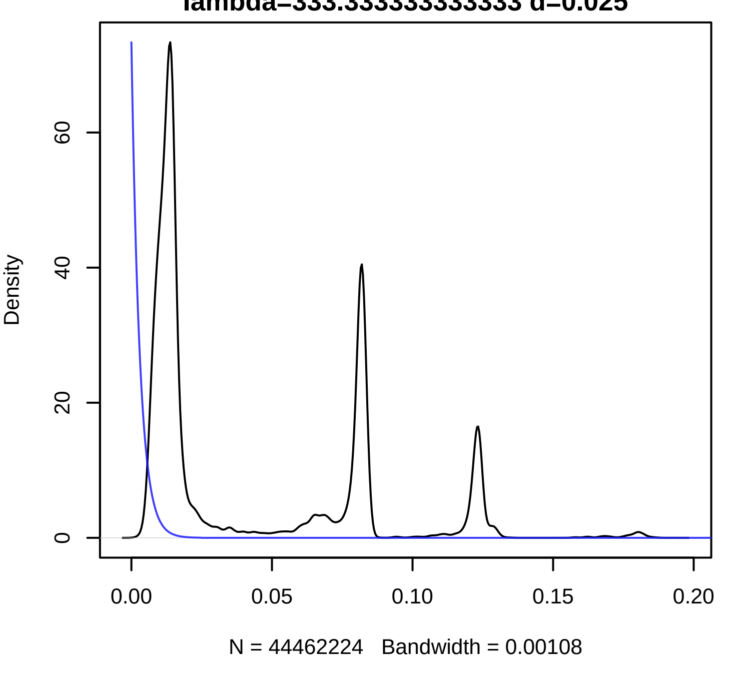

lambda=333.333333333333 d=0.025 r=0 knn=Inf

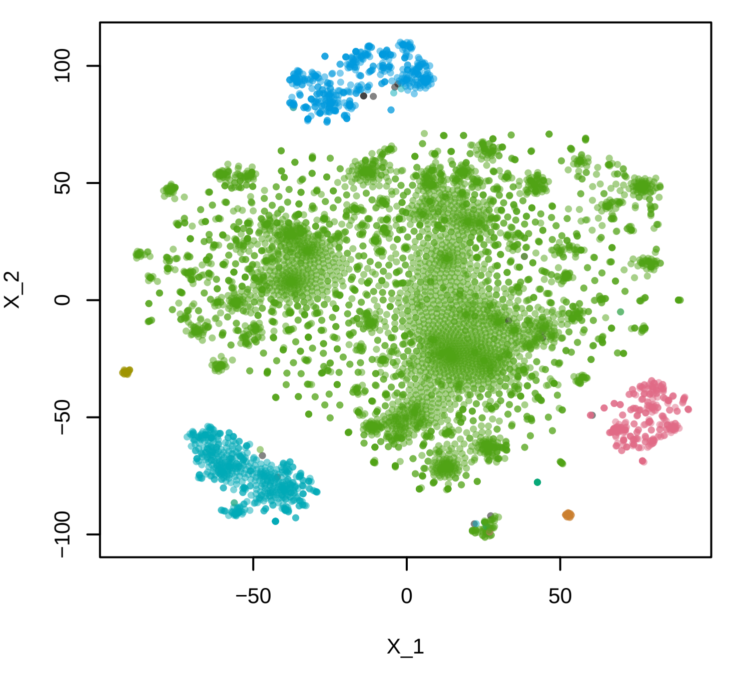

ambda=333.333333333333 d=0.025 r=0.333333333333333 kn

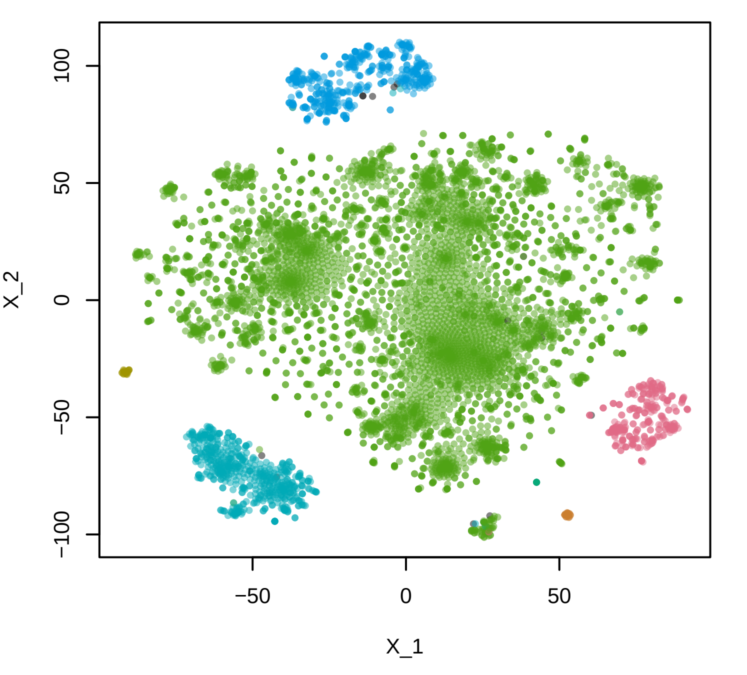

ambda=333.333333333333 d=0.025 r=0.666666666666667 kn

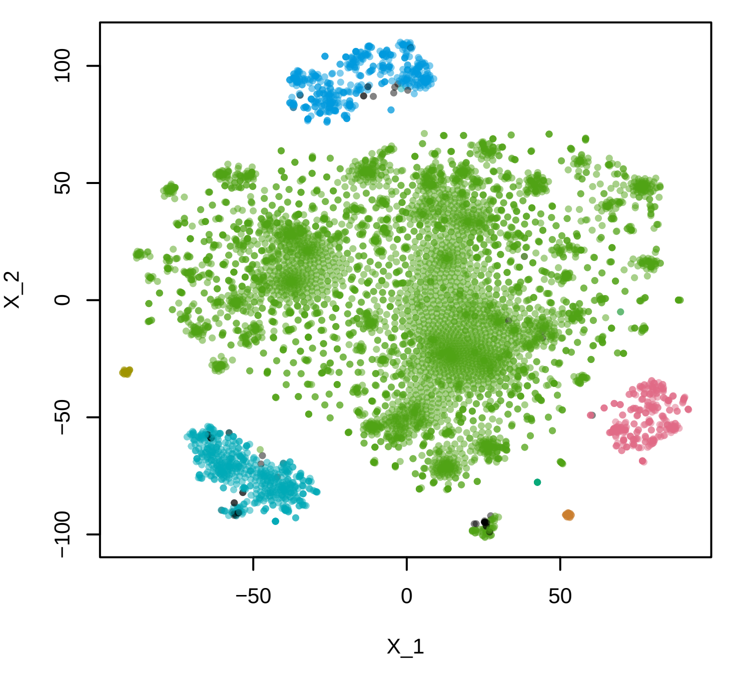

Distribution of IBS values with relative scaling factor for weighting (blue) lambda=333.333333333333 d=0.0275

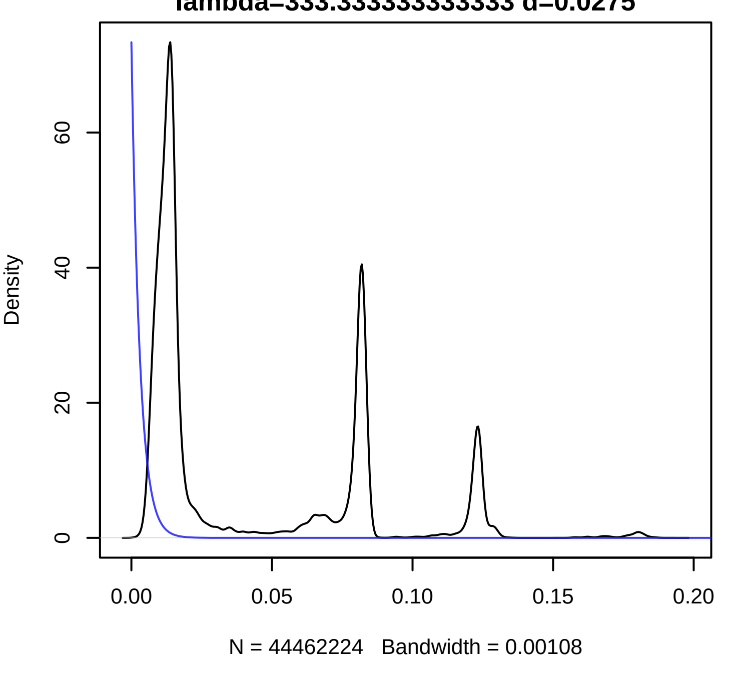

lambda=333.333333333333 d=0.0275 r=0 knn=Inf

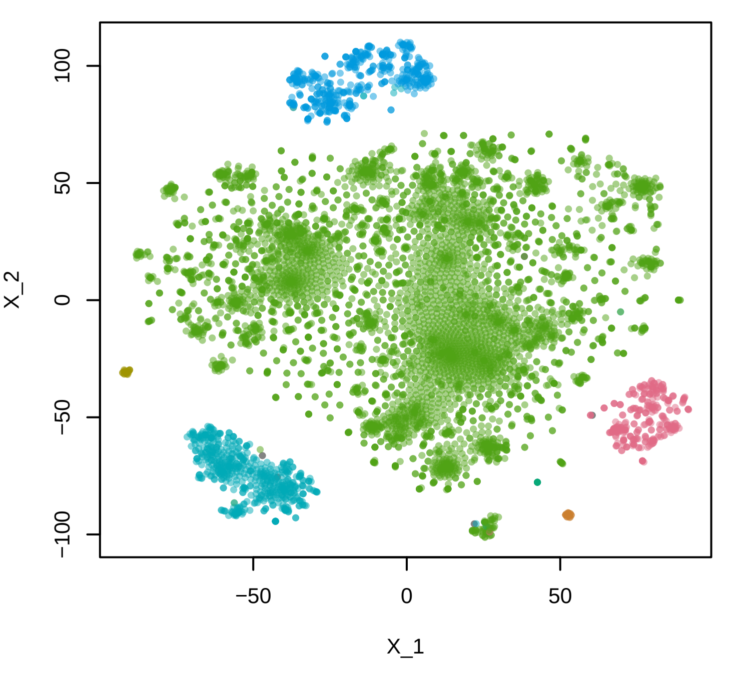

ambda=333.333333333333 d=0.0275 r=0.333333333333333 kn

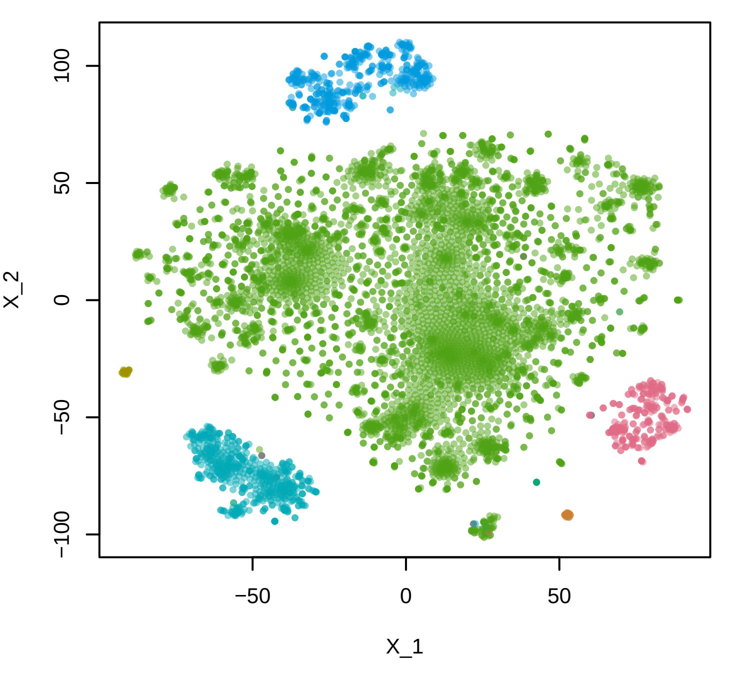

ambda=333.333333333333 d=0.0275 r=0.666666666666667 kn

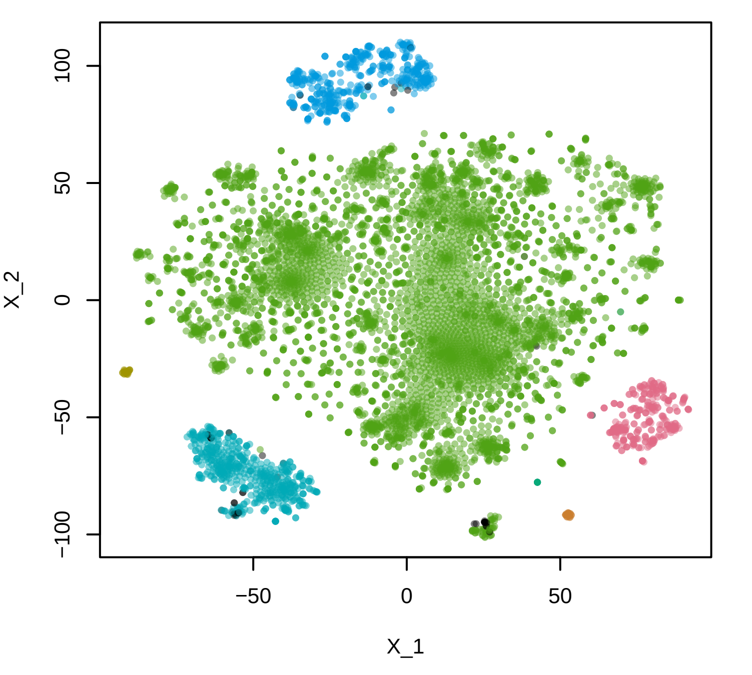

Distribution of IBS values with relative scaling factor for weighting (blue) lambda=333.333333333333 d=0.03

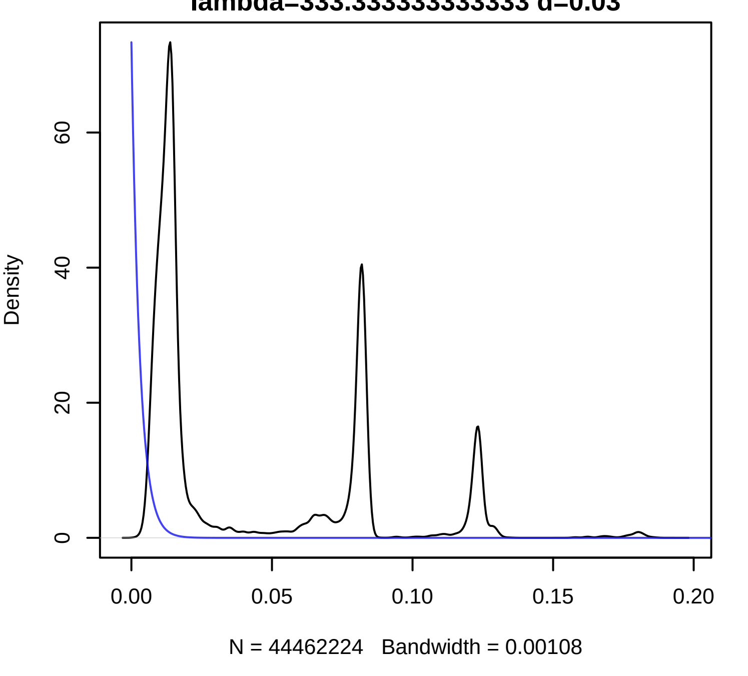

lambda=333.333333333333 d=0.03 r=0 knn=Inf

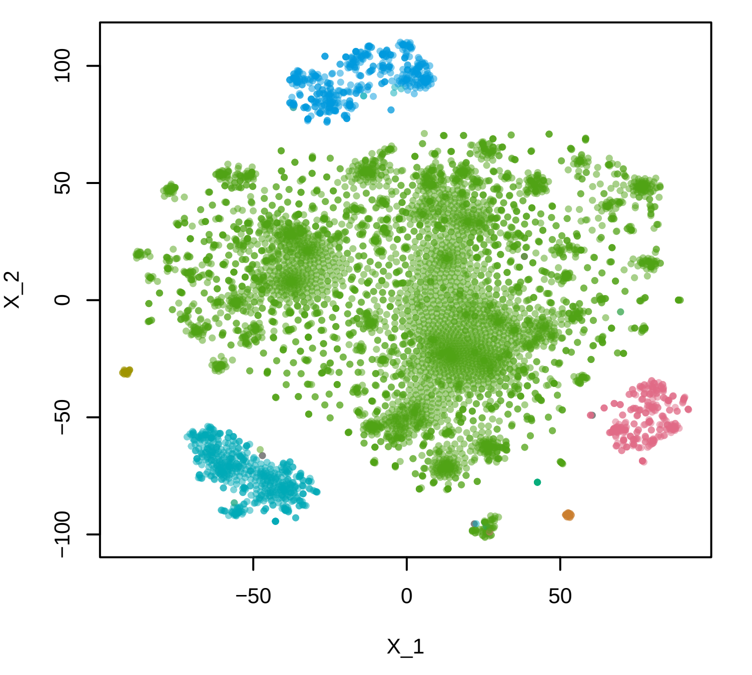

ambda=333.333333333333 d=0.03 r=0.333333333333333 knn

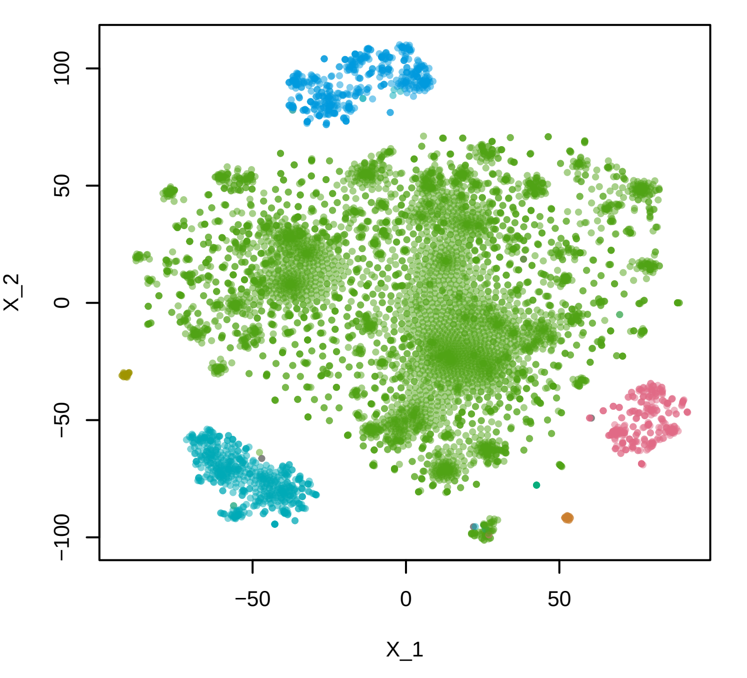

lambda=333.333333333333 d=0.03 r=0.666666666666667 knn

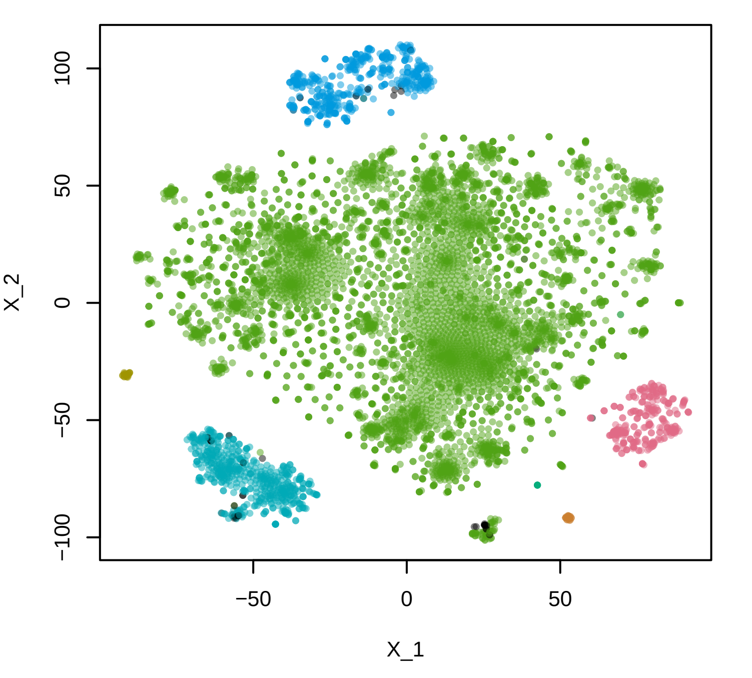

Distribution of IBS values with relative scaling factor for weighting (blue) lambda=400 d=0.01

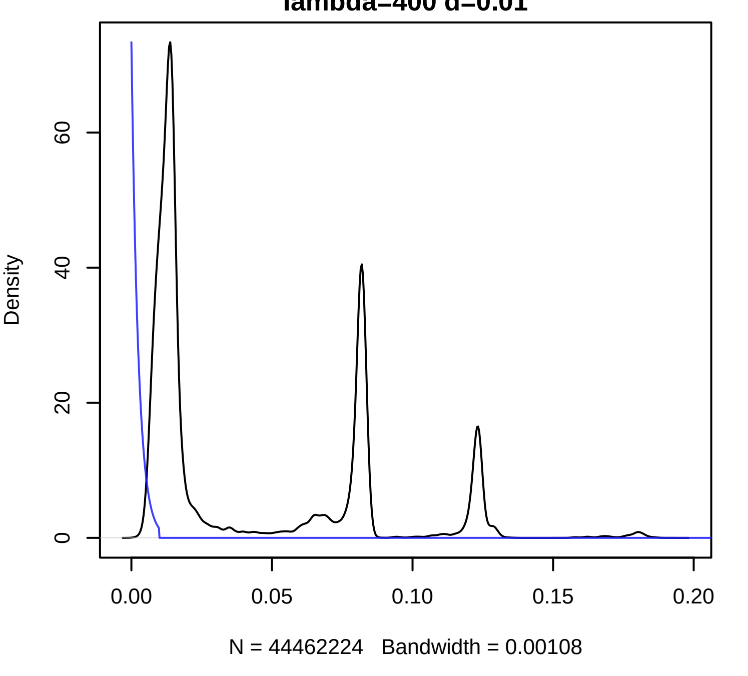

lambda=400 d=0.01 r=0 knn=Inf

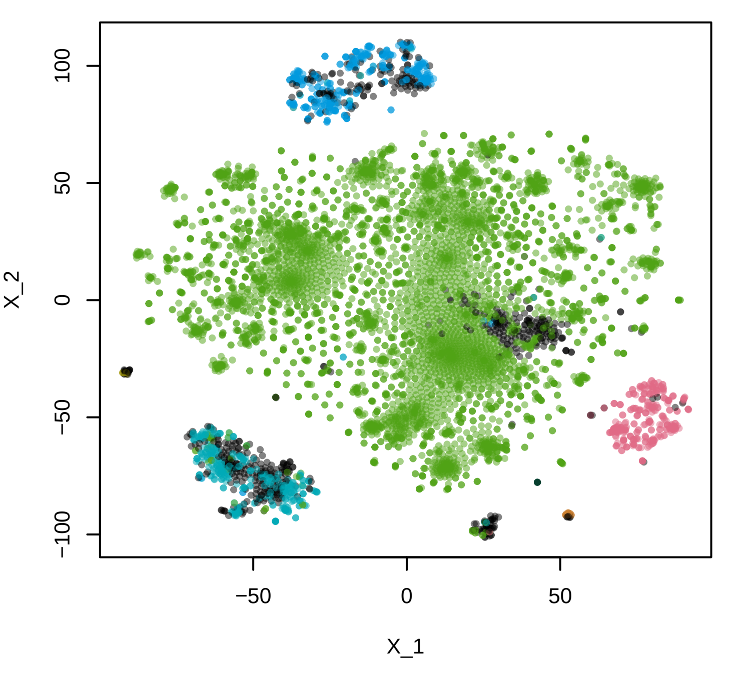

lambda=400 d=0.01 r=0.333333333333333 knn=Inf

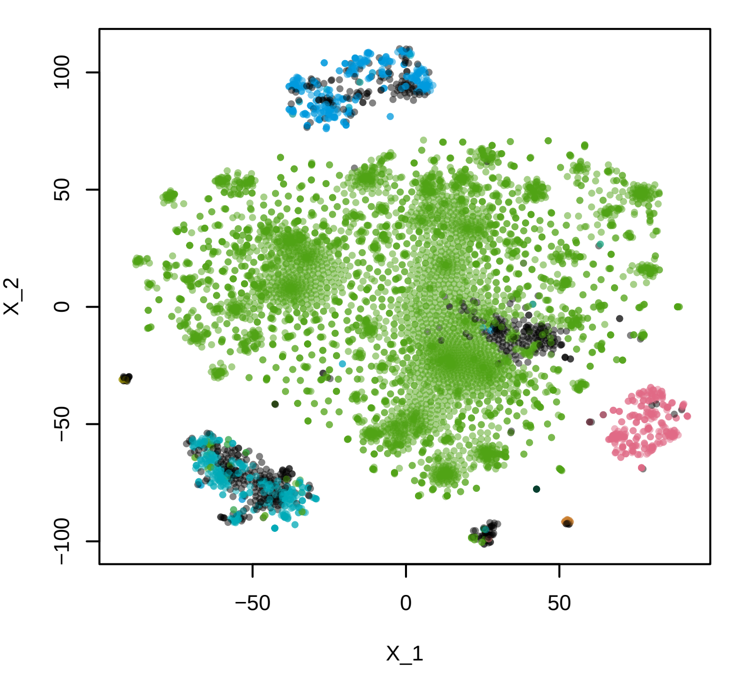

lambda=400 d=0.01 r=0.666666666666667 knn=Inf

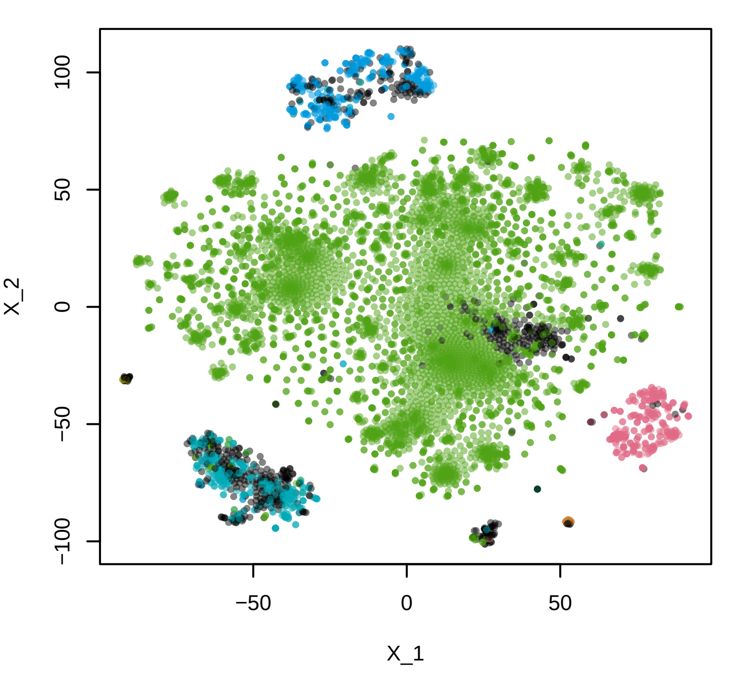

Distribution of IBS values with relative scaling factor for weighting (blue) lambda=400 d=0.0125

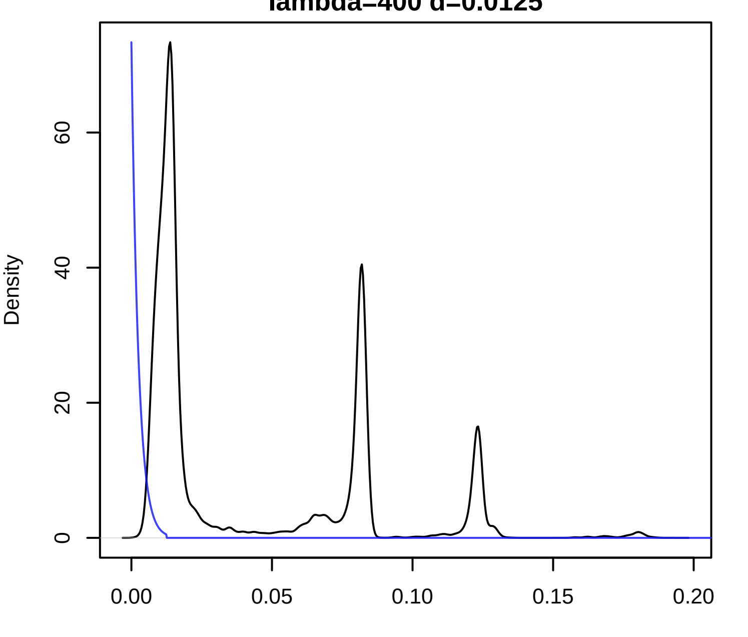

lambda=400 d=0.0125 r=0 knn=Inf

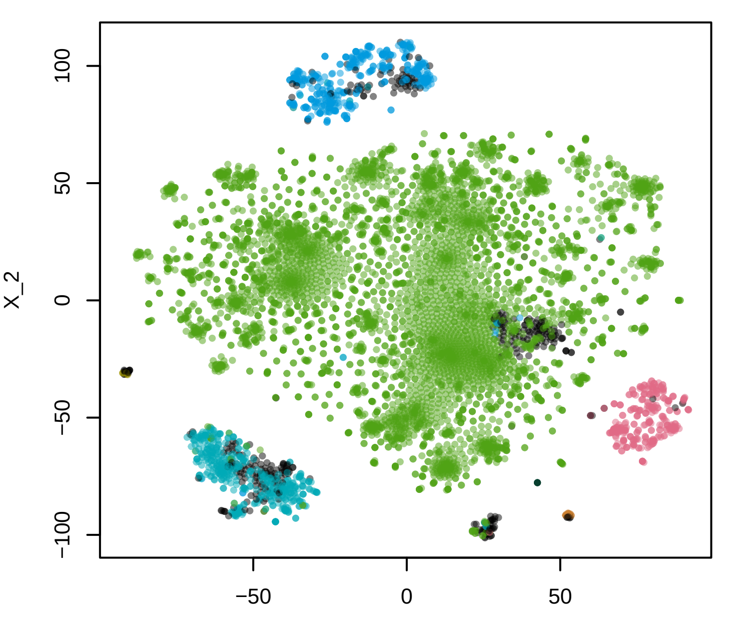

lambda=400 d=0.0125 r=0.333333333333333 knn=Inf

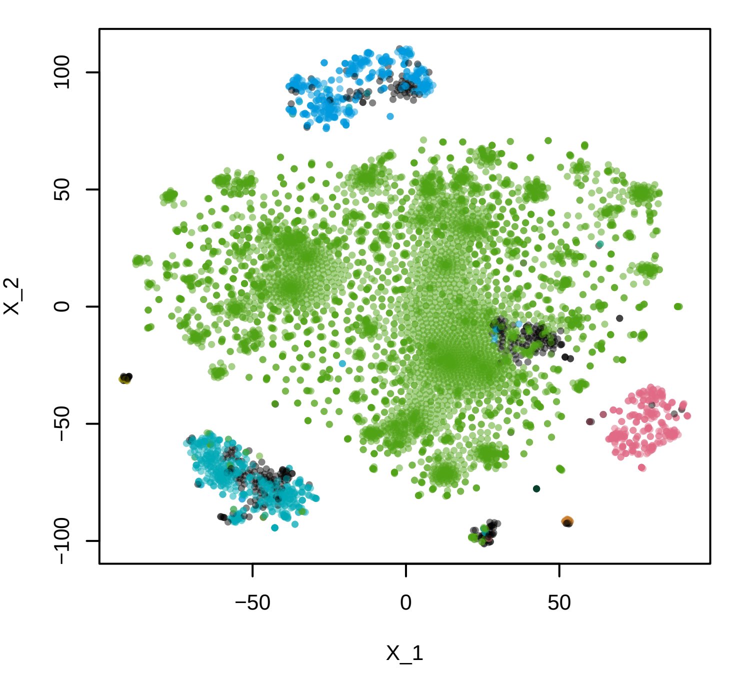

lambda=400 d=0.0125 r=0.666666666666667 knn=Inf

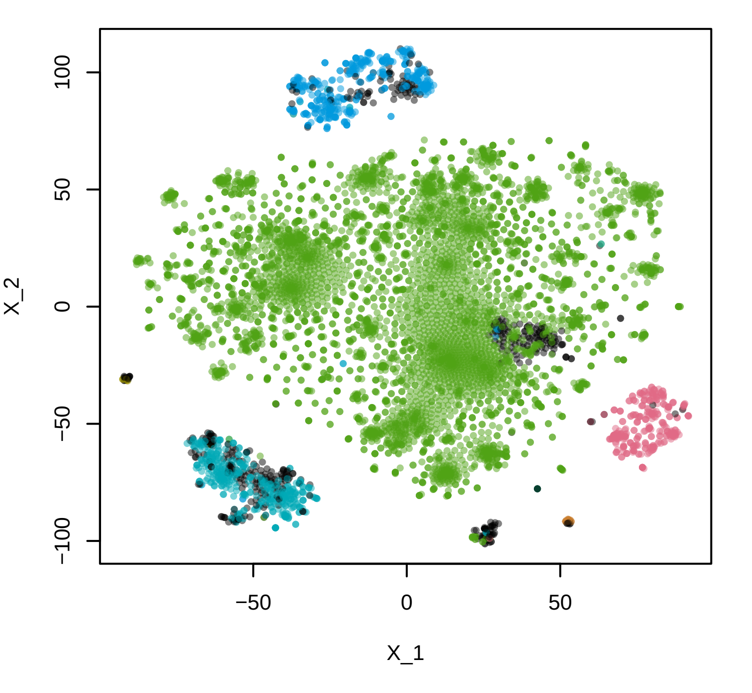

Distribution of IBS values with relative scaling factor for weighting (blue) lambda=400 d=0.015

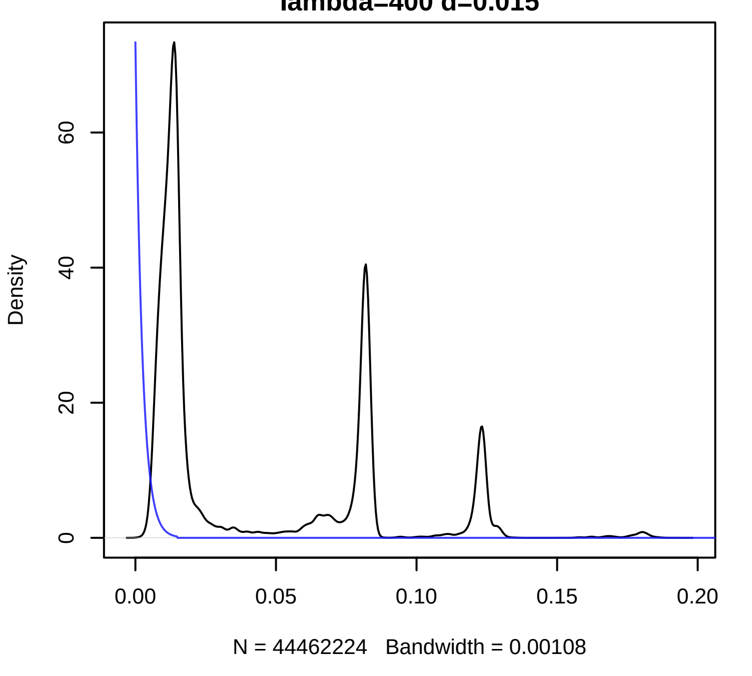

lambda=400 d=0.015 r=0 knn=Inf

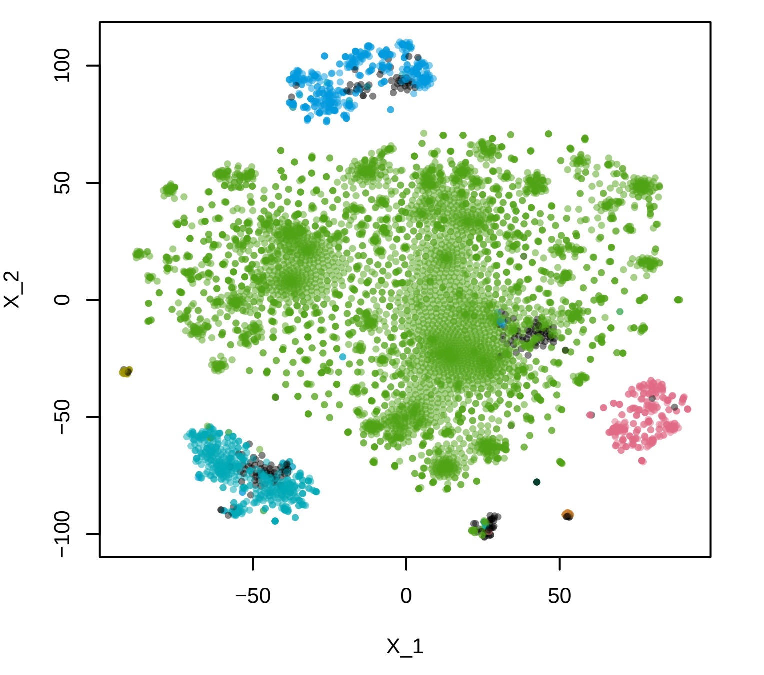

lambda=400 d=0.015 r=0.333333333333333 knn=Inf

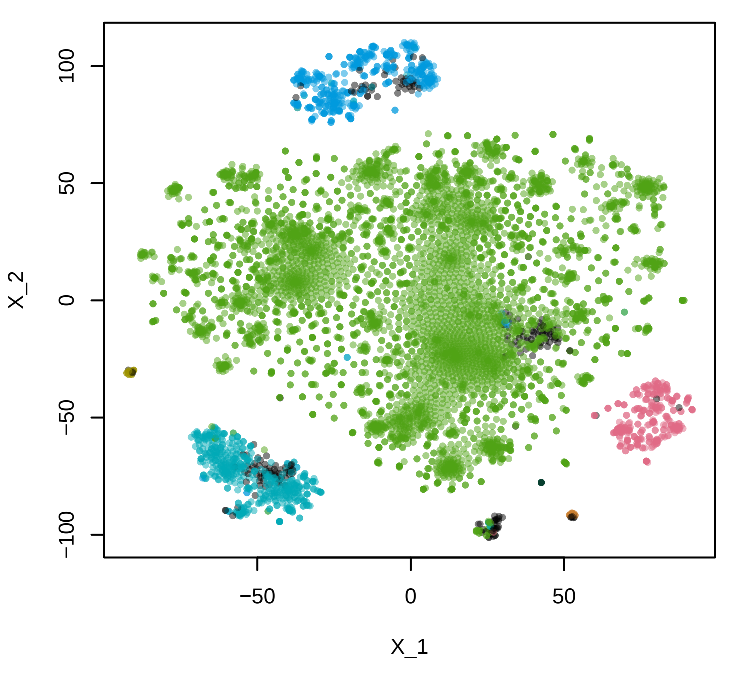

lambda=400 d=0.015 r=0.666666666666667 knn=Inf

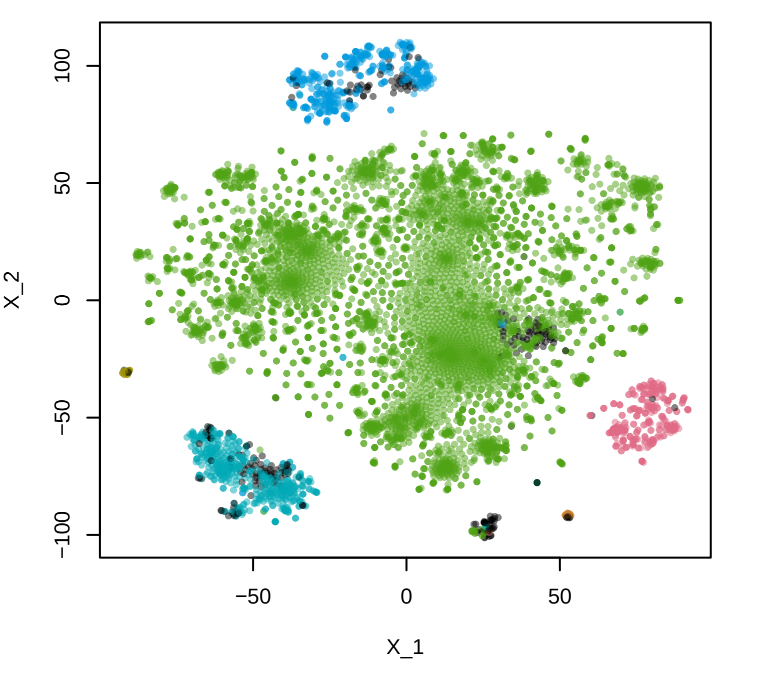

Distribution of IBS values with relative scaling factor for weighting (blue) lambda=400 d=0.0175

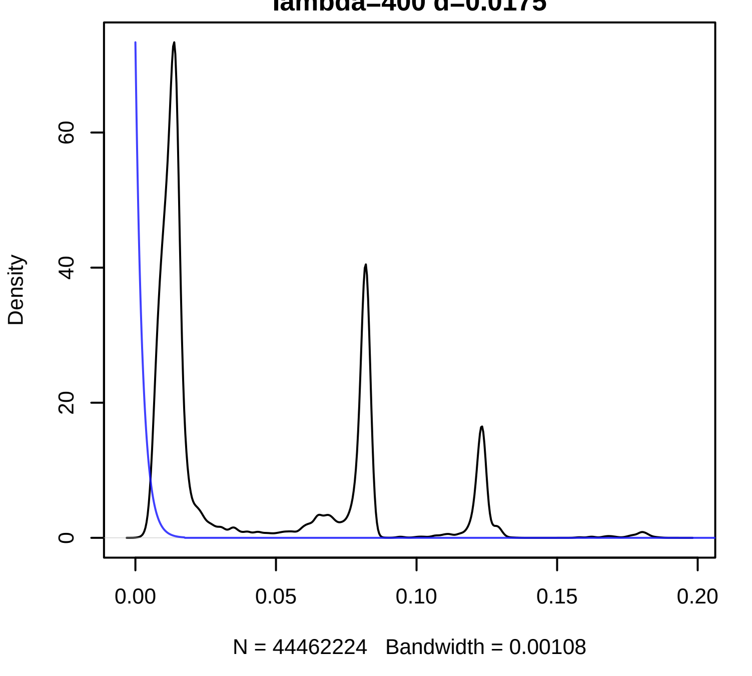

lambda=400 d=0.0175 r=0 knn=Inf

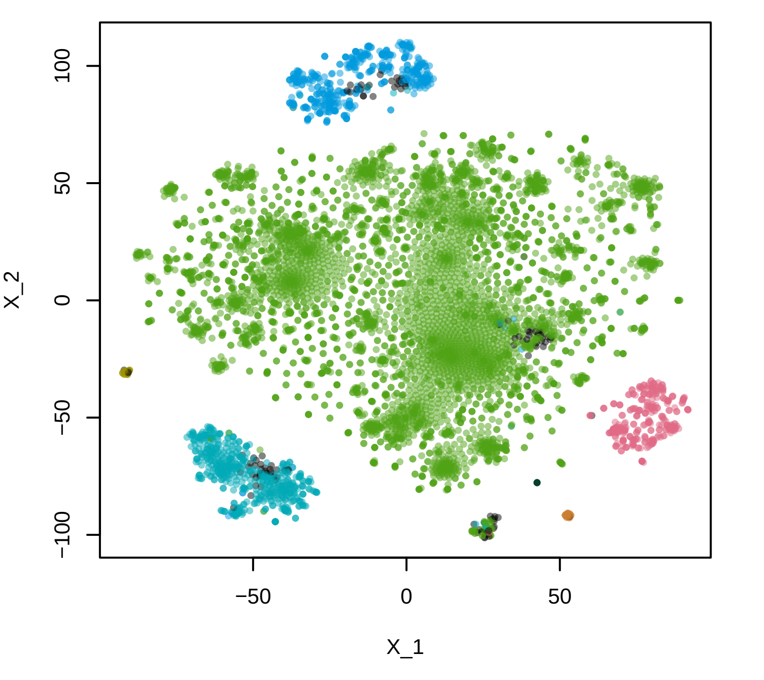

lambda=400 d=0.0175 r=0.333333333333333 knn=Inf

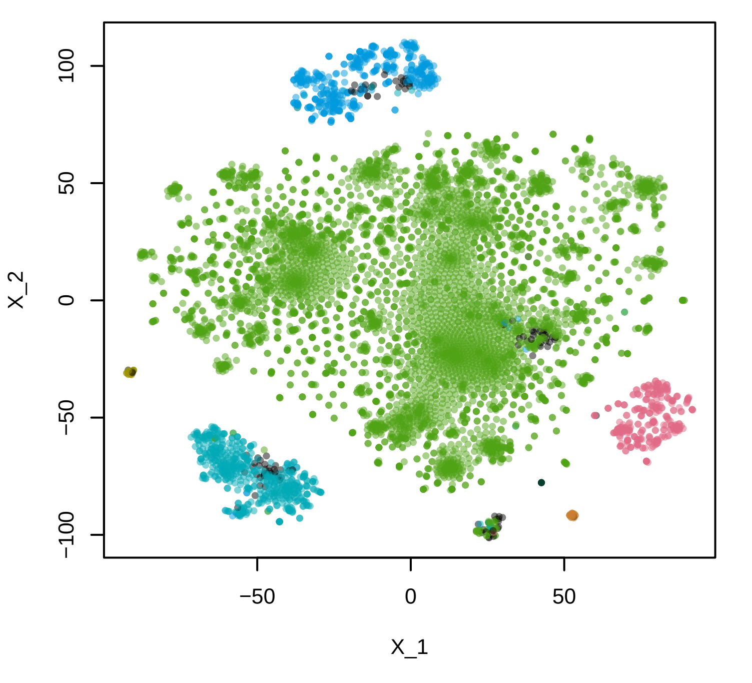

lambda=400 d=0.0175 r=0.666666666666667 knn=Inf

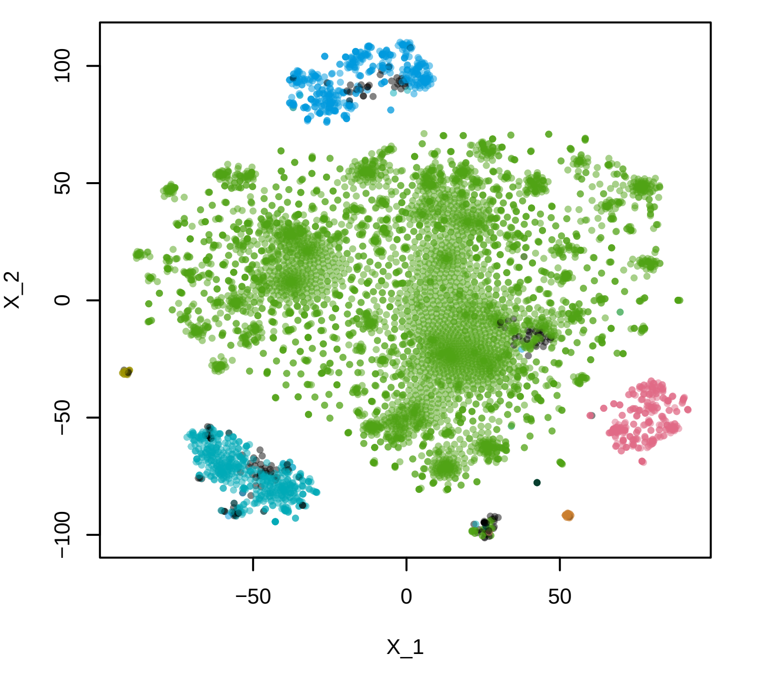

Distribution of IBS values with relative scaling factor for weighting (blue) lambda=400 d=0.02

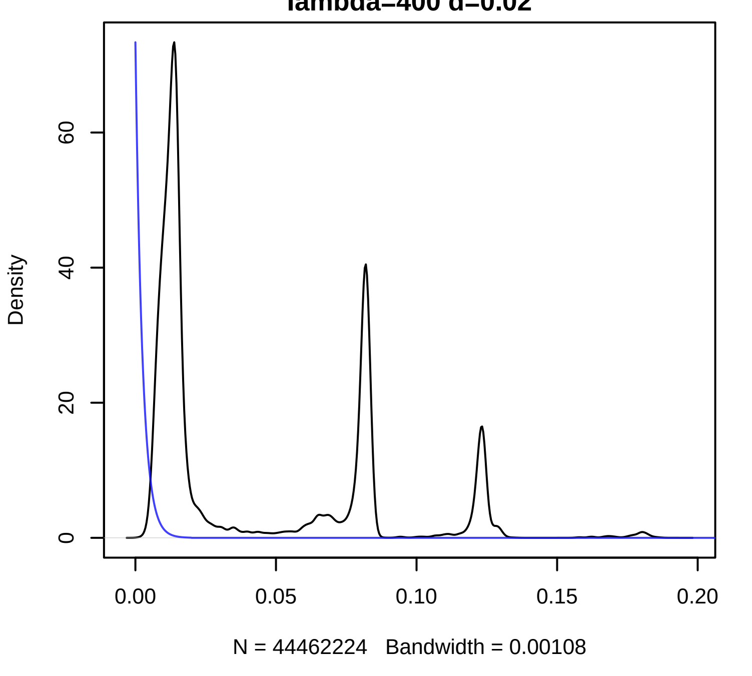

lambda=400 d=0.02 r=0 knn=Inf

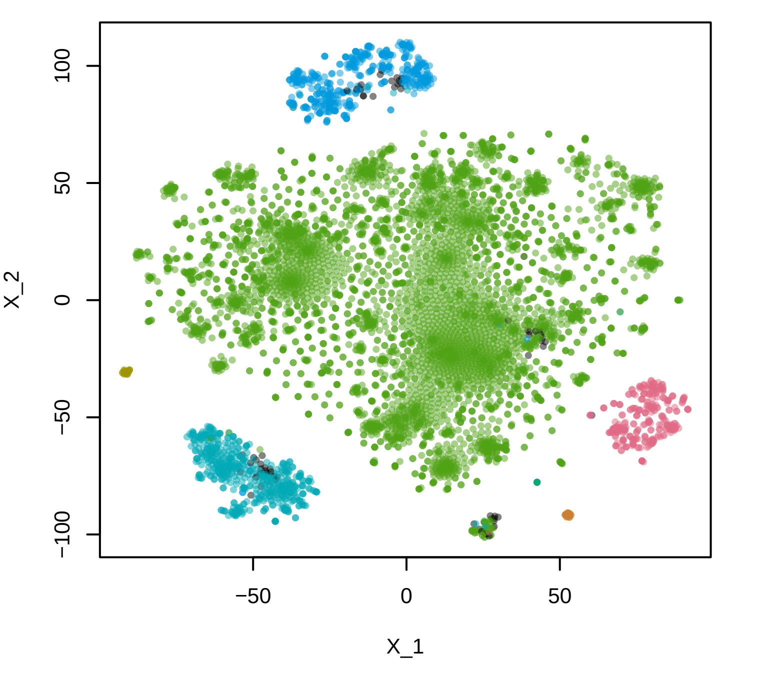

lambda=400 d=0.02 r=0.333333333333333 knn=Inf

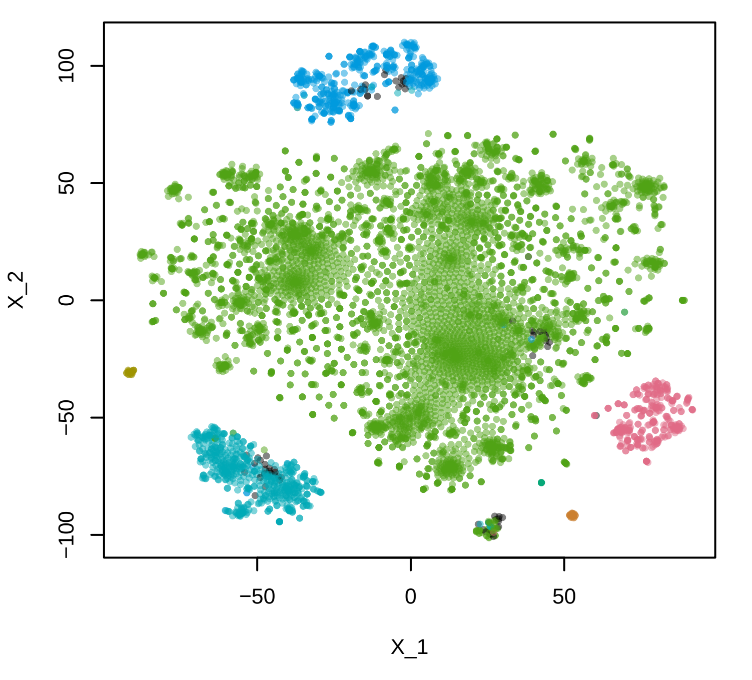

lambda=400 d=0.02 r=0.666666666666667 knn=Inf

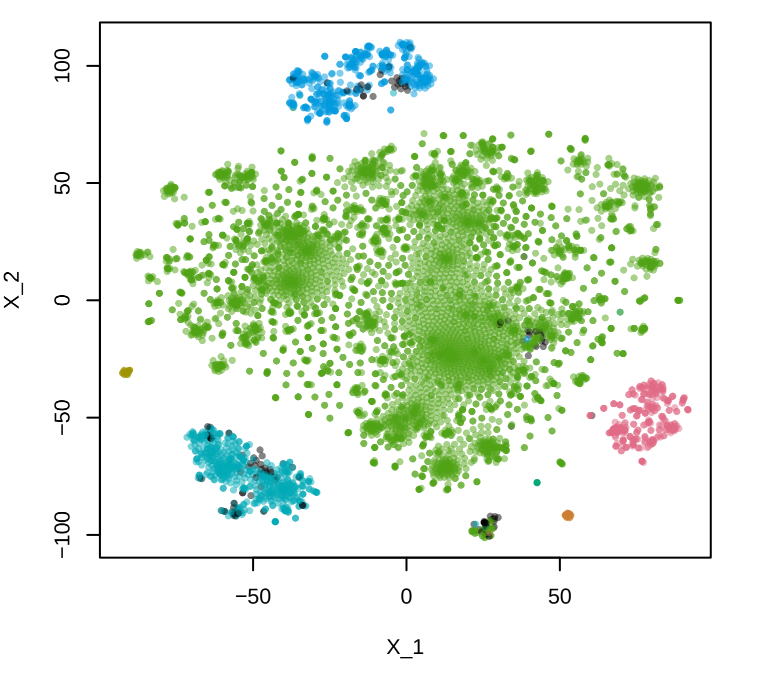

Distribution of IBS values with relative scaling factor for weighting (blue) lambda=400 d=0.0225

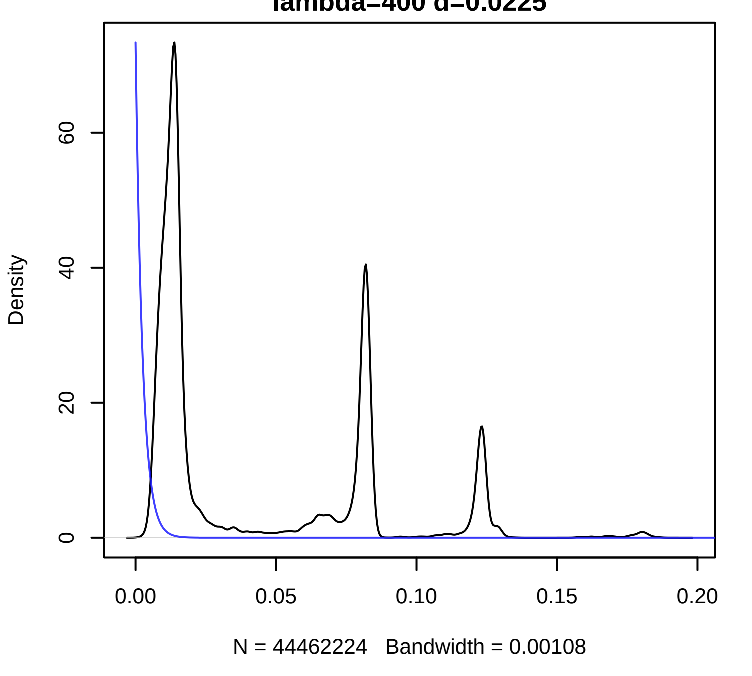

lambda=400 d=0.0225 r=0 knn=Inf

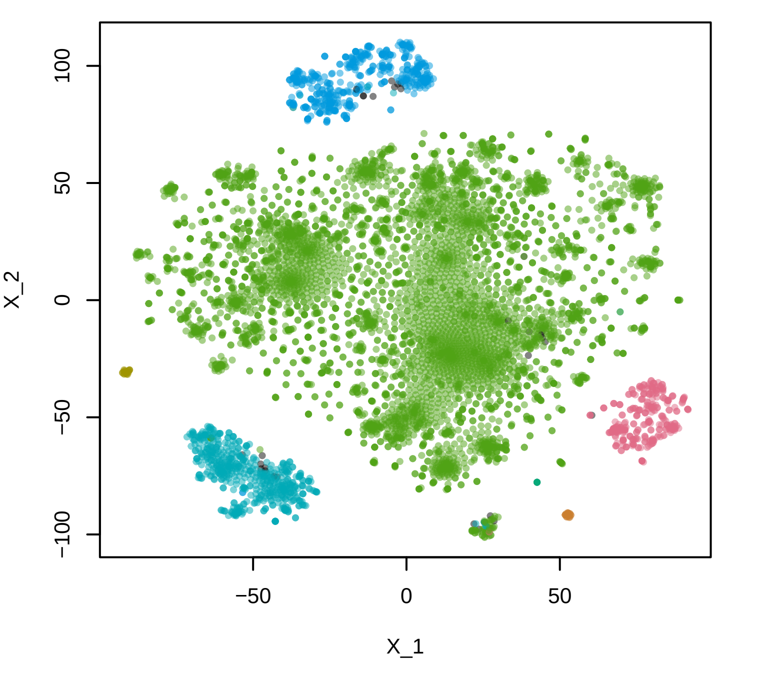

lambda=400 d=0.0225 r=0.333333333333333 knn=Inf

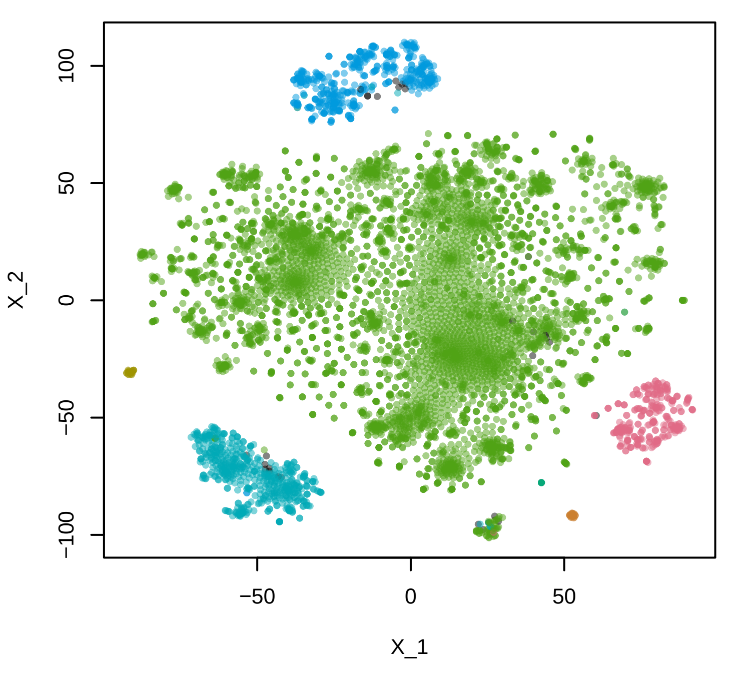

lambda=400 d=0.0225 r=0.666666666666667 knn=Inf

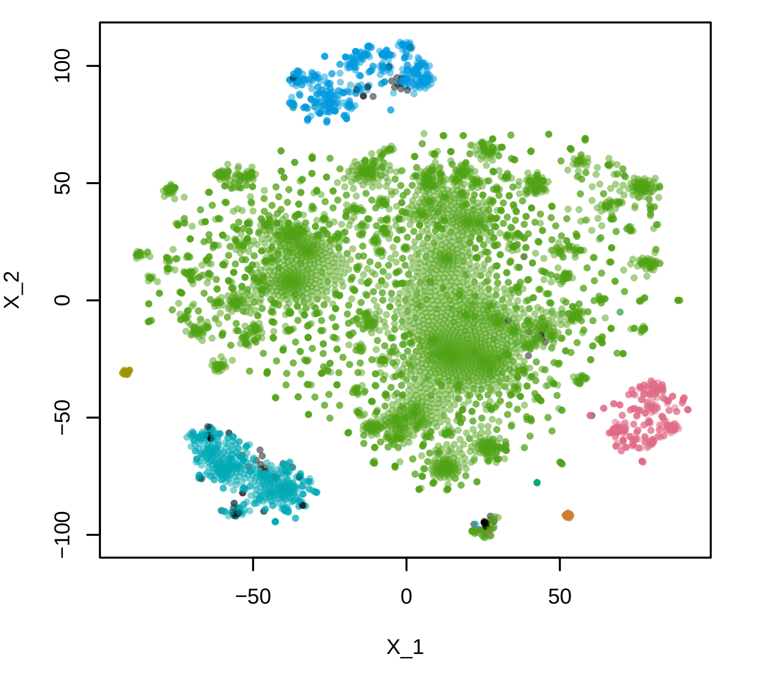

Distribution of IBS values with relative scaling factor for weighting (blue) lambda=400 d=0.025

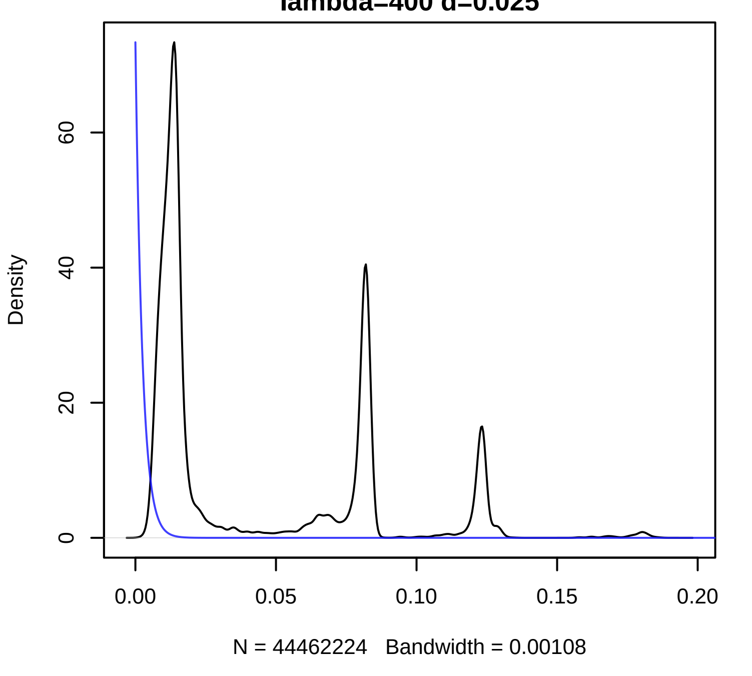

lambda=400 d=0.025 r=0 knn=Inf

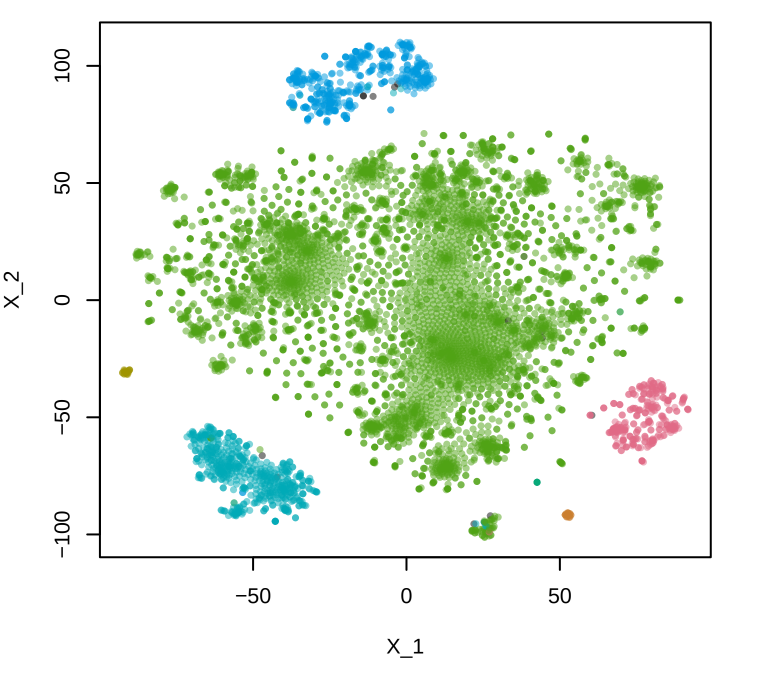

lambda=400 d=0.025 r=0.333333333333333 knn=Inf

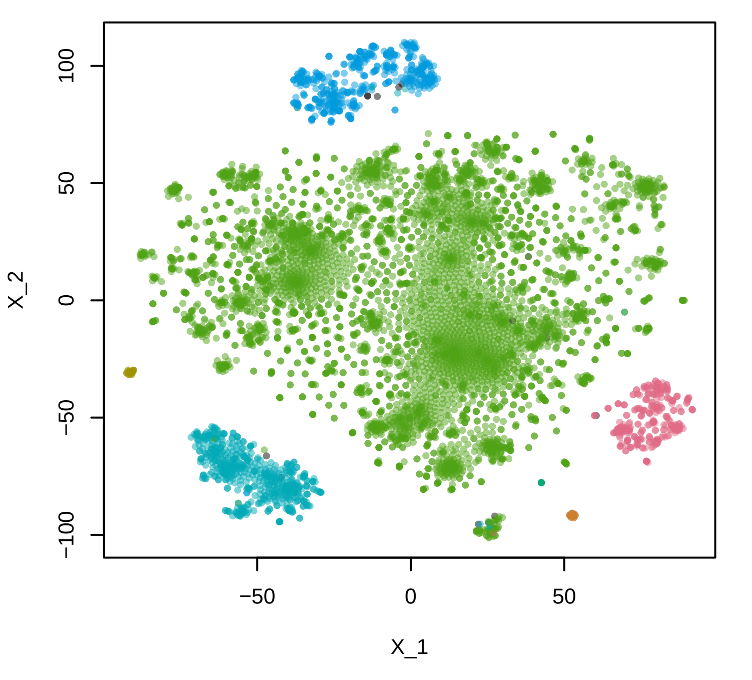

lambda=400 d=0.025 r=0.666666666666667 knn=Inf

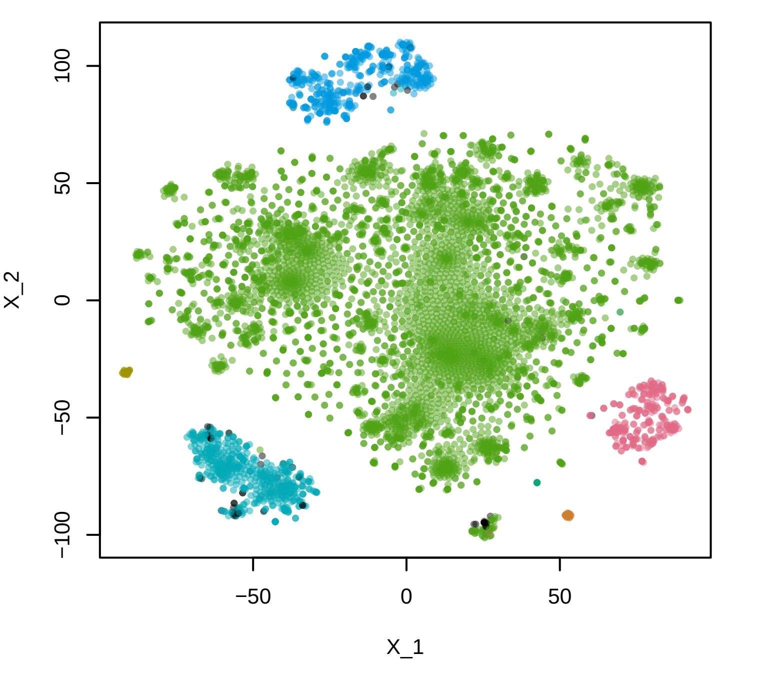

Distribution of IBS values with relative scaling factor for weighting (blue) lambda=400 d=0.0275

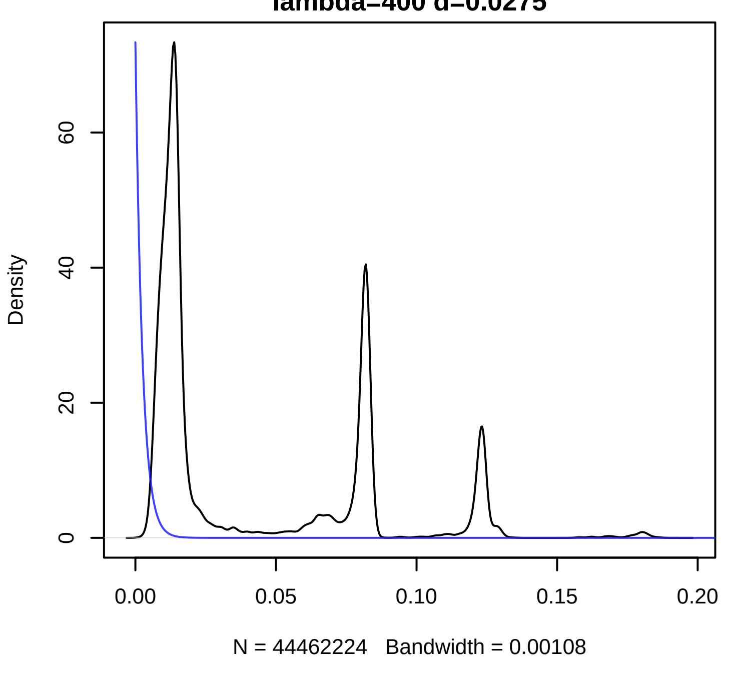

lambda=400 d=0.0275 r=0 knn=Inf

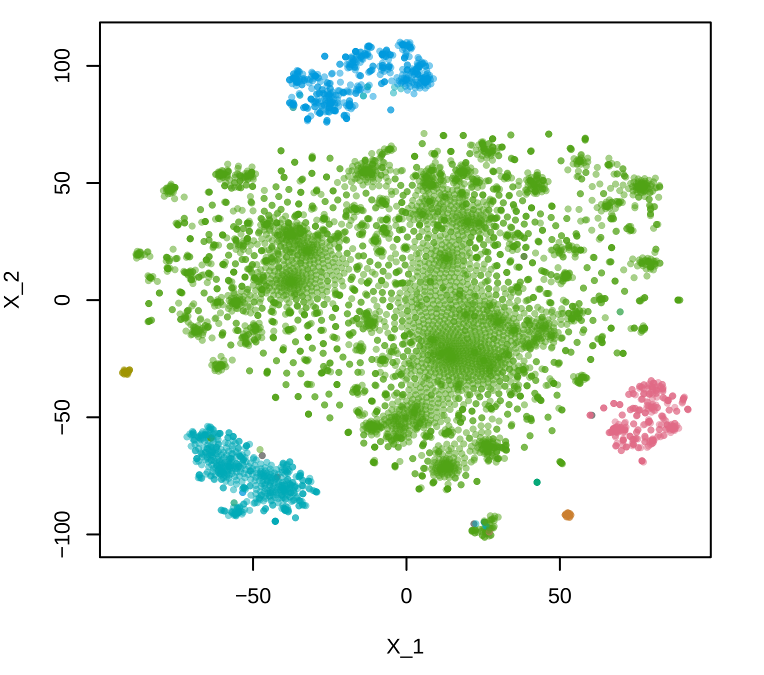

lambda=400 d=0.0275 r=0.333333333333333 knn=Inf

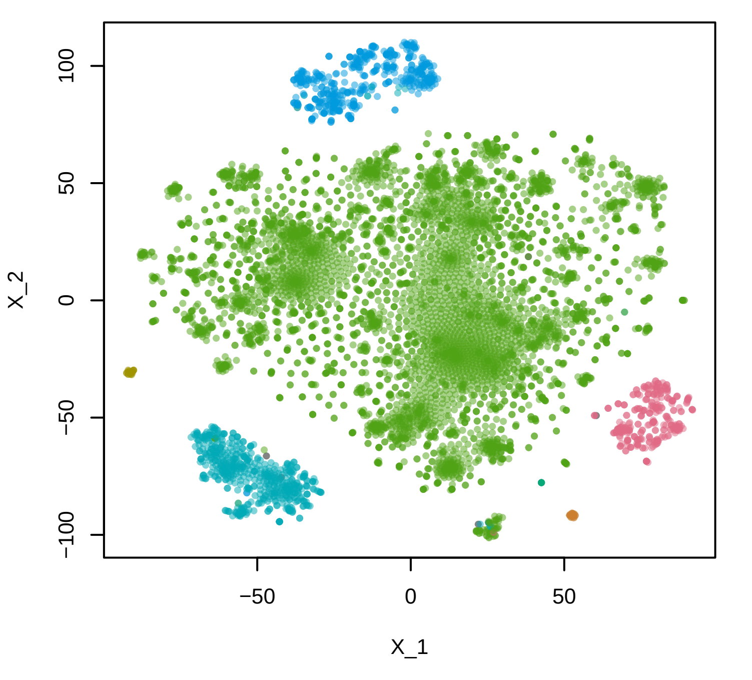

lambda=400 d=0.0275 r=0.666666666666667 knn=Inf

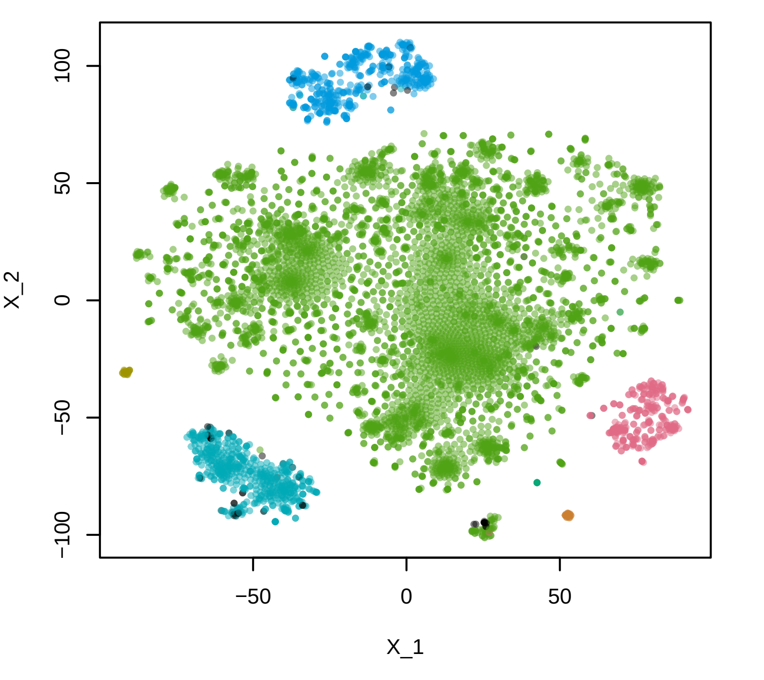

Distribution of IBS values with relative scaling factor for weighting (blue) lambda=400 d=0.03

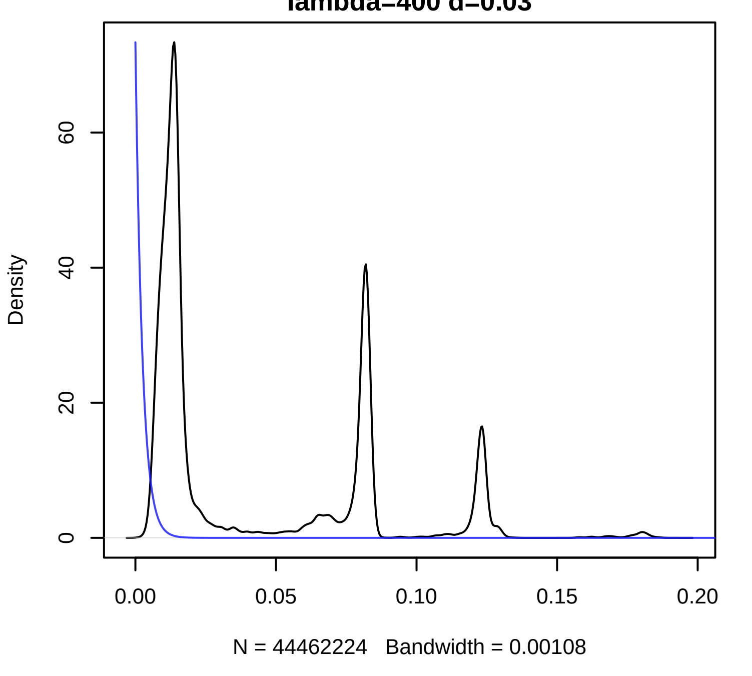

lambda=400 d=0.03 r=0 knn=Inf

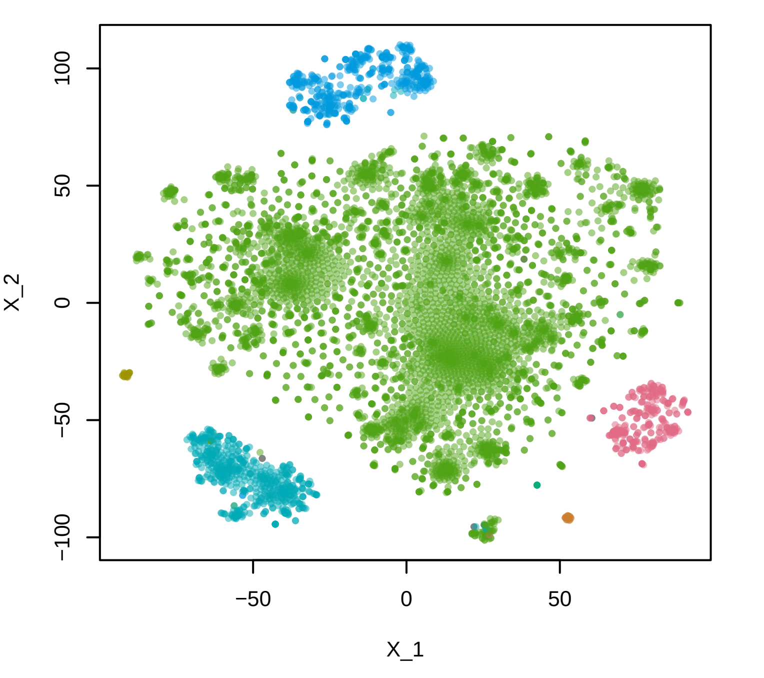

lambda=400 d=0.03 r=0.333333333333333 knn=Inf

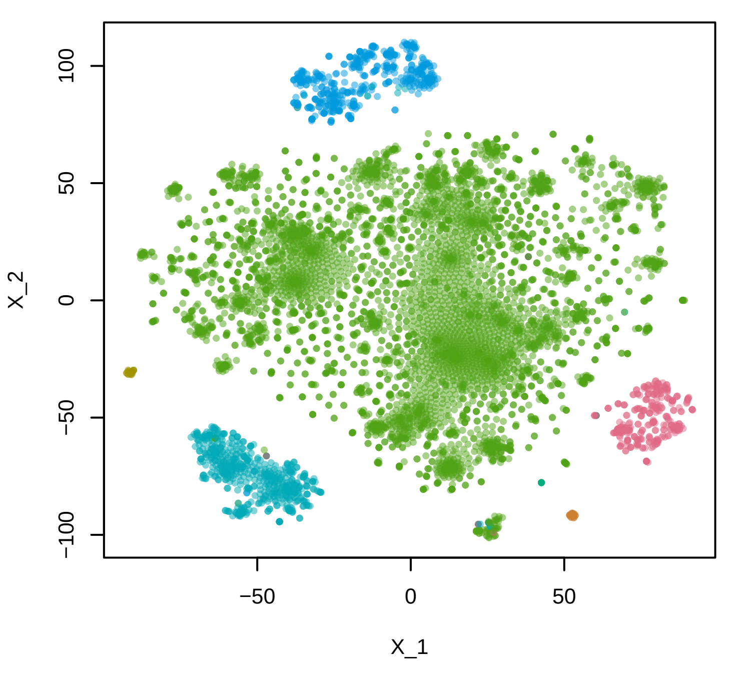

lambda=400 d=0.03 r=0.666666666666667 knn=Inf

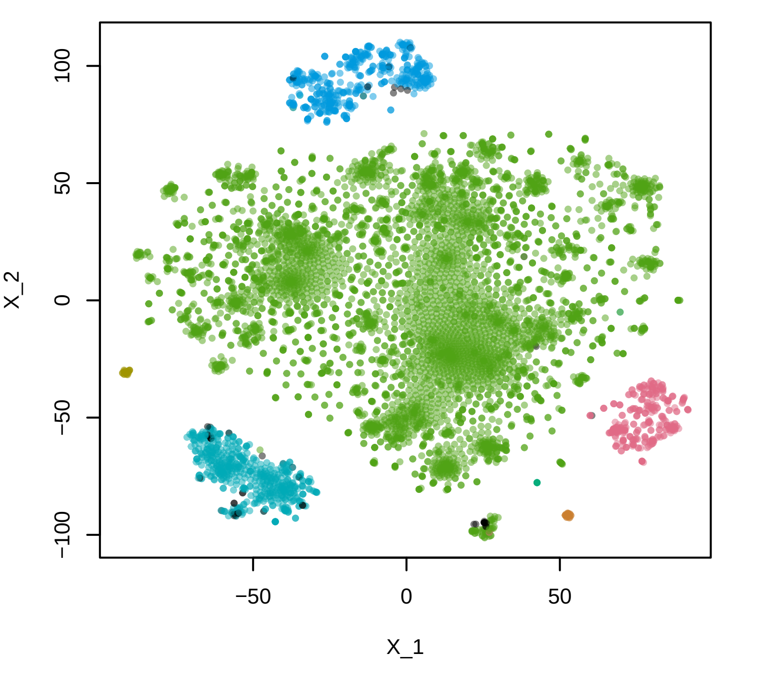

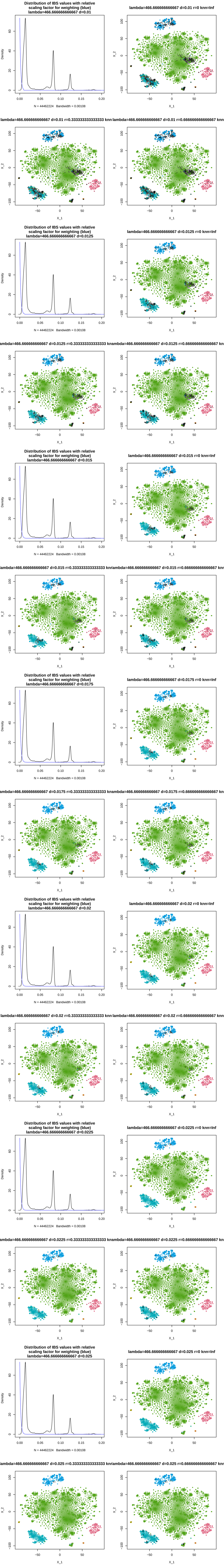
Distribution of IBS values with relative scaling factor for weighting (blue) lambda=466.666666666667 d=0.01
N = 44462224 Bandwidth = 0.00108
lambda=466.666666666667 d=0.01 r=0 knn=Inf
lambda=466.666666666667 d=0.01 r=0.333333333333333 knn
lambda=466.666666666667 d=0.01 r=0.666666666666667 knn
Distribution of IBS values with relative scaling factor for weighting (blue) lambda=466.666666666667 d=0.0125
N = 44462224 Bandwidth = 0.00108
lambda=466.666666666667 d=0.0125 r=0 knn=Inf
ambda=466.666666666667 d=0.0125 r=0.333333333333333 kn
ambda=466.666666666667 d=0.0125 r=0.666666666666667 kn
Distribution of IBS values with relative scaling factor for weighting (blue) lambda=466.666666666667 d=0.015
N = 44462224 Bandwidth = 0.00108
lambda=466.666666666667 d=0.015 r=0 knn=Inf
lambda=466.666666666667 d=0.015 r=0.333333333333333 kn
lambda=466.666666666667 d=0.015 r=0.666666666666667 kn
Distribution of IBS values with relative scaling factor for weighting (blue) lambda=466.666666666667 d=0.0175
N = 44462224 Bandwidth = 0.00108
lambda=466.666666666667 d=0.0175 r=0 knn=Inf
ambda=466.666666666667 d=0.0175 r=0.333333333333333 kn
ambda=466.666666666667 d=0.0175 r=0.666666666666667 kn
Distribution of IBS values with relative scaling factor for weighting (blue) lambda=466.666666666667 d=0.02
N = 44462224 Bandwidth = 0.00108
lambda=466.666666666667 d=0.02 r=0 knn=Inf
lambda=466.666666666667 d=0.02 r=0.333333333333333 knn
lambda=466.666666666667 d=0.02 r=0.666666666666667 knn
Distribution of IBS values with relative scaling factor for weighting (blue) lambda=466.666666666667 d=0.0225
N = 44462224 Bandwidth = 0.00108
lambda=466.666666666667 d=0.0225 r=0 knn=Inf
ambda=466.666666666667 d=0.0225 r=0.333333333333333 kn
ambda=466.666666666667 d=0.0225 r=0.666666666666667 kn
Distribution of IBS values with relative scaling factor for weighting (blue) lambda=466.666666666667 d=0.025
N = 44462224 Bandwidth = 0.00108
lambda=466.666666666667 d=0.025 r=0 knn=Inf
ambda=466.666666666667 d=0.025 r=0.333333333333333 kn
ambda=466.666666666667 d=0.025 r=0.666666666666667 kn
Density
X_1
X_2

Distribution of IBS values with relative scaling factor for weighting (blue) lambda=466.666666666667 d=0.0275

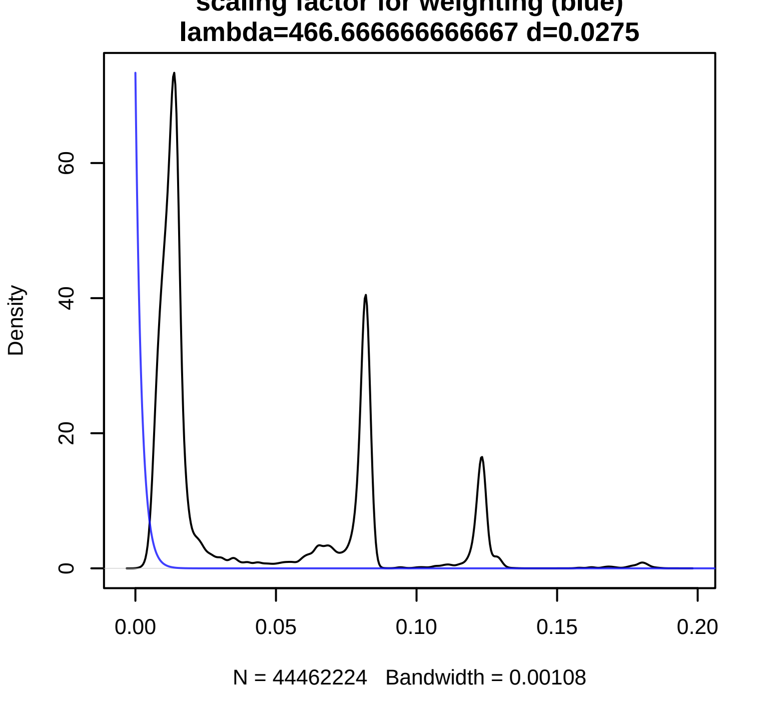

lambda=466.666666666667 d=0.0275 r=0 knn=Inf

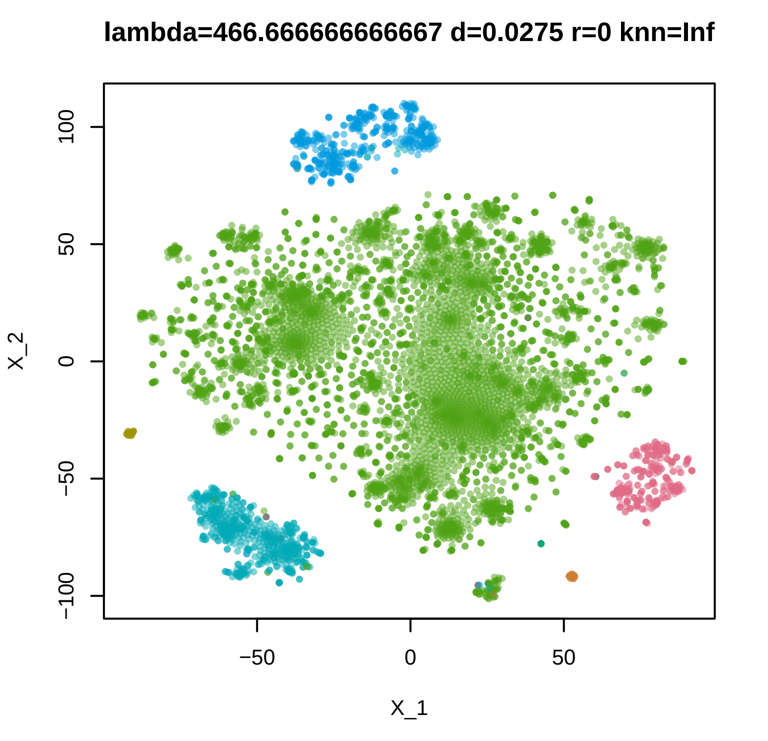

lambda=466.666666666667 d=0.0275 r=0.333333333333333 kn

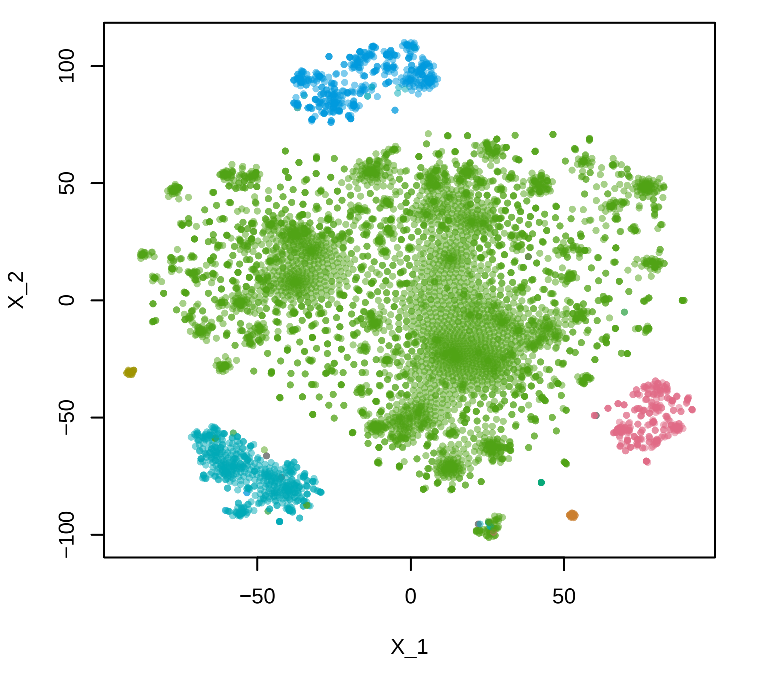

lambda=466.666666666667 d=0.0275 r=0.666666666666667 kn

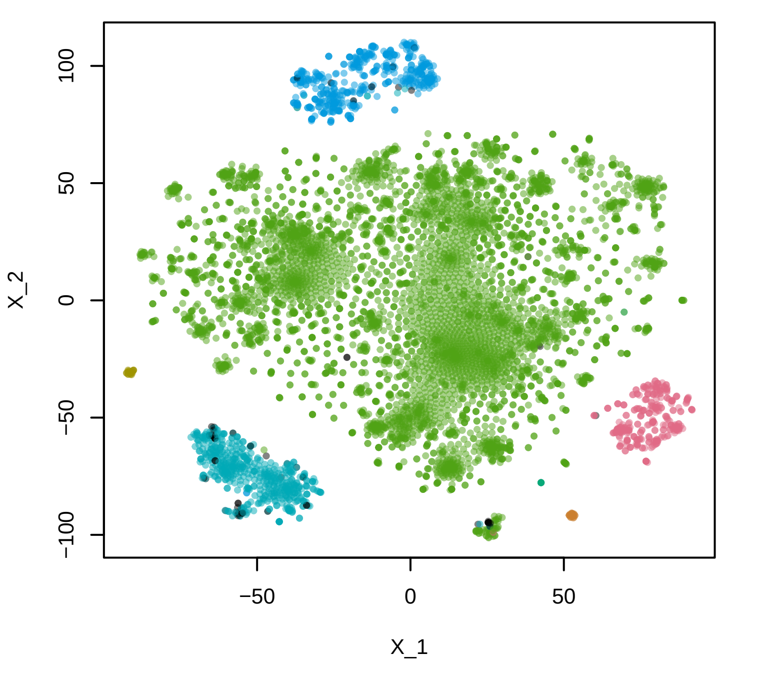

Distribution of IBS values with relative scaling factor for weighting (blue) lambda=466.666666666667 d=0.03

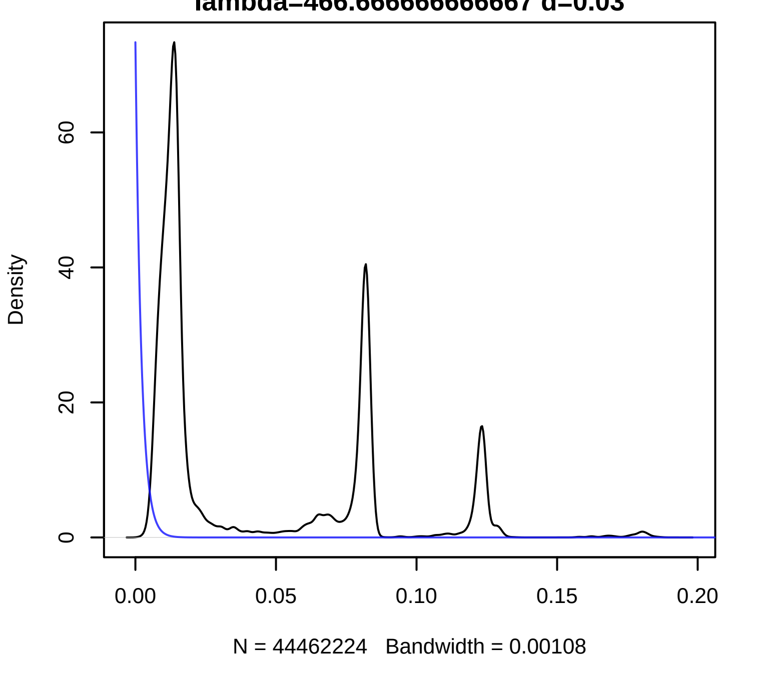

lambda=466.666666666667 d=0.03 r=0 knn=Inf

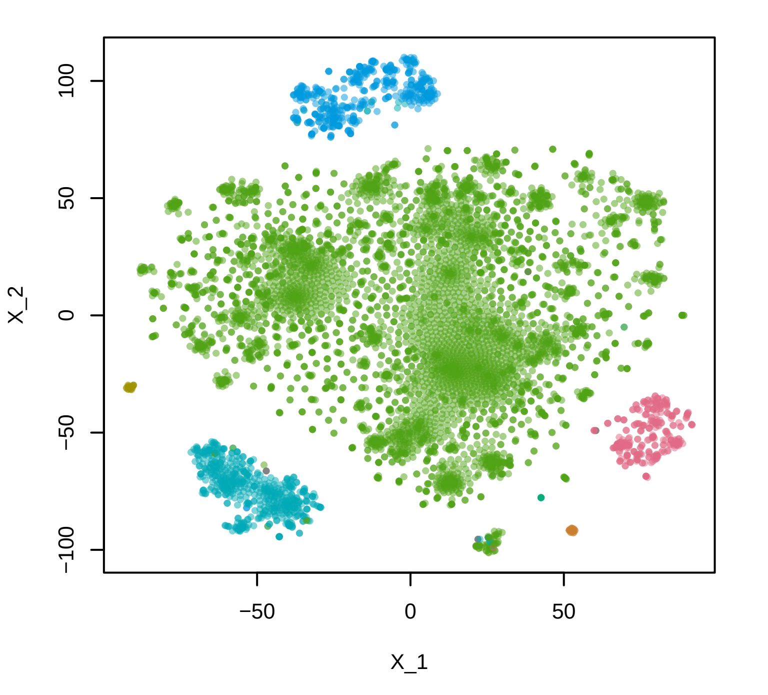

lambda=466.666666666667 d=0.03 r=0.333333333333333 knn

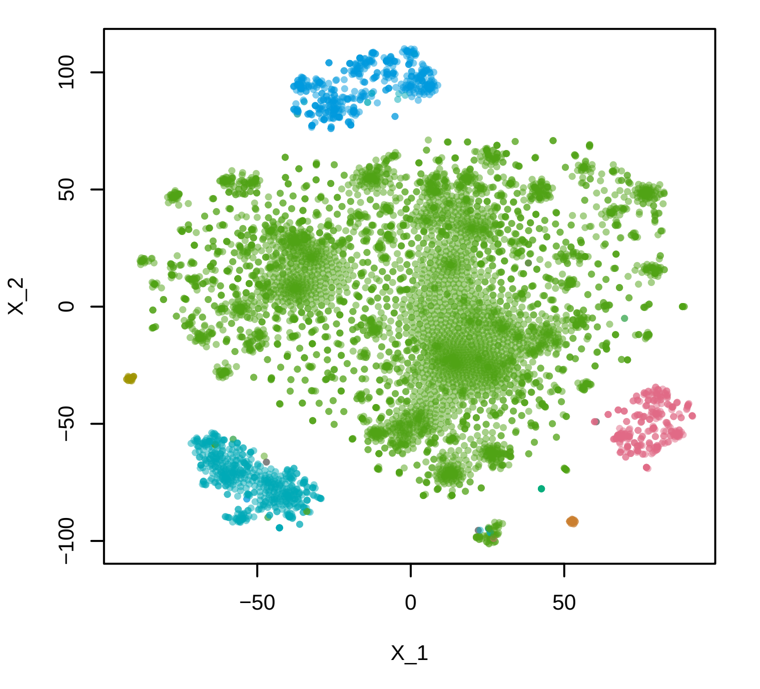

lambda=466.666666666667 d=0.03 r=0.666666666666667 knn

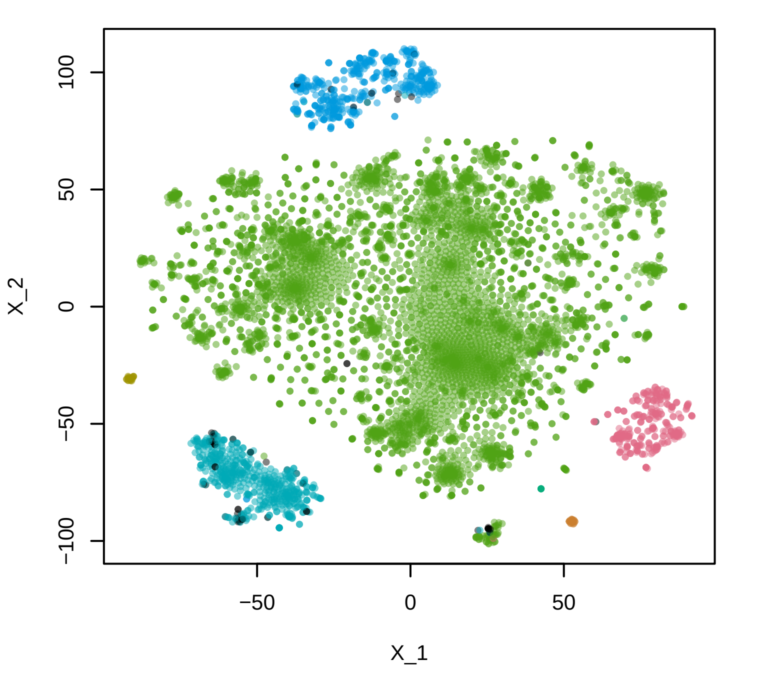

Distribution of IBS values with relative scaling factor for weighting (blue) lambda=533.333333333333 d=0.01

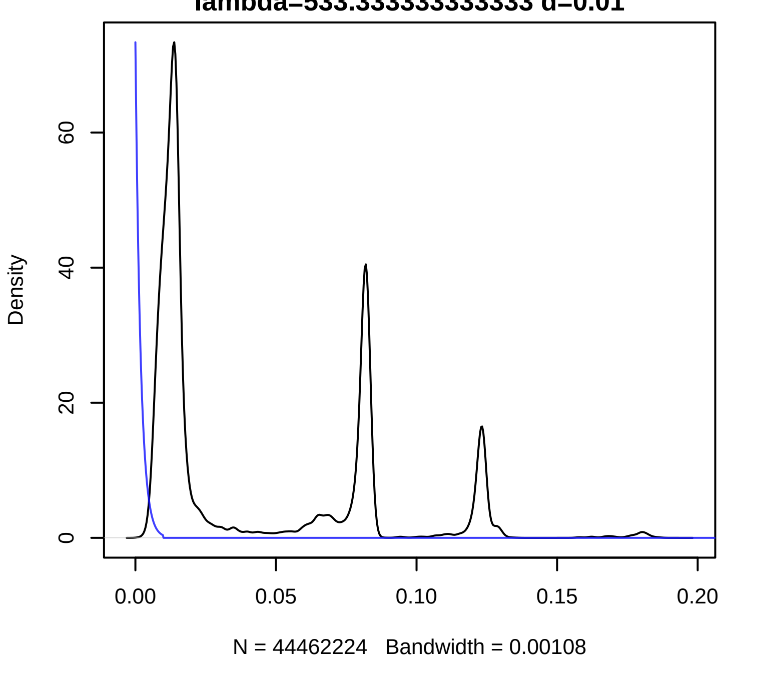

lambda=533.333333333333 d=0.01 r=0 knn=Inf

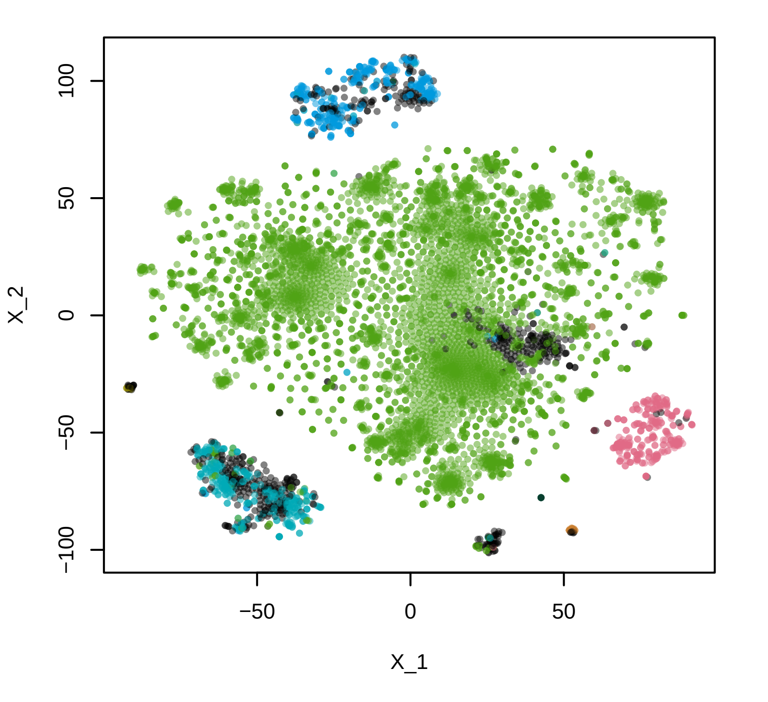

lambda=533.333333333333 d=0.01 r=0.333333333333333 knn

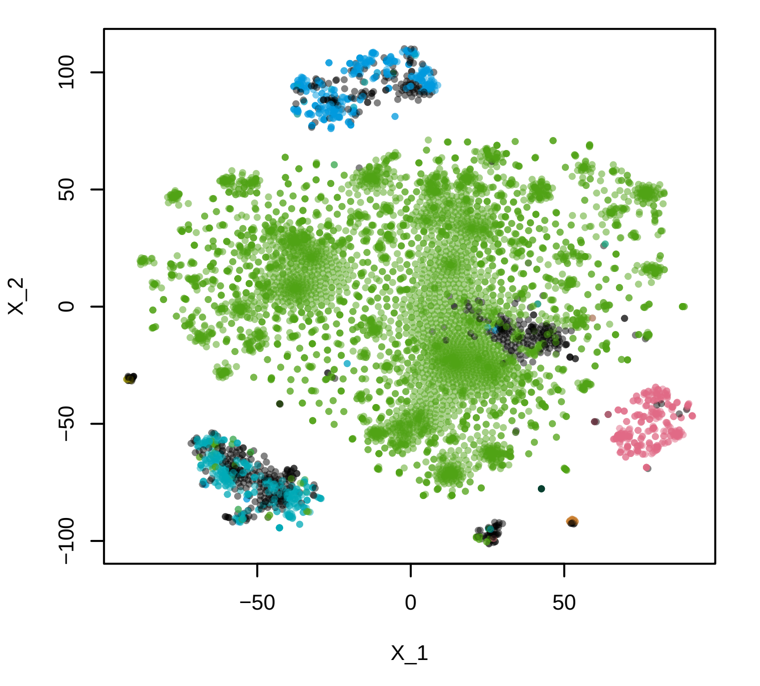

lambda=533.333333333333 d=0.01 r=0.666666666666667 knn

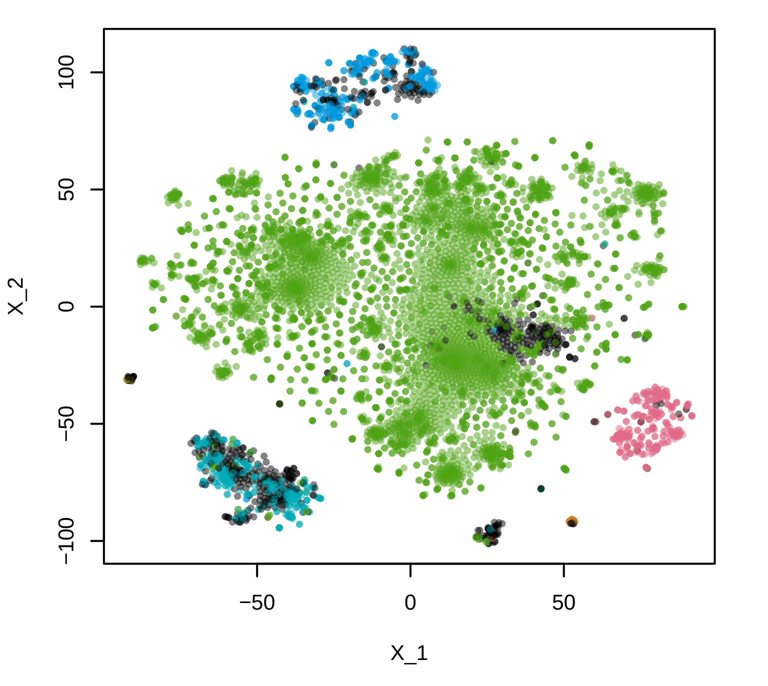

Distribution of IBS values with relative scaling factor for weighting (blue) lambda=533.333333333333 d=0.0125

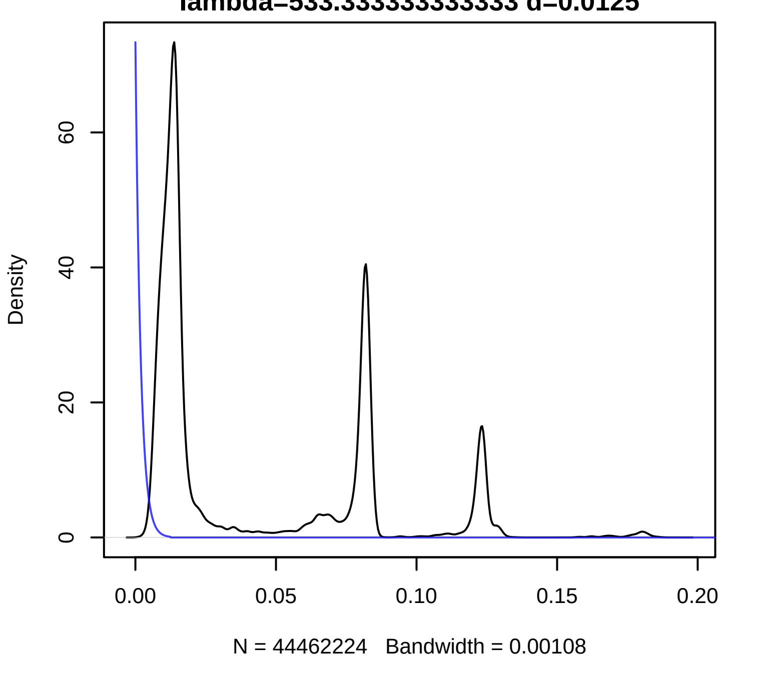

lambda=533.333333333333 d=0.0125 r=0 knn=Inf

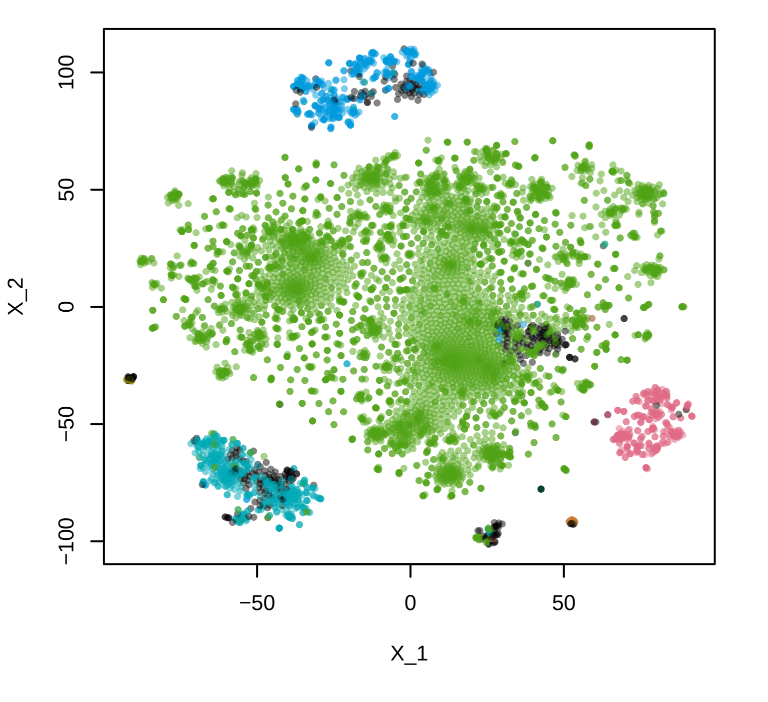

ambda=533.333333333333 d=0.0125 r=0.333333333333333 kn

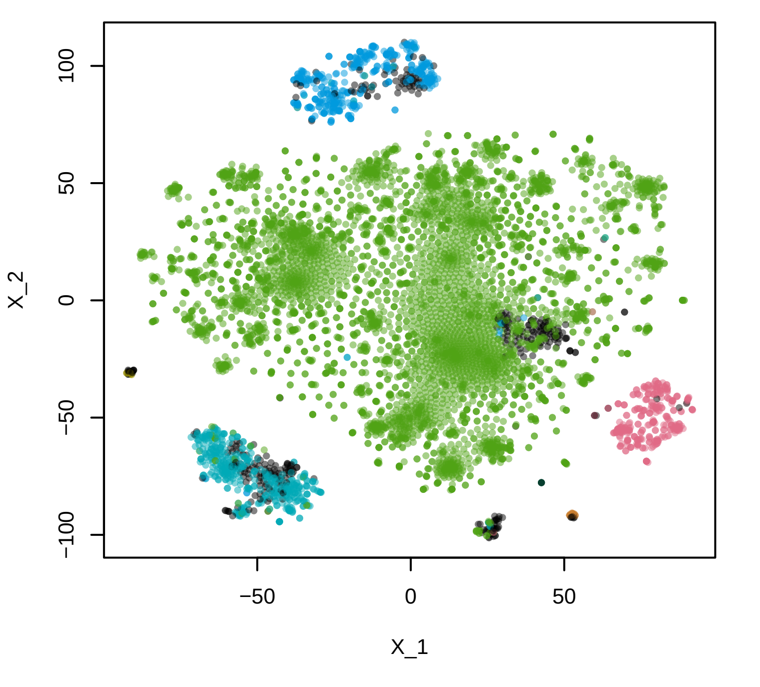

lambda=533.333333333333 d=0.0125 r=0.666666666666667 kn

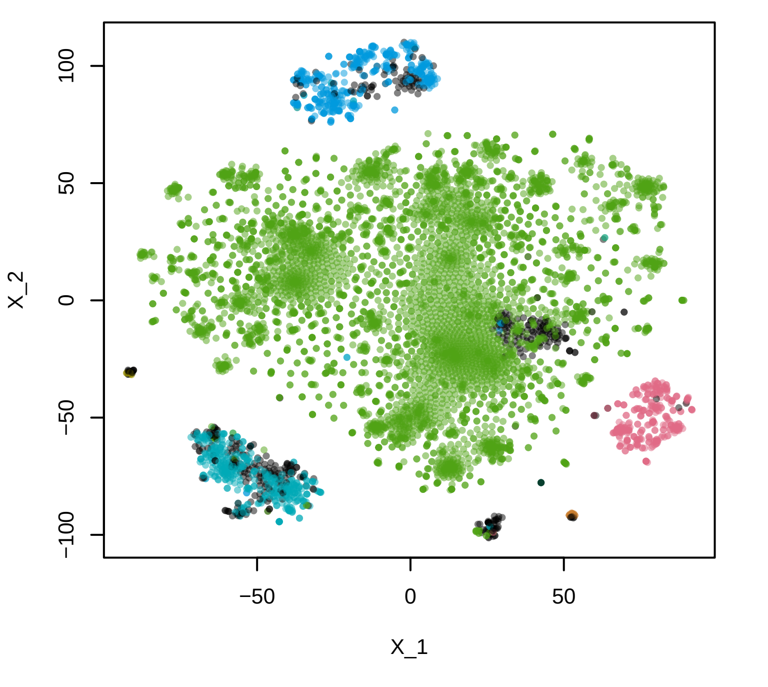

Distribution of IBS values with relative scaling factor for weighting (blue) lambda=533.333333333333 d=0.015

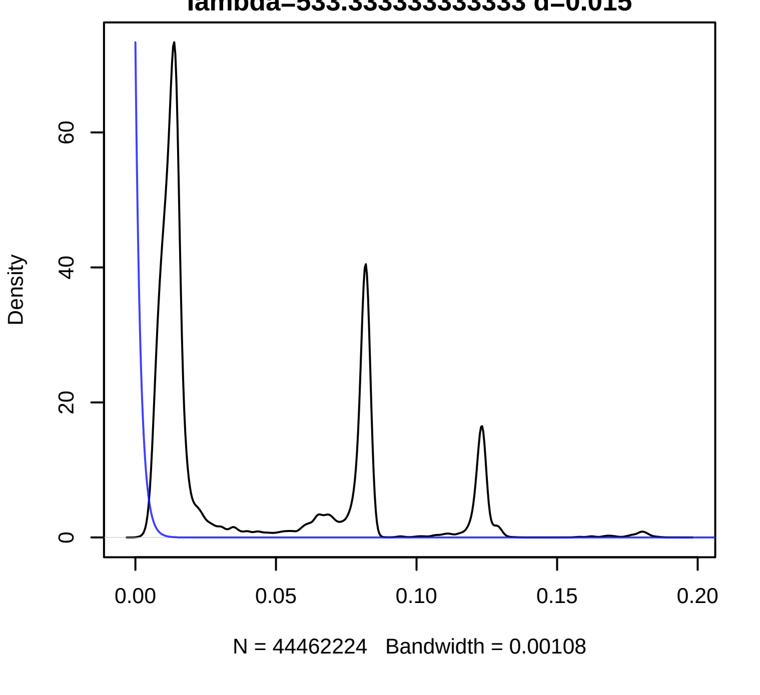

lambda=533.333333333333 d=0.015 r=0 knn=Inf

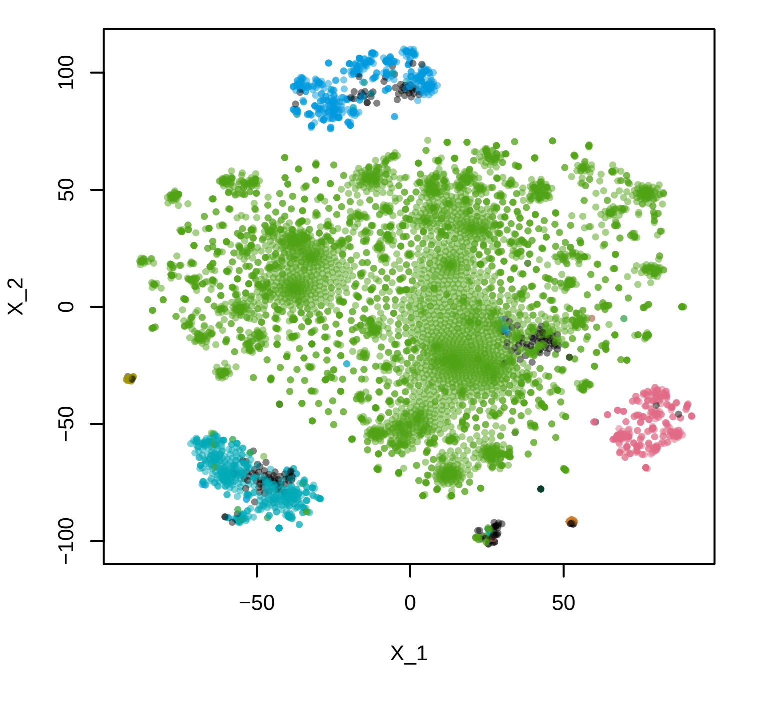

lambda=533.333333333333 d=0.015 r=0.333333333333333 kn

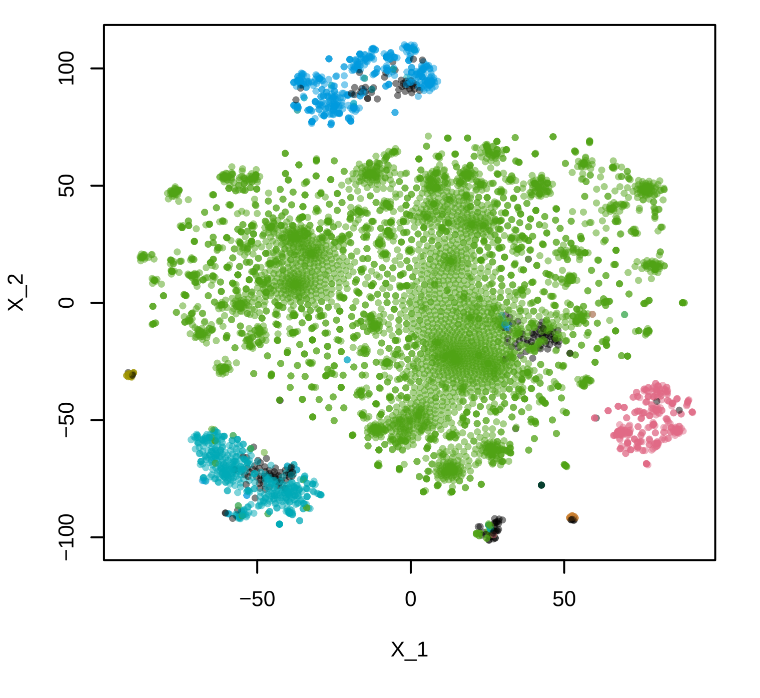

lambda=533.333333333333 d=0.015 r=0.666666666666667 kn

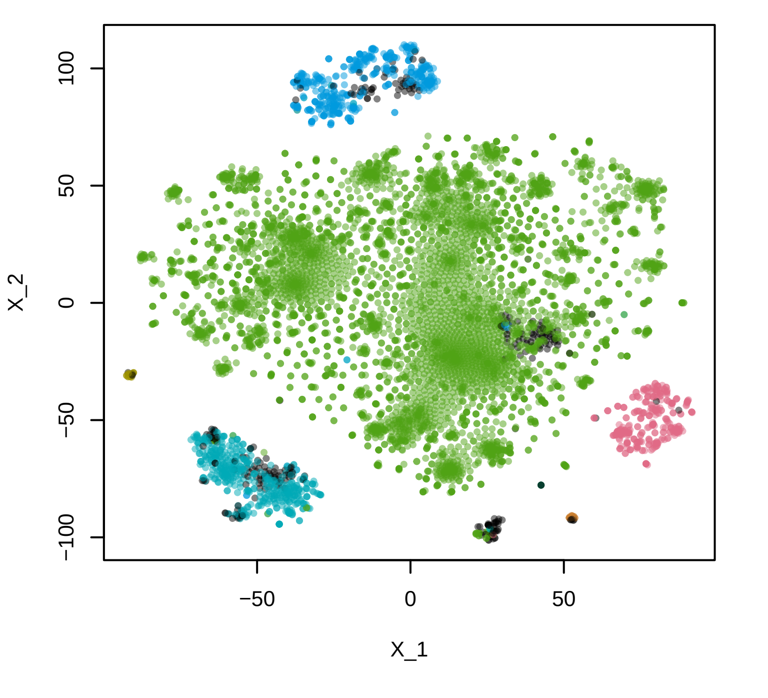

Distribution of IBS values with relative scaling factor for weighting (blue) lambda=533.333333333333 d=0.0175

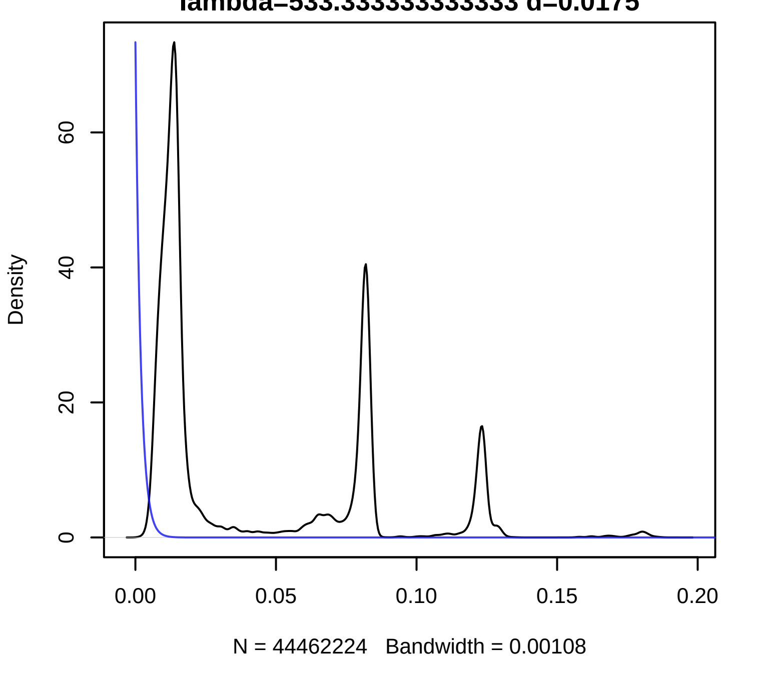

lambda=533.333333333333 d=0.0175 r=0 knn=Inf

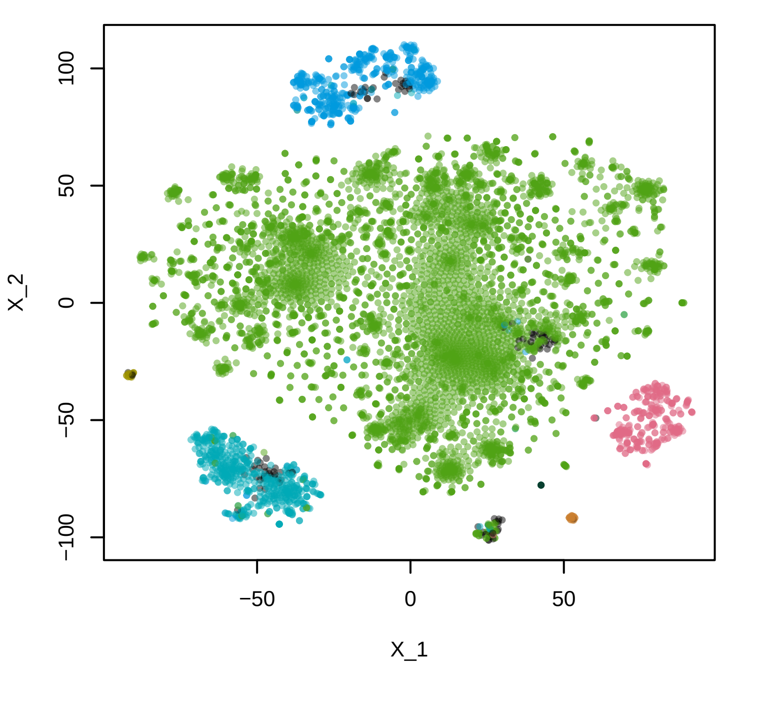

lambda=533.333333333333 d=0.0175 r=0.333333333333333 kn

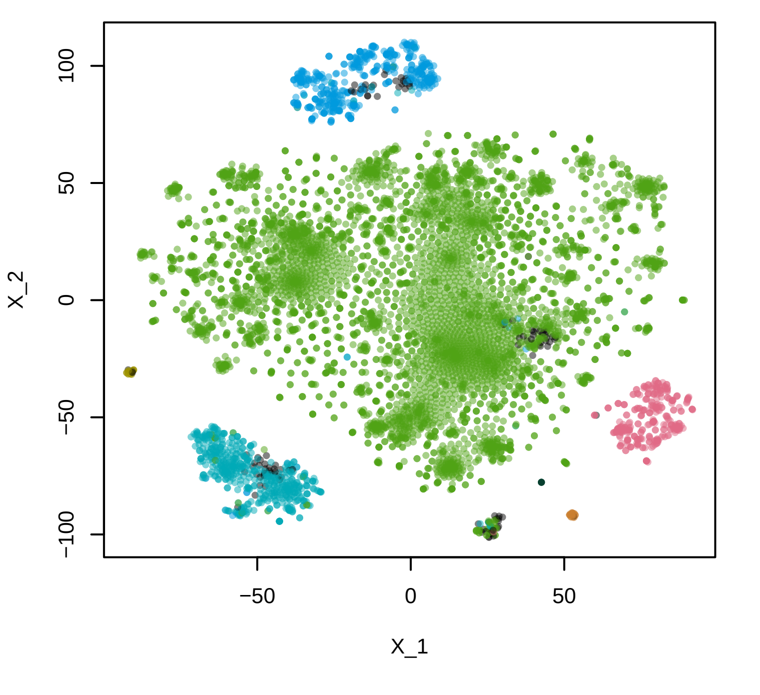

lambda=533.333333333333 d=0.0175 r=0.666666666666667 kn

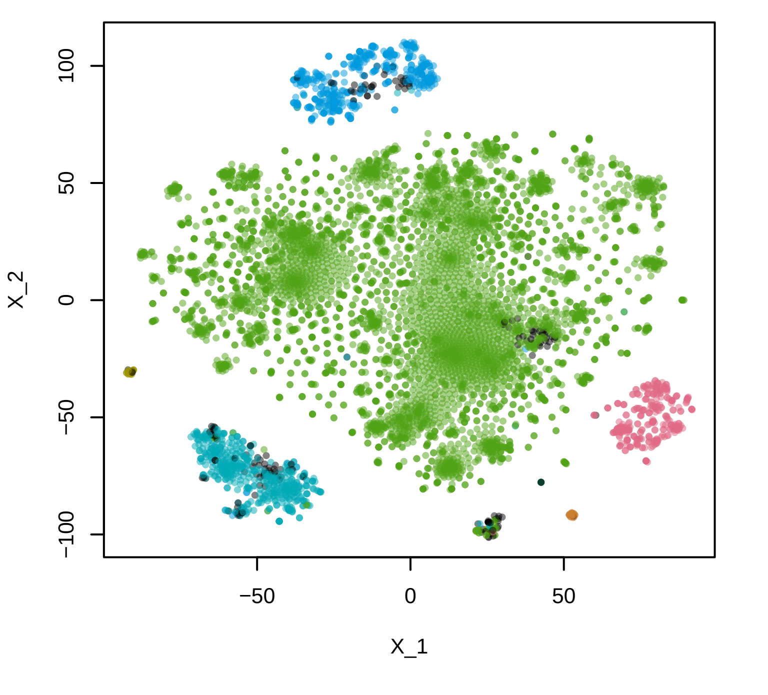

Distribution of IBS values with relative scaling factor for weighting (blue) lambda=533.333333333333 d=0.02

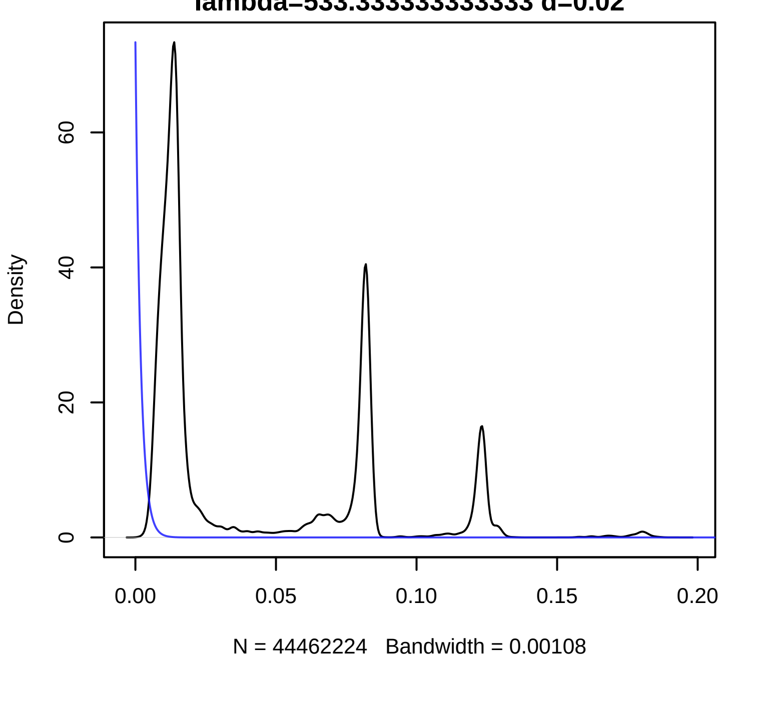

lambda=533.333333333333 d=0.02 r=0 knn=Inf

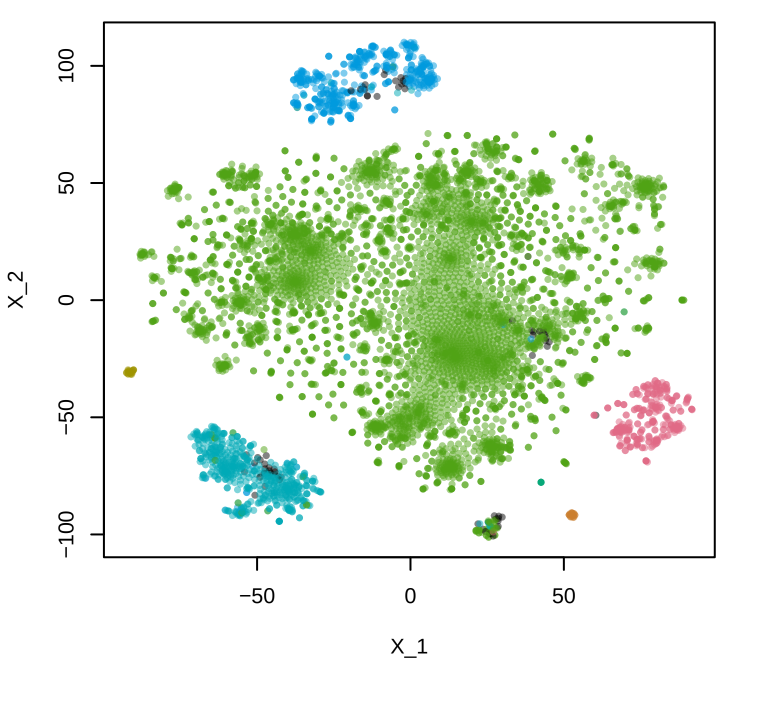

lambda=533.333333333333 d=0.02 r=0.333333333333333 kn

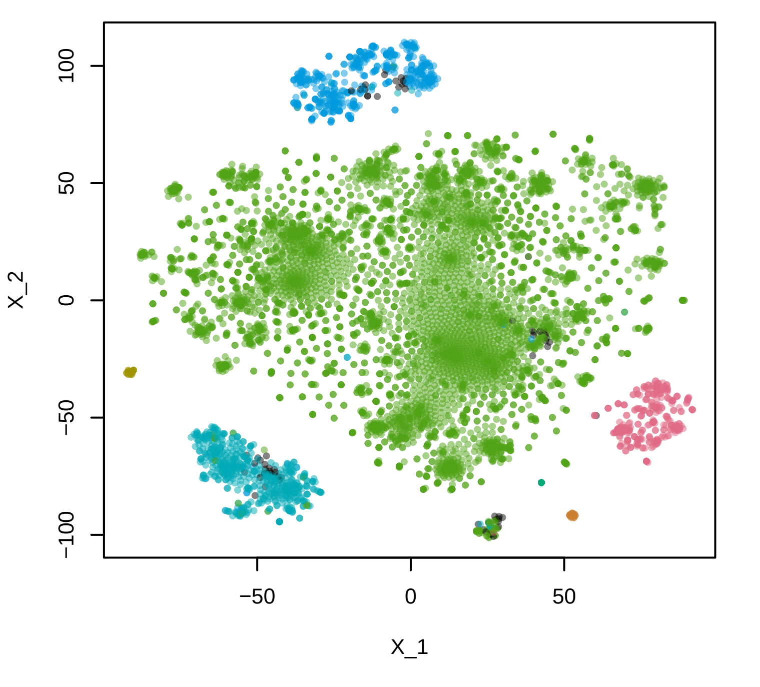

lambda=533.333333333333 d=0.02 r=0.666666666666667 kn

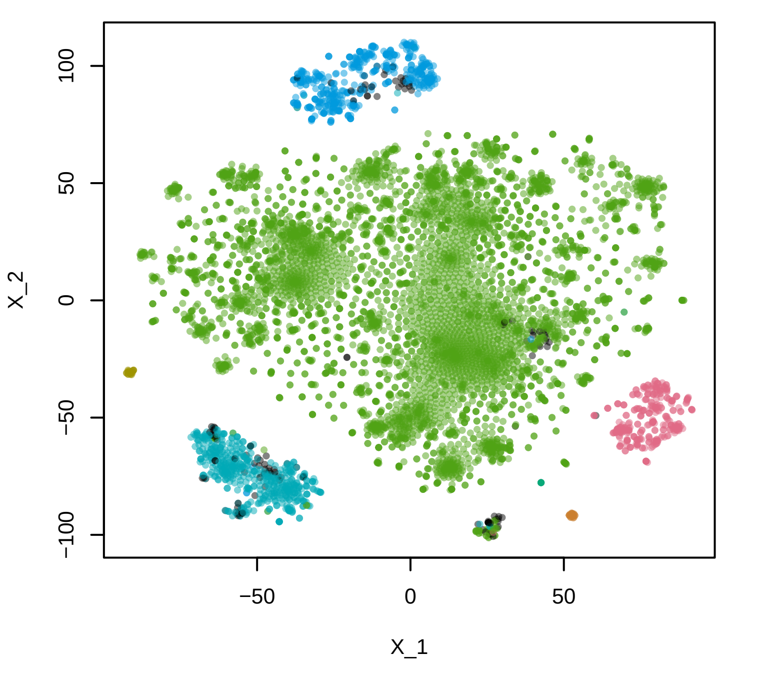

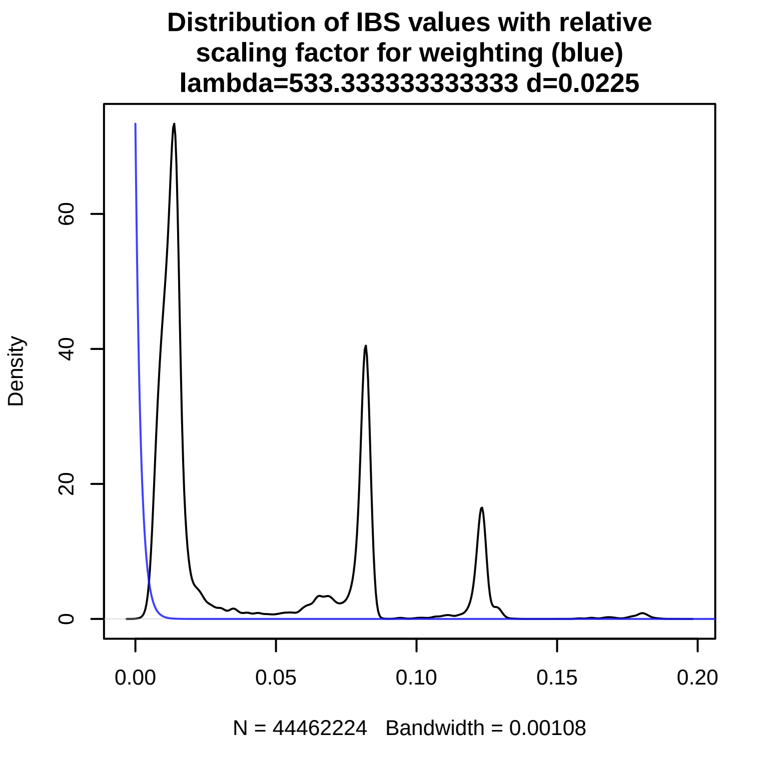
Distribution of IBS values with relative
scaling factor for weighting (blue)
lambda=533.333333333333 d=0.0225
Density
N = 44462224 Bandwidth = 0.00108

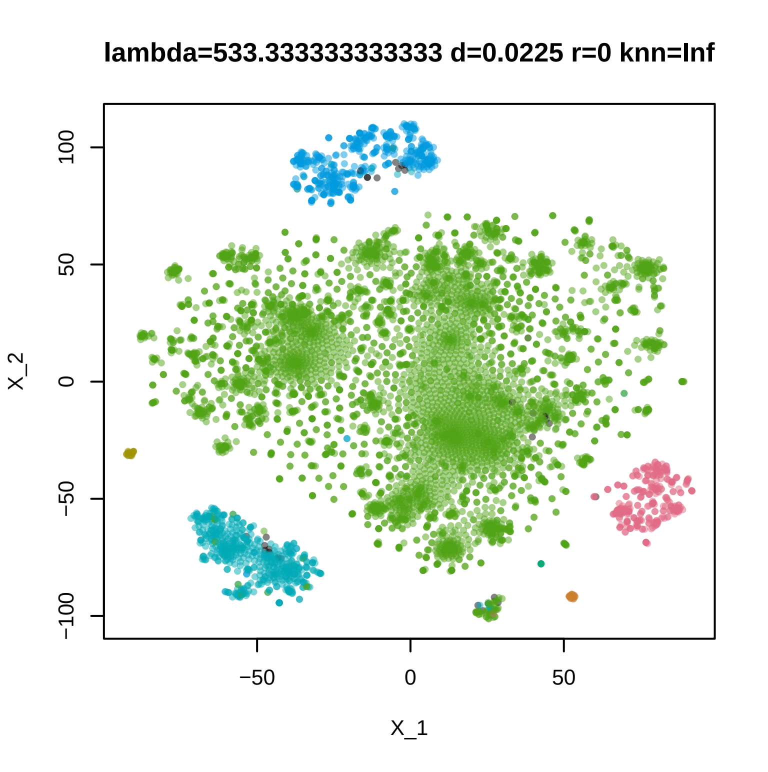
lambda=533.333333333333 d=0.0225 r=0 knn=Inf
X_2
X_1

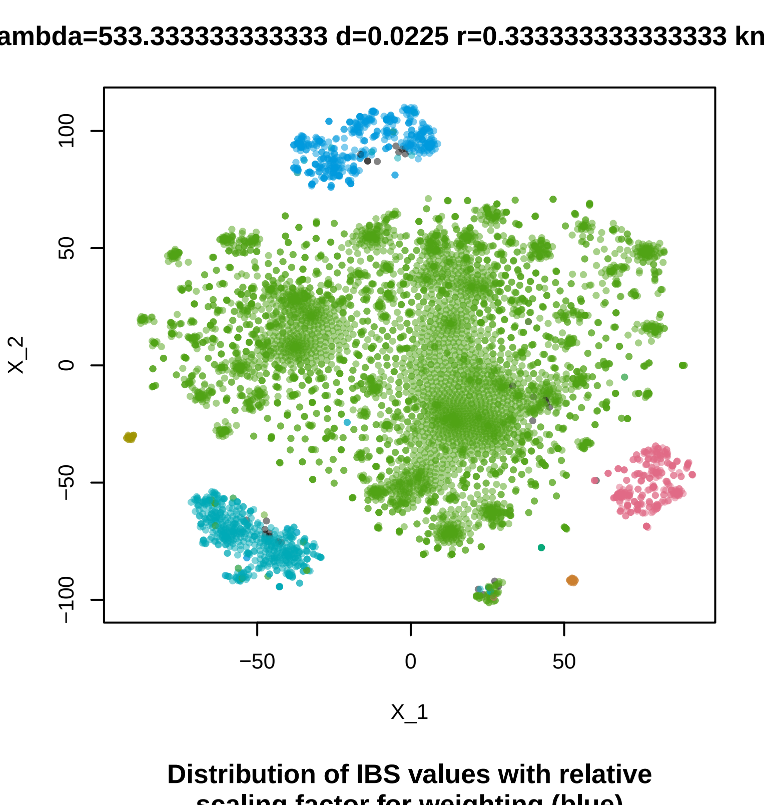
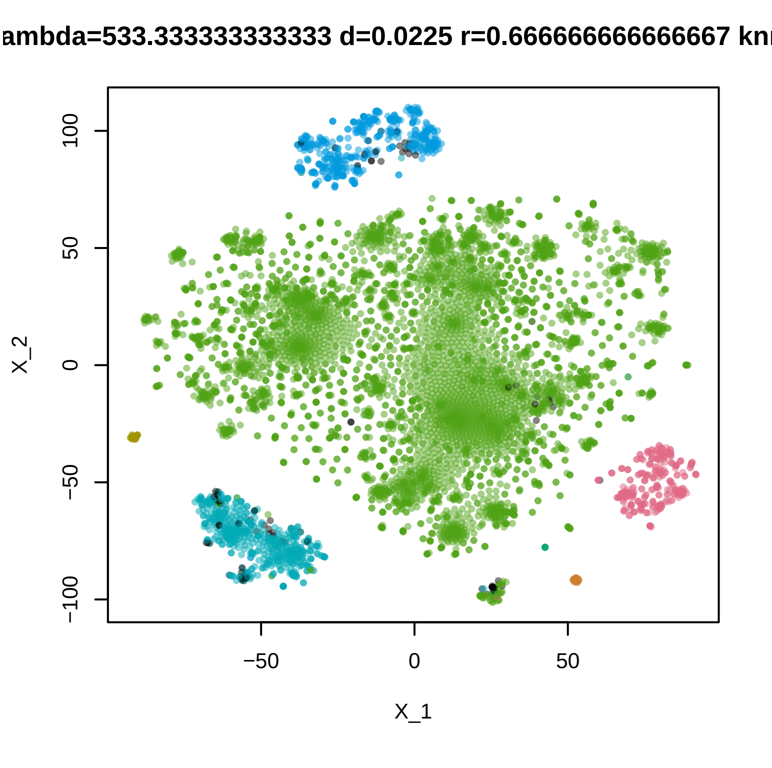
X_2
X_1

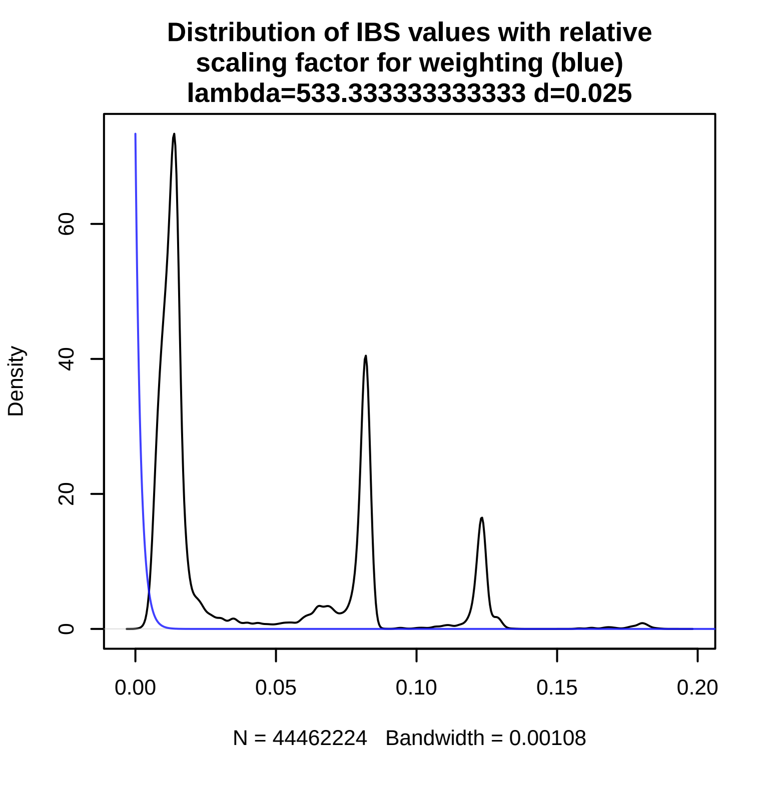
Distribution of IBS values with relative
scaling factor for weighting (blue)
lambda=533.333333333333 d=0.025
Density
N = 44462224 Bandwidth = 0.00108

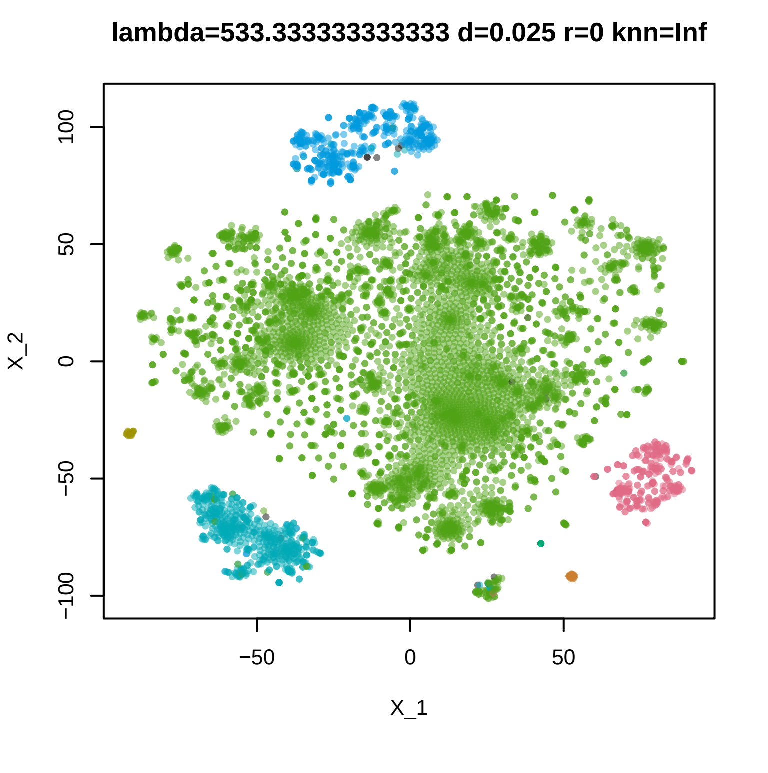
lambda=533.333333333333 d=0.025 r=0 knn=Inf
X_2
X_1

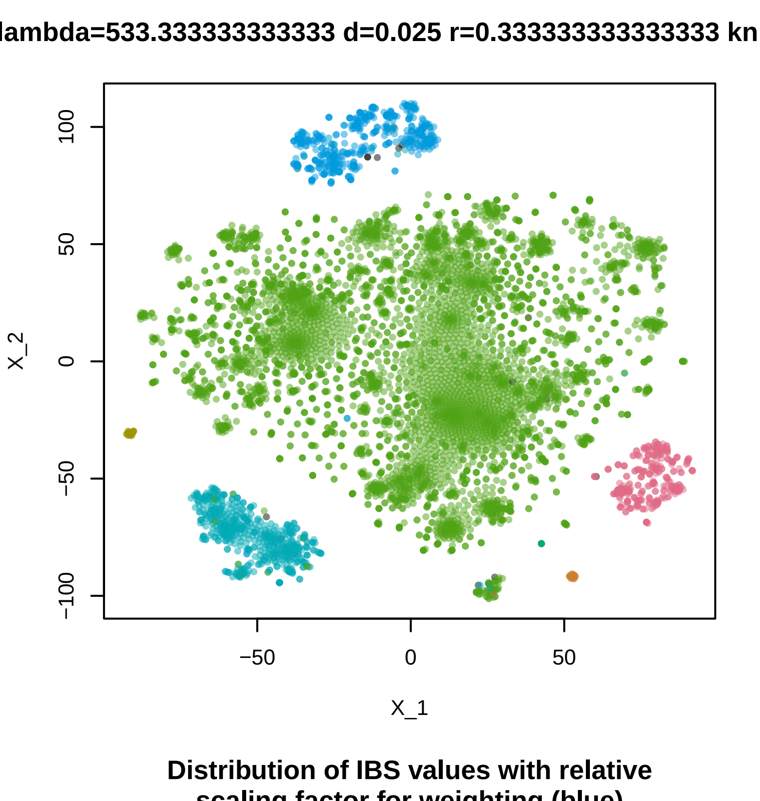
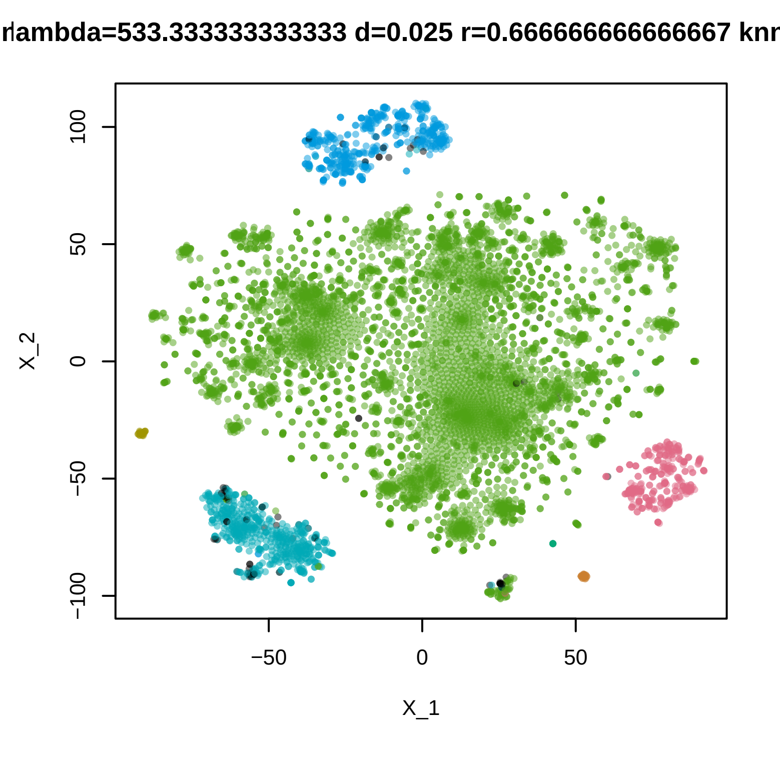
X_2
X_1

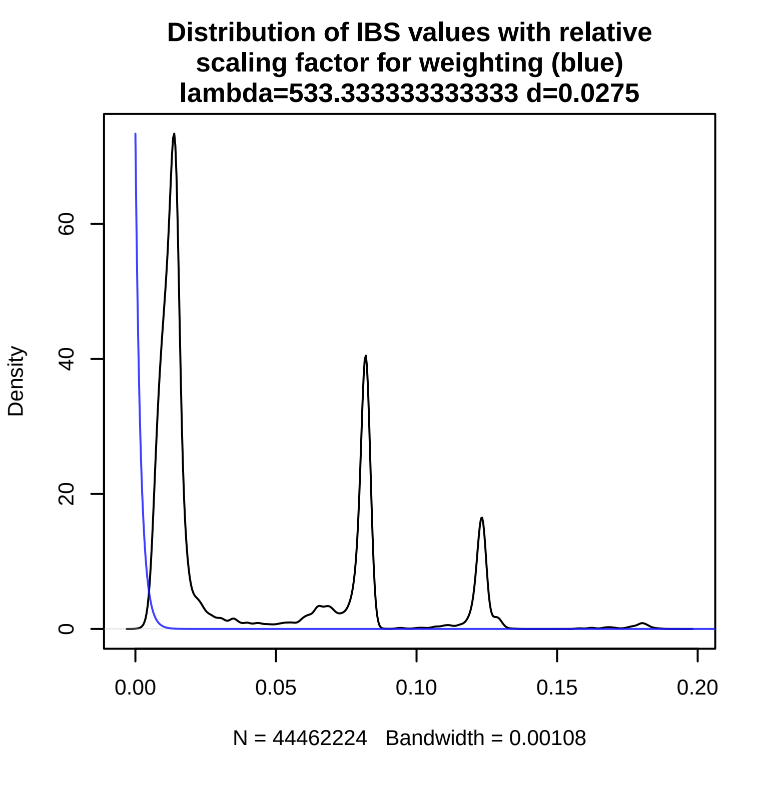
Distribution of IBS values with relative
scaling factor for weighting (blue)
lambda=533.333333333333 d=0.0275
Density
N = 44462224 Bandwidth = 0.00108

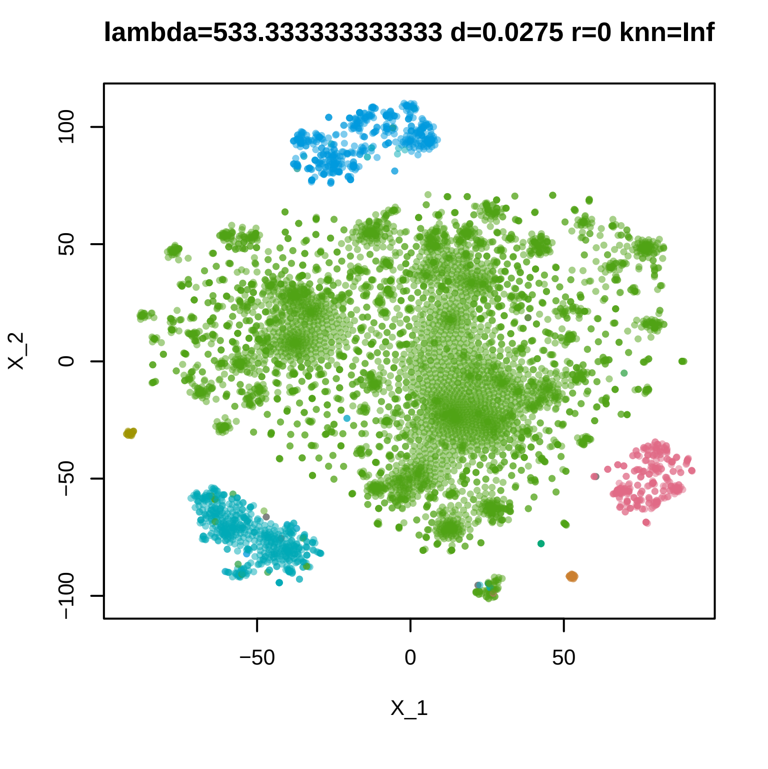
lambda=533.333333333333 d=0.0275 r=0 knn=Inf
X_2
X_1

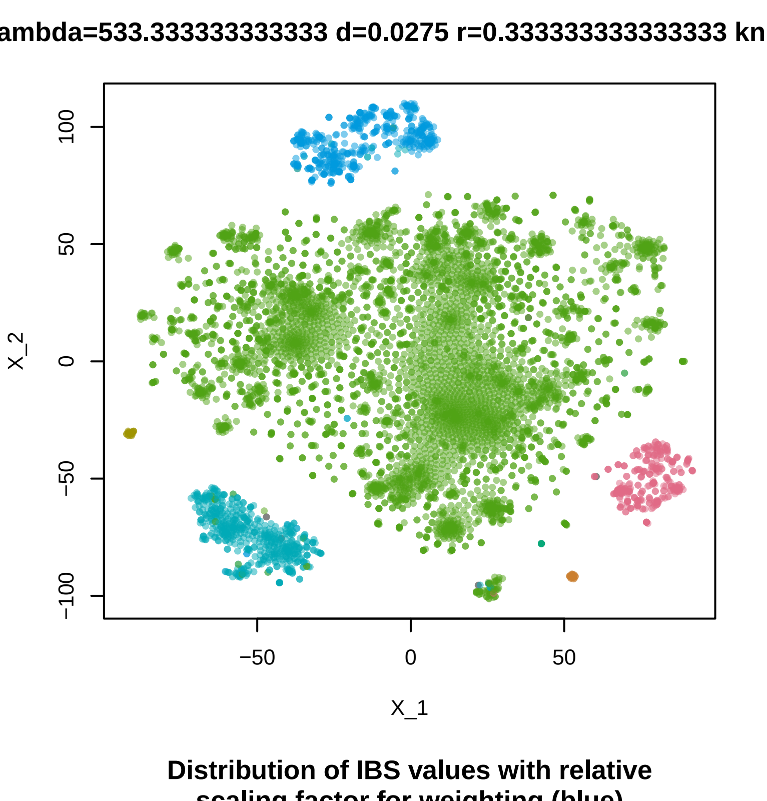
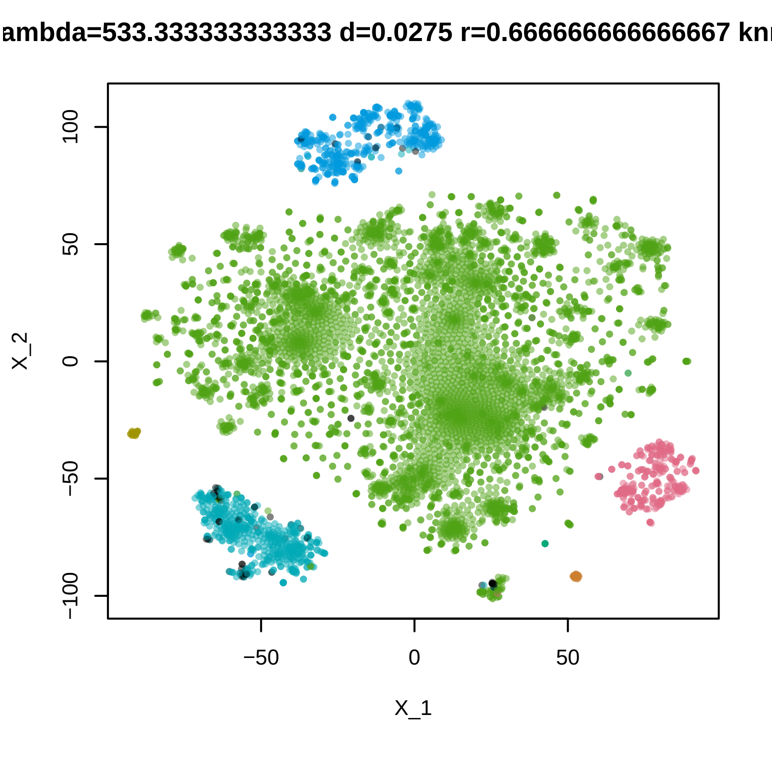
X_2
X_1

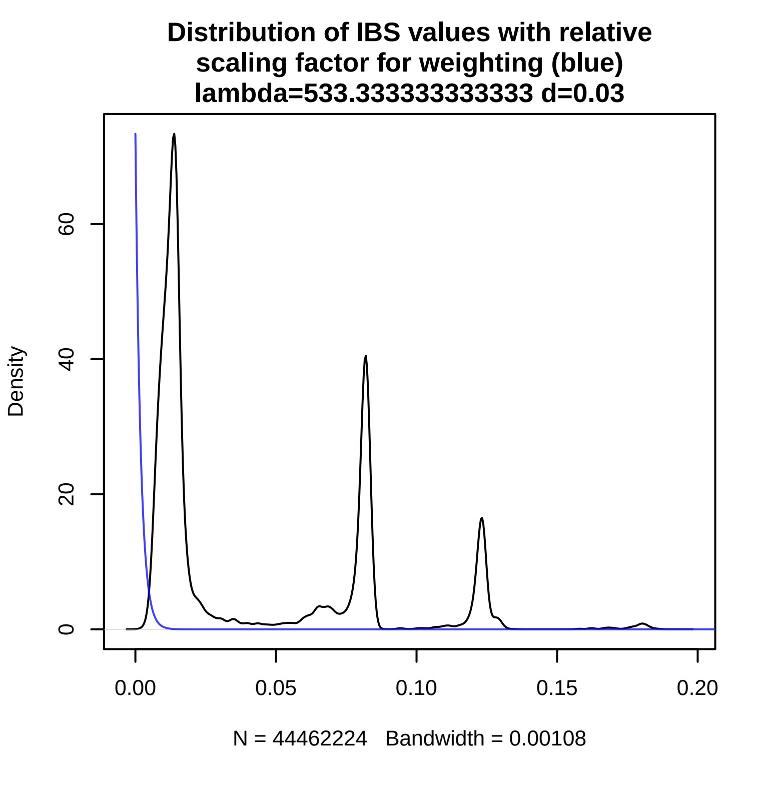
Distribution of IBS values with relative
scaling factor for weighting (blue)
lambda=533.333333333333 d=0.03
Density
N = 44462224 Bandwidth = 0.00108

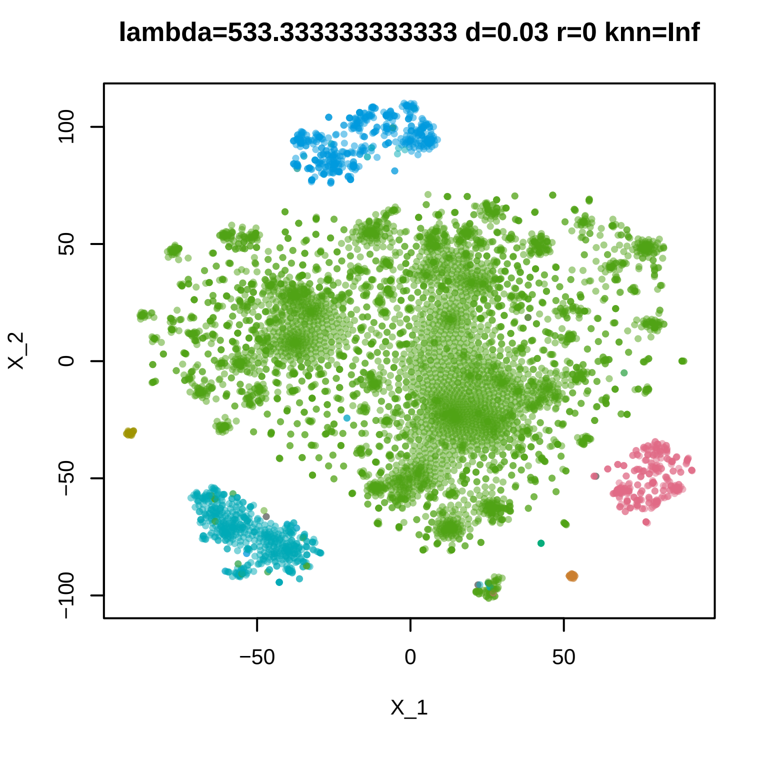
lambda=533.333333333333 d=0.03 r=0 knn=Inf
X_2
X_1

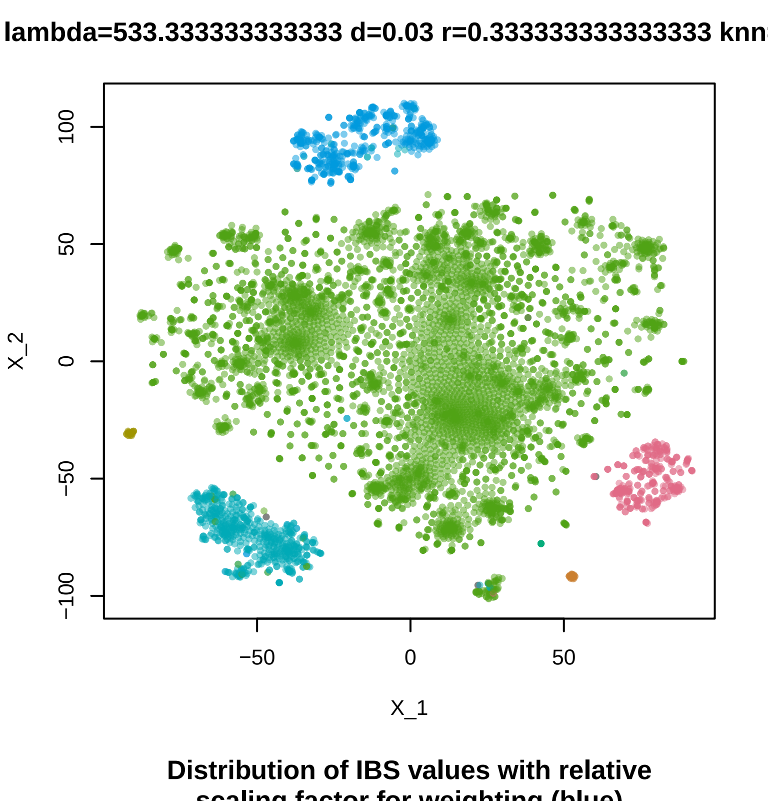
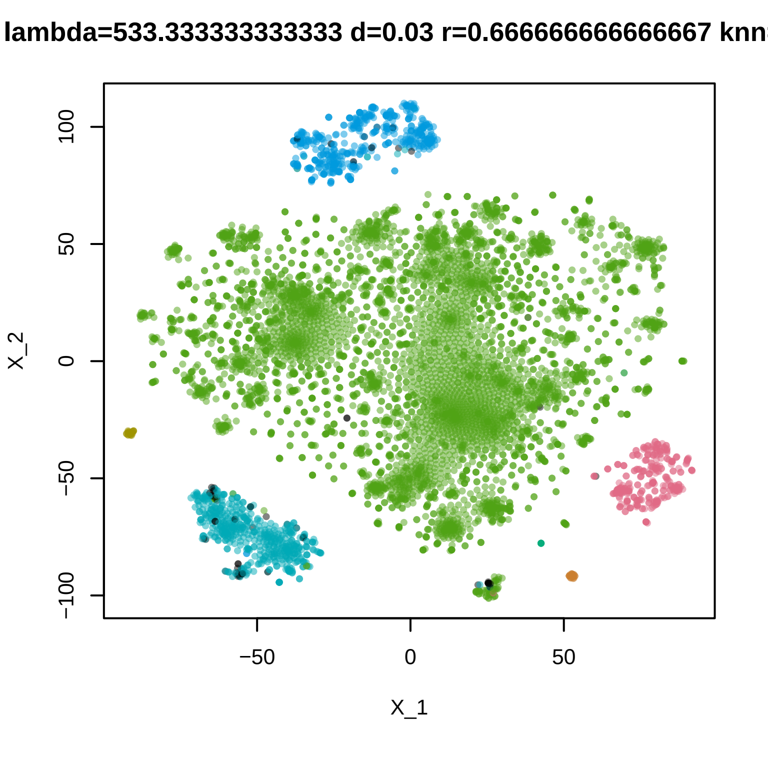
X_2
X_1

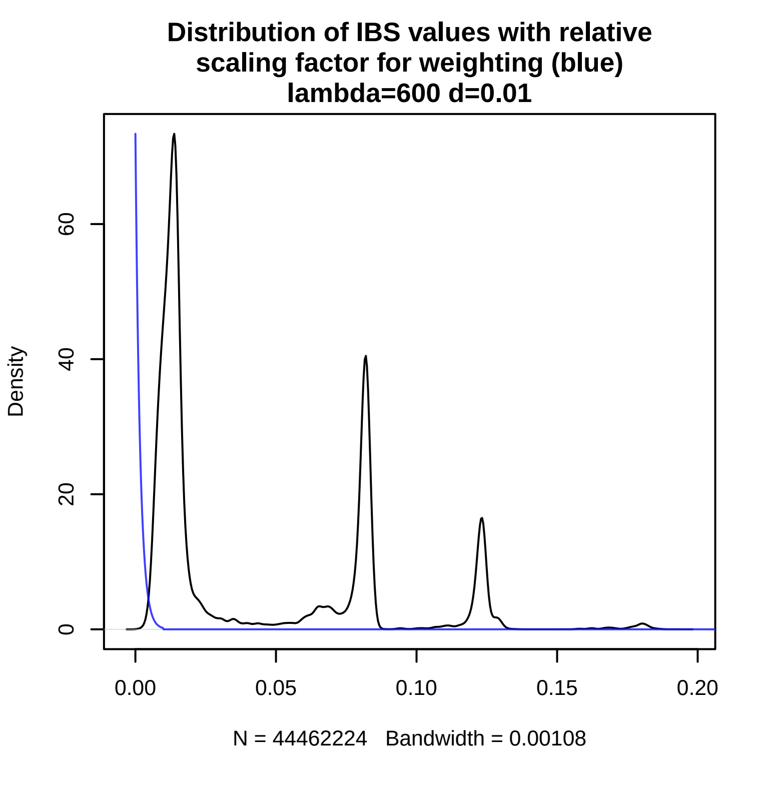
Distribution of IBS values with relative
scaling factor for weighting (blue)
lambda=600 d=0.01
Density
N = 44462224 Bandwidth = 0.00108

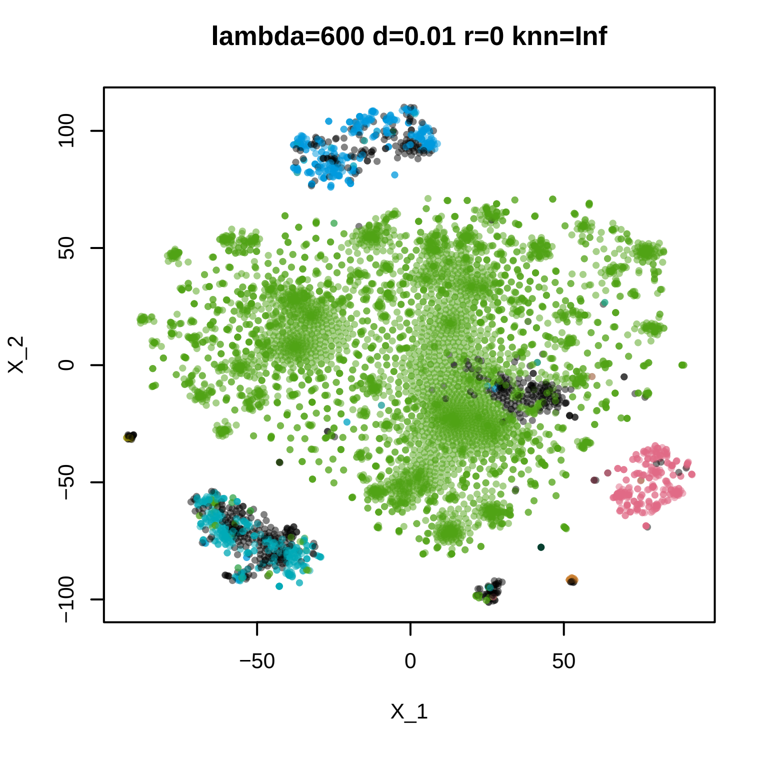
lambda=600 d=0.01 r=0 knn=Inf
X_2
X_1

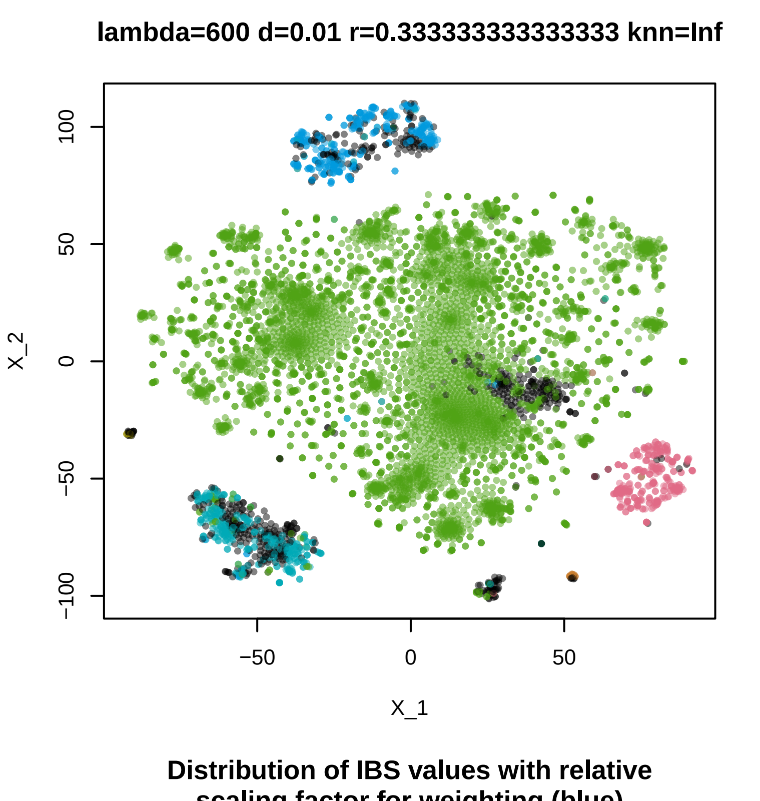
lambda=600 d=0.01 r=0.333333333333333 knn=Inf
X_2
X_1

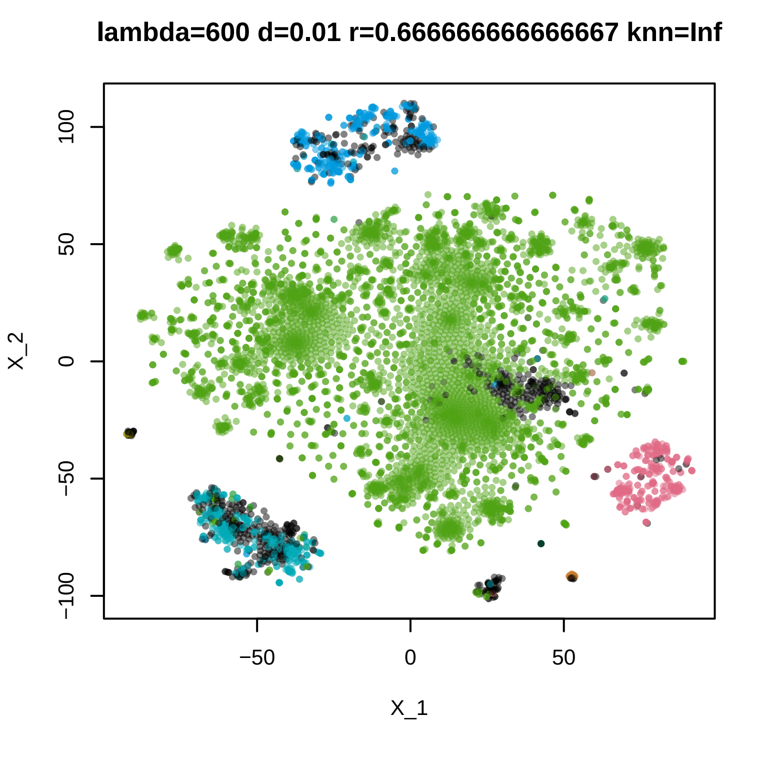
lambda=600 d=0.01 r=0.666666666666667 knn=Inf
X_2
X_1

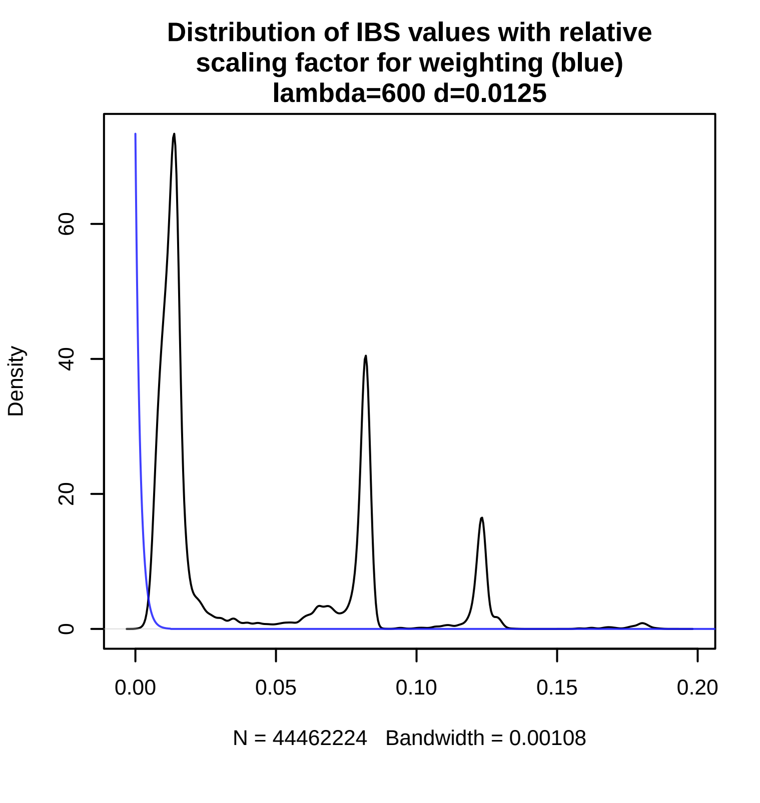
Distribution of IBS values with relative
scaling factor for weighting (blue)
lambda=600 d=0.0125
Density
N = 44462224 Bandwidth = 0.00108

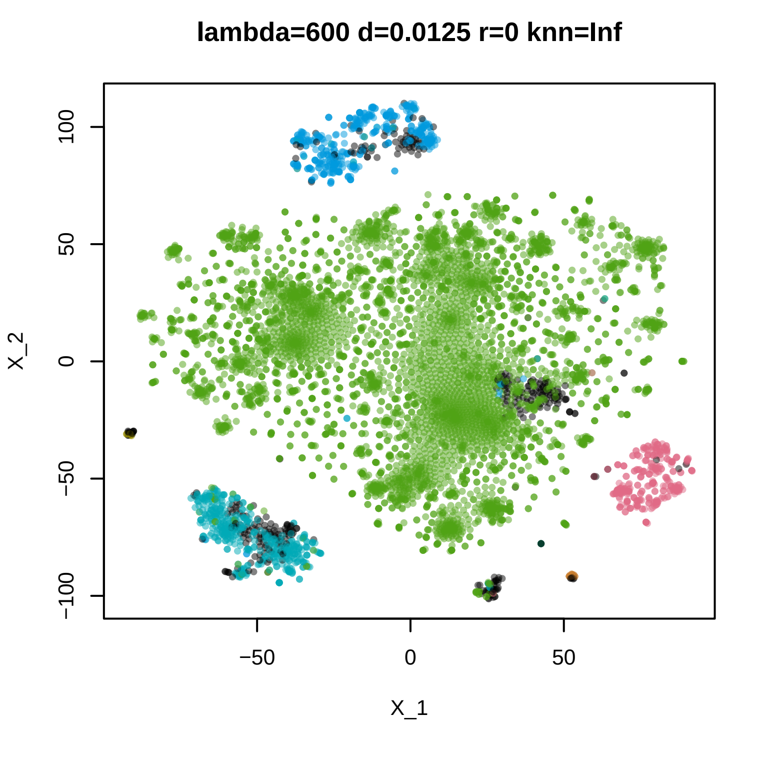
lambda=600 d=0.0125 r=0 knn=Inf
X_2
X_1

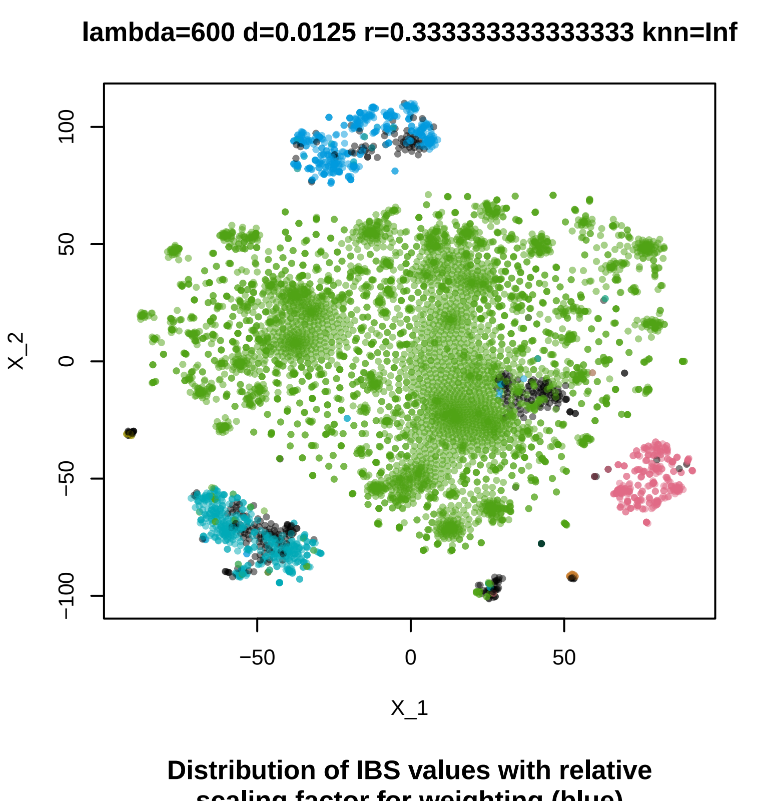
lambda=600 d=0.0125 r=0.333333333333333 knn=Inf
X_2
X_1

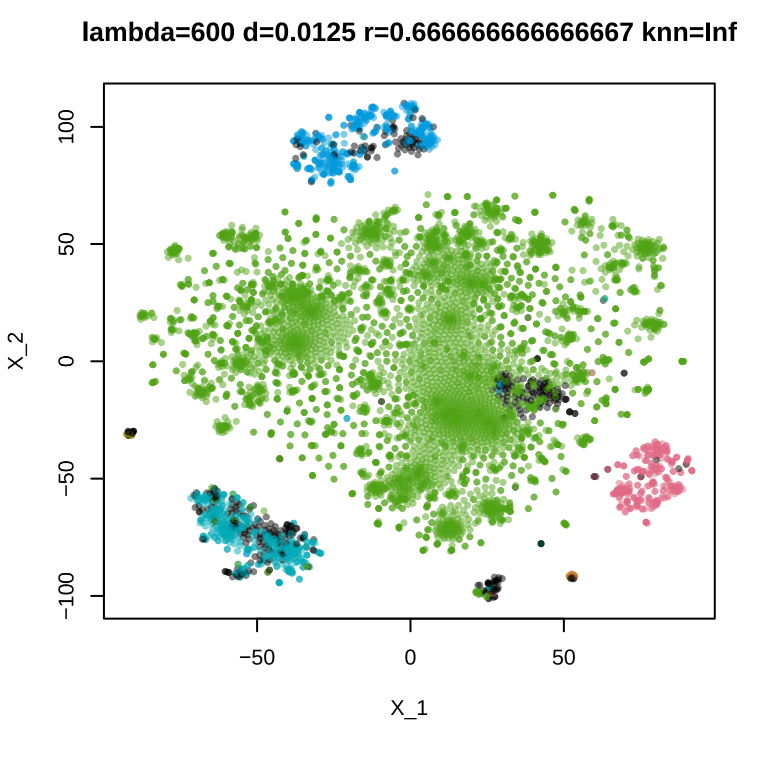
lambda=600 d=0.0125 r=0.666666666666667 knn=Inf
X_2
X_1

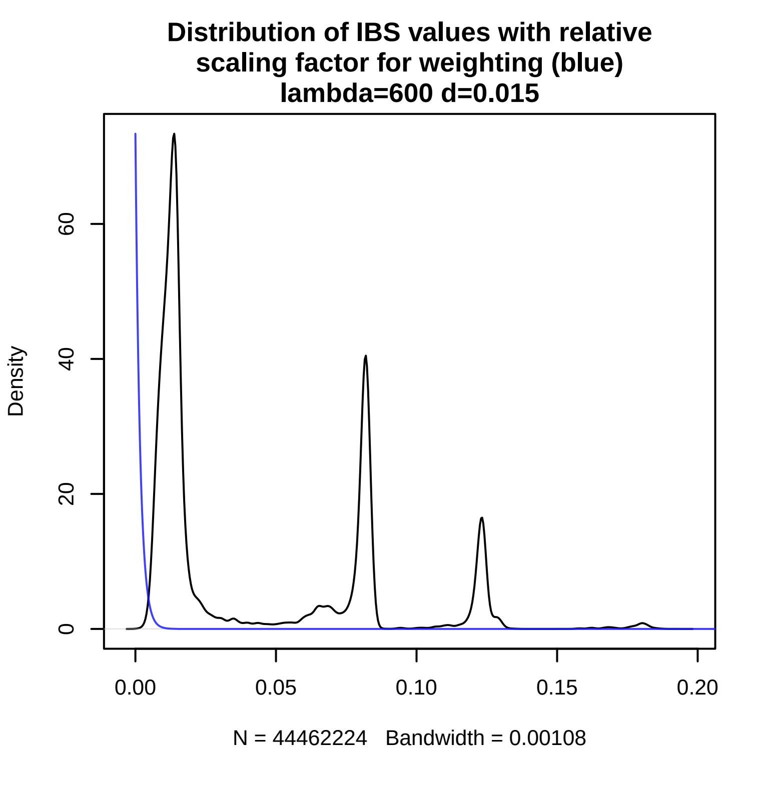
Distribution of IBS values with relative
scaling factor for weighting (blue)
lambda=600 d=0.015
Density
N = 44462224 Bandwidth = 0.00108

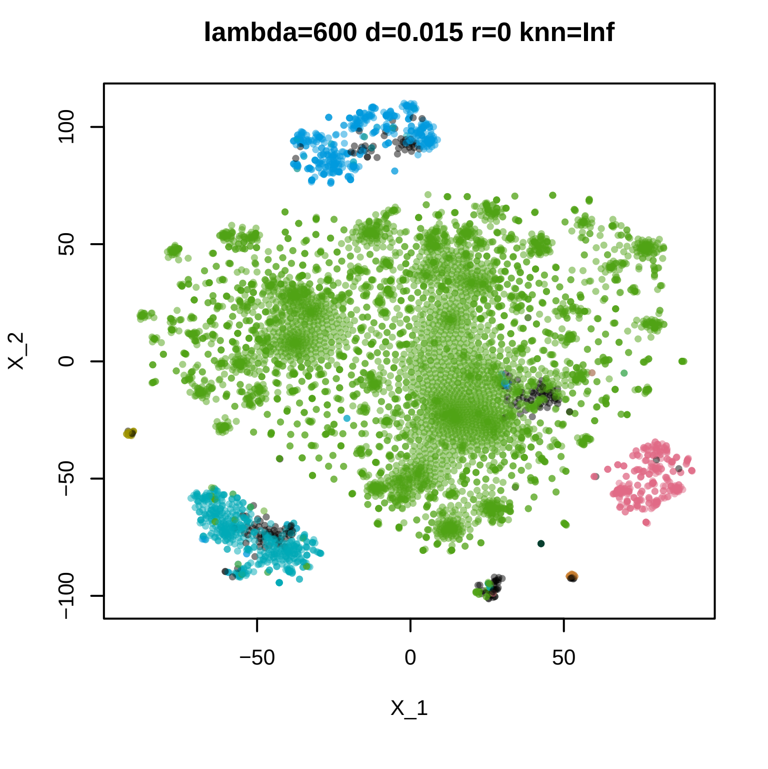
lambda=600 d=0.015 r=0 knn=Inf
X_2
X_1

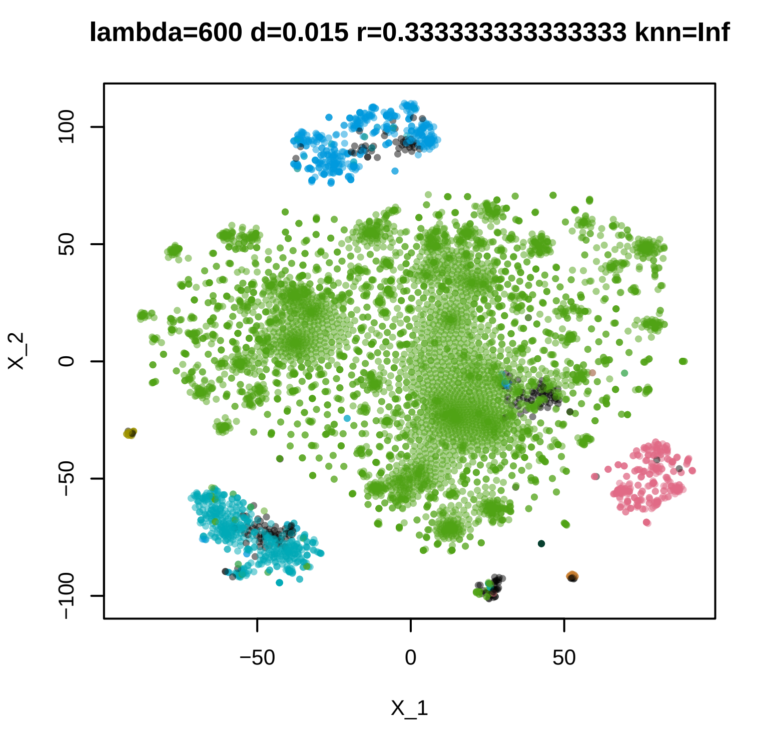
lambda=600 d=0.015 r=0.333333333333333 knn=Inf
X_2
X_1

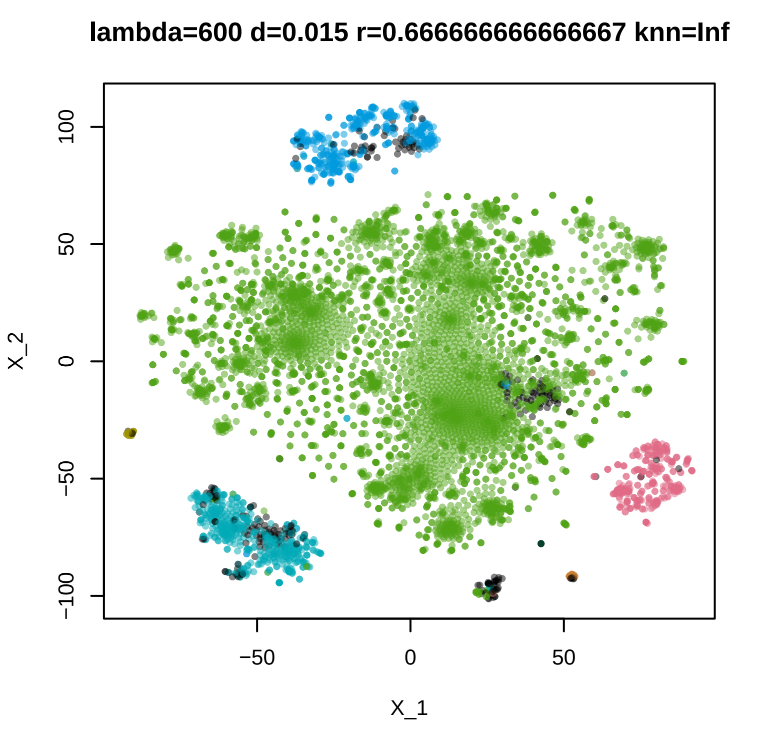
lambda=600 d=0.015 r=0.666666666666667 knn=Inf
X_2
X_1

Distribution of IBS values with relative scaling factor for weighting (blue)
lambda=600 d=0.0175

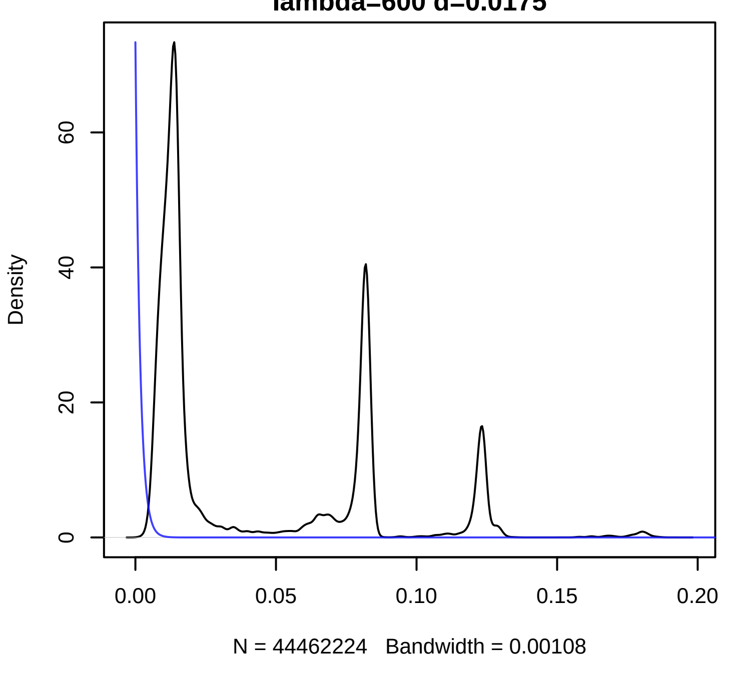

lambda=600 d=0.0175 r=0 knn=Inf

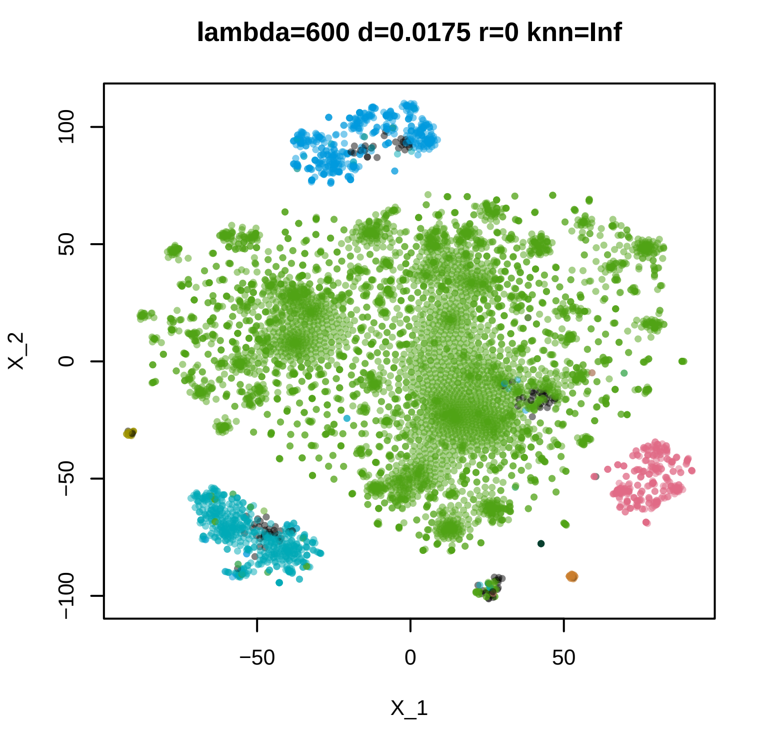

lambda=600 d=0.0175 r=0.333333333333333 knn=Inf

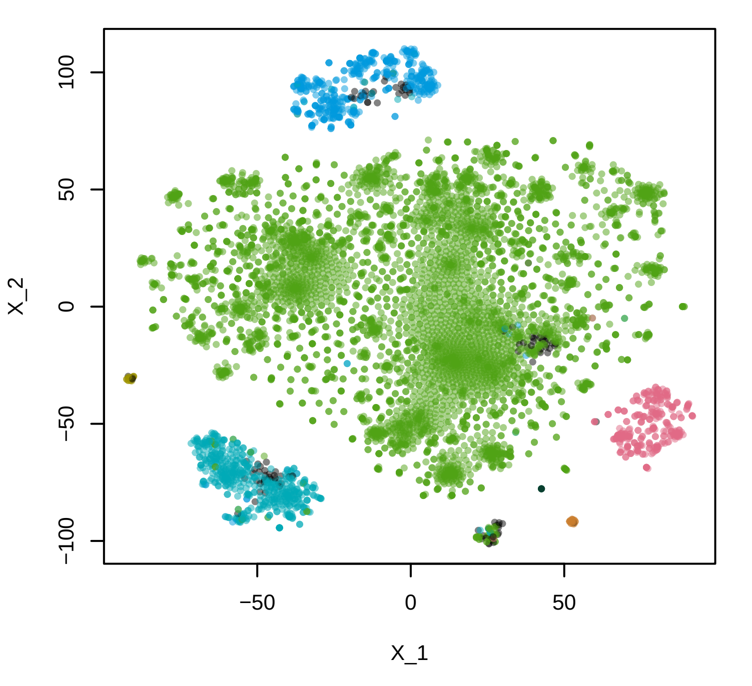

lambda=600 d=0.0175 r=0.666666666666667 knn=Inf

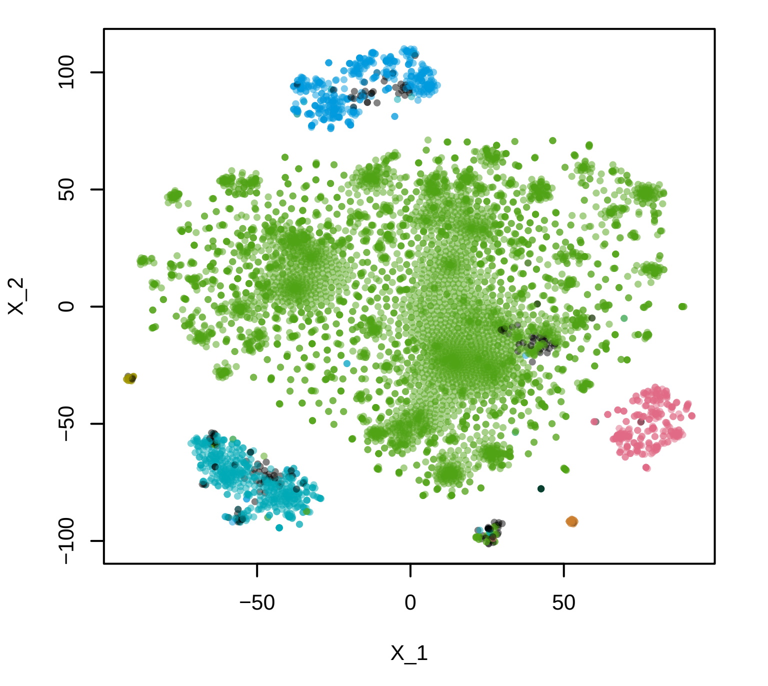

Distribution of IBS values with relative scaling factor for weighting (blue)
lambda=600 d=0.02

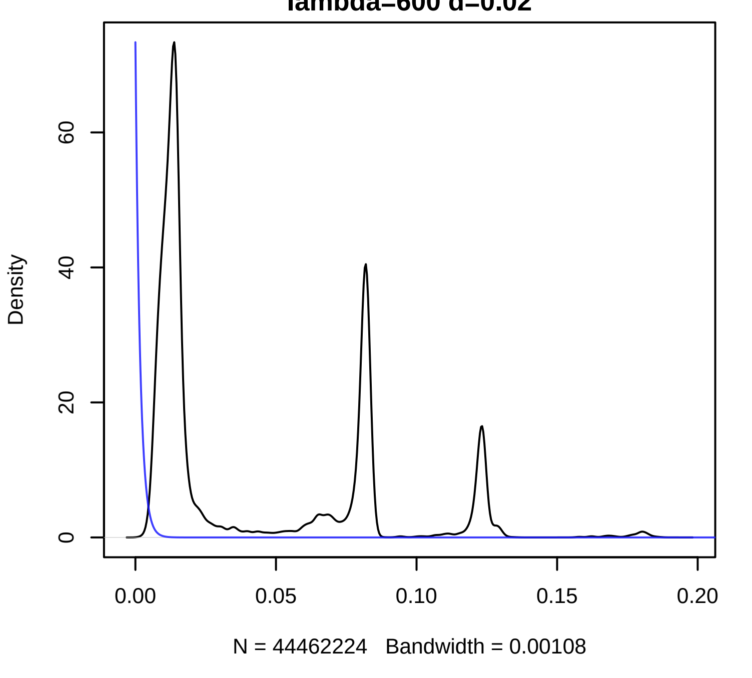

lambda=600 d=0.02 r=0 knn=Inf

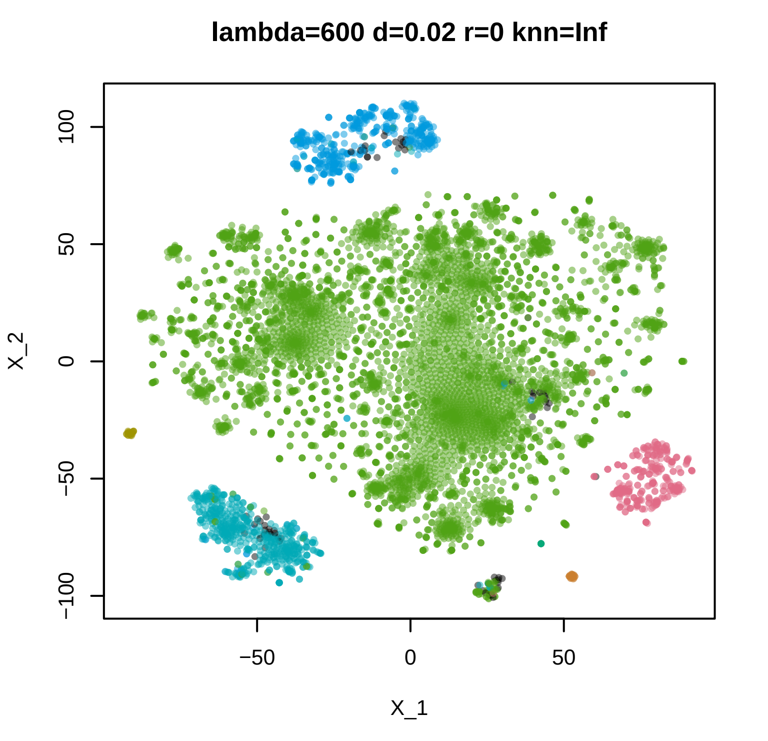

lambda=600 d=0.02 r=0.333333333333333 knn=Inf

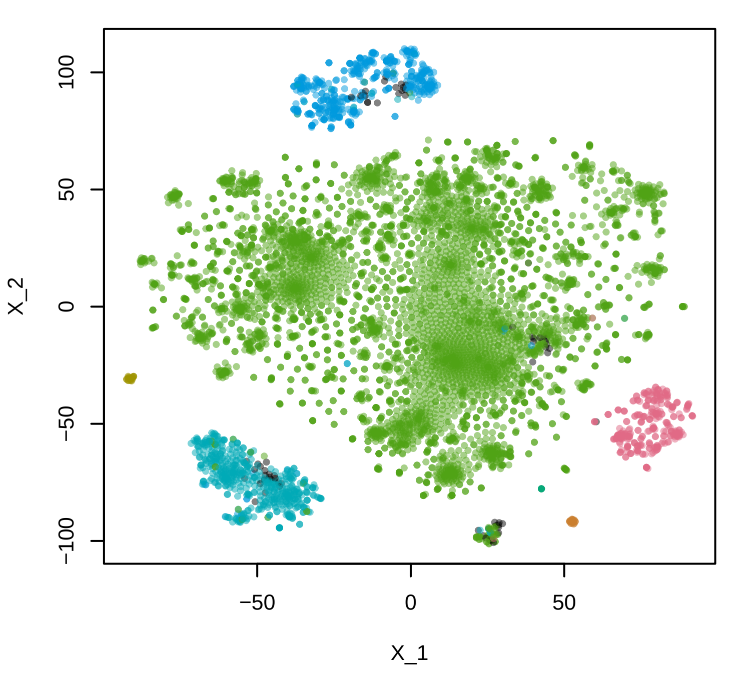

lambda=600 d=0.02 r=0.666666666666667 knn=Inf

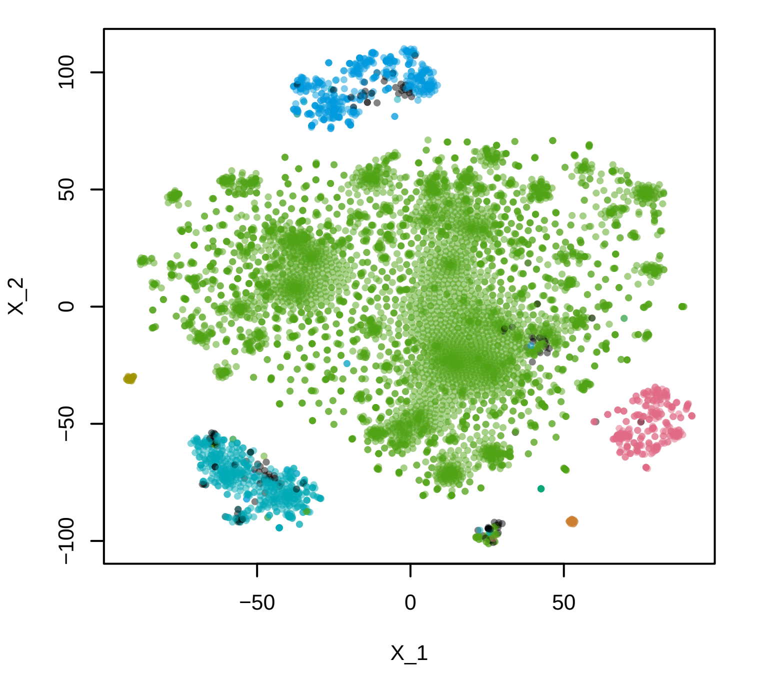

Distribution of IBS values with relative scaling factor for weighting (blue)
lambda=600 d=0.0225

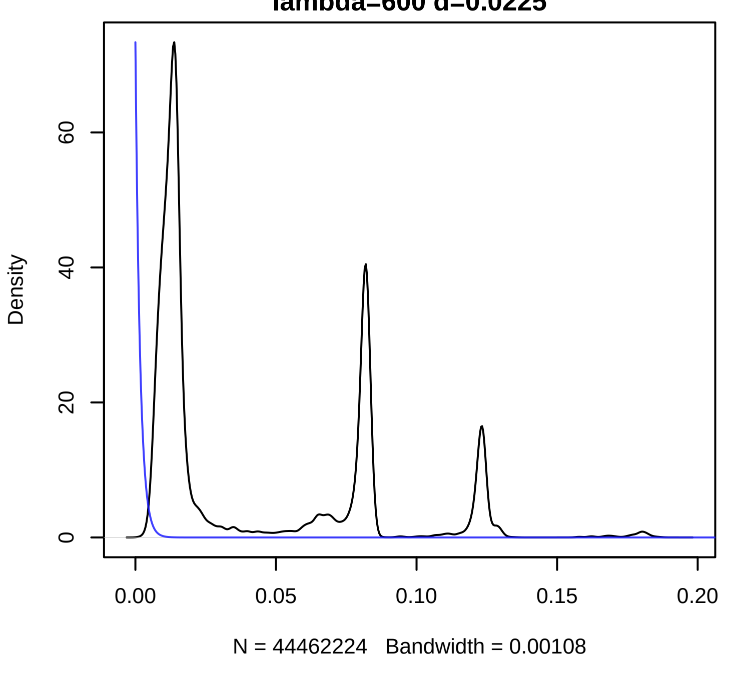

lambda=600 d=0.0225 r=0 knn=Inf

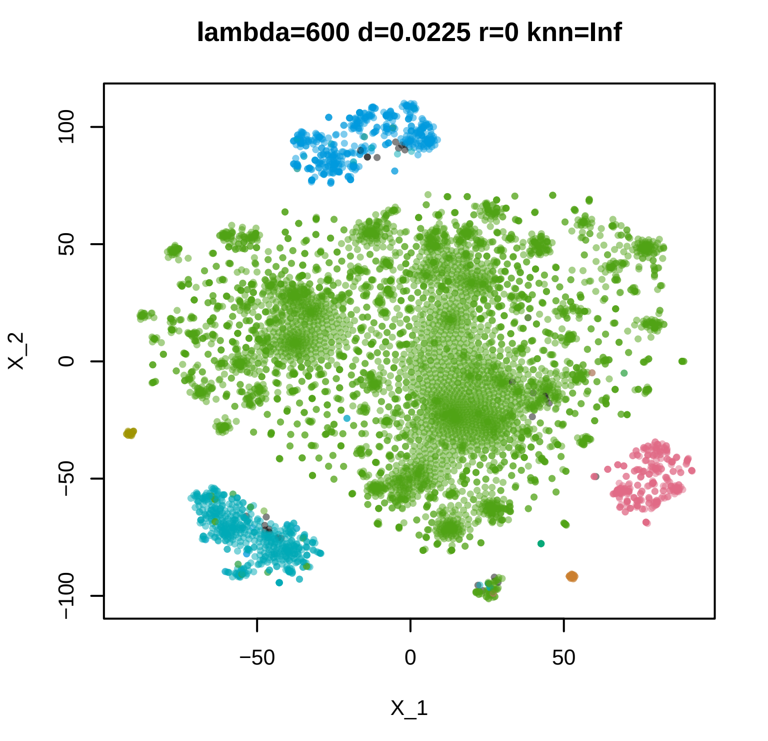

lambda=600 d=0.0225 r=0.333333333333333 knn=Inf

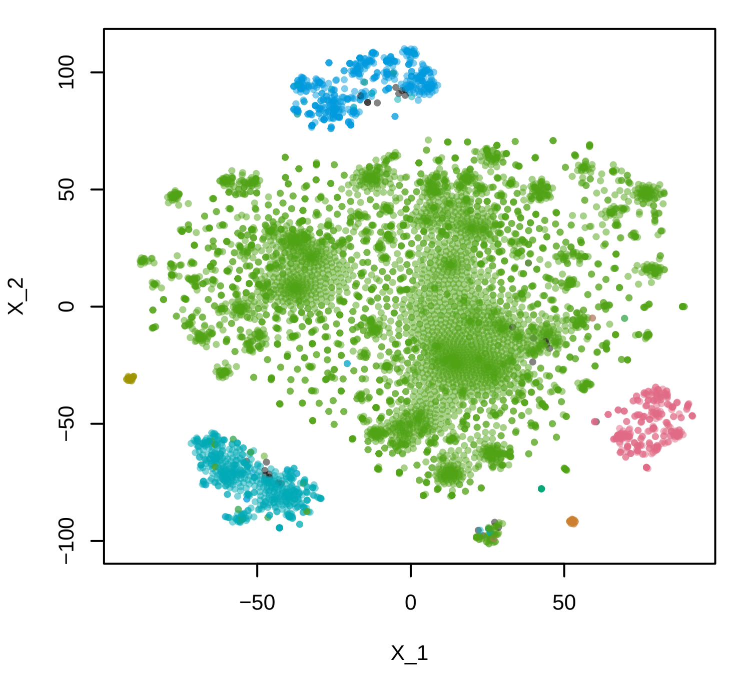

lambda=600 d=0.0225 r=0.666666666666667 knn=Inf

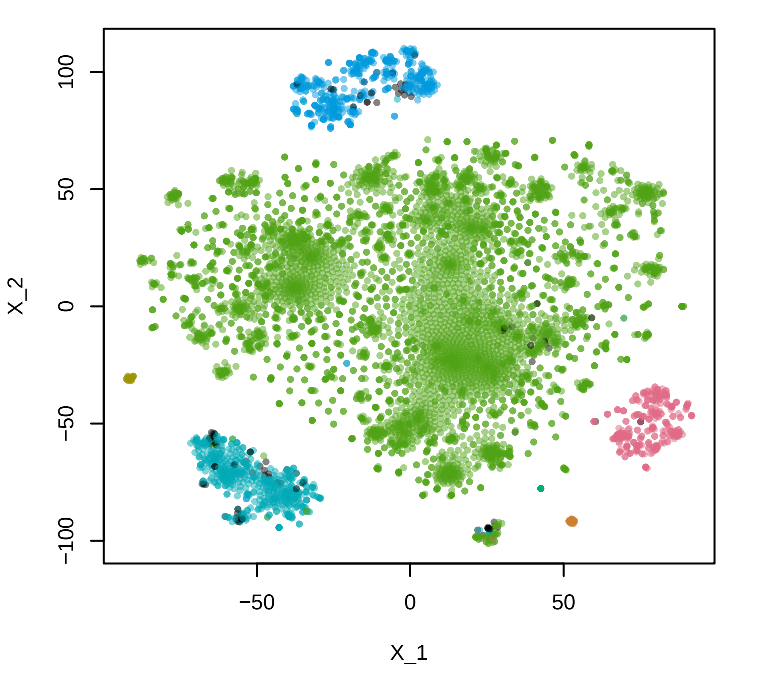

Distribution of IBS values with relative scaling factor for weighting (blue)
lambda=600 d=0.025

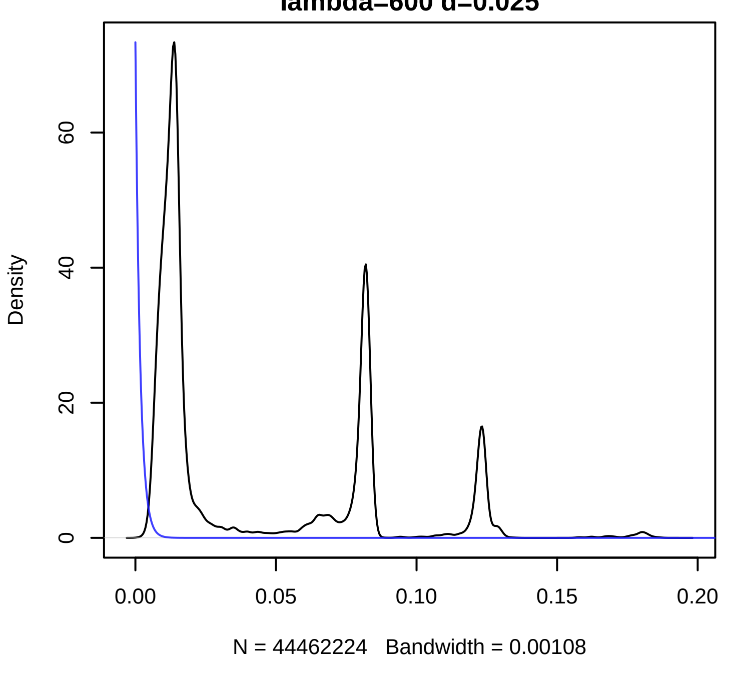

lambda=600 d=0.025 r=0 knn=Inf

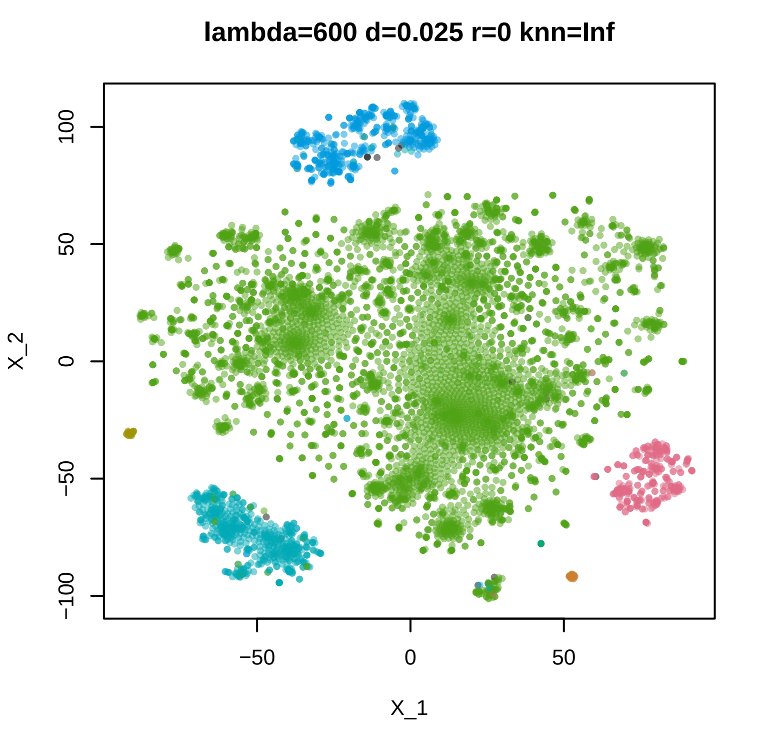

lambda=600 d=0.025 r=0.333333333333333 knn=Inf

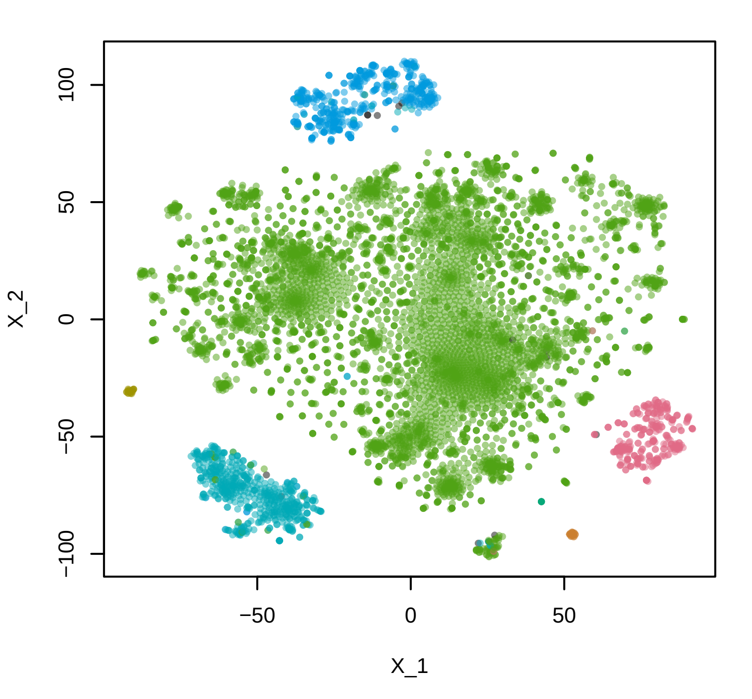

lambda=600 d=0.025 r=0.666666666666667 knn=Inf

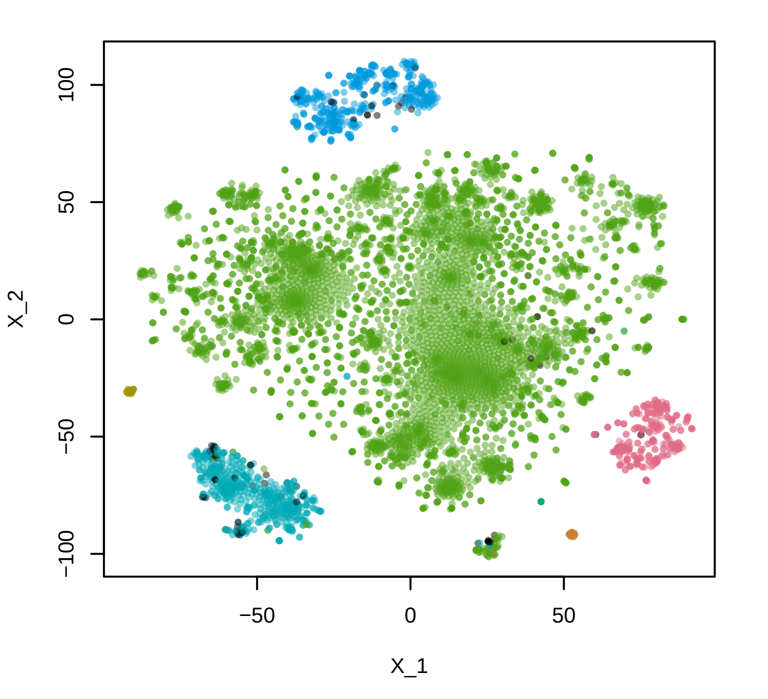

Distribution of IBS values with relative scaling factor for weighting (blue)
lambda=600 d=0.0275

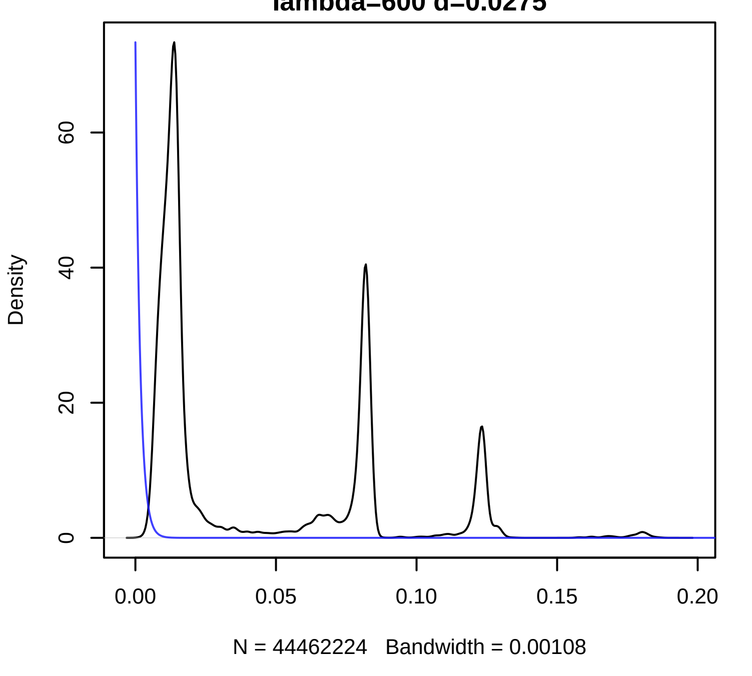

lambda=600 d=0.0275 r=0 knn=Inf

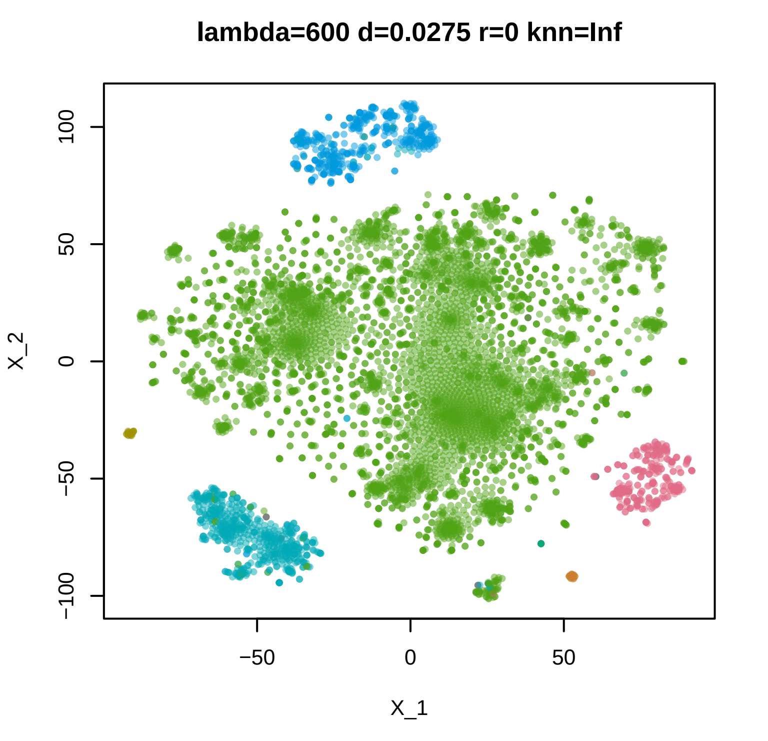

lambda=600 d=0.0275 r=0.333333333333333 knn=Inf

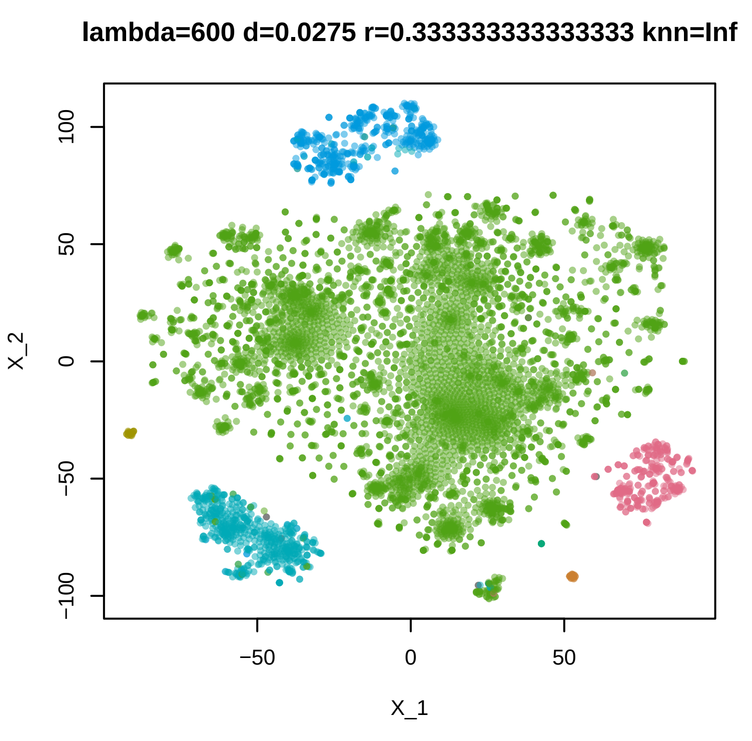

lambda=600 d=0.0275 r=0.666666666666667 knn=Inf

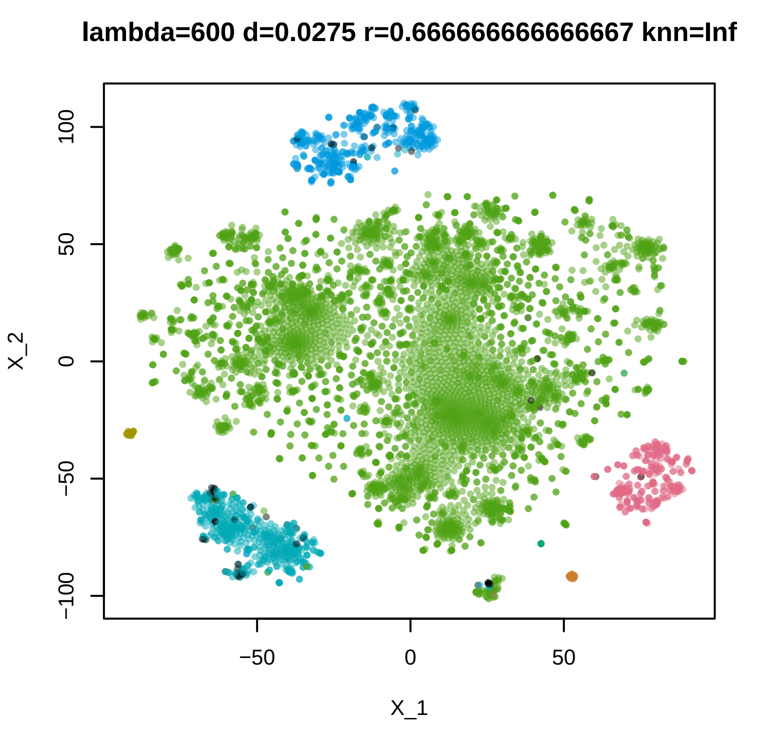

Distribution of IBS values with relative scaling factor for weighting (blue)
lambda=600 d=0.03

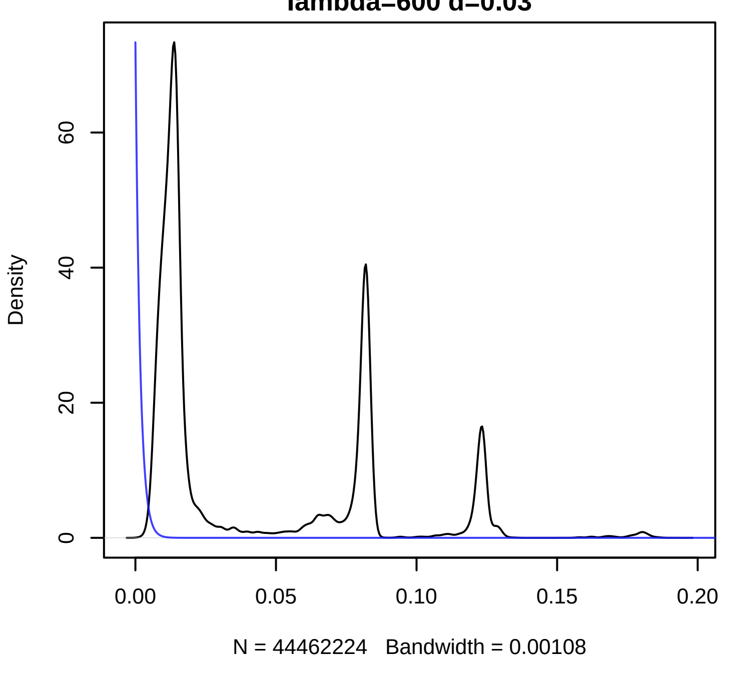

lambda=600 d=0.03 r=0 knn=Inf

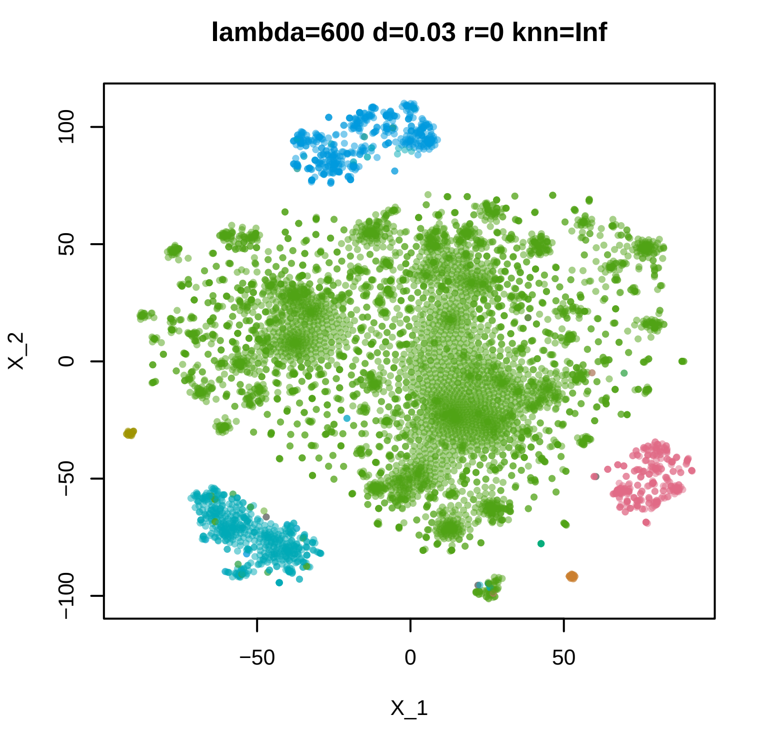

lambda=600 d=0.03 r=0.333333333333333 knn=Inf

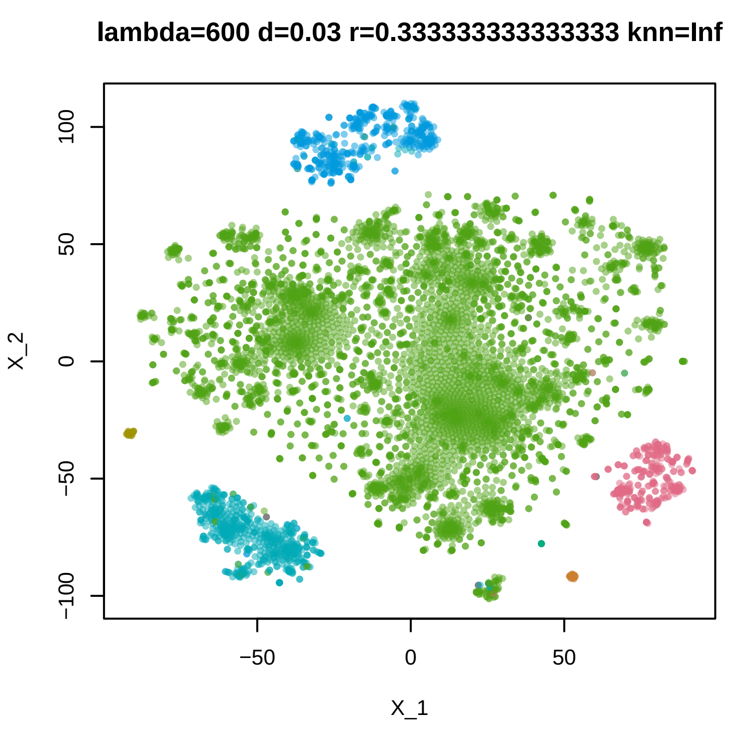

lambda=600 d=0.03 r=0.666666666666667 knn=Inf

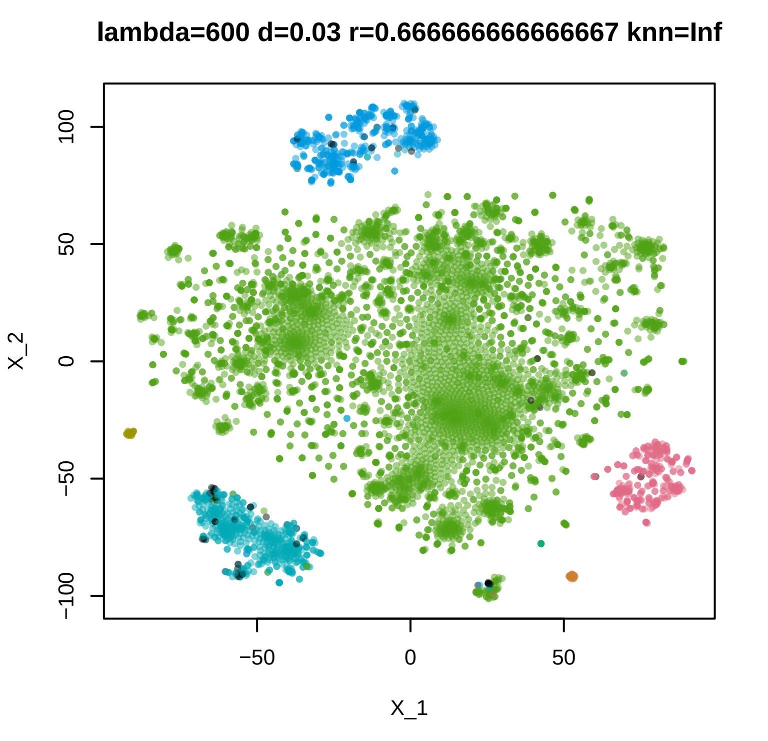

Supplement: Supplementary file 1 — Results of testing multiple parameter combinations on the classification of samples in the G2P-Sol Capsicum dataset using the kernel-based method presented in this study (PDF 101797 kb) [file 122_2023_4441_MOESM1_ESM.pdf]
