# Supplementary material for: Accurate, automated taxonomic assignment of genebank accessions: a new method demonstrated using high-throughput marker data from 10,000 Capsicum spp. accessions
Source: Theor Appl Genet. 2023 Sep 11;136(10):208. doi: 10.1007/s00122-023-04441-8 (PMC10495273; doi:10.1007/s00122-023-04441-8)

Rabanus-Wallace 2022

Supplementary Data S2

Description

Panels of each row represent the results of taxonomic reclassification of the G2P pepper dataset using the k-nearest neighbour method, with varying values of *k* and *r*, visualised using the t-SNE procedure with perplexity 30, as per main text figure 1. Colours as per figure 1, reproduced in the legend below.

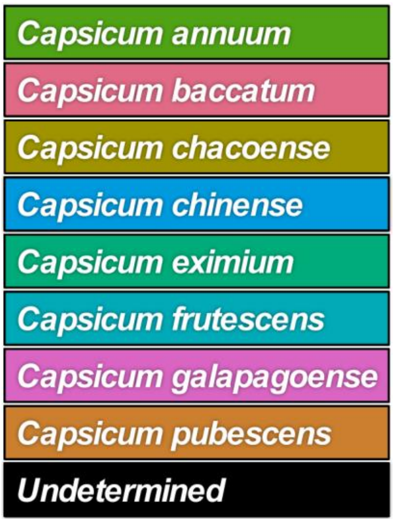

lambda=0 d=Inf r=0 knn=2

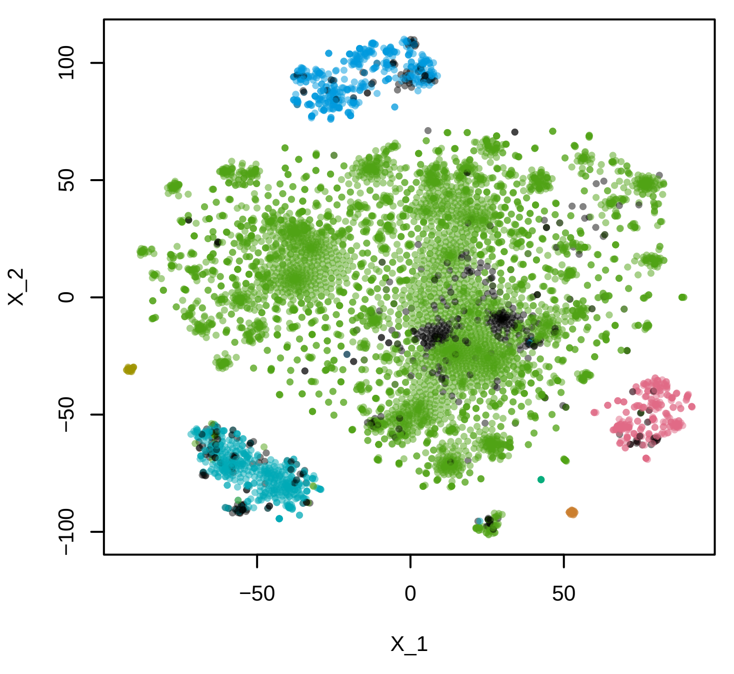

lambda=0 d=Inf r=0.333333333333333 knn=2

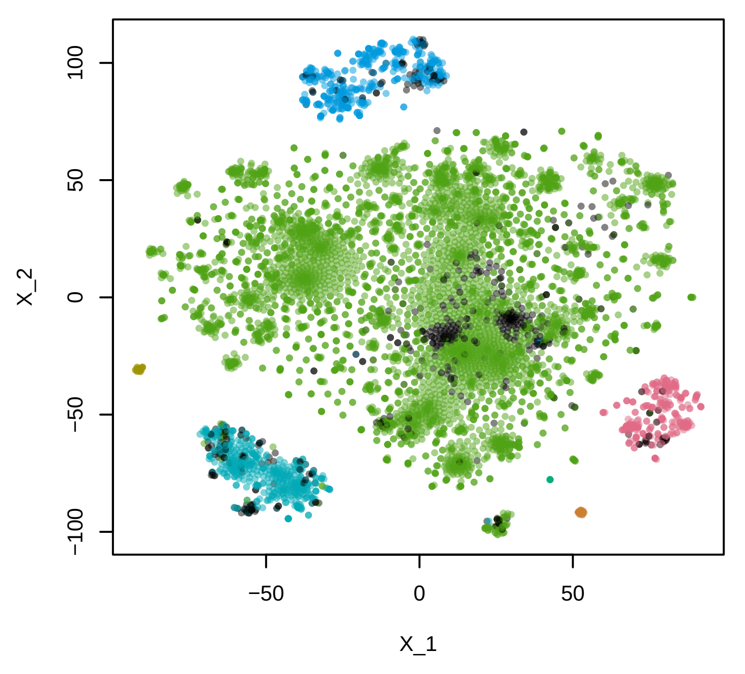

lambda=0 d=Inf r=0.666666666666667 knn=2

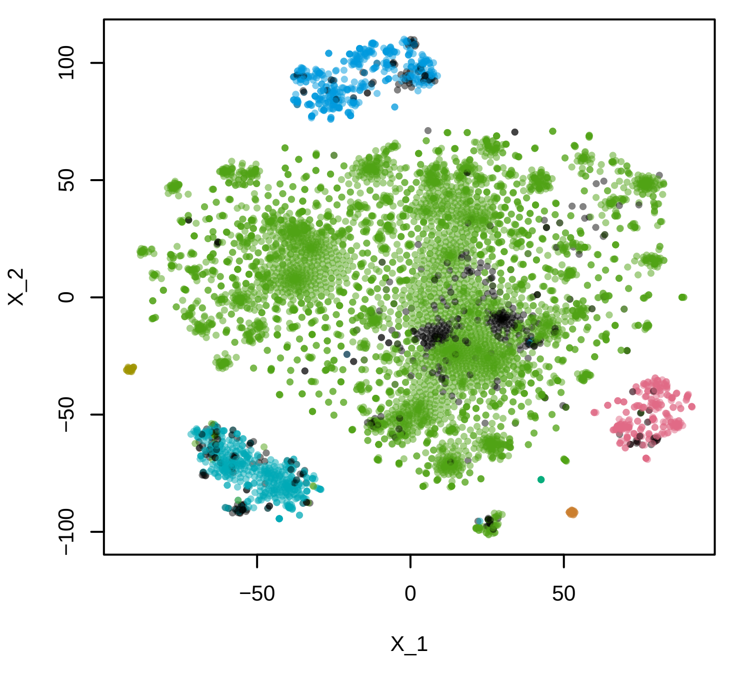

lambda=0 d=Inf r=0 knn=3

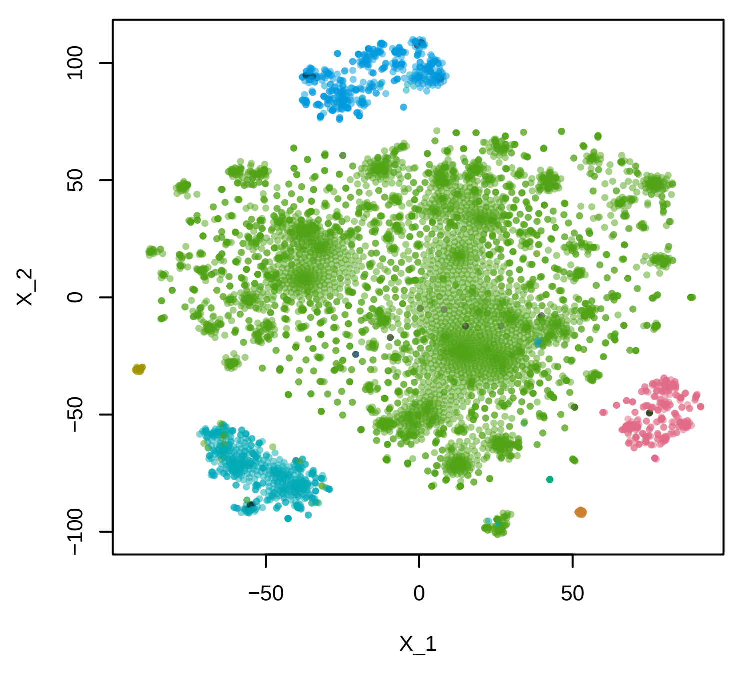

lambda=0 d=Inf r=0.333333333333333 knn=3

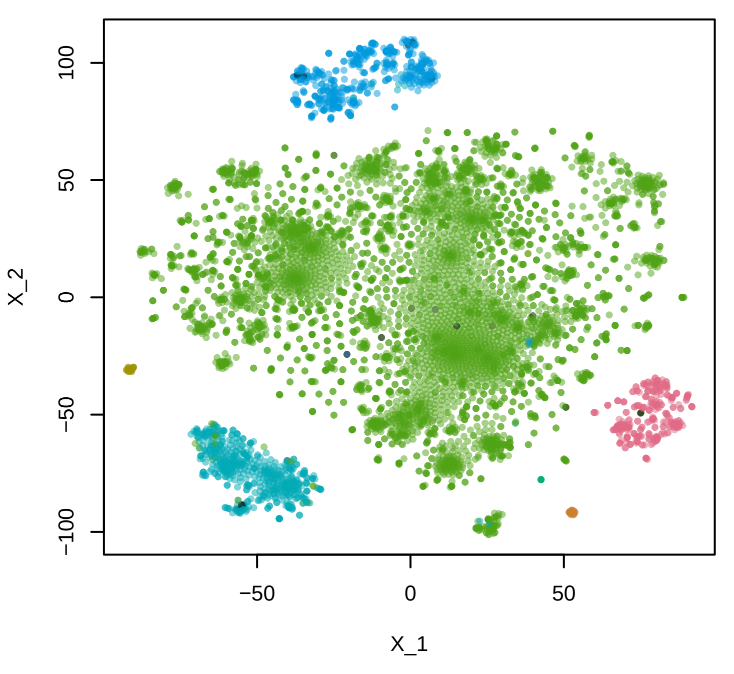

lambda=0 d=Inf r=0.666666666666667 knn=3

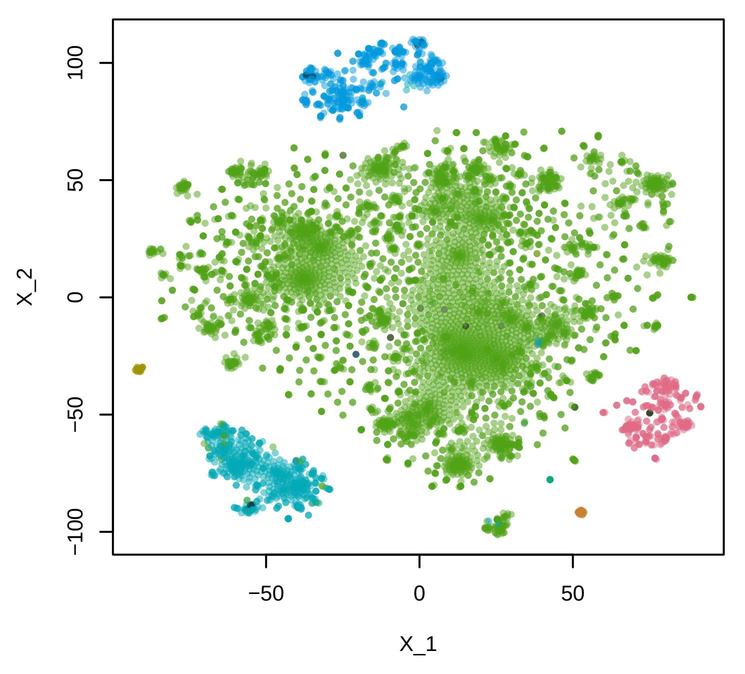

lambda=0 d=Inf r=0 knn=5

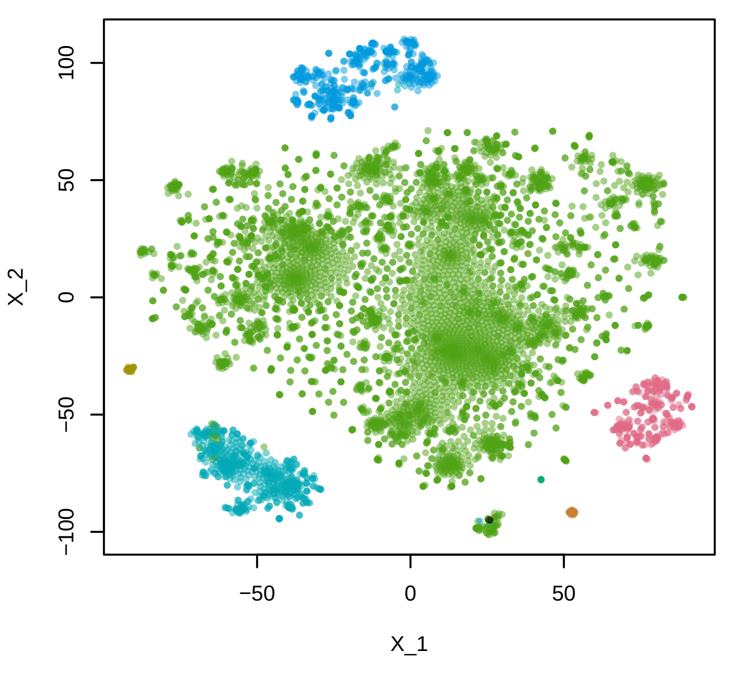

lambda=0 d=Inf r=0.333333333333333 knn=5

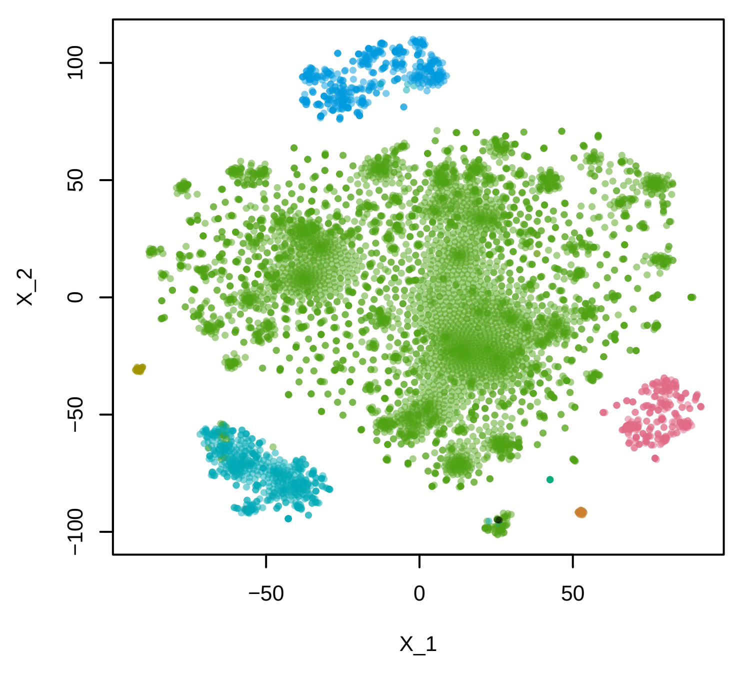

lambda=0 d=Inf r=0.666666666666667 knn=5

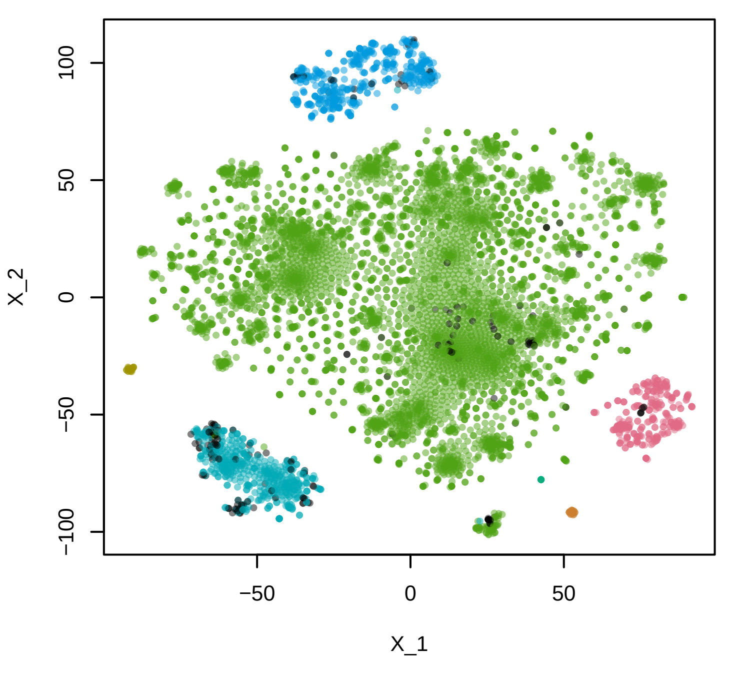

lambda=0 d=Inf r=0 knn=6

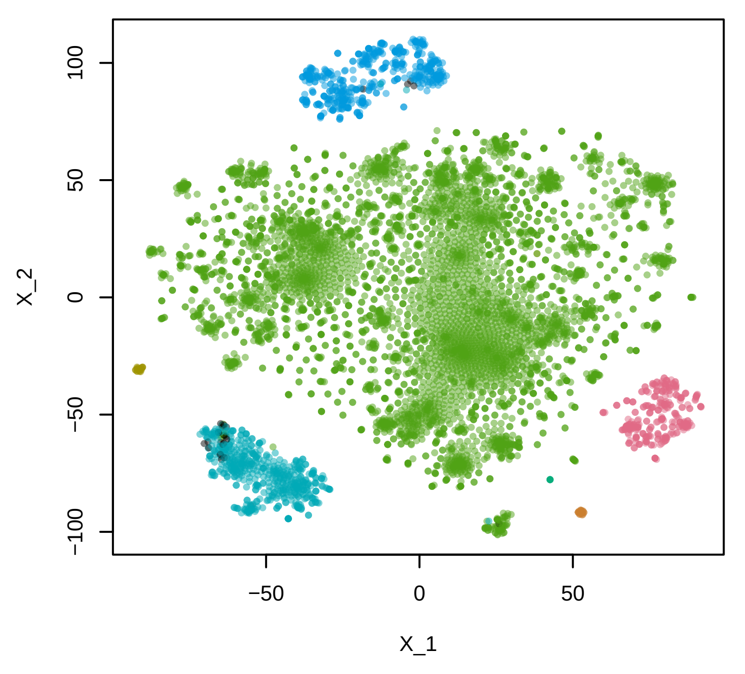

lambda=0 d=Inf r=0.333333333333333 knn=6

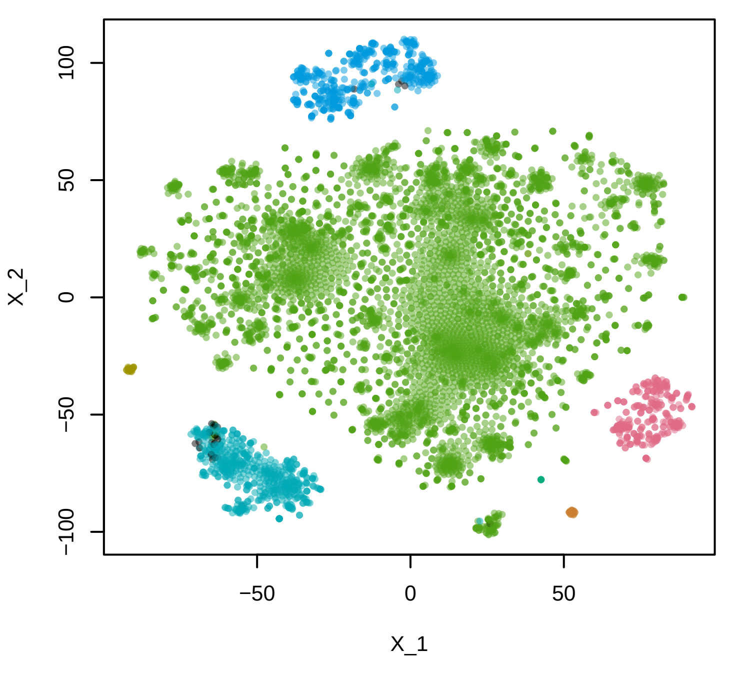

lambda=0 d=Inf r=0.666666666666667 knn=6

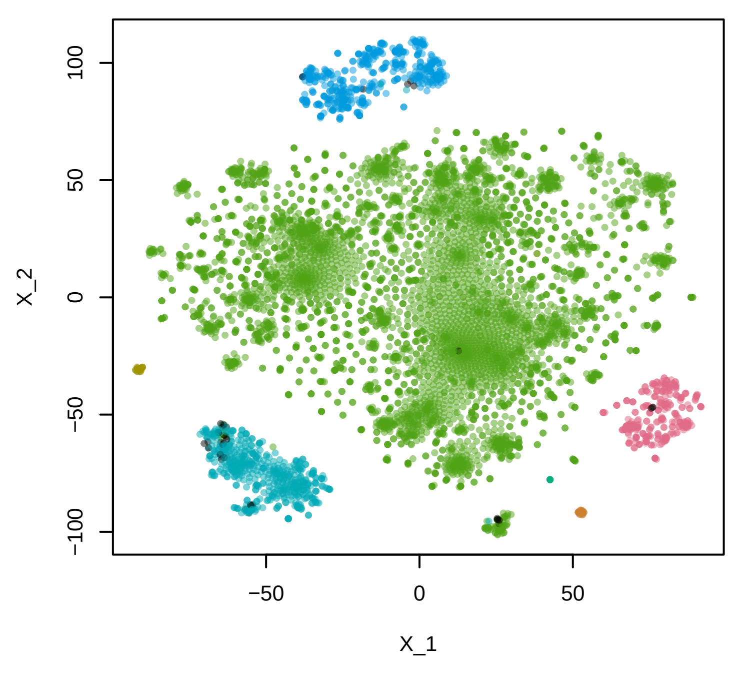

lambda=0 d=Inf r=0 knn=7

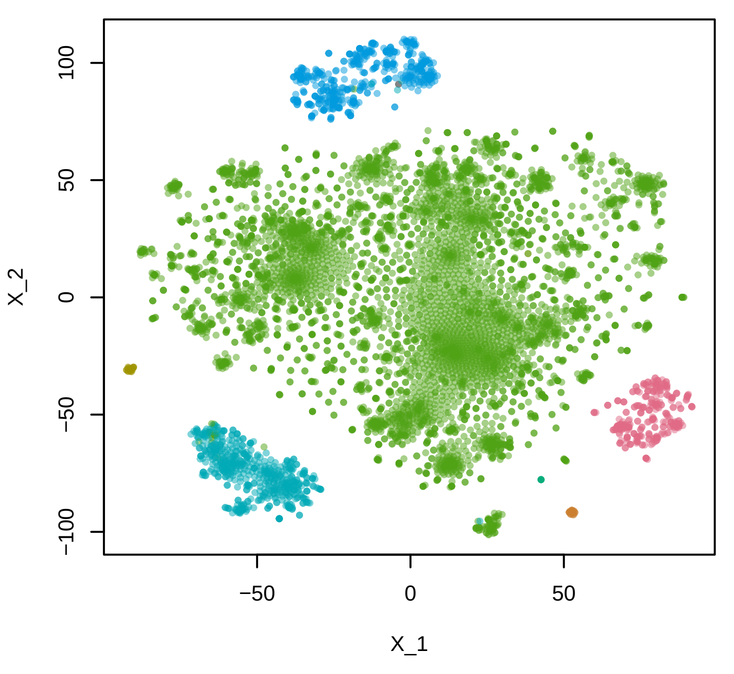

lambda=0 d=Inf r=0.333333333333333 knn=7

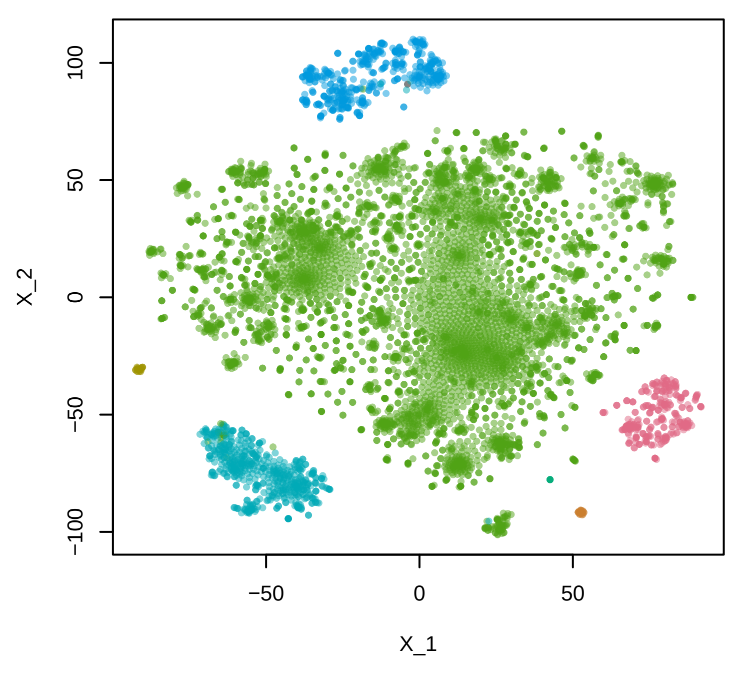

lambda=0 d=Inf r=0.666666666666667 knn=7

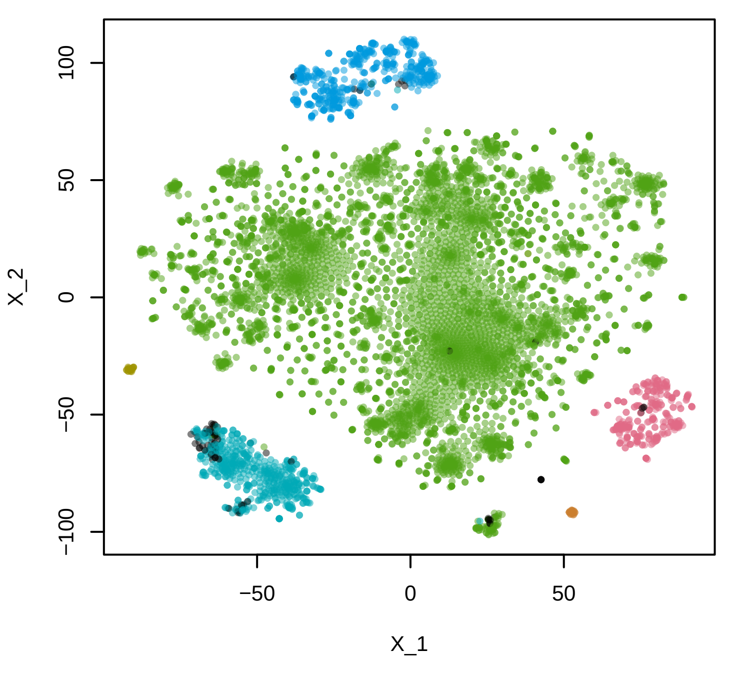

lambda=0 d=Inf r=0 knn=10

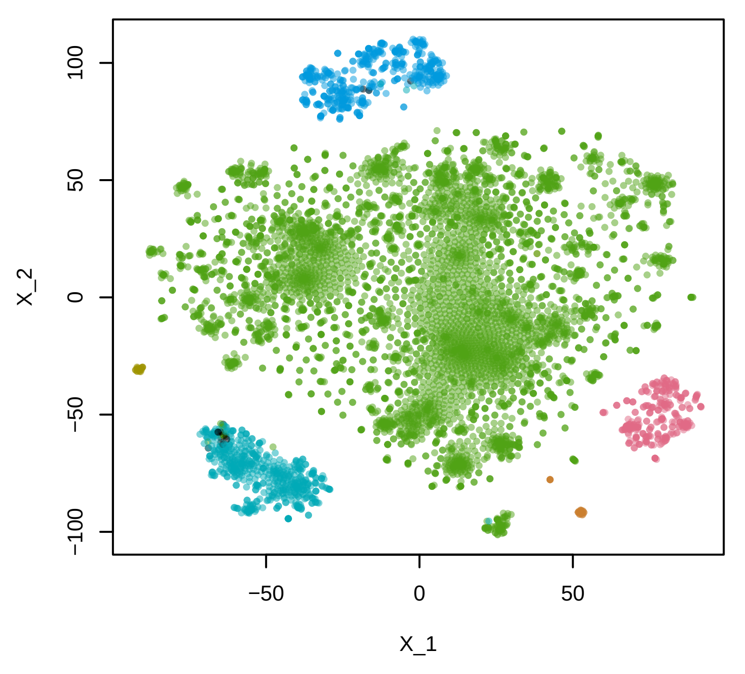

lambda=0 d=Inf r=0.333333333333333 knn=10

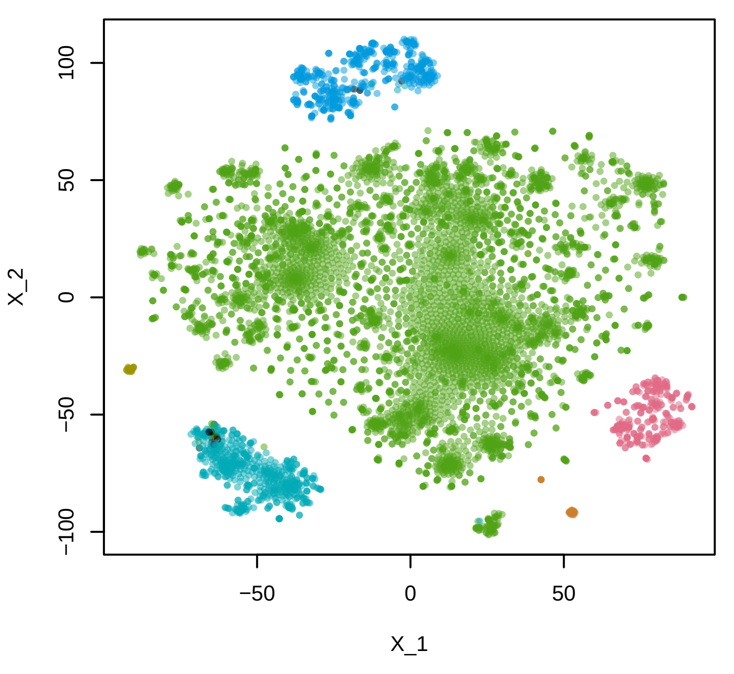

lambda=0 d=Inf r=0.666666666666667 knn=10

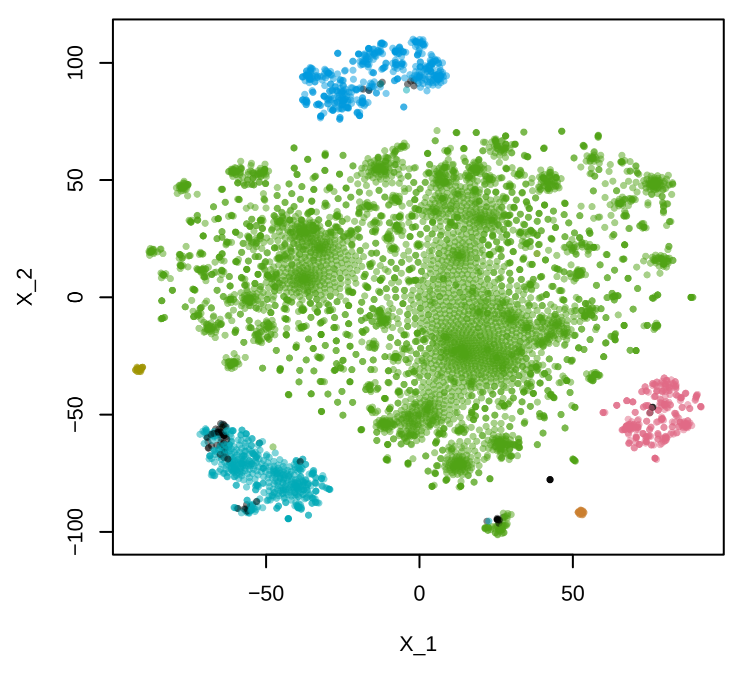

lambda=0 d=Inf r=0 knn=20

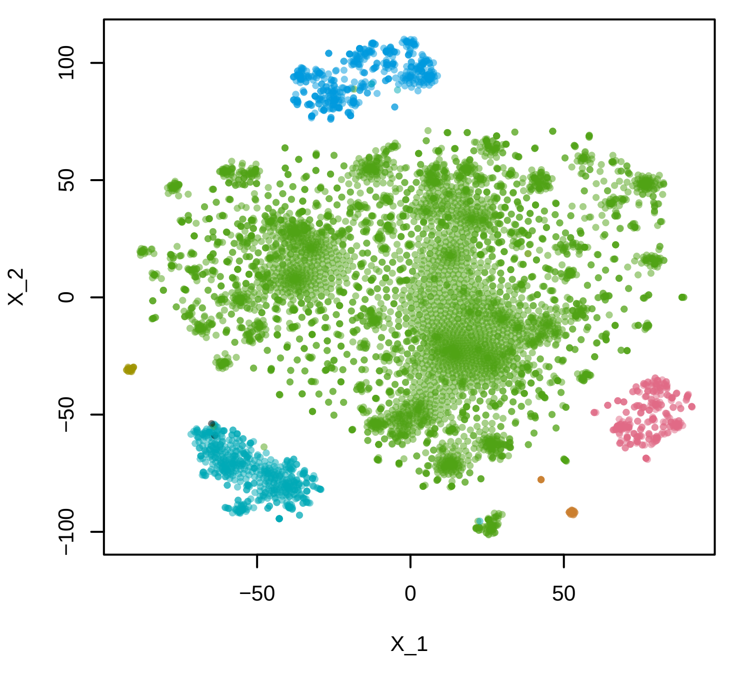

lambda=0 d=Inf r=0.333333333333333 knn=20

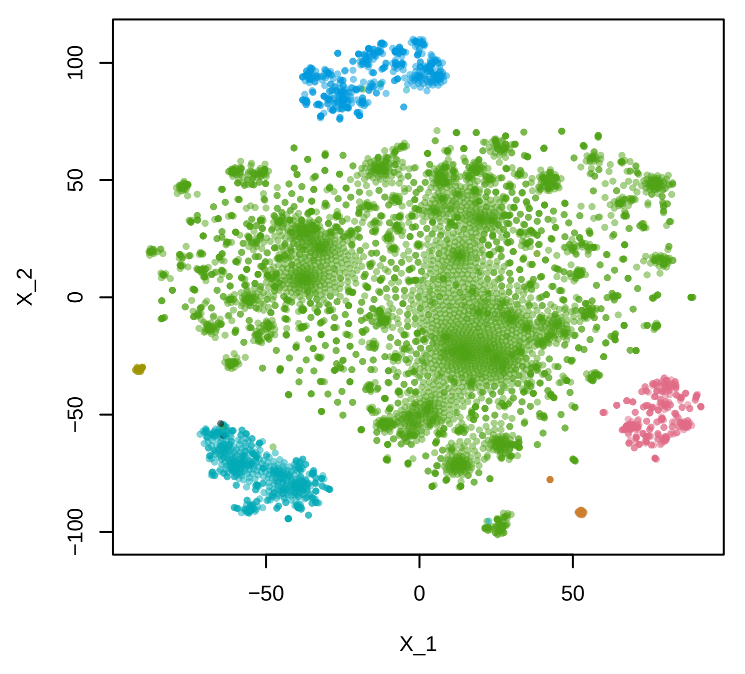

lambda=0 d=Inf r=0.666666666666667 knn=20

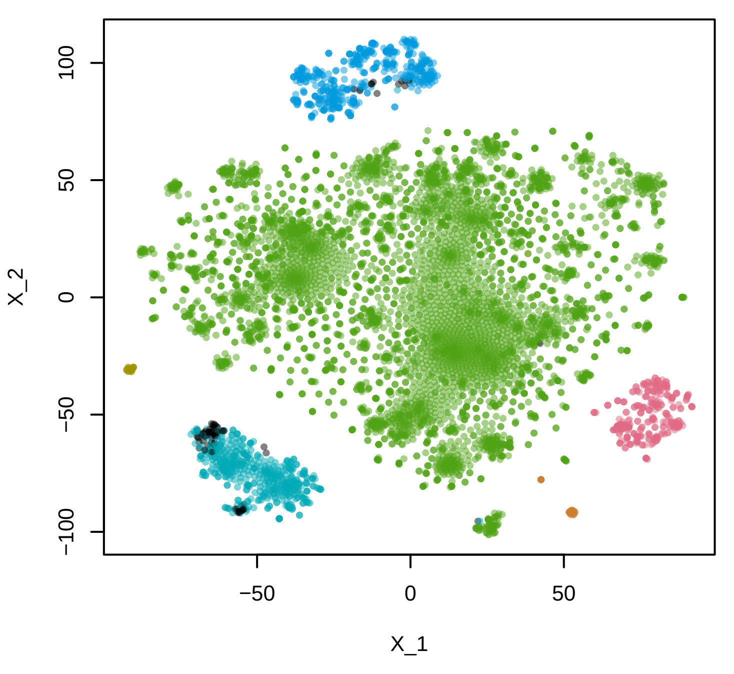

lambda=0 d=Inf r=0 knn=100

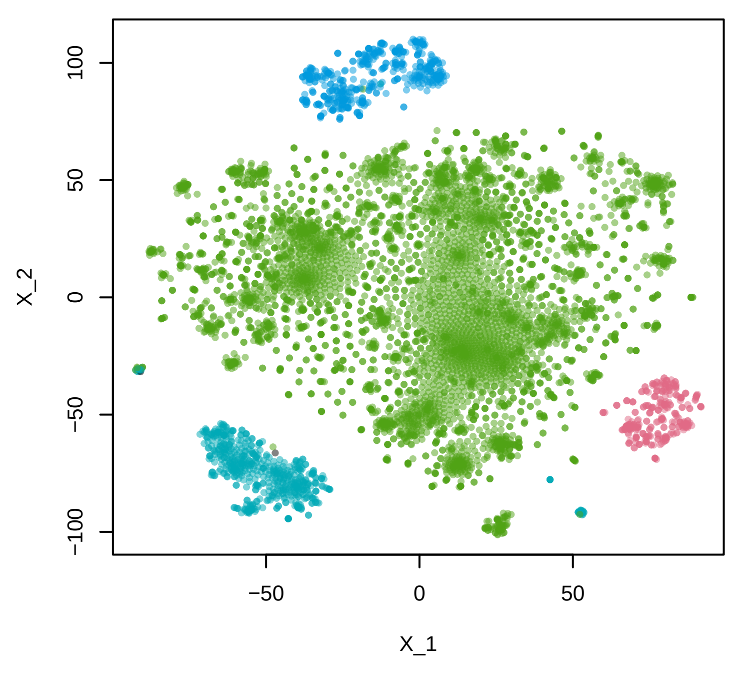

lambda=0 d=Inf r=0.333333333333333 knn=100

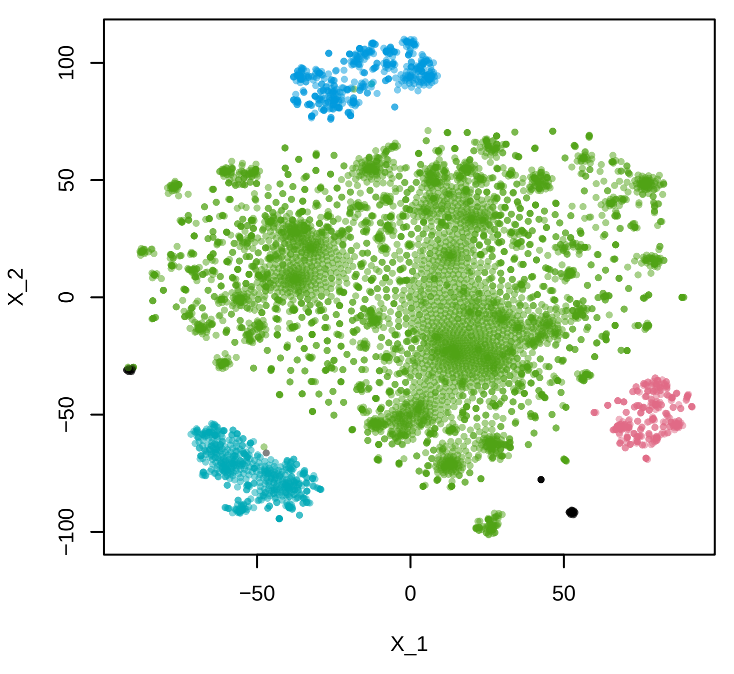

lambda=0 d=Inf r=0.666666666666667 knn=100

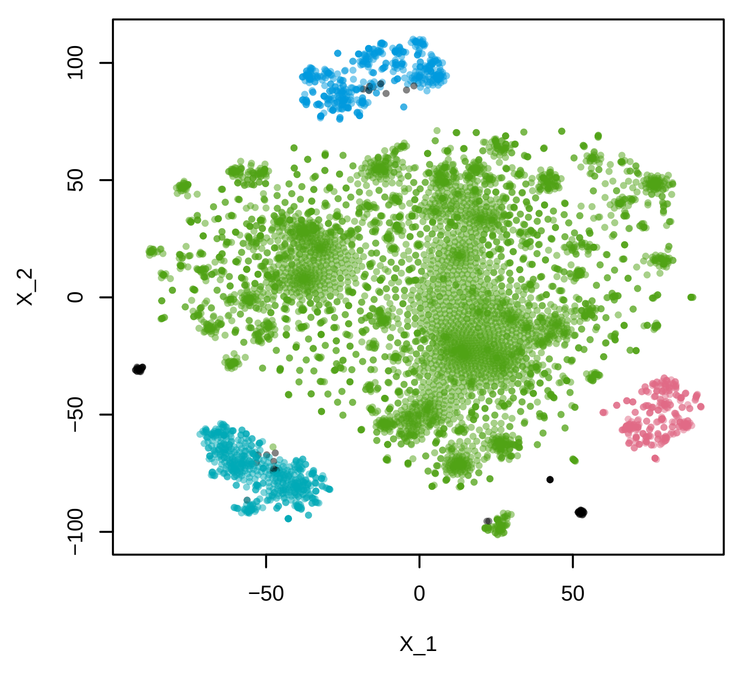

Supplement: Supplementary file 2 — As per S1, but showing results of k-nearest-neighbour classification with different parameter values for k (PDF 9028 kb) [file 122_2023_4441_MOESM2_ESM.pdf]
